# A multi-omics Mendelian randomization study reveals PAM as a potential therapeutic target for type 2 diabetes

**DOI:** 10.1186/s12967-025-07086-x

**Published:** 2025-10-08

**Authors:** Ming Yi, Xingrong Feng, Qiuyue Guan, Yin Liu, Yunqiang Liu, Zhiguang Su

**Affiliations:** 1 Department of High Altitude Medicine, High Altitude Medicine Key Laboratory of Sichuan Province,Center for High Altitude Medicine, State Key Laboratory of Biotherapy, West China Hospital, Sichuan University, Chengdu, 610041 China; 2 Department of Outpatient, Sichuan Provincial People’s Hospital, University of Electronic Science and Technology of China, Chengdu, China; 3 Department of Laboratory Medicine, West China Hospital, Sichuan University, Chengdu, China; 4 Department of Medical Genetics, State Key Laboratory of Biotherapy, West China Hospital, Sichuan University, Chengdu, 610041 China

**Keywords:** Mendelian randomization, Single-cell RNA-seq, Peptidylglycine-alpha-amidating monooxygenase, Type 2 diabetes; islets, Pancreatic β-cells, Molecular docking simulations, Oleic acid, Drug discovery

## Abstract

**Background:**

The progression of type 2 diabetes (T2D) is driven by pancreatic β-cell dysfunction and loss, yet current therapies fail to address this core pathophysiology.

**Methods:**

We implemented an integrative pipeline combining genetic and functional data to prioritize T2D targets. We leveraged genome-wide association study (GWAS) and protein quantitative trait loci (pQTL) summary data to infer causal associations between circulating proteins and disease risk. Phenome-wide association studies were conducted to evaluate pleiotropy and potential off-target effects. Single-cell RNA-seq was used to delineate cell-type-specific expression and identify biological pathways associated with candidate genes. Lead candidates were then validated by molecular docking and functional assays.

**Findings:**

Mendelian randomization and colocalization analyses prioritized 14 circulating proteins with causal links to T2D, nine of which shared strong causal variants (PPH4 > 0.8). Phenome-wide association studies (PheWAS) excluded off-target effects for 2 candidates (HP and SVEP1). Among the remaining 7 candidates (ENG, GOLM1, GSTA1, HIBCH, PAM, PLXND1, and PTN), PAM (peptidylglycine α-amidating monooxygenase) was found to be expressed in over 80% of β-cells, as revealed by single-cell RNA-sequencing. Moreover, genes co-expressed with PAM were functionally clustered in pathways related to insulin secretion and protein processing. Notably, PAM expression was significantly downregulated in islets of diabetic mice. Molecular docking simulations identified a high-affinity interaction between PAM and oleic acid (OA), a metabolite linked to β-cell function. Strikingly, pharmacological PAM inhibition in INS1 β-cells induced deficits in cell proliferation and survival that were unresponsive to OA supplementation, underscoring PAM’s indispensable role in β-cell integrity.

**Interpretation:**

This study positions PAM as a clinically relevant therapeutic target for T2D, offering new opportunities for β-cell preservation therapies and diagnostic biomarker development.

**Supplementary Information:**

The online version contains supplementary material available at 10.1186/s12967-025-07086-x.

## Highlights

Mendelian randomization and colocalization analyses identified 14 circulating proteins causally linked to T2D, 9 sharing causal variants. Phenome-wide association studies narrowed these to 7 targets by excluding pleiotropic effects.

Among the 7 candidates, peptidylglycine α-amidating monooxygenase (PAM) prioritized as the top therapeutic target due to its expression in over 80% of pancreatic β-cells.

PAM expression was significantly reduced in diabetic mouse islets.

Molecular docking revealed PAM bands tightly to β-cell-function-associated with oleic acid (OA). Inhibiting PAM in INS1 β-cells impaired proliferation and survival, even with OA supplementation.

**Supplementary Information:**

The online version contains supplementary material available at 10.1186/s12967-025-07086-x.

## Introduction

T2D represents one of the most significant global health challenges, with projections indicating it will affect over 700 million individuals by 2045 [1, 2]. Current therapeutic strategies predominantly manage hyperglycemia while failing to address the progressive deterioration of pancreatic β-cell function and mass—the fundamental pathophysiological process driving disease progression [3]. This limitation underscores the urgent need for novel interventions specifically designed to preserve or restore β-cell integrity, potentially transforming T2D treatment paradigms.

The human plasma proteome offers a rich reservoir of potential therapeutic targets, comprising proteins that may function as disease mediators or druggable targets [4, 5]. Observational studies frequently struggle to differentiate proteins causally involved in disease pathogenesis from those merely correlated with diabetic states. Mendelian randomization (MR) provides a powerful analytical framework to mitigate confounding factors by employing genetic variants as instrumental variables—effectively simulating randomized controlled trials at the population level [6]. By instrument variables, this strategy not only guides disease interventions but also minimizes the risk of off-target effects and adverse outcomes. Nevertheless, genetic pleiotropy can compromise MR inferences, particularly when variants within the same genomic region influence multiple traits through linkage disequilibrium (LD) [7]. Addressing this requires rigorous application of statistical genetics methods to isolate genuine causal effects.

MR studies of drug targets inform and prioritize drug development by establishing causal relationships between target perturbation and disease risk, thereby providing a robust framework for precision medicine approaches in T2D. However, a key challenge remains in delineating the specific cellular contexts in which these target proteins exert their pathogenic or therapeutic effects, which is crucial for effective drug development. Integrating MR findings with single-cell transcriptomic data enables the dissection of how plasma protein–encoded mRNAs modulate T2D pathogenesis at the resolution of individual cellular populations [8]. This approach enables prioritization of targets expressed in disease-relevant cell population, providing critical insights for drug development strategies.

Despite the power of integrated genetic and transcriptomic approaches, a critical knowledge gap remains: whether modulating candidate proteins directly impacts β-cell function. To address this, we employed molecular docking simulations to evaluate protein druggability and screen potential ligands for functional experiments. Furthermore, we confirmed the pathophysiological relevance of our targets in high-fat diet-induced T2D mice and determine their effects on β-cell function in INS1 cells treated with the identified molecules. These experimental validations bridge the gap between statistical association and therapeutic potential.

Our integrated approach translates findings from human population genetics and transcriptomics into tangible biological mechanisms. By combining genetic epidemiology, cellular transcriptomics, and functional validation, this research identifies and prioritizes potential drug targets that address the fundamental pathophysiology of T2D rather than merely managing symptoms, potentially revolutionizing treatment approaches for this pervasive metabolic disorder.

## Materials and methods

### Study design

Suppl. Figure 1 illustrates our integrated analytical framework for identifying and validating plasma proteins as potential T2D therapeutic targets. First, we employed MR and colocalization analyses to establish putative causal relationships between plasma proteins and T2D risk. Promising candidates underwent PheWAS to assess potential pleiotropic effects and on-target side effects, ensuring favorable safety profiles. Additionally, single-cell RNA sequencing of human pancreatic islets characterized cellular expression profiles of prioritized proteins, establishing biological plausibility through tissue-specific expression patterns in relevant cell types. Finally, the most promising targets were further validated through molecular docking simulations and functional assays in diabetic mice and pancreatic β-cell models, providing mechanistic insights into their effects on insulin secretion, glucose metabolism, and cell survival.

### Mendelian randomization (MR) analysis

We conducted a two-sample MR analysis using GWAS summary data to evaluate the causal effects of circulating proteins levels and T2D risk. Single-nucleotide polymorphisms (SNPs) associated with the levels of plasma proteins at the genome-wide significance level were derived from two large-scale GWASs in the deCODE [5] and ARIC health study [9]. Genetic variants associated with T2D were obtained from the DIAbetes genetics replication and meta-analysis (DIAGRAM) consortium [10]. All GWAS data were ethically approved, conformed to the Declaration of Helsinki, and obtained.

We implemented both single-variant and multiple-variant approaches for MR analysis [11]. For the single-variant approach, we selected the lead SNP (identified by maximum Z score) as the instrumental variable for each protein. To ensure robustness, exposure-associated variants were retained only if they reached genome-wide significance (*P* < 5 × 10⁻⁸) in both the discovery (deCODE) and replication (ARIC) cohorts and displayed concordant directional effects (β) between them (Suppl. Table 1). We excluded variants potentially associated with T2D-influencing confounders and outlier SNPs identified through MR-PRESSO analysis [12]. We further excluded SNPs with minor allele frequencies below 0.01 and palindromic SNPs to minimize potential bias. Instrumental variable strength was verified with F-statistics consistently exceeding the conventional threshold of 10, confirming adequate statistical power [13] (Suppl. Table 1). The causal effects were estimated via the Wald ratio method and inverse variance weighted (IVW) approach. Cochran’s Q statistic was used to assess heterogeneity among different instrumental variables, with *P* < 0.05 indicating significant heterogeneity. MR-Egger regression was employed to assess directional pleiotropy, with a significant non-zero intercept (*P* < 0.05) suggesting potential bias due to pleiotropic effects [14]. Leave-one-out analysis was performed by sequentially removing each instrumental variable and repeating the MR analysis with the remaining instruments to assess estimate stability.

### Colocalization analyses

To systematically evaluate the probability of shared causal variants between traits, we employed Bayesian colocalization analyses using the ‘coloc’ package (version 4.4.1) [15]. This statistical framework calculated posterior probability of hypothesis (PPH) of genetic association between trait pairs across 5 distinct scenarios: PPH0: absence of genetic association with either trait; PPH1: genetic association exclusively with trait 1; PPH2: genetic association exclusively with trait 2; PPH3: genetic associations with both traits, attributed to distinct causal variants; PPH4: genetic associations with both traits, attributed to a shared causal variant. The probability values of PPH4 between 0.5 and 0.8 (0.5 < PPH4 < 0.8) suggest a moderate likelihood of colocalization, whereas PPH4 values of 0.8 or higher (PPH4 ≥ 0.8) indicate a high likelihood of colocalization.

### Phenome-wide association studies (PheWAS)

We conducted variant-level PheWAS for each instrumental variable across 1,419 phenotypes using a Bonferroni-corrected significance threshold (*P* < 0.05/1419 = 3.52 × 10^− 5^) [16]. This analysis was specifically performed for putative targets supported by both 2SMR and colocalization genetic evidence, implemented through the PheWeb database [16].

### Cell type annotation and differential gene identification from single-cell data

All single-cell datasets were integrated into a Seurat object to ensure data consistency. Ambient RNA contamination was assessed and controlled using decontX [17]. The DoubletFinder algorithm was employed to score cells for multiple detection [18]. Quality control measures included filtering out cells expressing fewer than 250 genes, genes expressed in fewer than three cells, and cells with mitochondrial gene expression exceeding 20% [8]. Data normalization was performed using the NormalizeData function from the Seurat package with scale-factor parameter set to 10,000. Each sample was treated as an independent batch, and batch effects were corrected using the Harmony algorithm [19]. Unless otherwise specified, all algorithmic procedures were conducted using default parameter settings. The effectiveness of batch effect removal was evaluated using t-distributed stochastic neighbor embedding (t-SNE) and uniform manifold approximation and projection (UMAP), implemented via the RunTSNE and RunUMAP functions within the Seurat package.

Cell clustering was performed using the Leiden algorithm with resolution parameter set to 0.4 to identify distinct cell subpopulations. Differentially expressed genes between cell subpopulations were identified using the FindAllMarkers function from the Seurat package with the Wilcoxon rank-sum test. Major cell types were annotated based on established marker genes [20], including: CAP3 and TPSAB1 for mast cells; GCG and TMSF4 for alpha cells; PPY and ARX for PP cells; INS and IAPP for beta cells; SST and PRG4 for pancreatic D cells; PRSS1 and CPA1 for pancreatic acinar cells; KRT7 and KRT19 for pancreatic ductal cells; COL1A1 and COL1A2 for fibroblasts; RGS5 and CAV1 for pericytes; PECAM1 and CLDN5 for endothelial cells; and APOE and CD74 for macrophages.

### Molecular docking

Molecular docking analyses were performed to assess binding energies and interaction patterns between candidate drugs and target proteins. Structural data for candidate drugs were obtained from the PubChem compound database (https://pubchem.ncbi.nlm.nih.gov/), downloaded in SDF format, and converted to PDB format using OpenBabel 2.4.1. Protein structure data were sourced from the Protein Data Bank (PDB) (http://www.rcsb.org/). The molecular docking process utilized CB-DOCK2 (https://cadd.labshare.cn/cb-dock2/php/index.php) [21] and P2RANK (https://prankweb.cz/) [22], with small molecule drugs ranked by binding energy. Results were visualized using PyMol 3.1.4 (https://www.pymol.org/). By identifying ligands with high affinity and favorable interactions, we prioritized potential drug targets for subsequent experimental validation.

### Enrichment analysis and cell differentiation assessment

We performed Gene Ontology (GO), Kyoto Encyclopedia of Genes and Genomes (KEGG), and Reactome pathway enrichment analyses using the R packages clusterProfiler [23] and ReactomePA [24]. Meta β-cell analysis was conducted using the SuperCell package [25]. The package Cytotrace is employed to evaluate the differentiation potential of cells [26].

### Animals, treatments, and pancreatic islet mRNA expression analysis

All animal experiments were approved by the Animal Care and Use Committee of Sichuan University. Male C57BL/6J mice purchased from Dossy experimental animal Corporation (Chengdu, China) were housed under a 12-hour light/dark cycle with free access to food and water. Mice were given either standard chow or a high-fat diet (HFD) containing 60% fat (TROPHIC Animal Feed High-Tech Co., Ltd, Shanghai, China) from 6 weeks of age. The T2D model was established by HFD feeding for 3 months. The glucose tolerance test (GTT) and insulin tolerance test (ITT) were performed as previously described [27].

Mouse pancreatic islets were isolated according to the procedure detailed in our previous study [28]. Total RNA was extracted from approximately 30 mg of pancreatic islet samples using Trizol (ThermoFisher Ambion) and reverse-transcribed into cDNA with a cDNA synthesis kit (Takara). Quantitative real-time PCR was performed using SYBR Green technology, following the methods described previously [27], with specific primers (Suppl. Table 2). Gene expression levels were normalized to 18 S rRNA expression and are presented as relative quantification (RQ) value calculated using the comparative threshold cycle (ΔΔCt) method (RQ = 2^-ΔΔCt^).

### Cell culture, CCK-8 assay, and Western blot analysis

The INS-1 cell line was cultured in RPMI 1640 medium (Viva Cell) supplemented with 10% fetal bovine serum (Zeta Life), 1% penicillin/streptomycin (Ecotop), and 50 µM/mL β-mercaptoethanol (Macklin). Cultures were maintained in a humidified incubator with 5% CO₂ at 37 °C. For glucose treatment experiments, INS-1 cells were incubated in media containing either 5.5 mM or 16.7 mM glucose. Proliferation of INS-1 cells was measured using the CCK-8 assay (Oriscience). Cells were seeded into 96-well plates and incubated for 24 h to reach approximately 30% confluency before treatment with 4-phenyl-3-butenoic acid (4P3BA) and/or OA. Each condition was assayed in quintuplicate. At 24, 48, 72, and 96 h post-treatment, 10 µL of CCK-8 reagent was added to each well, followed by 2-hour incubation at 37 °C. Absorbance at 450 nm was measured using an Infinite 200 pro Eplex (Tecan).

Cellular protein extraction, protein concentration measurement, and Western blotting processes with primary antibodies (Suppl. Table 3) were carried out as described previously [29].

### Statistical analysis and visualization

Statistical analyses and visualization were performed using R software (version 4.2.3). For comparisons between two groups, Student’s t-test or, where appropriate, the Wilcoxon rank-sum test was employed. Comparisons among more than two groups were evaluated using one-way ANOVA test. The false discovery rate (FDR) was controlled to adjust for multiple comparison.

## Results

### Two-sample MR reveals broad landscape of potential therapeutic targets

**Fig. 1:**
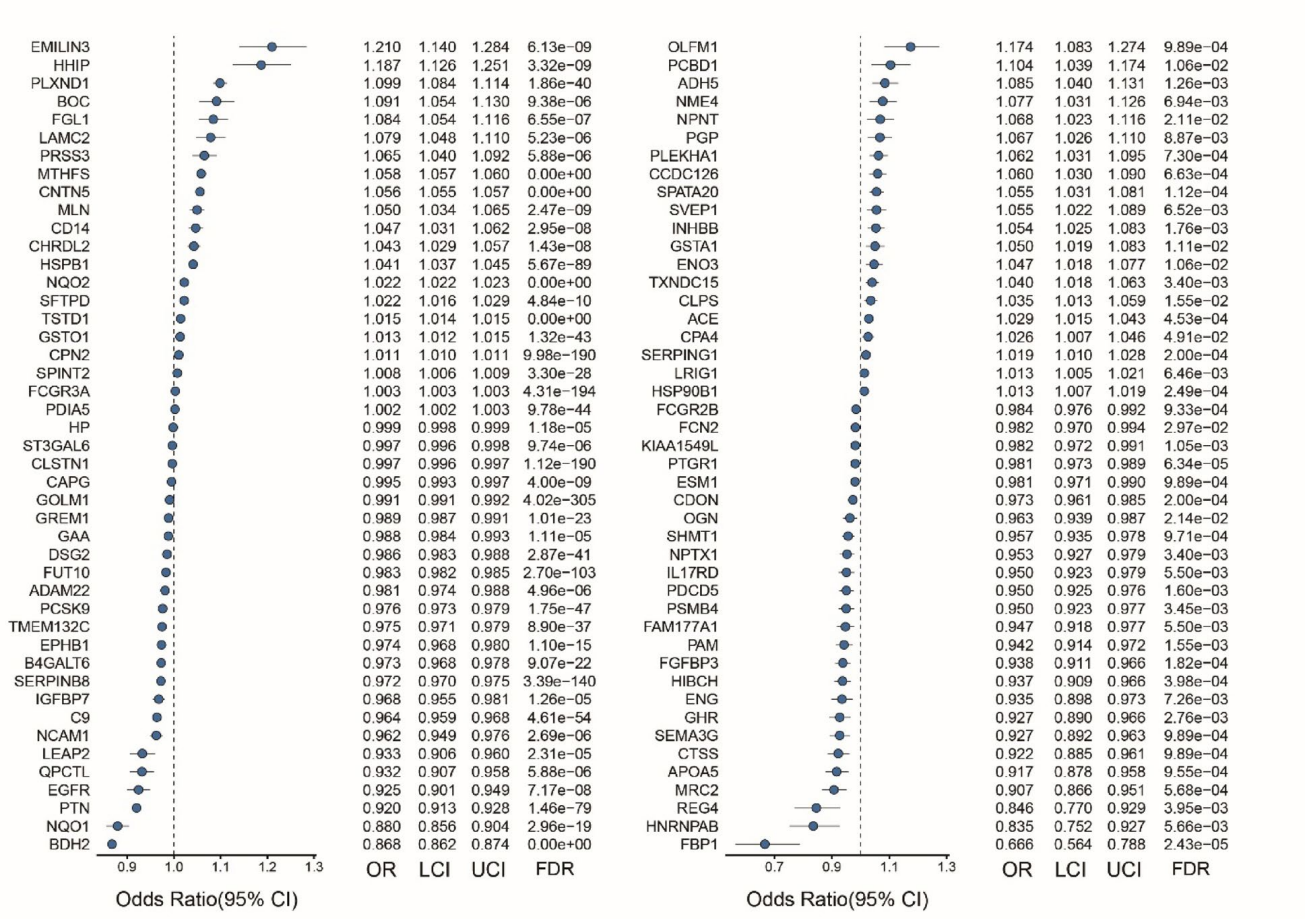
Genetic proxies identified significant associations between 90 plasma protein levels and T2D risk. The forest plot shows odds ratio (OR) with 95% confidence interval (CI) for each protein-T2D association. Results are adjusted for multiple testing using FDR correction. LCI, lower 95% CIs of OR; UCI, upper 95% CI of OR; FDR, false discovery rate

In the deCODE discovery cohort, a 2SMR analysis of 586 plasma proteins identified 90 proteins whose circulating levels were causally associated with T2D risk (FDR < 0.05, Fig. 1). These proteins were prioritized as potential therapeutic targets. The robustness of these associations was further confirmed through independent replication in the ARIC cohort (Fig. S2). The MR-Steiger tests validated the hypothesized causal direction, indicating that protein levels influence T2D risk. This was evidenced by the fact that the genetic instruments explained a significantly greater proportion of variance in protein abundance than in T2D susceptibility (Suppl. Table 4). Sensitivity analyses using multi-SNP instruments in the MR approach provided further support for these findings. There was no evidence of significant heterogeneity (Cochran’s Q test, *P* > 0.05) or directional horizontal pleiotropy (MR-Egger intercept *P* > 0.05, Fig. S3). These results collectively strengthened the causal evidence for the identified proteins as potential drug targets.

### Colocalization analysis prioritizes high-confidence therapeutic candidates

**Fig. 2:**
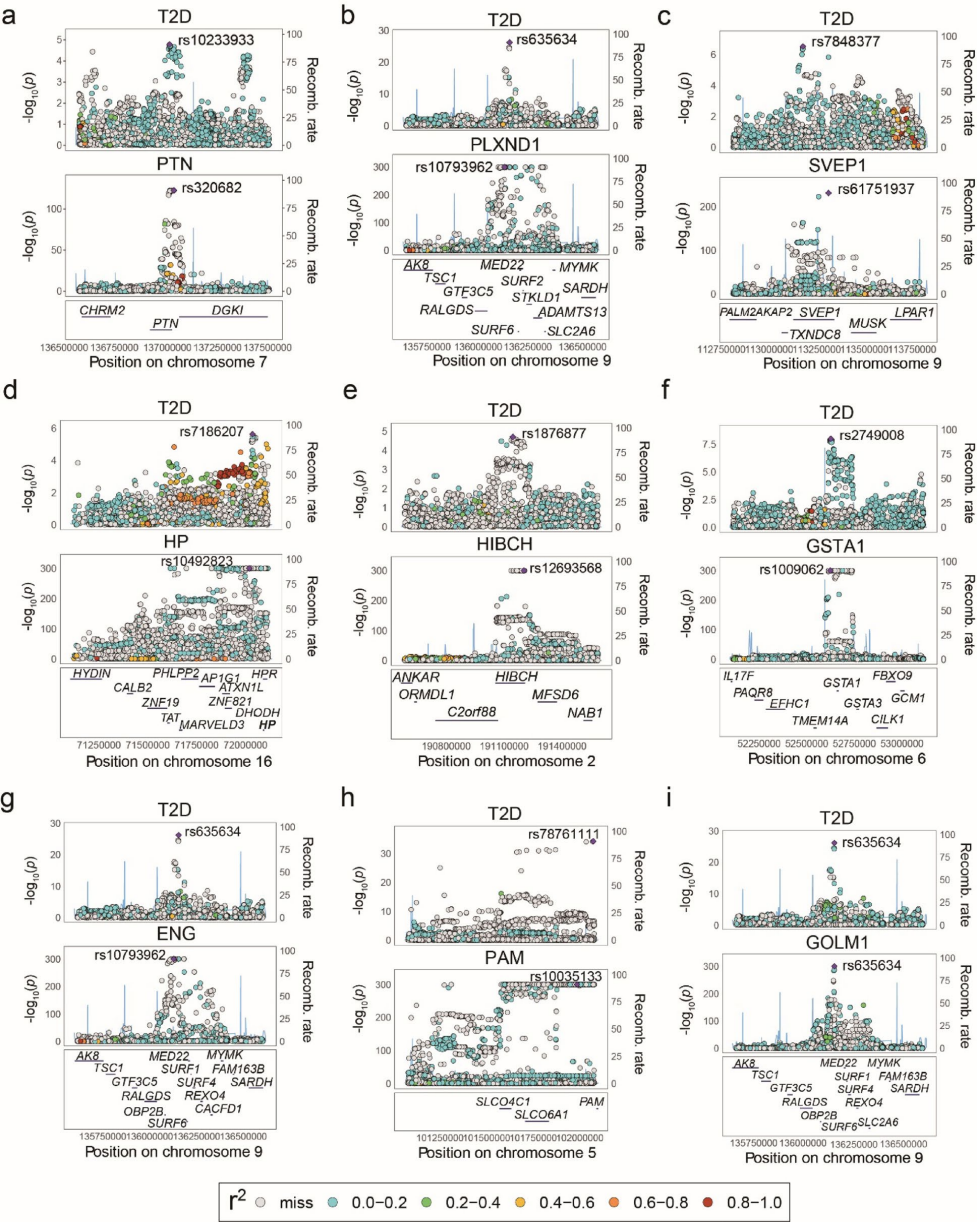
Regional colocalization plots illustrating the shared genetic architecture between plasma protein abundance and T2D risk in individuals of European ancestry. Each point represents a single nucleotide polymorphism (SNP), with genomic position shown on the x-axis and association strength (-log_10_ P value) for T2D risk (top) and protein abundance (bottom) on the y-axis. Colors indicate linkage disequilibrium (LD, r^2^) relative to the lead variant, with darker shades representing higher LD. Open circles denote SNPs with missing LD information

To address potential genetic pleiotropy signals arising from linkage disequilibrium, we performed Bayesian colocalization analysis across our 90 prioritized drug targets. Among these, nine exhibited strong colocalization genetic evidence, with a PPH4 > 0.8 (Fig. 2, Fig, S4). Additionally, five proteins demonstrated moderate colocalization genetic evidence, with PPH4 values between 0.5 and 0.8 (Fig. S4-S5). Collectively, these findings define a shared genetic architecture linking specific circulating protein levels to T2D susceptibility and refine our list to a focused set of high confidence therapeutic candidates for downstream investigation.

### Variant-level PheWAS evaluates target safety profiles

**Fig. 3:**
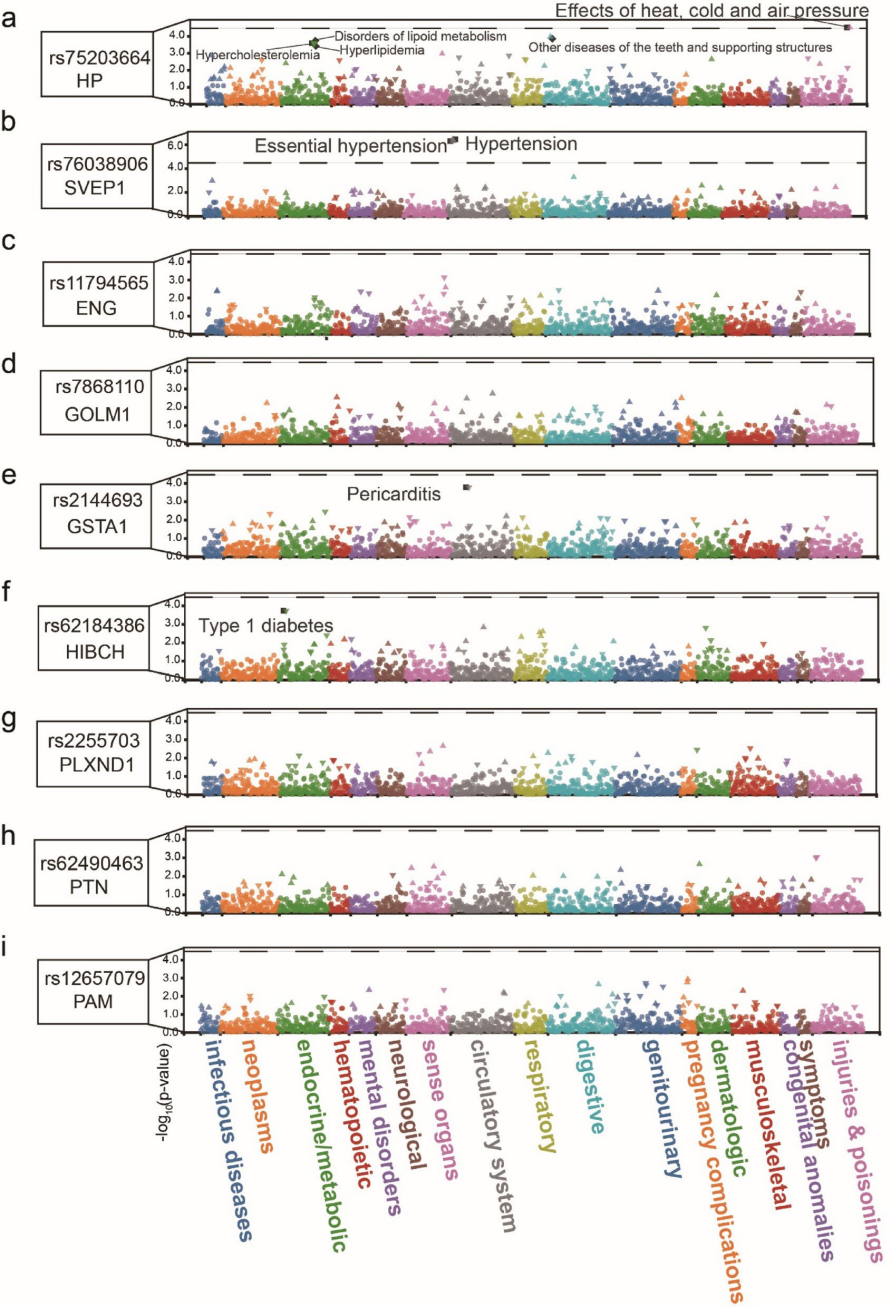
Summary of variant-level phenome-wide association studies (PheWAS). PheWAS results for nine genetic variants included as instrumental variables in Mendelian randomization (MR) analyses. Each point represents an association between a variant and a phenotype, with significance tested against a Bonferroni-corrected threshold (P < 0.05/1419 = 3.52×10^−5^)

To assess potential off-target effects and anticipate safety considerations of candidate therapeutic targets, we conducted comprehensive PheWAS for each instrumental variable across 1,419 phenotypes. We used a Bonferroni-corrected significance threshold (*P* < 0.05/1419 = 3.52 × 10^− 5^) to ensure rigorous control. Remarkably, two of our 9 candidate proteins exhibited significant phenotypic associations beyond T2D. The instrumental variable (rs75203664) for HP protein was significantly associated with effect of heat, cold, and air pressure (*P* = 3.1 × 10^− 5^, β = 3.10, 95% CI: 2.36–3.84) (Fig. 3a). The SVEP1 instrumental variable (rs76038906) showed significant associations with hypertension (*P* = 3.7 × 10^− 7^, β = 0.090, 95% CI: 0.072–0.108) and essential hypertension (*P* = 5.2 × 10^− 7^, β = 0.089, 95% CI: 0.071–0.107) (Fig. 3b). In contrast, the instrumental variables for the remaining 7 prioritized drug targets showed no significant associations with any comprehensive phenotypes (*P* > 3.52 × 10^− 5^) (Fig. 3c-i). This suggests that therapeutic interventions targeting these protein pathways may offer favorable safety profiles with minimal off-target effects.

### Single-cell transcriptomics elucidates cellular context of therapeutic targets

**Fig. 4:**
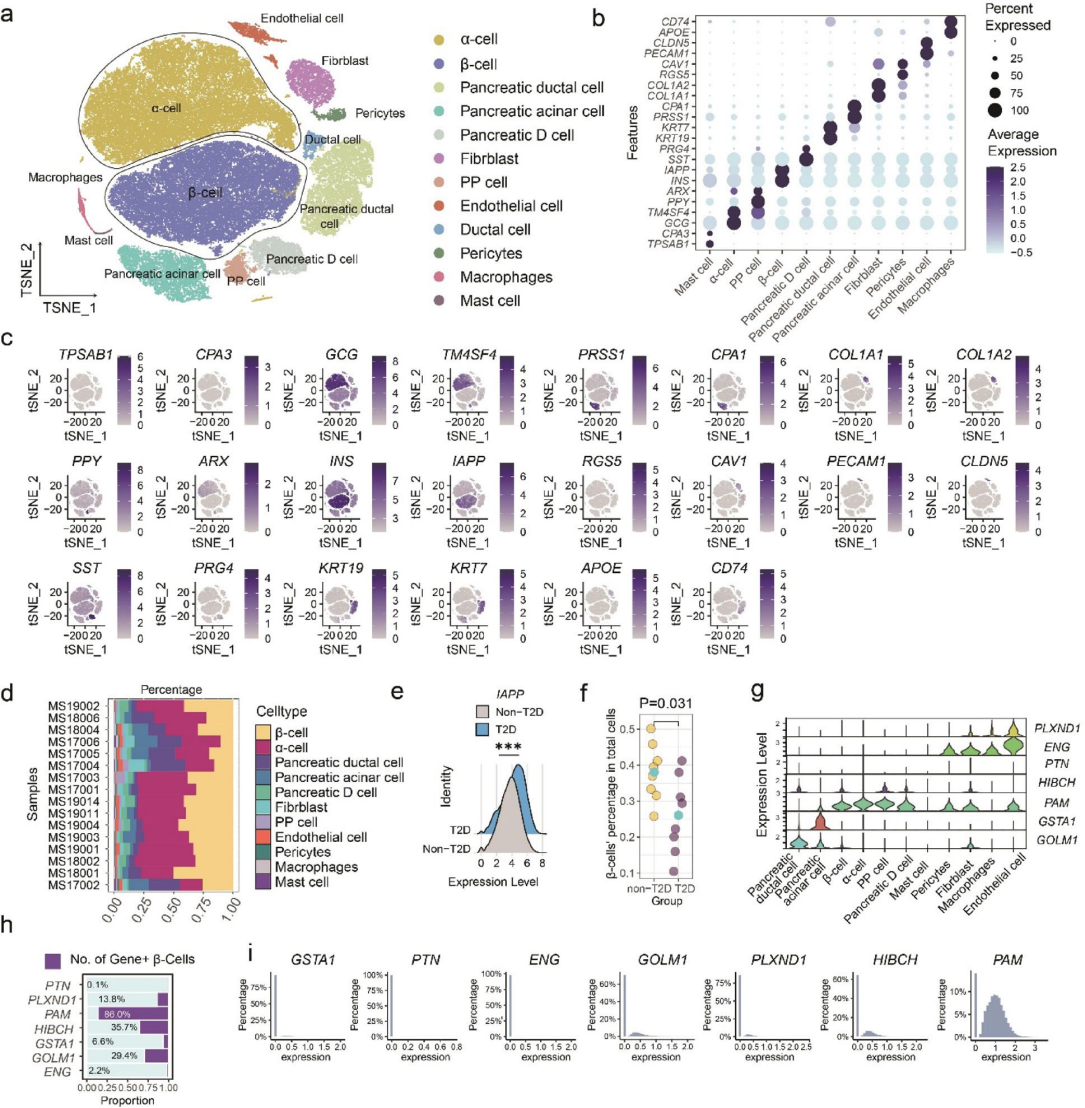
Single-cell resolution of plasma protein-encoded mRNAs.** a** t-distributed stochastic neighbor embedding (t-SNE) visualization of annotated cell types, including α-cells, β-cells, ductal cells, acinar cells, D cells, fibroblasts, PP cells, endothelial cells, pericytes, macrophages, and mast cells, showing distinct clustering of major populations. **b** Dot plots displaying expression levels of canonical marker genes across cell types, with color indicating mean normalized expression. **c** t-SNE plots showing the expression pattern of marker genes in integrated pancreatic single-cell populations. Each point represents a single cell, with color according to transcript abundance (normalized expression). **d** Stacked bar plots depicting the proportional composition of pancreatic cell types across clinical samples. **e** Expression distribution of IAPP in β-cells from T2D versus non-T2D donors. **f** Proportions of β-cells among all pancreatic cells per sample in non-T2D and T2D individuals, each dot represents one sample. **g** Violin plots showing expression of drug target genes across annotated pancreatic cell types. **h** Proportion of pancreatic β-cells expressing drug target gene among the total β-cell population. **i** Distribution of plasma protein-encoded mRNAs within β-cells

To delineate the cellular context of candidate therapeutic targets and assess their relevance to the pathophysiology of T2D, we integrated single-cell RNA sequencing data from 16 clinically derived pancreatic samples. Following rigorous quality control and batch effect correction (Fig. S6), a total of 76,096 cells were retained for subsequent analyses [30]. This approach enabled high-resolution mapping of target gene expression across 11 distinct pancreatic cell populations, including α-cells (27,926 cells), β-cells (24,759), ductal cells (8,150), acinar cells (5,253), δ-cells (3,105), fibroblasts (2,670 cells), endothelial cells (1,507 cells), PP cells (1,265 cells), pericytes (619 cells), macrophages (566 cells), and mast cells (276 cells) (Fig. 4a-c).

Comparative analyses further revealed significant heterogeneity in the cellular composition among clinical specimens (Fig. 4d). Islets isolated from individuals with T2D demonstrated increased expression of islet amyloid polypeptide (IAPP), consistent with its recognized role in amyloid deposition and β-cell dysfunction (Fig. 4e). Moreover, a statistically significant reduction in the proportion of β-cells was observed within pancreatic islets from T2D donors compared to non-diabetic controls (*P* = 0.031; Fig. 4f). These findings highlight β-cell loss as a hallmark of T2D pathogenesis and underscores the therapeutic imperative to preserve β-cell mass and function.

To underscore the potential utility for selective intervention, we analyzed transcripts encoding the 7 prioritized therapeutic target proteins. These proteins exhibited pronounced cell-type-specific expression profiles. Specifically, *PLXND1* and *ENG* were highly enriched within endothelial cells, while *GOLM1* and *GSTA1* exhibited specific enrichment in acinar and ductal cells. *PTN* expression was consistently detected at low basal levels across many cell types. Only *HIBCH* and *PAM* displayed broader expression across multiple endocrine populations, including β-cells, α-cells, PP cells, and δ-cells (Fig. 4g).

Further transcript prevalence analysis within the β-cell compartment revealed that *PAM* was detected in over 80% of β-cells, while *HIBCH* was only expressed in 36.7% of β-cells (Fig. 4h-i). This suggests that PAM may play a critical role in β-cell maintenance. Collectively, our data demonstrate a significant loss of β-cells in T2D, alongside the pervasive expression of *PAM* across the majority of β-cells, implicating a potential role for *PAM* in maintaining β-cell function.

### PAM is associated with insulin secretion and β-cell differentiated state

**Fig. 5:**
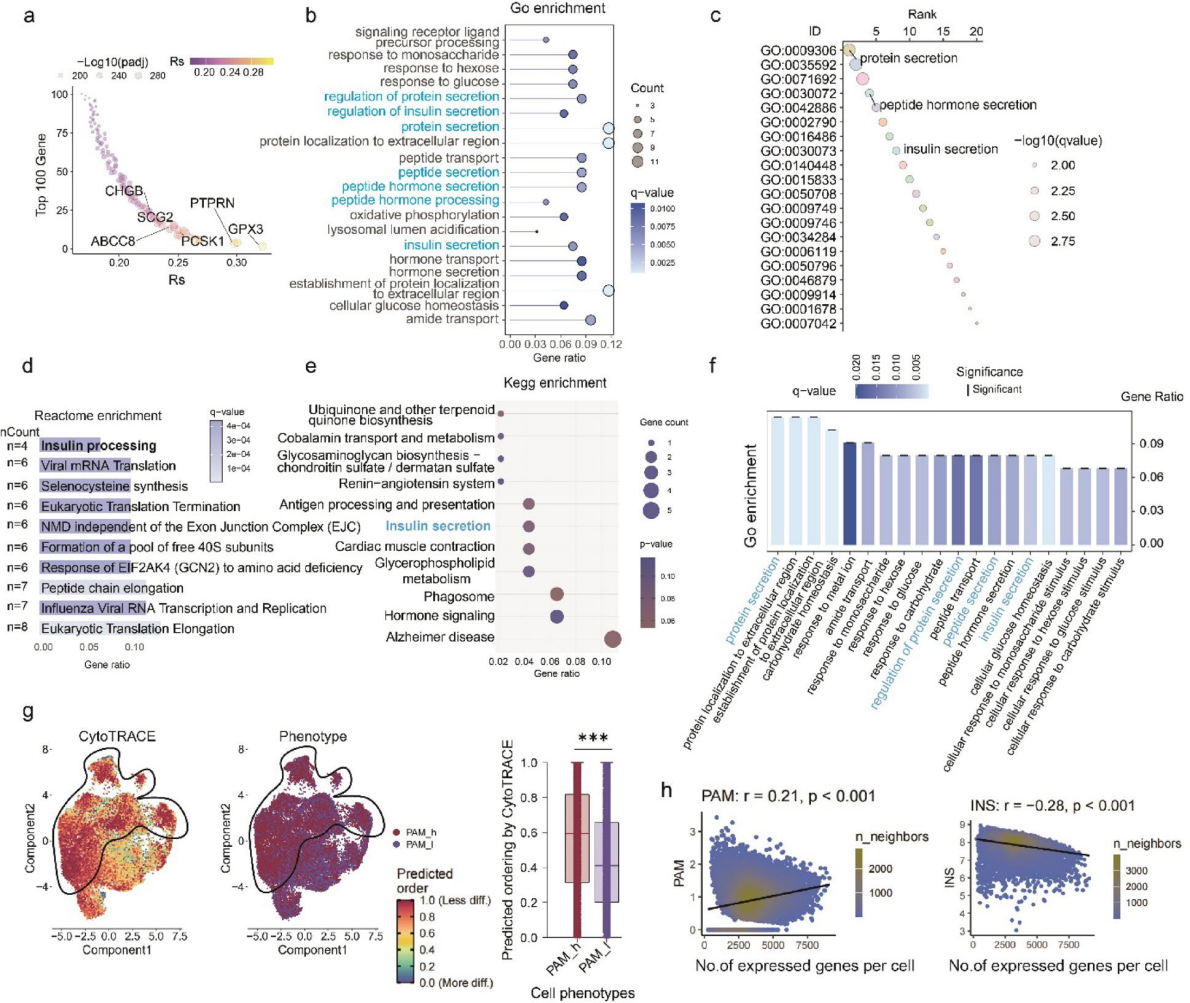
PAM expression is lined to insulin secretion pathways and a reduced β-cell differentiation state. **a** Spearman correlation analysis of PAM expression against all other genes in single β-cells, the top 100 most positively correlated genes are displayed. **b**–**e** Enrichment analysis of PAM-associated genes across multiple databases shows significant involvement in insulin secretion-related pathways, including **b** Gene Ontology (GO) biological processes, **c** GO ranked enrichment, **d** Reactome, and **e** KEGG pathways. Consistently enriched terms include insulin secretion, protein processing, and vesicle-mediated transport. **f** Re-analysis using meta β cells generated via the supercell algorithm reaffirmed enrichment of insulin secretion pathways among the top 100 PAM-correlated genes, supporting result robustness. **g** CytoTRACE analysis indicated that PAM-high β-cells are less differentiated than PAM-low cells. Shown are UMAP visualizations of CytoTRACE scores (left), PAM-expression groups (middle), and quantified differentiation scores (right). ***P < 0.001 by Wilcoxon rank-sum test. **h** Pearson correlation analysis revealed a positive association between gene count (an indicator of cell plasticity) and PAM expression (r = 0.21, *P* < 0.001) (left), and a negative correlation with INS expression (r = −0.28, *P* < 0.001) (right), supporting a role for PAM in maintaining a less differentiated transcriptional state in β-cells

Prior 2SMR and colocalization analyses, together with single‑cell transcriptomic profiling, identified PAM as a protective factor in T2D, playing a critical role in the maintenance of β‑cell mass. To elucidate how PAM preserves β‑cell mass and confers protection against T2D, we performed functional-enrichment analysis of PAM‑associated genes and assessed the impact of PAM on β‑cell differentiation state. By Spearman correlation analysis, we identified the top 100 genes most positively correlated with PAM expression and subjected them to pathway enrichment analysis (Fig. 5a). Notably, these PAM-correlated genes were significantly enriched in insulin secretion and related biological pathways (Fig. 5b-e). To enhance statistical robustness, we employed the supercell algorithm to aggregate single β-cells into lower-noise meta β-cells, and repeated the correlation analysis. Consistently, pathway enrichment of the top 100 PAM-correlated genes in meta β-cells also revealed significant enrichment in insulin secretion and related biological pathways (Fig. 5f), confirming the association between PAM and insulin secretory function.

Using the CytoTRACE algorithm to assess cellular differentiation status, we found that higher *PAM* expression was associated with a less differentiated β-cell state (Fig. 5g). Pearson correlation analysis between gene counts per cell (a proxy for gene expression diversity) and linearly scaled expression levels of *INS* and *PAM* revealed that PAM expression positively correlated with the number of detectable genes per cell (Fig. 5h), further supporting PAM’s potential role in maintaining β-cell plasticity. Conversely, *INS* expression negatively correlated with gene counts per cell, consistent with the established understanding that highly specialized, insulin-expressing β-cells exhibit lower gene expression diversity (Fig. 5h). These findings suggest PAM as a promising therapeutic target for promoting β-cell regeneration and functional recovery.

### Functional validation establishes PAM as a druggable target for T2D therapy

Building on our genomic and transcriptomic evidence, we identified PAM as a protectively interval target for T2D. However, a critical knowledge gap remains, whether modulating candidate proteins directly impacts β-cell function.

**Fig. 6:**
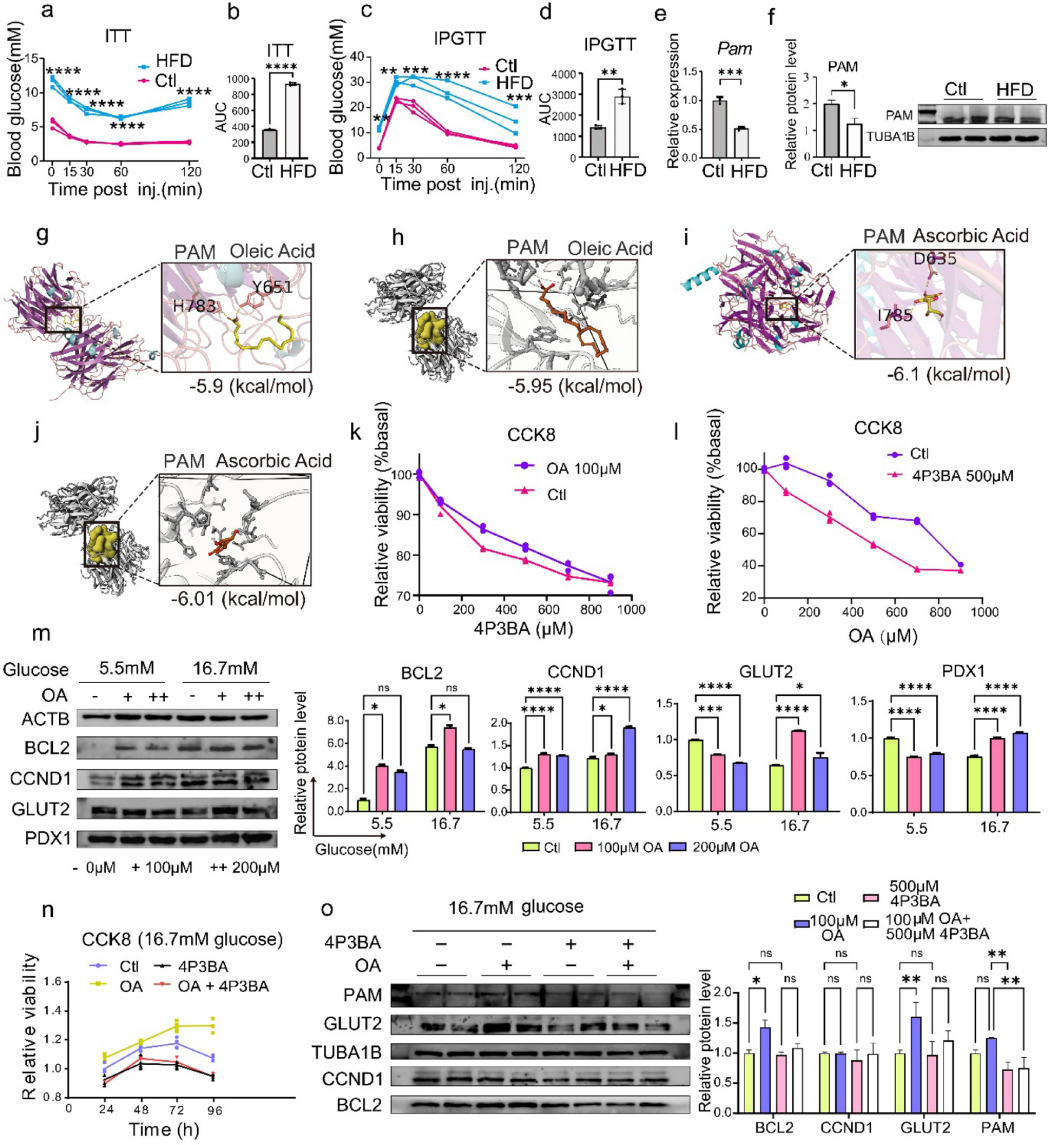
In vitro and in vivo functional validation of PAM as a potential therapeutic target. **a**–**d** The diabetic phenotype in a high-fat diet (HFD)-induced T2D mouse model was confirmed through both intraperitoneal glucose tolerance testing (IPGTT) and insulin tolerance testing (ITT). **e**-**f** The expression of PAM mRNA **e** and protein levels **f** in mouse pancreatic islet tissue were assessed using quantitative PCR and Western blotting, respectively. **g**–**j** Molecular docking analyses were conducted to evaluate the binding energies and interaction patterns between the PAM and oleic acid (OA) (g and h) or ascorbic acid (**i** and** j**). **k**–**o** Effects of OA and the PAM inhibitor 4-phenyl-3-butenoic acid (4P3BA) on INS-1 β-cell viability and function. Cell viability was assessed using the CCK-8 assay after treatment with various concentrations of 4P3BA or OA (**k, l**); After a 48-hour exposure to 100 or 200 μM OA under low (5.5 mM) or high (16.7 mM) glucose conditions, protein levels of cyclin D1 (CCND1), BCL2, PDX1 and GLUT2 were measured by Western blot (**m**); INS-1 cells cultured under hyperglycemic conditions (16.7 mM glucose) were treated with 100 μM OA, 500 μM 4P3BA, or their combination, cell proliferation was evaluated at 24, 48, 72, and 96 hours using the CCK-8 assay (**n**), and corresponding protein levels were quantified via Western blot (**o**)

To evaluate the therapeutic potential of PAM in vivo, we established an HFD-induced T2D mouse model. The successful induction of the diabetic phenotype was confirmed by both glucose tolerance testing and insulin tolerance testing (Fig. 6a–d). In the pancreatic islet tissue of these diabetic mice, both the mRNA expression and protein levels of PAM were significantly downregulated compared to those in normal controls (Fig. 6e–f). This downregulation of PAM suggests that its decline contributes to the development of T2D and further confirms that PAM functions as a protective factor against T2D.

Subsequently, we performed molecular docking simulations to identify potential small-molecule ligands for PAM. The results showed OA and ascorbic acid as potential therapeutic ligands (Fig. 6g-j), with docking simulations revealing comparable binding affinities (OA: CB-DOCK2: -5.90 kcal/mol, P2RANK: -5.95 kcal/mol; ascorbic acid: CB-DOCK2: -6.10 kcal/mol, P2RANK: -6.01 kcal/mol). However, considering ascorbic acid’s intrinsic role as an enzymatic cofactor which could potentially confound functional characterization, to migrate this, OA was selected for our representative PAM ligand for further functional arrays.

In vitro treatment of INS-1 cells revealed distinct effects of OA and the PAM inhibitor 4P3BA. Treatment with 100 µM OA significantly promoted cell proliferation, whereas 500 µM 4P3BA markedly reduced cell viability (Fig. 6k-l). Co-treatment demonstrated that 500 µM 4P3BA consistently suppressed cell viability across various OA concentrations. Conversely, 100 µM OA effectively counteracted the viability reduction induced by different concentrations of 4P3BA (Fig. 6k-l). Following 48-hour exposure to 100 or 200 µM of OA, Western blot analysis revealed that OA increased of cyclin D1 protein (CCND1) levels, indicative of stimulated proliferation, and elevated BCL-2 protein, suggesting enhanced anti-apoptotic capacity (Fig. 6m). Furthermore, under high-glucose conditions, OA upregulated expression of key β-cell functional proteins including PDX1 and GLUT2, contributing to improved overall β-cell functionality (Fig. 6m).

Cell viability analyses demonstrated that PAM inhibition significantly attenuated β-cell proliferation and survival under hyperglycemic conditions, an effect that remained unaltered even with OA supplementation (Fig. 6n). Western blot analysis revealed that OA treatment significantly upregulated anti-apoptotic BCL-2, glucose transporter GLUT2, and cell cycle regulator CCND1 in pancreatic β-cells (Fig. 6o). However, concurrent PAM inhibition with 4P3BA and OA supplementation resulted in marked downregulation of these critical protein (Fig. 6o). These findings demonstrate that PAM inhibition abolishes the beneficial effects of OA on β-cell mass and quantity, thereby establishing PAM activity as an essential prerequisite for OA-mediated β-cell protection. These mechanistic insights establish a causal relationship between PAM activity, OA binding, and β-cell function, definitively validating PAM as a druggable therapeutic target for T2D with potential clinical applications.

## Discussion

Molecular trait omics provides a powerful framework for therapeutic target discovery [7, 31, 32]. This study integrated genetic epidemiology, single-cell transcriptomics, and functional validation to systematically identify PAM as a high-confidence therapeutic target for preserving pancreatic β-cell function in T2D. T2D is characterized by progressive pancreatic β-cell dysfunction and loss [3], a pathophysiology corroborated by our findings of significantly reduced β-cell proportions in islets from T2D patients compared to non-diabetic controls.

Genetic evidence is a critical for validating causal drug target-diseases relationships and enhancing drug development success [33, 34]. Leveraging this, our MR analysis of 586 plasma proteins identified 90 candidates causally associated with T2D risk. Subsequent colocalization refined this to 14 high-confidence targets, 9 of which exhibited strong evidence for shared causal variants (PPH4 > 0.8). PheWAS further prioritized targets with favorable safety profiles by excluding candidate displaying pleiotropic effects (e.g., HP-linked thermoregulation phenotypes, SVEP1-hypertension associations), yielding 7 promising candidates. To delineate cellular context and disease relevance, we employed single-cell RNA-sequencing, which enables precise mapping of gene expression and pathway activity within specific cell populations, boosting more precise drug target discovery [35, 36]. This revealed predominant *PAM* expression in over 80% of human β-cells, with significant downregulation in diabetic islets. Functional validation confirmed PAM’s essential role: molecular docking identified high-affinity binding between PAM and OA, while pharmacological inhibition of PAM induced β-cell dysfunction that remained unresponsive to OA supplementation. These assays definitively demonstrated PAM’s requirement for OA-mediated β-cell protection.

The bifunctional enzyme PAM is evolutionarily conserved from *Chlamydomonas reinhardtii* to *Homo sapiens* and serves as the sole catalyst for bioactive peptide amidation [37]. This post-translational modification converts inactive peptidylglycine precursors into α-amidated peptides—a terminal step essential for activating numerous neural and endocrine peptides [38]. Critically, PAM deficiency disrupts insulin homeostasis, prior study demonstrates reduced insulin content and dysregulated secretory in human β-cell models [39]. Human genetic evidence further supports PAM’s role in diabetes pathogenesis: loss-of-function variants (Ser539Trp and Asp563Gly) impair amidating activity and associate independently with diabetes, defective insulin secretion, and elevated GH/IGF-1 [40]. Our findings expand this mechanistic understanding: the top 100 PAM-correlated genes significantly associated with insulin biosynthesis and secretory trafficking, reinforcing its centrality in insulin biosynthesis. Furthermore, we observed significant PAM downregulation in pancreatic islets of HFD-induced diabetic mice, directly linking reduced PAM expression to diabetic pathophysiology.

Beyond classical peptide amidation, PAM catalyzes oleamide biosynthesis using OA as substrate [41]. Notably, elevated OA not only provides substrate but also augments PAM activity, accelerating oleamide production [42]. Our computational docking reveals a direct physical basis for this regulation: OA binds PAM with high affinity, suggesting allosteric activation that enhances catalytic efficiency. This dual role establishes a feed-forward activation loop: OA both increases substrate supply and boosts PAM activity, creates a synergistic effect that dramatically amplifies oleamide biosynthesis. Oleamide itself exerts protective effects by inhibiting apoptosis [43] and promoting proliferation [44], this explains the improved INS1 β-cell functionality we observed upon OA treatment. Further functional validation confirmed PAM’s indispensability: pharmacological inhibition of PAM with 4P3BA severely compromised INS1 cell viability and function. Crucially, OA supplementation failed to rescue these deficits during PAM inhibition, proving that OA-mediated protection strictly requires PAM activity. Collectively, we identify OA as both a precursor and putative allosteric activator of PAM-driven oleamide synthesis. This paradigm revises models of lipid-mediated β-cell regulation and nominates the OA-PAM axis as a high-value therapeutic target, warranting future validation via mutagenesis and enzymatic studies.

While our integrated approach provides robust evidence for PAM’s therapeutic potential, there still exists several methodological constraints. First, the Eurocentric GWAS cohorts in our MR analysis may constrain its generalizability across diverse ethnic groups. Large-scale longitudinal studies are needed to validate our findings across diverse populations and establish the temporal dynamics of PAM expression in diabetes progression. Second, the validation of PAM’s effects on β-cells was primarily conducted in INS1 cell lines, which may not fully recapitulate the complexity of human pancreatic β-cell physiology. The development of more sophisticated in vitro models, including human islet organoids and patient-derived β cell cultures, would provide better translation of our findings to human pathophysiology. Third, although molecular docking identified OA as a potential PAM ligand, the precise contact residues and binding mode remained unresolved. Mutagenesis studies are required to validate the allosteric binding locus and to quantify the activation kinetics of OA.

As a whole, our study reveals that PAM is a paradigm-shifting target addressing β-cell failure—the core T2D pathophysiology. Its β-cell-restricted expression and minimal pleiotropy (per PheWAS) suggest improved safety over systemically acting agents. Our mechanistic work further positions OA as a potential therapeutic scaffold for PAM activation. The identification of PAM as a therapeutic target represents a paradigm shift from traditional glucose-lowering approaches to β-cell preservation strategies, addressing the fundamental pathophysiology of T2D progression.

## Supplementary Information

Below is the link to the electronic supplementary material.

Supplementary Material 1

Supplementary Material 2

Supplementary Material 3

Supplementary Material 4

Supplementary Material 5

Supplementary Material 6

Supplementary Material 7

## Data Availability

The datasets utilized in this study are available in online repositories. The specific repository/repositories and corresponding accession number(s) can be found in the article or Supplementary Material.

## Abbreviations

GEO: Gene expression omnibus
GWAS: Genome wide association studies
LD: Linkage disequilibrium
MR: Mendelian randomization
OA: Oleic acid
PAM: Peptidylglycine α-amidating monooxygenase
PheWAS: Phenome-wide association studies
PPH: Posterior probability of hypothesis
pQTLs: Protein quantitative trait loci
RNA-seq: RNA sequencing
SNP: Single nucleotide polymorphism
T2D: Type 2 diabetes
4P3BA: 4-phenyl-3-butenoic acid
