# Supplementary figures and images for: A multi-omics Mendelian randomization study reveals PAM as a potential therapeutic target for type 2 diabetes
Source: J Transl Med. 2025 Oct 8;23:1067. doi: 10.1186/s12967-025-07086-x (PMC12506332; doi:10.1186/s12967-025-07086-x)

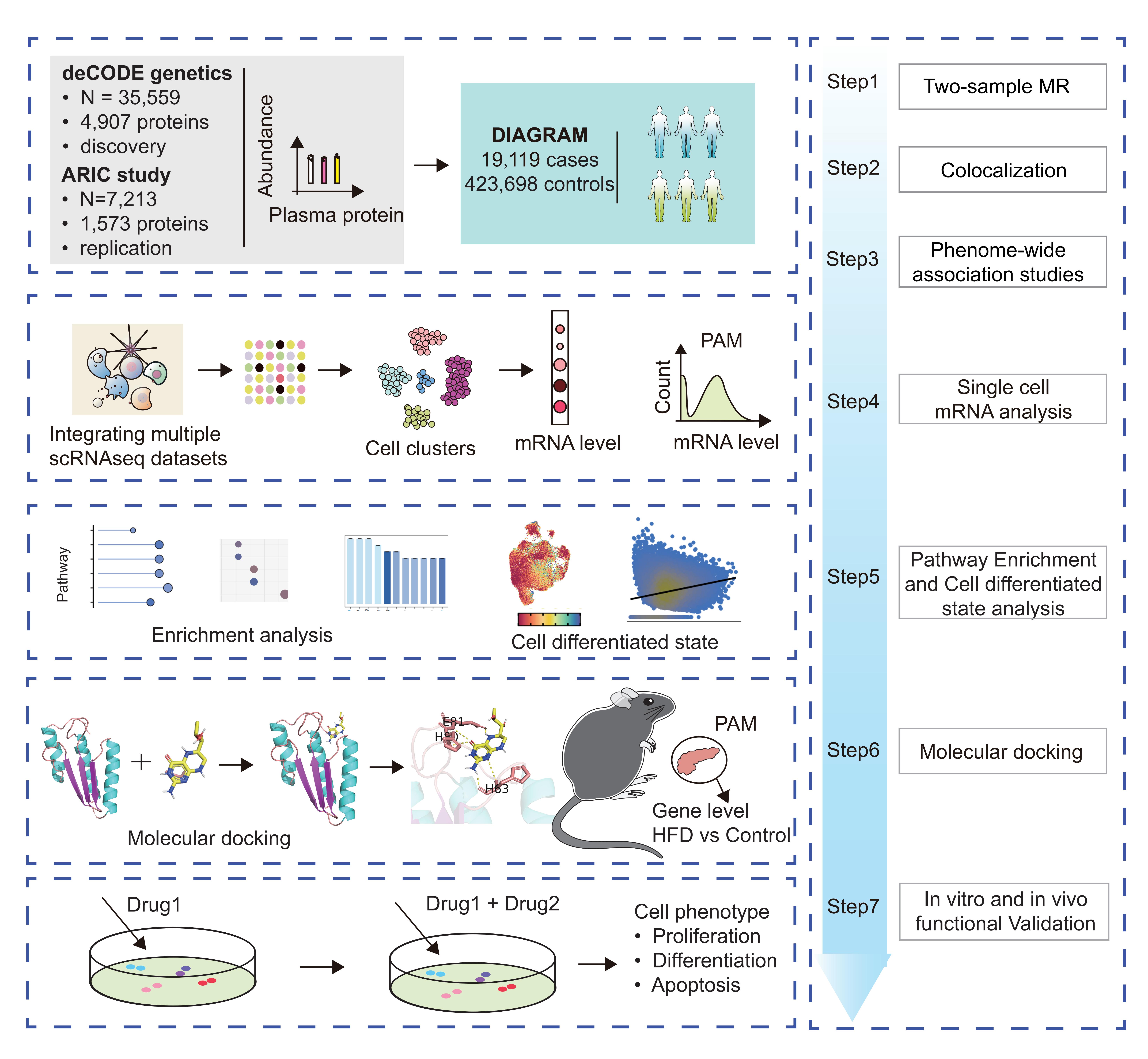

Supplement: Supplementary file 1 — Supplementary Material 1 [file 12967_2025_7086_MOESM1_ESM.jpg]

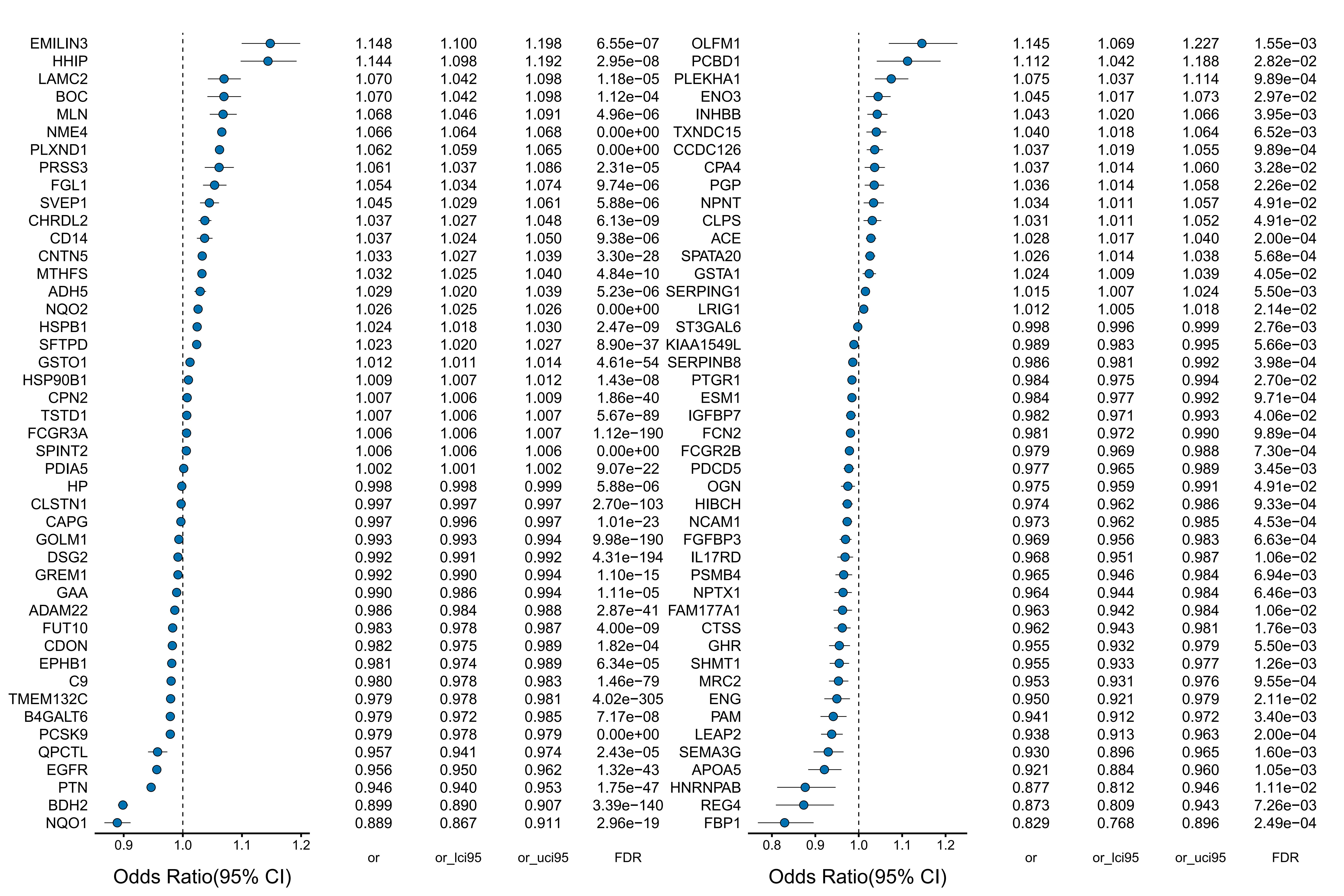

Supplement: Supplementary file 2 — Supplementary Material 2 [file 12967_2025_7086_MOESM2_ESM.jpg]

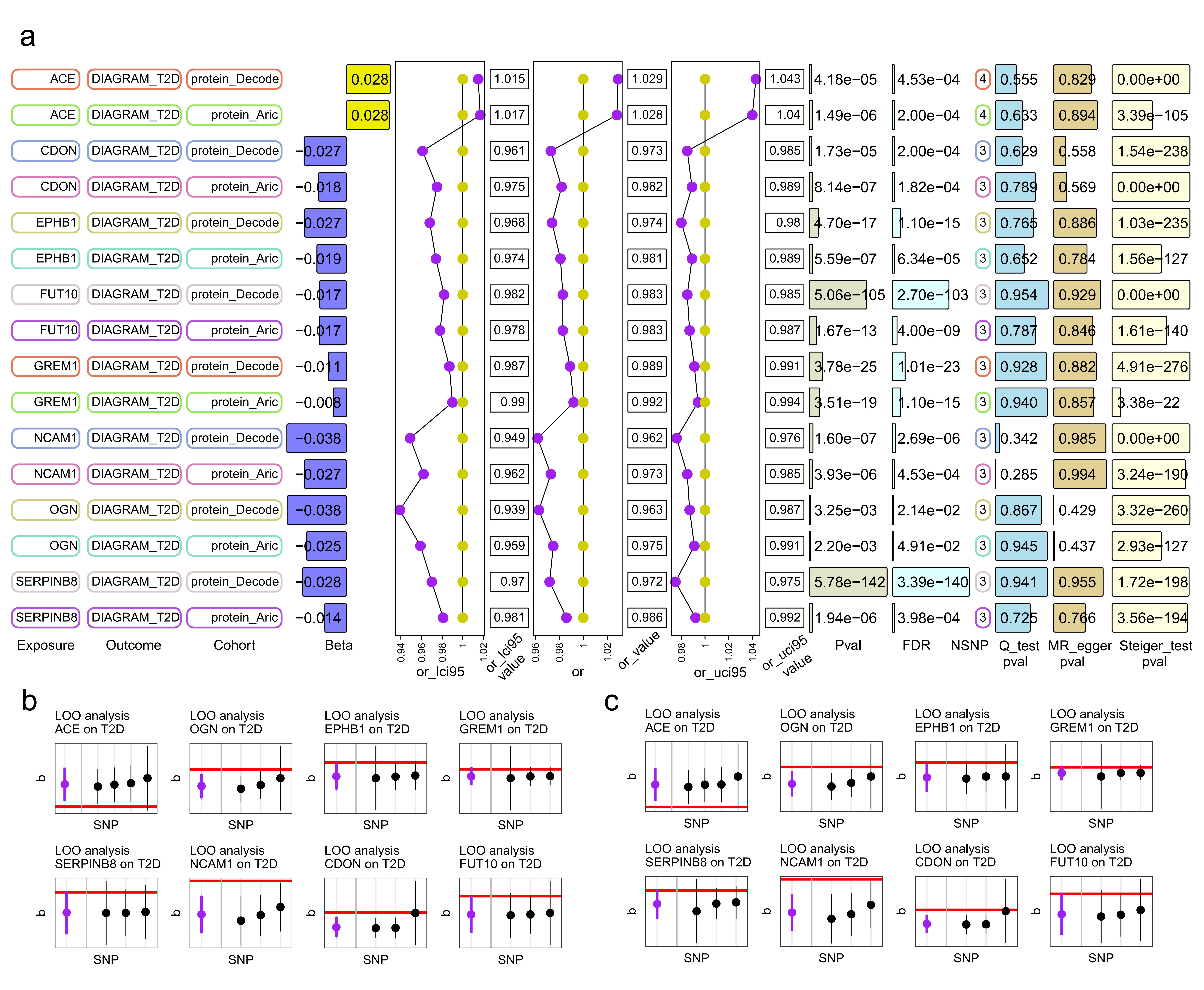

Supplement: Supplementary file 3 — Supplementary Material 3 [file 12967_2025_7086_MOESM3_ESM.jpg]

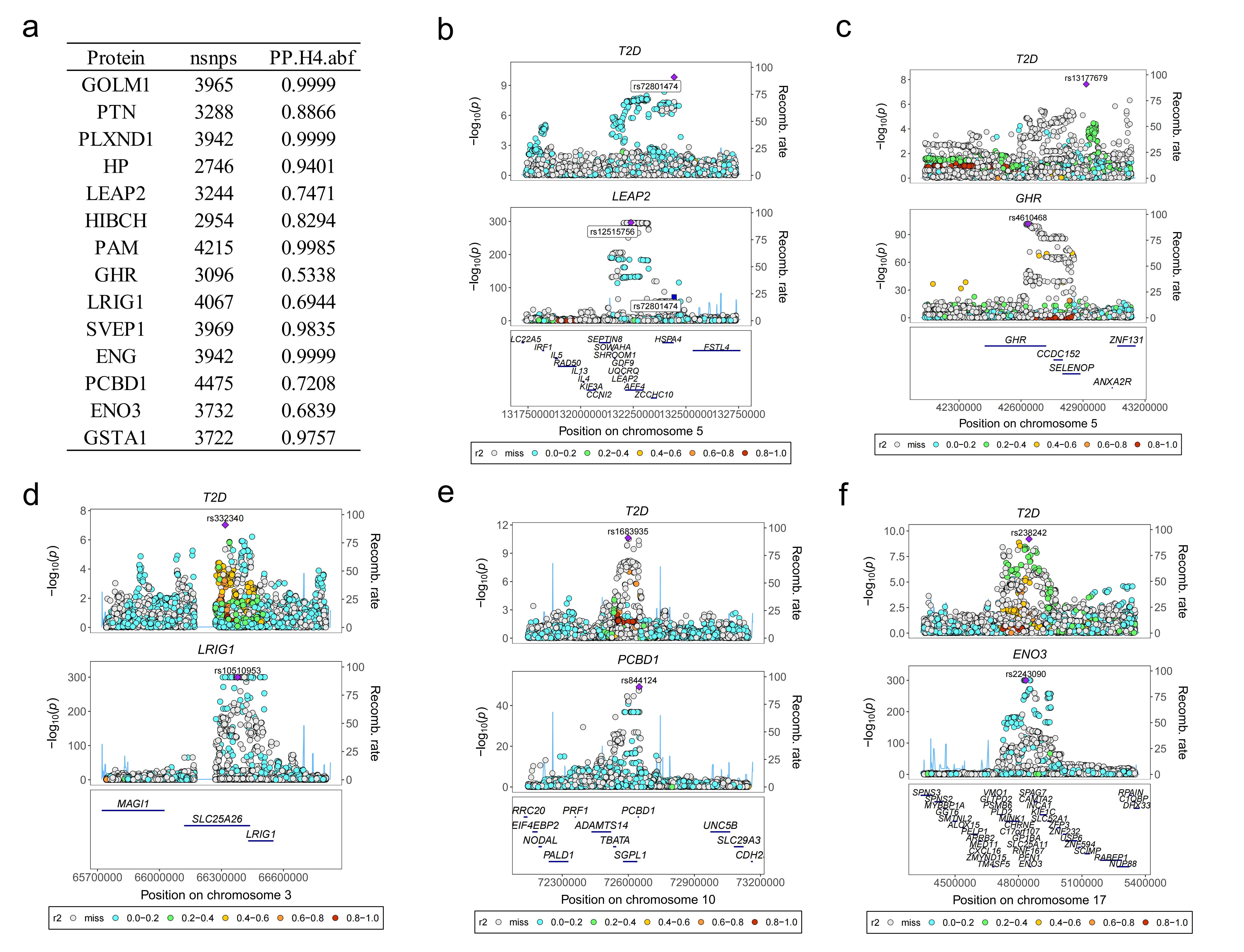

Supplement: Supplementary file 4 — Supplementary Material 4 [file 12967_2025_7086_MOESM4_ESM.jpg]

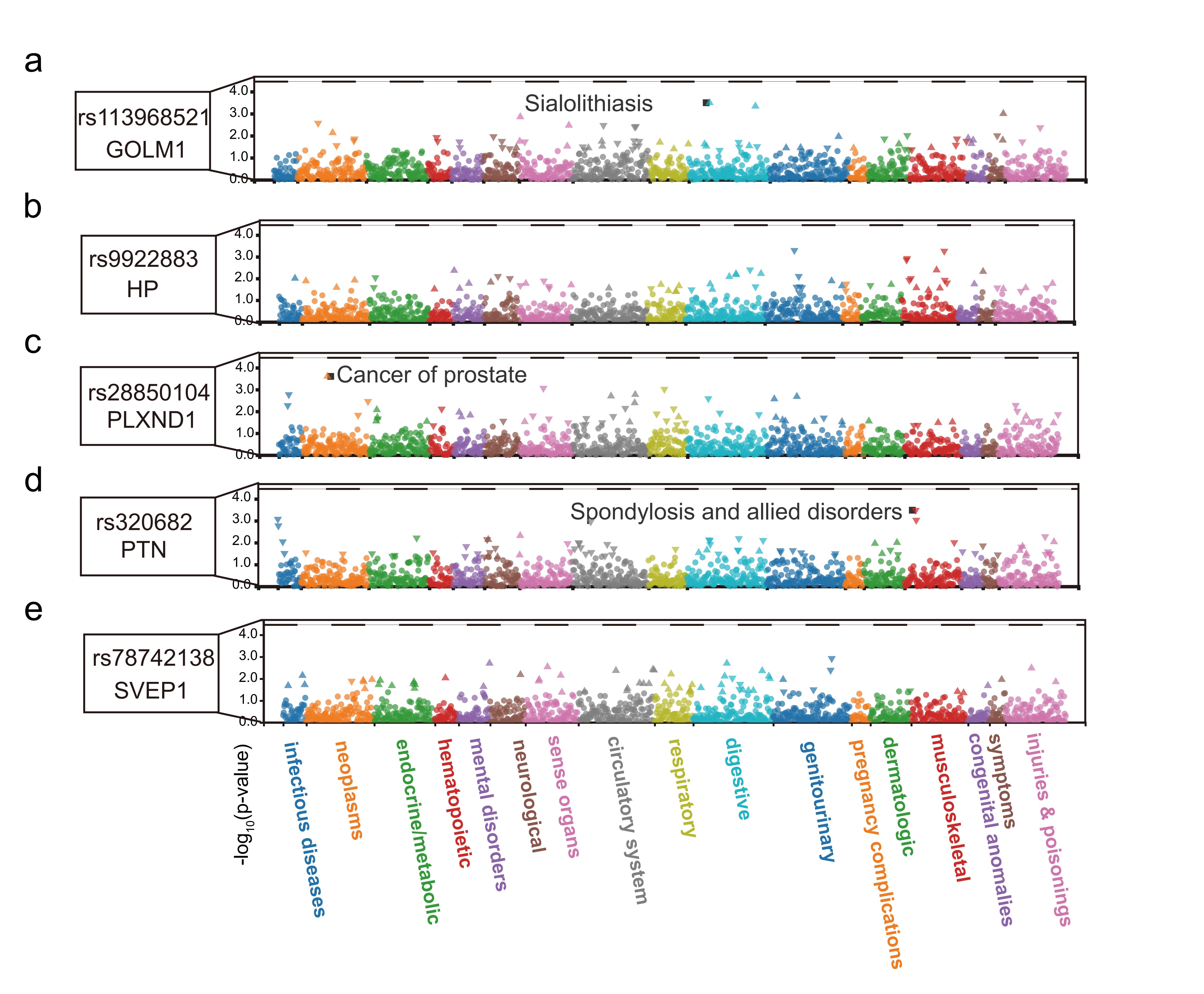

Supplement: Supplementary file 5 — Supplementary Material 5 [file 12967_2025_7086_MOESM5_ESM.jpg]

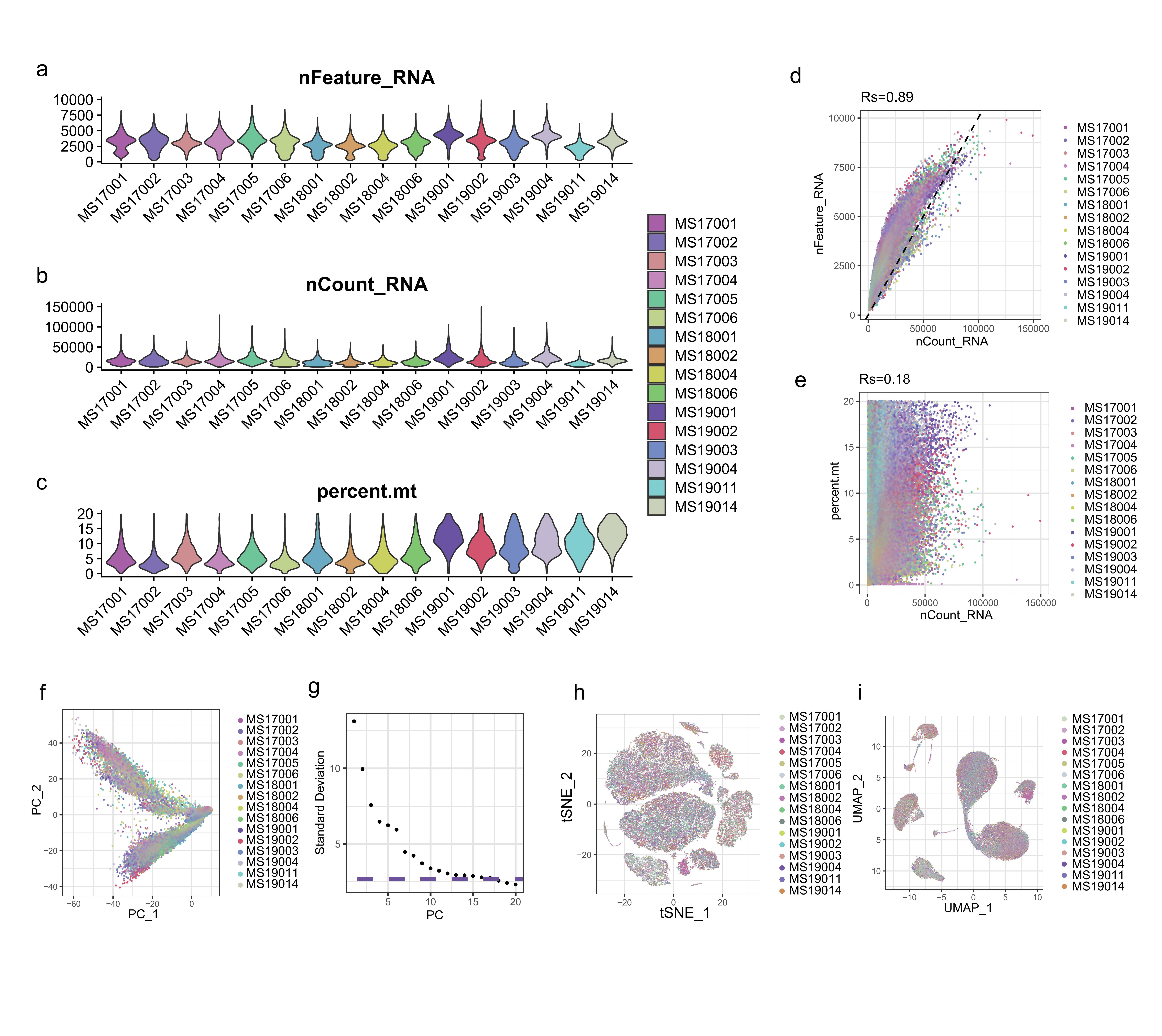

Supplement: Supplementary file 6 — Supplementary Material 6 [file 12967_2025_7086_MOESM6_ESM.jpg]
