# Supplementary material for: A multi-omics Mendelian randomization study reveals PAM as a potential therapeutic target for type 2 diabetes
Source: J Transl Med. 2025 Oct 8;23:1067. doi: 10.1186/s12967-025-07086-x (PMC12506332; doi:10.1186/s12967-025-07086-x)
Supplement: Supplementary file 7 — Supplementary Material 7 [file 12967_2025_7086_MOESM7_ESM.pdf]

Supplementary Table1. A summary of the SNPs used as genetic instruments for conducting the two-sample MR analysis between 586 plasma proteins levels and T2D.

	allele		exposure					outcome								
SNP	effect	other	beta	eaf	se	pval	N	beta	eaf	se	pval	N	exposure	study	r2	F
rs10031141	G	A	0.493	0.4121	0.0060	1.0E-200	35350	0.001	0.3993	0.004	0.7515	2E+06	CXCL6	decode	0.1598	6722.6
rs10069277	G	A	-0.136	0.1027	0.0136	1.5E-23	35341	0.000	0.0706	0.007	0.9726	2E+06	THBS4	decode	0.0028	100.0
rs13167730	T	G	0.375	0.0812	0.0153	5.6E-133	35343	-0.003	0.0914	0.006	0.6388	2E+06	THBS4	decode	0.0168	602.1
rs2404710	A	C	0.093	0.4189	0.0084	3.1E-28	35341	0.010	0.4277	0.004	0.0060	2E+06	THBS4	decode	0.0034	121.4
rs10075736	T	C	0.474	0.1376	0.0062	1.0E-200	35349	-0.002	0.1522	0.005	0.7602	2E+06	ERAP1	decode	0.1425	5873.1
rs76200405	T	C	0.834	0.0228	0.0274	1.0E-200	35349	0.008	0.0344	0.01	0.4360	2E+06	ERAP1	decode	0.0255	926.2
rs10148939	C	A	-0.424	0.1129	0.0127	1.0E-200	35369	0.018	0.1444	0.005	0.0008	2E+06	NID2	decode	0.0305	1114.4
rs754423	T	C	-0.240	0.1808	0.0107	3.1E-112	35367	-0.008	0.234	0.004	0.0570	2E+06	NID2	decode	0.0141	506.8
rs10231308	C	T	0.125	0.2347	0.0096	2.1E-38	35340	0.004	0.2421	0.004	0.3087	2E+06	HSPB1	decode	0.0047	167.9
rs13240755	A	G	0.292	0.3428	0.0085	1.0E-200	35341	0.012	0.6604	0.004	0.0021	2E+06	HSPB1	decode	0.0323	1180.2
rs1036491	C	A	-0.152	0.1705	0.0111	1.2E-42	35388	0.003	0.2127	0.005	0.5720	2E+06	CBLN4	decode	0.0053	187.4
rs6024417	G	A	-0.154	0.2974	0.0090	4.2E-65	35388	-0.008	0.2997	0.004	0.0587	2E+06	CBLN4	decode	0.0081	290.3
rs7351418	T	C	0.154	0.3446	0.0087	5.2E-70	35390	-0.010	0.3232	0.004	0.0117	2E+06	CBLN4	decode	0.0088	312.9
rs5851	G	A	-0.084	0.4556	0.0084	1.9E-23	35382	-0.008	0.4511	0.004	0.0452	2E+06	ACP5	decode	0.0028	99.5
rs1040402	A	G	-0.185	0.1909	0.0106	3.2E-68	35370	0.002	0.1953	0.005	0.7363	2E+06	FBLN1	decode	0.0085	304.6
rs136743	C	A	0.069	0.4453	0.0085	4.1E-16	35369	-0.006	0.4675	0.004	0.1278	2E+06	FBLN1	decode	0.0019	66.2
rs12981737	T	C	-0.197	0.0161	0.0329	2.0E-09	35378	-0.013	0.0294	0.012	0.2898	2E+06	GP6	decode	0.0010	36.0
rs1043784	C	T	-0.195	0.1092	0.0131	3.0E-50	35364	-0.010	0.1192	0.006	0.0766	2E+06	TXNDC5	decode	0.0062	222.2
rs10467359	G	A	-0.319	0.0403	0.0208	6.9E-53	35364	0.016	0.0546	0.008	0.0457	2E+06	KL	decode	0.0066	234.3
rs10501089	T	C	0.163	0.0706	0.0161	6.1E-24	35360	0.007	0.0543	0.009	0.4203	2E+06	BDNF	decode	0.0029	101.8
rs10506379	A	G	-0.124	0.0665	0.0166	8.3E-14	35364	0.010	0.0578	0.008	0.2141	2E+06	LRIG3	decode	0.0016	55.7
rs6581219	G	A	-0.168	0.4788	0.0083	8.1E-91	35366	0.005	0.4218	0.004	0.1539	2E+06	LRIG3	decode	0.0114	408.4
rs10512462	G	A	-0.563	0.0783	0.0155	1.0E-200	35372	-0.002	0.0751	0.007	0.7839	2E+06	CCL8	decode	0.0358	1313.5
rs10853162	G	A	-0.334	0.3240	0.0063	1.0E-200	35373	-0.005	0.6512	0.004	0.2189	2E+06	CCL8	decode	0.0736	2811.0
rs1059367	T	C	-0.126	0.0797	0.0149	4.3E-17	35360	0.023	0.0853	0.007	0.0008	2E+06	HNRNPAB	decode	0.0020	70.6

rs10739734	G	T	-0.091	0.4014	0.0084	7.6E-28	35360	-0.001	0.3757	0.004	0.8284	2E+06	SH3GLB2	decode	0.0034	119.6
rs10814111	A	C	0.182	0.0785	0.0149	2.0E-34	35350	0.011	0.0636	0.008	0.1512	2E+06	NUDT2	decode	0.0042	149.7
rs7025269	T	C	0.300	0.2075	0.0098	1.0E-200	35350	-0.020	0.7779	0.004	0.0000	2E+06	NUDT2	decode	0.0259	941.0
rs10817700	G	A	-0.316	0.3162	0.0089	1.0E-200	35362	0.006	0.3382	0.004	0.1018	2E+06	TNC	decode	0.0346	1265.5
rs10820918	G	A	0.148	0.3138	0.0086	9.6E-67	35358	-0.004	0.3189	0.004	0.3648	2E+06	ROR2	decode	0.0084	297.9
rs10849454	C	T	-0.300	0.3390	0.0089	1.0E-200	35381	0.003	0.2877	0.004	0.4743	2E+06	TAPBPL	decode	0.0308	1125.8
s117244485	C	T	-0.477	0.0244	0.0270	3.4E-70	35388	-0.026	0.0219	0.014	0.0720	2E+06	TAPBPL	decode	0.0088	313.7
rs56983450	A	G	0.717	0.0344	0.0235	1.0E-200	35375	0.004	0.0391	0.01	0.7217	2E+06	TAPBPL	decode	0.0255	926.7
rs10883974	C	T	-0.429	0.2546	0.0067	1.0E-200	35364	-0.006	0.2585	0.004	0.1552	2E+06	GSTO1	decode	0.1031	4066.4
rs805670	T	C	-0.244	0.4399	0.0081	1.0E-200	35362	-0.003	0.417	0.004	0.4451	2E+06	GSTO1	decode	0.0253	917.7
rs10889352	C	T	-0.219	0.3377	0.0085	5.1E-146	35346	-0.004	0.325	0.004	0.3133	2E+06	ANGPTL3	decode	0.0184	662.1
rs10920287	T	G	0.283	0.3508	0.0086	1.0E-200	35346	-0.008	0.3077	0.004	0.0676	2E+06	RNPEP	decode	0.0294	1071.0
rs2678208	G	T	-0.200	0.1412	0.0120	5.4E-63	35347	0.018	0.0925	0.007	0.0095	2E+06	RNPEP	decode	0.0079	280.6
rs10941528	T	C	-0.293	0.2558	0.0096	1.0E-200	35357	-0.007	0.2385	0.004	0.1239	2E+06	C7	decode	0.0257	932.8
rs10972159	A	G	-0.558	0.0311	0.0235	8.3E-125	35350	-0.015	0.0286	0.013	0.2359	2E+06	CNTFR	decode	0.0157	564.6
rs2183012	A	G	0.256	0.0219	0.0286	3.6E-19	35350	-0.006	0.019	0.014	0.6598	2E+06	CNTFR	decode	0.0023	80.1
rs72735302	C	T	-0.207	0.0677	0.0163	5.6E-37	35350	-0.009	0.056	0.008	0.2733	2E+06	CNTFR	decode	0.0045	161.4
rs10973326	A	G	0.260	0.3278	0.0085	1.0E-200	35353	0.000	0.3362	0.004	0.9740	2E+06	GRHPR	decode	0.0257	931.7
rs10981890	G	A	-0.164	0.4940	0.0081	1.6E-90	35346	-0.001	0.466	0.004	0.8762	2E+06	CTSV	decode	0.0114	407.1
rs11002760	G	A	-0.557	0.0327	0.0235	3.9E-124	35357	0.013	0.0396	0.01	0.2182	2E+06	MBL2	decode	0.0156	561.5
rs7475006	C	T	-0.264	0.3884	0.0083	1.0E-200	35361	-0.002	0.5898	0.004	0.6057	2E+06	MBL2	decode	0.0276	1005.0
rs10734190	C	T	0.186	0.2010	0.0105	1.5E-70	35375	0.003	0.8363	0.005	0.6188	2E+06	DKK3	decode	0.0088	315.3
rs11022114	A	G	0.332	0.3333	0.0070	1.0E-200	35377	0.000	0.3172	0.004	0.9752	2E+06	DKK3	decode	0.0605	2278.5
rs11025480	A	G	0.321	0.2133	0.0100	1.0E-200	35370	-0.002	0.2523	0.004	0.6291	2E+06	HTATIP2	decode	0.0285	1035.8
rs11055175	G	A	-1.330	0.0280	0.0202	1.0E-200	35374	0.030	0.0331	0.01	0.0045	2E+06	HEBP1	decode	0.1096	4353.8
rs11055473	T	C	-0.413	0.2940	0.0064	1.0E-200	35385	0.003	0.3018	0.004	0.4536	2E+06	CLEC4C	decode	0.1065	4215.9
rs8035095	T	C	0.074	0.2778	0.0093	1.7E-15	35362	0.000	0.2346	0.004	0.9805	2E+06	RGMA	decode	0.0018	63.4
rs11078596	T	C	-0.173	0.2182	0.0099	1.5E-68	35352	0.004	0.1862	0.005	0.4581	2E+06	SERPINF2	decode	0.0086	306.1
rs11089474	T	G	0.370	0.2032	0.0103	1.0E-200	35364	-0.004	0.2048	0.005	0.4286	2E+06	TCN2	decode	0.0354	1299.1
s112812772	A	G	0.943	0.0220	0.0282	1.0E-200	35365	0.013	0.0291	0.011	0.2315	2E+06	TCN2	decode	0.0307	1118.7
rs2413004	G	A	-0.303	0.3336	0.0089	1.0E-200	35362	0.012	0.3531	0.004	0.0036	1E+06	TCN2	decode	0.0318	1160.1
rs5997658	T	G	-0.298	0.4719	0.0084	1.0E-200	35366	0.004	0.489	0.004	0.2557	2E+06	TCN2	decode	0.0347	1271.0

rs1109980	G	A	0.303	0.0992	0.0135	8.3E-112	35375	0.012	0.0973	0.006	0.0540	2E+06	GLCE	decode	0.0141	504.8
rs12708499	C	T	0.275	0.2902	0.0089	1.0E-200	35376	-0.005	0.714	0.004	0.2551	2E+06	GLCE	decode	0.0264	960.4
rs11103667	T	C	0.116	0.1817	0.0103	4.2E-29	35322	0.019	0.1879	0.005	0.0001	2E+06	OLFM1	decode	0.0035	125.4
rs11107427	A	G	0.402	0.0363	0.0231	1.5E-67	35368	-0.003	0.0235	0.012	0.8251	2E+06	PLXNC1	decode	0.0085	301.6
s11565155(	A	G	-2.044	0.0148	0.0255	1.0E-200	35368	-0.006	0.0182	0.015	0.6834	2E+06	PLXNC1	decode	0.1539	6432.3
rs11111878	A	C	0.400	0.0992	0.0133	3.3E-197	35361	0.007	0.1203	0.006	0.1976	2E+06	HSP90B1	decode	0.0248	897.6
rs1165593	G	A	0.605	0.1690	0.0081	1.0E-200	35361	0.007	0.1573	0.005	0.1814	2E+06	HSP90B1	decode	0.1377	5648.5
s11125703(	T	C	-0.436	0.0865	0.0144	1.0E-200	35363	0.001	0.0964	0.006	0.8208	2E+06	CST4	decode	0.0252	915.7
s11128415(	G	T	0.539	0.0545	0.0187	1.2E-181	35374	0.002	0.0438	0.009	0.8009	2E+06	TIMP3	decode	0.0228	826.0
rs3788507	A	G	-0.464	0.1156	0.0133	1.0E-200	35375	-0.003	0.0922	0.006	0.5933	2E+06	TIMP3	decode	0.0331	1212.6
rs732446	G	T	0.360	0.1650	0.0115	1.0E-200	35374	-0.002	0.2094	0.005	0.7080	2E+06	TIMP3	decode	0.0271	986.0
rs10013945	G	A	-0.301	0.2368	0.0097	1.0E-200	35358	0.009	0.7798	0.005	0.0460	2E+06	IGFBP7	decode	0.0267	968.7
rs11133472	T	C	0.109	0.3637	0.0086	1.4E-36	35358	-0.005	0.3354	0.004	0.1795	2E+06	IGFBP7	decode	0.0045	159.6
s11148679(	A	G	-0.092	0.2155	0.0099	2.3E-20	35372	0.010	0.2033	0.005	0.0363	2E+06	SH3BGRL3	decode	0.0024	85.5
rs6676630	C	T	0.457	0.0876	0.0142	1.0E-200	35372	-0.004	0.9272	0.007	0.5858	2E+06	SH3BGRL3	decode	0.0284	1035.0
rs11158538	T	C	-0.099	0.4366	0.0082	2.2E-33	35347	-0.003	0.4326	0.004	0.3635	2E+06	MTHFD1	decode	0.0041	145.0
s11158759:	T	C	0.650	0.0396	0.0215	1.0E-200	35372	0.007	0.044	0.009	0.4387	2E+06	ADAMTS13	decode	0.0253	916.4
rs41302905	T	C	-0.854	0.0235	0.0271	1.0E-200	35370	-0.001	0.0222	0.013	0.9176	2E+06	ADAMTS13	decode	0.0273	994.1
s11161327(	T	C	1.236	0.0124	0.0377	1.0E-200	35373	-0.001	0.0144	0.016	0.9308	2E+06	MIA	decode	0.0296	1077.1
s18458961:	C	T	0.893	0.0206	0.0289	1.0E-200	35375	-0.008	0.025	0.013	0.5565	2E+06	MIA	decode	0.0263	953.9
rs74746550	C	T	0.894	0.0347	0.0168	1.0E-200	35374	0.010	0.0482	0.009	0.2487	2E+06	MIA	decode	0.0743	2838.2
s11172584	T	C	0.330	0.0626	0.0168	1.1E-85	35356	0.007	0.0517	0.009	0.4120	2E+06	ACE	decode	0.0108	384.9
s14237719:	A	G	0.268	0.0289	0.0251	1.4E-26	35356	0.011	0.0224	0.014	0.4473	2E+06	ACE	decode	0.0032	113.9
s15024195(	G	A	0.337	0.0160	0.0321	7.6E-26	35356	0.025	0.0178	0.015	0.0920	2E+06	ACE	decode	0.0031	110.5
rs4968748	A	G	-0.347	0.2515	0.0093	1.0E-200	35357	-0.009	0.2329	0.004	0.0426	2E+06	ACE	decode	0.0378	1388.0
rs11186737	T	C	-0.291	0.2633	0.0091	1.0E-200	35366	0.019	0.3165	0.004	0.0000	1E+06	FGFBP3	decode	0.0284	1035.1
rs11197747	C	T	-0.576	0.1825	0.0076	1.0E-200	35379	-0.002	0.1554	0.005	0.7431	2E+06	PNLIPRP2	decode	0.1397	5746.2
rs17735613	G	A	0.559	0.1518	0.0082	1.0E-200	35379	0.012	0.1484	0.005	0.0273	2E+06	PNLIPRP2	decode	0.1163	4655.0
s11212648(	A	G	0.283	0.0388	0.0213	3.2E-40	35323	0.006	0.0217	0.013	0.6494	2E+06	DPEP1	decode	0.0050	176.2
rs12918760	G	T	0.224	0.0194	0.0297	4.6E-14	35320	0.049	0.019	0.015	0.0008	2E+06	DPEP1	decode	0.0016	56.9
rs62067101	T	C	-0.445	0.0156	0.0328	6.8E-42	35322	0.004	0.0274	0.012	0.7256	2E+06	DPEP1	decode	0.0052	183.9
rs11214489	T	C	-0.269	0.1876	0.0108	9.0E-137	35364	0.014	0.205	0.005	0.0032	2E+06	NCAM1	decode	0.0172	619.5

rs2288158	G	T	0.327	0.1301	0.0126	2.7E-149	35365	-0.009	0.1358	0.006	0.0945	2E+06	NCAM1	decode	0.0188	677.1
rs61902388	T	C	0.144	0.4515	0.0085	6.4E-64	35362	-0.005	0.4982	0.004	0.1872	2E+06	NCAM1	decode	0.0080	284.8
rs11219769	T	G	-0.204	0.2429	0.0096	5.2E-101	35365	0.005	0.2562	0.004	0.2520	2E+06	ESAM	decode	0.0127	455.2
s112278379	G	T	-0.632	0.0158	0.0350	9.4E-73	35353	0.001	0.023	0.013	0.9239	2E+06	CD177	decode	0.0091	325.4
rs11229039	A	G	-0.474	0.1098	0.0132	1.0E-200	35362	-0.010	0.1293	0.006	0.0683	2E+06	SERPING1	decode	0.0353	1294.7
s117960683	C	T	-0.322	0.0762	0.0158	4.2E-92	35368	-0.004	0.0921	0.007	0.5792	2E+06	SERPING1	decode	0.0116	414.3
s112437560	T	C	0.194	0.0164	0.0320	1.4E-09	35367	0.006	0.0184	0.014	0.6940	2E+06	IL1RL2	decode	0.0010	36.7
s112488423	G	A	0.810	0.0148	0.0336	2.8E-128	35338	-0.010	0.024	0.014	0.4909	2E+06	CFHR4	decode	0.0162	580.6
rs12131802	T	C	-0.126	0.0375	0.0213	3.4E-09	35376	-0.003	0.0543	0.008	0.6837	2E+06	FCRL3	decode	0.0010	35.0
s112735728	C	T	0.695	0.0305	0.0252	6.4E-168	35372	0.014	0.0323	0.011	0.1894	2E+06	SIGLEC12	decode	0.0211	762.8
rs79506121	C	T	0.751	0.0310	0.0245	1.0E-200	35369	-0.006	0.0355	0.01	0.5569	2E+06	SIGLEC12	decode	0.0259	938.6
s112885443	G	T	0.246	0.0727	0.0157	2.0E-55	35375	0.001	0.0567	0.008	0.8675	2E+06	SPINT2	decode	0.0069	245.9
rs6508781	C	A	-0.423	0.1598	0.0085	1.0E-200	35375	-0.003	0.1626	0.005	0.5170	2E+06	SPINT2	decode	0.0648	2452.6
s112963922	T	C	-0.915	0.0112	0.0395	1.2E-118	35371	-0.052	0.0199	0.014	0.0003	2E+06	SERPINA1	decode	0.0149	536.3
s148597299	C	T	0.229	0.0196	0.0308	9.3E-14	35372	0.014	0.0244	0.013	0.2674	2E+06	SERPINA1	decode	0.0016	55.5
rs72702354	G	A	-0.802	0.0299	0.0245	1.0E-200	35372	0.007	0.0369	0.011	0.5266	2E+06	SERPINA1	decode	0.0294	1072.1
s113098364	C	T	-0.372	0.1134	0.0120	1.0E-200	35337	-0.005	0.1247	0.006	0.4003	2E+06	PLA2R1	decode	0.0264	957.5
rs12471260	A	G	-0.246	0.4135	0.0077	1.0E-200	35338	0.003	0.584	0.004	0.3819	2E+06	PLA2R1	decode	0.0279	1015.2
s113286331	T	C	0.520	0.0583	0.0189	8.1E-166	35375	0.009	0.0682	0.008	0.2202	2E+06	ENPP7	decode	0.0208	753.2
s113400434	A	G	0.627	0.0381	0.0207	1.0E-200	35364	0.009	0.0376	0.01	0.3482	2E+06	CTRB2	decode	0.0253	916.4
s113529953	T	C	0.688	0.0401	0.0213	1.0E-200	35372	-0.027	0.0293	0.012	0.0177	2E+06	CEL	decode	0.0286	1041.1
rs1135816	G	A	-0.230	0.3782	0.0081	5.4E-177	35372	0.000	0.4223	0.004	0.9069	2E+06	KLRB1	decode	0.0222	804.6
s114648232	A	C	0.801	0.0501	0.0117	1.0E-200	35381	-0.014	0.0411	0.01	0.1547	2E+06	GMPR	decode	0.1165	4664.9
s114787025	G	T	-0.478	0.0239	0.0284	1.9E-63	35381	0.000	0.0345	0.012	0.9901	2E+06	GMPR	decode	0.0079	282.7
s138143229	A	G	0.694	0.0133	0.0370	1.8E-78	35381	-0.033	0.0139	0.017	0.0479	2E+06	GMPR	decode	0.0098	351.7
s140659976	A	G	-0.456	0.0415	0.0219	1.3E-96	35381	0.004	0.0231	0.014	0.7986	2E+06	GMPR	decode	0.0121	435.0
s141485758	G	A	0.734	0.0359	0.0230	1.0E-200	35381	-0.002	0.022	0.014	0.8966	2E+06	GMPR	decode	0.0280	1020.5
s113856318	C	T	0.738	0.0185	0.0321	1.7E-116	35365	-0.001	0.0185	0.014	0.9472	2E+06	PCOLCE2	decode	0.0147	526.4
rs35351308	A	G	0.398	0.0415	0.0213	1.3E-77	35365	0.004	0.0197	0.014	0.8086	2E+06	PCOLCE2	decode	0.0097	347.8
rs9829564	C	T	-0.423	0.1653	0.0113	1.0E-200	35364	-0.006	0.1719	0.005	0.2484	2E+06	PCOLCE2	decode	0.0380	1395.7
rs11103545	T	G	-0.309	0.2870	0.0097	1.0E-200	35340	0.007	0.7175	0.004	0.1291	2E+06	FCN2	decode	0.0281	1021.0
s113909851	A	G	0.277	0.0646	0.0181	1.0E-52	35333	-0.002	0.0709	0.007	0.8333	2E+06	FCN2	decode	0.0066	233.5

s11395352	A	G	-0.822	0.0288	0.0255	1.0E-200	35365	-0.015	0.0156	0.016	0.3340	2E+06	INHBC	decode	0.0286	1041.6
s11395825	A	G	0.629	0.0619	0.0176	1.0E-200	35372	0.002	0.0699	0.007	0.8197	2E+06	SERPINA12	decode	0.0348	1275.8
rs55911632	G	A	-0.176	0.1618	0.0117	3.5E-51	35371	-0.003	0.1645	0.005	0.5399	2E+06	SERPINA12	decode	0.0064	226.4
s11396852	C	T	0.181	0.1366	0.0121	3.2E-50	35354	-0.002	0.133	0.006	0.7555	2E+06	GOLM1	decode	0.0062	222.1
rs7868110	A	G	0.362	0.0959	0.0142	1.9E-144	35356	-0.003	0.1068	0.006	0.6045	2E+06	GOLM1	decode	0.0182	654.9
s11397913	T	C	-1.350	0.0110	0.0367	1.0E-200	35371	-0.019	0.0194	0.014	0.1575	2E+06	CRISP2	decode	0.0368	1352.0
s11402244	G	A	0.254	0.0191	0.0298	1.8E-17	35373	0.012	0.0348	0.011	0.2702	2E+06	EPHA4	decode	0.0020	72.4
rs4674589	C	T	0.088	0.4270	0.0082	7.8E-27	35373	-0.001	0.4548	0.004	0.7902	2E+06	EPHA4	decode	0.0032	115.0
s11409976	C	T	0.349	0.0225	0.0282	2.6E-35	35365	-0.001	0.0295	0.011	0.9239	2E+06	MYOC	decode	0.0043	153.8
rs7535044	A	G	0.335	0.0188	0.0306	5.5E-28	35362	0.016	0.0175	0.014	0.2734	2E+06	MYOC	decode	0.0034	120.3
s11411643	T	C	1.143	0.0166	0.0316	1.0E-200	35390	-0.004	0.0201	0.015	0.7912	2E+06	CLSTN1	decode	0.0357	1308.8
s11451359	A	G	-0.494	0.0215	0.0286	9.1E-67	35391	0.002	0.0175	0.015	0.9019	2E+06	CLSTN1	decode	0.0083	298.0
s11416250	C	T	-0.486	0.0123	0.0382	4.4E-37	35352	0.004	0.0144	0.016	0.8004	2E+06	AMY2A	decode	0.0046	161.9
s11428644	A	G	-0.722	0.0103	0.0393	2.5E-75	35371	-0.017	0.0158	0.016	0.2679	2E+06	CRELD1	decode	0.0094	337.3
s14436382	A	G	0.560	0.0250	0.0253	7.7E-109	35372	0.011	0.023	0.013	0.4296	2E+06	CRELD1	decode	0.0137	491.2
rs17537486	G	A	0.556	0.0350	0.0213	2.7E-150	35373	0.002	0.0278	0.012	0.8750	2E+06	CRELD1	decode	0.0189	681.8
rs34684179	A	G	-0.642	0.0348	0.0215	3.9E-195	35372	0.012	0.026	0.012	0.3154	2E+06	CRELD1	decode	0.0245	888.0
s11439553	A	G	-0.177	0.0307	0.0238	1.1E-13	35363	-0.002	0.0175	0.015	0.8874	2E+06	TSTD1	decode	0.0016	55.1
rs11580071	A	G	0.411	0.1133	0.0128	1.0E-200	35364	0.006	0.1288	0.006	0.2758	2E+06	TSTD1	decode	0.0284	1032.2
s11442548	T	C	0.265	0.0681	0.0158	5.1E-63	35371	0.007	0.0487	0.009	0.4435	2E+06	CNTNAP2	decode	0.0079	280.8
s11447221	T	C	0.222	0.0411	0.0204	1.4E-27	35366	-0.008	0.0359	0.01	0.4341	2E+06	C9	decode	0.0033	118.4
s11509622	T	C	0.237	0.0119	0.0385	7.5E-10	35367	-0.010	0.0194	0.015	0.5164	2E+06	C9	decode	0.0011	37.9
s11450070	G	A	-0.487	0.0727	0.0161	1.0E-200	35349	-0.006	0.0491	0.009	0.4898	2E+06	AMY2B	decode	0.0253	917.2
rs72861085	G	A	-0.253	0.0197	0.0305	1.1E-16	35377	0.000	0.0245	0.014	0.9896	2E+06	LYVE1	decode	0.0019	68.8
rs76318574	C	T	0.335	0.0243	0.0280	5.4E-33	35373	0.029	0.0277	0.012	0.0120	2E+06	LYVE1	decode	0.0040	143.1
s11492838	A	G	0.259	0.0381	0.0213	8.9E-34	35363	0.007	0.0267	0.012	0.5668	2E+06	CD14	decode	0.0041	146.7
rs5744454	G	T	-0.239	0.3075	0.0088	3.6E-161	35363	-0.012	0.2492	0.004	0.0066	2E+06	CD14	decode	0.0203	731.7
s11496597	A	G	0.251	0.0101	0.0414	1.2E-09	35330	0.000	0.0134	0.017	0.9971	2E+06	ST3GAL6	decode	0.0010	36.9
rs74890720	C	T	0.568	0.0612	0.0165	1.0E-200	35331	-0.002	0.0519	0.009	0.8345	2E+06	ST3GAL6	decode	0.0325	1188.0
s11498654	A	G	0.475	0.0945	0.0148	1.0E-200	35380	-0.012	0.0571	0.008	0.1512	2E+06	PLA2G2A	decode	0.0283	1028.8
rs2063054	T	G	0.271	0.4733	0.0087	1.0E-200	35377	-0.002	0.4404	0.004	0.5530	2E+06	PLA2G2A	decode	0.0266	964.8
s11500041	T	C	-0.442	0.0322	0.0234	1.9E-79	35345	0.008	0.0208	0.013	0.5513	2E+06	HTN3	decode	0.0100	356.2

rs74320783	A	G	-1.267	0.0169	0.0233	1.0E-200	35354	-0.026	0.0119	0.017	0.1352	2E+06	ITIH3	decode	0.0772	2959.5
s115181845	T	C	0.552	0.0779	0.0163	1.0E-200	35345	0.014	0.0682	0.008	0.0838	2E+06	GSTM1	decode	0.0314	1145.1
rs2269340	C	T	0.807	0.0529	0.0138	1.0E-200	35346	0.004	0.0798	0.007	0.5572	2E+06	GSTM1	decode	0.0878	3402.2
s115214168	T	C	1.125	0.0128	0.0369	1.0E-200	35351	-0.010	0.0265	0.012	0.4228	2E+06	CXCL1	decode	0.0256	928.1
s140569632	A	G	0.823	0.0119	0.0382	4.1E-103	35351	0.002	0.0195	0.014	0.8856	2E+06	CXCL1	decode	0.0130	464.9
s115264949	A	G	1.036	0.0259	0.0287	1.0E-200	35351	-0.021	0.0143	0.017	0.2178	2E+06	FCGR2B	decode	0.0356	1306.7
s116769960	T	G	0.794	0.0341	0.0252	1.0E-200	35355	-0.009	0.0213	0.014	0.5031	2E+06	FCGR2B	decode	0.0273	992.2
s115329695	G	A	0.937	0.0232	0.0271	1.0E-200	35366	-0.017	0.0278	0.012	0.1295	2E+06	KNG1	decode	0.0326	1190.3
s144274836	T	C	-0.371	0.1320	0.0120	1.0E-200	35368	-0.002	0.1182	0.006	0.7412	2E+06	KNG1	decode	0.0262	950.0
s115370013	T	C	0.233	0.0658	0.0174	3.7E-41	35261	-0.002	0.0603	0.008	0.8235	2E+06	CNTN4	decode	0.0051	180.5
rs13071423	A	C	0.253	0.1878	0.0111	7.4E-116	35250	0.008	0.1918	0.005	0.1177	2E+06	CNTN4	decode	0.0146	523.4
rs11555096	T	C	-1.476	0.0231	0.0219	1.0E-200	35347	0.000	0.0253	0.012	0.9752	2E+06	FAH	decode	0.1139	4544.6
s115645824	T	C	0.374	0.0292	0.0251	2.7E-50	35357	0.029	0.0295	0.012	0.0110	2E+06	LECT2	decode	0.0063	222.4
rs801576	C	T	0.551	0.0971	0.0102	1.0E-200	35349	-0.001	0.9007	0.007	0.9160	2E+06	LECT2	decode	0.0768	2941.0
rs11591147	T	G	-0.892	0.0121	0.0388	4.0E-117	35363	0.023	0.0209	0.014	0.1059	2E+06	PCSK9	decode	0.0147	529.3
rs472495	T	G	0.157	0.3573	0.0086	8.9E-74	35364	-0.003	0.632	0.004	0.3759	2E+06	PCSK9	decode	0.0093	330.2
s116161634	T	C	-0.605	0.0154	0.0333	7.1E-74	35359	-0.002	0.0217	0.013	0.8940	2E+06	ACP6	decode	0.0093	330.6
s116523799	C	T	0.553	0.0634	0.0173	1.0E-200	35358	0.004	0.9395	0.008	0.6790	2E+06	ACP6	decode	0.0282	1026.7
s143333817	G	A	-0.925	0.0303	0.0246	1.0E-200	35358	-0.021	0.0136	0.018	0.2392	2E+06	ACP6	decode	0.0383	1408.4
s145317206	T	C	-0.958	0.0205	0.0309	1.0E-200	35357	-0.017	0.014	0.017	0.3291	2E+06	ACP6	decode	0.0265	961.3
rs76390540	A	G	-0.641	0.0249	0.0270	1.9E-124	35358	0.029	0.0303	0.011	0.0094	2E+06	ACP6	decode	0.0157	563.0
s116165405	G	T	-0.359	0.0248	0.0271	5.0E-40	35357	0.043	0.0206	0.014	0.0023	2E+06	IDH1	decode	0.0049	175.3
rs73070958	T	C	-0.352	0.0598	0.0168	2.1E-97	35356	-0.019	0.0544	0.009	0.0287	2E+06	IDH1	decode	0.0123	438.6
rs72659937	T	G	-0.306	0.1114	0.0134	1.0E-115	35330	0.007	0.0887	0.007	0.3318	2E+06	ANG	decode	0.0146	522.8
rs11629094	C	T	-0.136	0.2154	0.0100	5.9E-42	35360	0.006	0.2457	0.004	0.1869	2E+06	GALNT16	decode	0.0052	184.2
rs12100668	A	G	0.169	0.4077	0.0083	7.3E-91	35357	-0.016	0.602	0.004	0.0000	2E+06	GALNT16	decode	0.0114	408.6
rs1303451	T	C	-0.201	0.0293	0.0244	2.3E-16	35358	-0.010	0.027	0.013	0.4140	2E+06	GALNT16	decode	0.0019	67.3
rs4392019	T	C	0.751	0.0138	0.0341	2.0E-107	35357	0.003	0.0309	0.011	0.8148	2E+06	STARD5	decode	0.0135	484.7
rs11638020	A	G	0.268	0.4067	0.0086	1.0E-200	35359	-0.003	0.3828	0.004	0.4042	2E+06	CTSH	decode	0.0271	983.7
rs75299846	G	A	-0.572	0.0420	0.0210	5.1E-163	35362	-0.024	0.0583	0.011	0.0212	2E+06	CTSH	decode	0.0205	740.3
rs11667546	T	C	-0.571	0.1728	0.0086	1.0E-200	35372	-0.003	0.1923	0.005	0.5112	2E+06	IL27RA	decode	0.1104	4389.7
rs34317732	T	C	-0.763	0.0222	0.0313	1.1E-131	35369	0.010	0.0216	0.014	0.4676	2E+06	IL27RA	decode	0.0166	596.2

rs2098953	C	T	-0.181	0.0575	0.0178	4.0E-24	35377	0.005	0.0533	0.009	0.5450	2E+06	LILRA2	decode	0.0029	102.6
rs11121209	A	G	0.169	0.3154	0.0096	1.5E-69	35388	0.009	0.6334	0.004	0.0215	2E+06	CA6	decode	0.0087	310.7
s116775713	A	C	0.377	0.0392	0.0232	2.7E-59	35389	-0.005	0.0267	0.012	0.6511	2E+06	CA6	decode	0.0074	263.6
rs35994272	C	T	0.501	0.0117	0.0412	5.7E-34	35386	0.009	0.0168	0.016	0.5678	2E+06	CA6	decode	0.0042	147.6
rs72641551	G	T	-0.423	0.1109	0.0139	1.0E-200	35389	0.007	0.0932	0.007	0.2575	2E+06	CA6	decode	0.0256	928.3
rs11680831	C	T	-0.171	0.3755	0.0082	2.7E-96	35353	-0.001	0.3479	0.004	0.7784	2E+06	NAGK	decode	0.0121	433.6
s116891509	T	C	0.250	0.0759	0.0153	2.7E-60	35375	-0.008	0.075	0.007	0.2312	2E+06	TXNL4B	decode	0.0075	268.3
s116977380	T	C	0.477	0.0376	0.0215	7.2E-109	35319	0.031	0.0467	0.01	0.0012	2E+06	PGP	decode	0.0137	491.3
rs11698358	G	A	0.664	0.0779	0.0101	1.0E-200	35369	0.014	0.0586	0.008	0.0742	2E+06	FLRT3	decode	0.1083	4294.9
s117932517	C	T	-0.353	0.0457	0.0196	1.7E-72	35372	-0.003	0.0367	0.01	0.7925	2E+06	FLRT3	decode	0.0091	324.3
s144808273	G	A	-0.488	0.0278	0.0239	5.4E-93	35369	-0.001	0.0263	0.012	0.9357	2E+06	FLRT3	decode	0.0117	418.4
s145650062	T	G	-0.336	0.0264	0.0250	2.7E-41	35369	0.026	0.0146	0.016	0.1033	2E+06	FLRT3	decode	0.0051	181.1
rs56265150	A	G	0.400	0.1058	0.0129	1.0E-200	35370	0.011	0.0953	0.006	0.0903	2E+06	FLRT3	decode	0.0263	954.0
rs11698530	A	G	-0.665	0.0419	0.0219	1.0E-200	35372	0.000	0.042	0.009	0.9763	2E+06	BPI	decode	0.0254	922.1
rs6069524	C	T	0.297	0.3834	0.0089	1.0E-200	35367	-0.005	0.3556	0.004	0.2052	2E+06	BPI	decode	0.0306	1116.8
s117014247	T	C	-0.725	0.0225	0.0283	1.4E-144	35384	-0.015	0.0217	0.014	0.2846	2E+06	ENGASE	decode	0.0182	655.5
s117502073	A	G	-0.983	0.0382	0.0175	1.0E-200	35384	0.028	0.0306	0.011	0.0112	2E+06	ENGASE	decode	0.0816	3143.5
s117570724	A	G	-0.346	0.0102	0.0429	7.3E-16	35385	0.017	0.0142	0.017	0.3150	2E+06	ENGASE	decode	0.0018	65.1
rs3803780	G	A	-0.600	0.0163	0.0331	3.6E-73	35385	-0.015	0.037	0.01	0.1304	2E+06	ENGASE	decode	0.0092	327.4
s117029024	A	G	-0.565	0.0332	0.0240	1.2E-122	35373	-0.002	0.0182	0.015	0.9106	2E+06	RETN	decode	0.0154	554.6
rs11703790	C	T	0.366	0.1437	0.0121	1.0E-200	35335	0.000	0.1456	0.005	0.9999	2E+06	PLXNB2	decode	0.0254	919.5
rs73183310	G	A	0.126	0.2855	0.0093	1.2E-41	35333	-0.023	0.3274	0.004	0.0000	2E+06	PLXNB2	decode	0.0051	182.7
rs11704345	T	C	-0.120	0.2337	0.0104	8.0E-31	35371	0.003	0.2279	0.004	0.4736	2E+06	APOL3	decode	0.0038	133.2
rs11704700	T	C	0.095	0.2070	0.0104	4.1E-20	35373	-0.012	0.2851	0.004	0.0038	2E+06	SCUBE1	decode	0.0024	84.4
rs695786	T	C	-0.118	0.4901	0.0084	3.9E-45	35371	0.007	0.4781	0.004	0.0582	2E+06	SCUBE1	decode	0.0056	198.8
s117083993	G	A	0.675	0.0359	0.0219	1.0E-200	35368	-0.005	0.0355	0.011	0.6603	2E+06	CLEC11A	decode	0.0260	945.3
s117147437	A	G	1.137	0.0385	0.0171	1.0E-200	35387	0.026	0.0319	0.011	0.0221	2E+06	SAA4	decode	0.1111	4423.4
rs1356979	C	T	-0.381	0.3937	0.0068	1.0E-200	35383	0.007	0.3995	0.004	0.0562	2E+06	SAA4	decode	0.0814	3133.5
rs11727676	C	T	0.235	0.1037	0.0133	5.1E-70	35350	0.040	0.0964	0.006	0.0000	2E+06	HHIP	decode	0.0088	312.9
s117284274	T	G	-0.884	0.0417	0.0132	1.0E-200	35374	0.020	0.0346	0.011	0.0664	2E+06	SIRPB1	decode	0.1120	4462.4
rs17791824	T	C	0.865	0.0315	0.0251	1.0E-200	35373	-0.020	0.0197	0.014	0.1764	2E+06	SIRPB1	decode	0.0326	1190.6
rs62187494	A	G	0.523	0.0553	0.0199	1.2E-151	35373	0.004	0.0476	0.009	0.6319	2E+06	SIRPB1	decode	0.0191	688.1

rs73081790	A	G	-0.415	0.1420	0.0127	1.0E-200	35373	0.011	0.1422	0.005	0.0393	2E+06	SIRPB1	decode	0.0294	1070.4
rs11729397	A	G	-0.259	0.1055	0.0131	6.9E-87	35334	-0.026	0.0928	0.007	0.0001	2E+06	HPGDS	decode	0.0109	390.3
rs28649197	G	T	0.363	0.0202	0.0286	5.6E-37	35334	-0.005	0.0228	0.013	0.6992	2E+06	HPGDS	decode	0.0045	161.4
rs74354031	C	T	-0.850	0.0232	0.0269	1.0E-200	35333	0.025	0.0434	0.011	0.0248	2E+06	HPGDS	decode	0.0276	1002.7
rs78283599	G	A	-0.849	0.0805	0.0086	1.0E-200	35354	-0.027	0.0652	0.008	0.0004	2E+06	CPA4	decode	0.2178	9841.1
rs9641869	G	A	0.380	0.1284	0.0124	1.0E-200	35357	0.004	0.1547	0.005	0.4223	2E+06	CPA4	decode	0.0257	933.5
s11739682!	C	T	0.440	0.0146	0.0354	1.7E-35	35333	0.023	0.0128	0.018	0.1873	2E+06	TFF1	decode	0.0044	154.6
rs225358	T	C	0.290	0.3089	0.0090	1.0E-200	35341	0.005	0.7053	0.004	0.2494	2E+06	TFF1	decode	0.0286	1039.0
s11739900(	A	G	-0.559	0.0268	0.0256	6.5E-106	35360	-0.009	0.0419	0.01	0.3870	2E+06	CA10	decode	0.0133	477.8
rs2106331	A	G	-0.089	0.3198	0.0090	5.2E-23	35362	-0.008	0.3367	0.004	0.0510	2E+06	CA10	decode	0.0028	97.6
rs2938140	T	C	-0.142	0.4604	0.0084	1.8E-64	35360	-0.009	0.4556	0.004	0.0124	2E+06	CA10	decode	0.0081	287.4
rs79833227	A	C	-0.354	0.1857	0.0106	1.0E-200	35371	-0.001	0.1747	0.005	0.8440	2E+06	PLTP	decode	0.0307	1119.7
s11748243!	A	G	-0.671	0.0516	0.0152	1.0E-200	35373	0.014	0.0459	0.009	0.1250	2E+06	PDGFRL	decode	0.0525	1959.6
rs79088892	T	G	-0.411	0.0189	0.0293	8.5E-45	35374	-0.002	0.0327	0.011	0.8377	2E+06	PDGFRL	decode	0.0055	197.2
rs11751347	T	C	-0.375	0.0795	0.0153	3.7E-132	35388	0.013	0.1011	0.006	0.0347	2E+06	PLG	decode	0.0166	598.4
s14212673⁄	A	G	0.250	0.0381	0.0220	4.0E-30	35390	-0.021	0.0481	0.009	0.0198	2E+06	PLG	decode	0.0037	130.0
s11755773⁚	G	A	0.273	0.0452	0.0195	1.9E-44	35366	0.008	0.0324	0.011	0.4567	2E+06	MMP8	decode	0.0055	195.6
rs11757527	A	G	0.291	0.2535	0.0106	1.6E-166	35360	-0.003	0.2898	0.004	0.5165	2E+06	MDGA1	decode	0.0209	756.4
rs1619327	C	T	-0.480	0.1121	0.0147	1.0E-200	35359	-0.008	0.898	0.006	0.2194	2E+06	MDGA1	decode	0.0291	1058.6
rs1776441	A	G	0.229	0.4670	0.0092	1.6E-136	35360	-0.005	0.4814	0.004	0.1563	2E+06	MDGA1	decode	0.0172	618.5
s11765869!	C	T	-0.805	0.0477	0.0155	1.0E-200	35359	-0.027	0.0315	0.011	0.0162	2E+06	NMRAL1	decode	0.0709	2699.9
s11777390⁚	T	C	1.320	0.0172	0.0263	1.0E-200	35364	0.012	0.0279	0.012	0.3250	2E+06	NPTXR	decode	0.0666	2525.1
s11778134(	T	C	-0.337	0.0111	0.0430	4.6E-15	35368	0.012	0.0134	0.016	0.4699	2E+06	CRTAC1	decode	0.0017	61.4
rs684225	A	C	-0.346	0.4852	0.0063	1.0E-200	35367	0.003	0.4265	0.004	0.4637	2E+06	CRTAC1	decode	0.0786	3017.3
s11779931(	T	G	-0.125	0.0324	0.0224	2.2E-08	35319	-0.021	0.0251	0.013	0.0999	2E+06	NME4	decode	0.0009	31.3
rs6600214	T	C	0.163	0.3997	0.0080	4.3E-91	35319	0.011	0.3321	0.004	0.0050	2E+06	NME4	decode	0.0115	409.7
rs75203664	A	C	0.799	0.0183	0.0304	1.3E-152	35375	-0.001	0.0222	0.013	0.9223	2E+06	HP	decode	0.0192	692.4
rs9922883	T	C	0.531	0.0276	0.0245	3.3E-104	35373	-0.001	0.0167	0.015	0.9740	2E+06	HP	decode	0.0131	469.9
s11788624⁚	T	C	-0.750	0.0446	0.0202	1.0E-200	35371	0.012	0.0329	0.011	0.2609	2E+06	LILRB1	decode	0.0375	1376.2
rs11794565	T	C	0.186	0.4398	0.0085	2.5E-105	35359	-0.013	0.4662	0.004	0.0011	2E+06	ENG	decode	0.0133	475.1
rs72748038	T	C	-0.501	0.0528	0.0183	1.5E-164	35371	0.012	0.0489	0.009	0.2042	2E+06	PTGR1	decode	0.0207	747.4
rs74602773	A	G	0.704	0.0316	0.0232	1.0E-200	35371	-0.011	0.0258	0.012	0.3746	2E+06	PTGR1	decode	0.0253	919.0

s117966464	T	G	-0.181	0.0495	0.0189	1.0E-21	35380	-0.006	0.0334	0.011	0.5798	2E+06	SEZ6L	decode	0.0026	91.7
rs6005031	A	G	0.074	0.4536	0.0083	8.8E-19	35381	-0.009	0.5046	0.004	0.0164	2E+06	SEZ6L	decode	0.0022	78.3
s118161729	G	A	-0.581	0.0922	0.0089	1.0E-200	35352	0.001	0.0795	0.007	0.8503	2E+06	SEMA3E	decode	0.1072	4242.8
rs4577905	A	G	0.401	0.0596	0.0169	6.2E-124	35351	-0.003	0.0443	0.009	0.7619	2E+06	SEMA3E	decode	0.0156	560.6
rs11850199	A	C	-0.181	0.2485	0.0095	2.7E-80	35372	-0.002	0.2587	0.004	0.7073	2E+06	SERPINA9	decode	0.0101	360.0
rs11886092	G	A	0.379	0.3949	0.0062	1.0E-200	35369	0.001	0.3749	0.004	0.8699	2E+06	MATN3	decode	0.0963	3770.1
rs3771248	A	G	-0.163	0.3926	0.0084	4.9E-84	35371	0.001	0.5413	0.004	0.7961	2E+06	MATN3	decode	0.0106	377.2
rs9306885	C	T	-0.139	0.2450	0.0096	9.8E-48	35370	0.005	0.2546	0.004	0.2749	2E+06	MATN3	decode	0.0059	210.7
rs11900990	G	T	-0.205	0.1446	0.0118	1.4E-67	35355	0.007	0.1321	0.006	0.2196	2E+06	EDAR	decode	0.0085	301.6
rs77304476	G	A	1.523	0.0103	0.0415	1.0E-200	35354	-0.021	0.0138	0.016	0.1904	2E+06	EDAR	decode	0.0366	1342.9
rs11909509	A	G	-0.165	0.2104	0.0100	1.2E-61	35252	0.001	0.2015	0.005	0.7754	2E+06	COL18A1	decode	0.0077	274.5
rs12024571	T	G	-0.619	0.0306	0.0245	1.6E-140	35347	0.008	0.0499	0.009	0.4108	2E+06	PTGFRN	decode	0.0177	636.8
rs12037202	C	T	-0.362	0.1591	0.0116	1.0E-200	35347	0.008	0.1575	0.005	0.1023	2E+06	PTGFRN	decode	0.0267	970.1
rs75567925	A	G	-0.942	0.0183	0.0315	2.4E-196	35352	-0.002	0.0144	0.017	0.9301	2E+06	PTGFRN	decode	0.0247	893.5
rs12067235	T	G	0.319	0.1683	0.0111	7.3E-181	35374	-0.009	0.2118	0.005	0.0488	2E+06	CNTN2	decode	0.0227	822.3
rs2802840	C	T	-0.515	0.0641	0.0169	1.0E-200	35373	0.000	0.9073	0.007	0.9822	2E+06	CNTN2	decode	0.0255	924.2
rs3903399	C	T	-0.318	0.2478	0.0096	1.0E-200	35375	0.035	0.2122	0.005	0.0000	2E+06	CNTN2	decode	0.0299	1089.9
rs12074147	C	T	-0.287	0.4265	0.0082	1.0E-200	35374	0.001	0.3673	0.004	0.8757	2E+06	PPIE	decode	0.0334	1222.2
rs12117281	C	T	0.136	0.1519	0.0117	1.2E-31	35361	-0.001	0.1163	0.006	0.9152	2E+06	MAN1A2	decode	0.0039	136.9
rs73013841	T	C	-0.296	0.1120	0.0133	7.7E-110	35364	-0.007	0.1173	0.006	0.2500	2E+06	MAN1A2	decode	0.0138	495.8
rs12121180	T	C	0.432	0.1426	0.0117	1.0E-200	35363	-0.015	0.1535	0.005	0.0052	2E+06	IL6R	decode	0.0371	1361.7
s142768042	T	C	-0.548	0.0284	0.0247	6.9E-109	35363	-0.003	0.0281	0.012	0.8048	2E+06	IL6R	decode	0.0137	491.4
rs57569414	A	C	0.527	0.0971	0.0140	1.0E-200	35364	0.003	0.1215	0.006	0.6564	2E+06	IL6R	decode	0.0383	1409.9
rs12129832	C	T	-0.187	0.0423	0.0200	8.1E-21	35336	0.008	0.0446	0.009	0.4046	2E+06	GLRX2	decode	0.0025	87.6
s148212596	G	A	0.768	0.0324	0.0226	1.0E-200	35336	-0.004	0.0194	0.014	0.7624	2E+06	GLRX2	decode	0.0315	1150.8
rs12146099	A	G	0.256	0.3647	0.0084	1.0E-200	35360	0.019	0.3969	0.004	0.0000	2E+06	LAMC2	decode	0.0259	941.1
rs12192369	G	A	-0.212	0.1404	0.0114	8.6E-77	35359	0.002	0.1456	0.005	0.7264	2E+06	PREP	decode	0.0096	344.0
rs12205095	G	T	-0.544	0.3292	0.0061	1.0E-200	35364	0.004	0.359	0.004	0.3268	2E+06	VNN2	decode	0.1860	8082.3
rs10999762	A	G	-0.113	0.2210	0.0098	3.0E-30	35374	0.006	0.7738	0.004	0.1623	2E+06	UNC5B	decode	0.0037	130.7
rs12318199	A	G	0.495	0.0659	0.0157	1.0E-200	35365	-0.029	0.0484	0.009	0.0009	2E+06	ART4	decode	0.0275	999.3
s138585600	T	C	0.524	0.0179	0.0295	6.4E-71	35363	0.015	0.0161	0.016	0.3449	2E+06	ART4	decode	0.0089	317.0
rs12326826	C	T	-0.189	0.2785	0.0095	3.1E-88	35368	0.002	0.2896	0.004	0.7021	2E+06	CNDP1	decode	0.0111	396.5

rs17817077	A	G	0.285	0.3895	0.0086	1.0E-200	35378	0.004	0.389	0.004	0.3374	2E+06	CNDP1	decode	0.0301	1098.2
rs1238449	T	C	-0.159	0.4697	0.0082	8.3E-83	35357	-0.005	0.5039	0.004	0.1499	2E+06	LIPN	decode	0.0104	371.6
rs2576155	T	C	-0.318	0.2912	0.0090	1.0E-200	35357	0.006	0.723	0.004	0.1351	2E+06	LIPN	decode	0.0341	1248.9
rs12423250	T	C	-0.426	0.1721	0.0074	1.0E-200	35364	0.009	0.1787	0.005	0.0708	2E+06	COL2A1	decode	0.0857	3313.2
rs12459073	A	G	-0.342	0.1782	0.0107	1.0E-200	35376	0.004	0.1755	0.005	0.4628	2E+06	IGFLR1	decode	0.0278	1013.0
rs12461127	T	C	0.068	0.4837	0.0084	4.6E-16	35369	0.009	0.4696	0.004	0.0186	2E+06	BCAM	decode	0.0019	66.0
rs8105118	C	T	0.311	0.1191	0.0129	1.2E-127	35351	0.002	0.1293	0.006	0.6980	2E+06	GDF15	decode	0.0161	577.7
rs12535512	C	T	0.258	0.4686	0.0081	1.0E-200	35354	-0.006	0.4358	0.004	0.1332	2E+06	ADAM22	decode	0.0278	1012.6
rs35844181	A	C	0.202	0.3722	0.0084	2.2E-126	35352	-0.003	0.3354	0.004	0.4770	2E+06	ADAM22	decode	0.0159	571.8
rs35186095	T	C	-0.646	0.1699	0.0071	1.0E-200	35377	-0.007	0.1511	0.006	0.1806	2E+06	ICAM1	decode	0.1898	8288.5
rs12635047	A	G	0.155	0.1589	0.0113	4.1E-43	35224	-0.001	0.1502	0.005	0.8780	2E+06	XXYLT1	decode	0.0054	189.5
rs12695049	T	C	0.195	0.4671	0.0082	6.9E-127	35225	0.007	0.5766	0.004	0.0548	2E+06	XXYLT1	decode	0.0160	574.2
rs12657079	C	T	0.329	0.1658	0.0104	1.0E-200	35343	-0.020	0.1606	0.005	0.0002	2E+06	PAM	decode	0.0275	999.2
rs12664015	A	G	-0.336	0.1452	0.0118	1.5E-177	35019	0.007	0.1196	0.006	0.2400	2E+06	THBS2	decode	0.0225	807.1
rs73034020	T	C	0.449	0.1121	0.0130	1.0E-200	35039	0.017	0.0939	0.007	0.0106	2E+06	THBS2	decode	0.0328	1187.3
rs3136630	T	C	-0.328	0.2195	0.0103	1.0E-200	35359	0.007	0.2923	0.004	0.1036	2E+06	IL15RA	decode	0.0276	1003.8
rs74116316	G	A	0.071	0.2210	0.0104	9.9E-12	35362	-0.009	0.2245	0.004	0.0443	2E+06	IL15RA	decode	0.0013	46.4
rs12763713	A	C	-0.243	0.0337	0.0231	9.1E-26	35353	-0.007	0.0306	0.012	0.5292	1E+06	ASAH2	decode	0.0031	110.1
rs146622748	A	G	-0.742	0.0388	0.0213	1.0E-200	35353	0.013	0.0297	0.012	0.2702	2E+06	ASAH2	decode	0.0331	1208.4
rs12877225	A	G	-0.136	0.2563	0.0094	1.9E-47	35339	0.007	0.2882	0.004	0.0873	2E+06	SLITRK5	decode	0.0059	209.3
rs12941038	T	C	-0.158	0.2187	0.0099	1.7E-57	35368	0.014	0.2335	0.004	0.0014	2E+06	FAM20A	decode	0.0072	255.4
rs929477	A	G	-0.202	0.0869	0.0145	7.7E-44	35368	0.001	0.0968	0.006	0.9046	2E+06	FAM20A	decode	0.0054	192.8
rs12950560	C	T	-0.090	0.4382	0.0082	1.2E-27	35368	0.000	0.42	0.004	0.9822	2E+06	CCL3	decode	0.0033	118.6
rs12980031	T	G	-0.292	0.2435	0.0100	2.7E-187	35371	0.007	0.7531	0.005	0.1099	2E+06	FCER2	decode	0.0235	851.9
rs12974746	G	A	-0.542	0.0379	0.0220	1.6E-134	35349	-0.013	0.0278	0.012	0.2916	2E+06	COMP	decode	0.0169	609.2
rs12980552	G	A	-0.269	0.4986	0.0085	1.0E-200	35377	0.004	0.5002	0.004	0.2575	2E+06	CCL25	decode	0.0278	1010.4
rs78039161	C	T	-0.309	0.1118	0.0135	3.0E-116	35377	-0.003	0.1107	0.006	0.6580	2E+06	CCL25	decode	0.0146	525.3
rs12984853	A	G	-0.546	0.4261	0.0062	1.0E-200	35373	-0.003	0.3896	0.004	0.4777	2E+06	SIGLEC9	decode	0.1793	7725.3
rs2072689	T	C	-0.336	0.4473	0.0111	1.0E-200	35372	0.003	0.472	0.004	0.4158	2E+06	SIGLEC9	decode	0.0252	915.1
rs12990312	T	C	-0.212	0.1099	0.0131	7.5E-59	35344	-0.004	0.1262	0.006	0.4313	2E+06	REG1A	decode	0.0073	261.7
rs12999504	G	T	-0.263	0.0370	0.0224	1.2E-31	35344	0.000	0.0403	0.01	0.9917	2E+06	REG1A	decode	0.0039	136.9
rs76841471	G	T	0.527	0.0685	0.0164	1.0E-200	35343	0.001	0.0728	0.007	0.8868	2E+06	REG1A	decode	0.0284	1034.8

rs13008230	G	T	-0.841	0.0506	0.0151	1.0E-200	35359	-0.002	0.0537	0.008	0.8394	2E+06	GKN2	decode	0.0810	3116.7
rs11129766	A	G	0.436	0.0132	0.0360	7.9E-34	35229	0.000	0.9778	0.015	0.9837	2E+06	CHL1	decode	0.0042	147.0
rs13077895	G	A	-0.229	0.3554	0.0086	6.1E-156	35349	0.003	0.3547	0.004	0.5269	2E+06	CHL1	decode	0.0196	707.6
rs13091025	C	A	-0.281	0.1137	0.0129	7.4E-105	35350	0.021	0.1358	0.006	0.0001	2E+06	SEMA3G	decode	0.0132	472.9
rs13100619	C	A	-0.938	0.0507	0.0147	1.0E-200	35369	-0.006	0.0556	0.008	0.4509	2E+06	BTD	decode	0.1034	4077.6
rs13115901	A	G	-0.437	0.2264	0.0077	1.0E-200	35352	-0.004	0.194	0.005	0.4044	2E+06	FGF2	decode	0.0831	3202.4
rs6833731	A	G	0.287	0.2844	0.0091	1.0E-200	35355	0.002	0.3173	0.004	0.5732	2E+06	ENPEP	decode	0.0275	999.9
rs13125919	T	C	0.114	0.1149	0.0128	5.3E-19	35337	0.014	0.0986	0.006	0.0243	2E+06	ADH5	decode	0.0022	79.3
rs28894371	A	G	-0.357	0.0460	0.0192	1.4E-77	35337	-0.025	0.0379	0.01	0.0092	2E+06	ADH5	decode	0.0097	347.6
rs13143783	T	C	-0.404	0.0888	0.0142	1.8E-178	35363	0.002	0.0851	0.007	0.7259	2E+06	SPOCK3	decode	0.0224	811.3
rs77904897	C	A	-0.208	0.0159	0.0324	1.4E-10	35363	0.004	0.0214	0.014	0.7716	2E+06	SPOCK3	decode	0.0012	41.2
rs13144424	A	G	-0.298	0.1597	0.0110	1.2E-162	35363	-0.001	0.201	0.005	0.7851	2E+06	SPARCL1	decode	0.0205	738.6
rs13158921	T	C	-0.505	0.0300	0.0238	5.1E-100	35368	-0.012	0.0171	0.015	0.4116	2E+06	UBLCP1	decode	0.0126	450.7
rs13164140	A	G	0.272	0.2706	0.0091	7.9E-197	35358	0.004	0.2481	0.004	0.3161	2E+06	HEXB	decode	0.0247	895.9
rs13209147	A	G	0.298	0.1884	0.0106	3.8E-173	35357	0.012	0.2	0.005	0.0129	2E+06	MLN	decode	0.0218	786.9
rs2296329	G	A	-0.380	0.1857	0.0107	1.0E-200	35360	-0.021	0.2075	0.005	0.0000	2E+06	MLN	decode	0.0348	1273.9
rs13216122	C	T	-0.574	0.0765	0.0124	1.0E-200	35336	0.000	0.0749	0.007	0.9560	2E+06	RNASET2	decode	0.0572	2143.9
rs13263968	C	T	0.314	0.2003	0.0101	1.0E-200	35360	0.003	0.1869	0.005	0.4846	2E+06	PENK	decode	0.0268	973.1
rs2610035	A	G	0.173	0.4386	0.0082	2.1E-99	35364	-0.018	0.4436	0.004	0.0000	2E+06	PENK	decode	0.0125	447.8
rs1330064	A	G	-0.207	0.3153	0.0087	1.1E-124	35355	-0.002	0.3392	0.004	0.5914	2E+06	GPC5	decode	0.0157	564.1
s139282387	A	G	-0.397	0.1525	0.0113	1.0E-200	35354	-0.012	0.1596	0.005	0.0216	2E+06	GPC5	decode	0.0339	1238.8
rs13342837	T	C	0.321	0.3242	0.0090	1.0E-200	35356	0.008	0.3025	0.004	0.0722	2E+06	SCARF1	decode	0.0351	1284.9
rs13375787	T	C	0.870	0.0388	0.0148	1.0E-200	35354	0.000	0.067	0.009	0.9651	2E+06	CRYZ	decode	0.0893	3466.4
rs277392	G	T	-0.287	0.3323	0.0084	1.0E-200	35353	-0.003	0.2865	0.004	0.5448	2E+06	CRYZ	decode	0.0315	1150.3
rs76296629	T	G	0.479	0.0736	0.0151	1.0E-200	35354	0.012	0.0515	0.009	0.1673	2E+06	CRYZ	decode	0.0277	1008.2
rs1344544	G	A	0.136	0.3062	0.0085	1.5E-57	35370	0.007	0.3175	0.004	0.0739	2E+06	MMAB	decode	0.0072	255.7
rs61898281	G	A	0.543	0.0490	0.0196	1.1E-169	35371	-0.002	0.0509	0.008	0.8473	2E+06	OAF	decode	0.0213	771.0
rs1363864	C	T	-0.081	0.4761	0.0081	2.5E-23	35362	0.002	0.5058	0.004	0.5554	2E+06	ESM1	decode	0.0028	99.0
rs4242051	C	T	0.148	0.2411	0.0094	2.1E-55	35364	-0.002	0.7472	0.004	0.5759	2E+06	ESM1	decode	0.0069	245.9
rs1380642	T	C	-0.302	0.2272	0.0096	1.0E-200	35358	-0.017	0.197	0.005	0.0002	2E+06	MTHFS	decode	0.0273	993.5
rs72736560	A	C	0.240	0.0268	0.0253	1.8E-21	35358	0.013	0.0259	0.012	0.2657	2E+06	MTHFS	decode	0.0026	90.5
s138073243	A	C	0.214	0.0483	0.0198	2.3E-27	35302	0.002	0.0358	0.01	0.8429	2E+06	CPN2	decode	0.0033	117.5

rs6774800	A	C	-0.324	0.2669	0.0094	1.0E-200	35303	-0.004	0.3041	0.004	0.3904	2E+06	CPN2	decode	0.0322	1175.5
s138834887	A	C	0.502	0.0233	0.0274	4.6E-75	35363	-0.020	0.0141	0.017	0.2390	2E+06	UCMA	decode	0.0094	336.0
s139277266	A	G	-0.261	0.0274	0.0243	6.5E-27	35346	0.015	0.0262	0.012	0.2215	2E+06	CD109	decode	0.0033	115.4
rs76081669	T	C	-0.215	0.0466	0.0188	2.8E-30	35346	-0.001	0.0305	0.011	0.9306	2E+06	CD109	decode	0.0037	130.8
rs140174	G	A	-0.284	0.2533	0.0100	2.2E-177	35356	-0.002	0.271	0.004	0.5745	2E+06	IGLL1	decode	0.0223	806.4
s140590049	G	A	-0.477	0.0109	0.0389	1.6E-34	35362	0.012	0.0135	0.016	0.4703	2E+06	CNRIP1	decode	0.0042	150.2
s184832464	T	C	-0.423	0.0303	0.0240	1.0E-69	35358	-0.014	0.0261	0.012	0.2467	2E+06	CNRIP1	decode	0.0087	311.5
rs7592976	G	A	-0.607	0.0794	0.0122	1.0E-200	35362	-0.006	0.0841	0.007	0.3866	2E+06	CNRIP1	decode	0.0657	2487.5
s140864960	A	G	-1.176	0.0372	0.0112	1.0E-200	35344	-0.009	0.0229	0.013	0.5182	2E+06	MST1	decode	0.2382	11052.1
rs34810691	A	G	0.398	0.0346	0.0228	4.8E-68	35344	0.015	0.0259	0.012	0.2216	2E+06	MST1	decode	0.0085	303.8
s141950547	T	C	-0.819	0.0207	0.0292	1.5E-173	35359	0.004	0.0256	0.012	0.7527	2E+06	ADAM23	decode	0.0218	788.7
rs78652356	G	A	0.519	0.0248	0.0261	8.3E-88	35360	0.007	0.0204	0.014	0.6078	2E+06	ADAM23	decode	0.0110	394.5
rs79387475	A	G	0.337	0.1704	0.0109	1.0E-200	35360	-0.007	0.1693	0.005	0.1790	2E+06	ADAM23	decode	0.0261	948.3
s142086573	T	C	0.294	0.0187	0.0313	6.5E-21	35350	0.017	0.0121	0.018	0.3410	2E+06	LUM	decode	0.0025	88.0
rs1803343	C	T	-0.690	0.0175	0.0325	3.8E-100	35349	0.016	0.0214	0.014	0.2272	2E+06	LUM	decode	0.0126	451.3
rs77751442	T	G	-0.375	0.0189	0.0305	1.4E-34	35349	0.009	0.0203	0.013	0.4975	2E+06	LUM	decode	0.0042	150.4
s142332135	A	G	-0.609	0.0499	0.0195	1.0E-200	35362	-0.010	0.025	0.013	0.4582	2E+06	IL16	decode	0.0270	979.7
rs17875585	T	G	0.281	0.0190	0.0309	1.2E-19	35362	-0.007	0.0189	0.014	0.6295	2E+06	IL16	decode	0.0023	82.3
rs4778640	G	A	-1.000	0.0281	0.0195	1.0E-200	35362	0.021	0.0308	0.011	0.0576	2E+06	IL16	decode	0.0695	2639.7
rs72746169	T	C	-0.885	0.0101	0.0409	5.8E-104	35362	-0.017	0.0188	0.015	0.2556	2E+06	IL16	decode	0.0131	468.8
s142439242	G	A	-1.175	0.0278	0.0184	1.0E-200	35363	0.000	0.0152	0.016	0.9934	2E+06	CPQ	decode	0.1031	4064.6
rs72680193	A	C	-0.596	0.0370	0.0219	8.1E-163	35363	0.003	0.0364	0.01	0.7659	2E+06	CPQ	decode	0.0205	739.4
rs76898837	T	G	-0.288	0.0123	0.0386	9.0E-14	35363	-0.008	0.0178	0.015	0.6038	2E+06	CPQ	decode	0.0016	55.6
s143104579	A	G	0.492	0.0195	0.0312	7.9E-56	35376	0.015	0.0188	0.014	0.2841	2E+06	BTN3A3	decode	0.0070	247.8
rs72845505	G	A	-0.193	0.0287	0.0252	1.6E-14	35377	-0.022	0.024	0.013	0.0806	2E+06	BTN3A3	decode	0.0017	59.0
s143257534	T	C	-0.635	0.0344	0.0240	1.9E-154	35367	-0.011	0.0267	0.012	0.3583	2E+06	ADIPOQ	decode	0.0194	700.9
s144444914	A	C	0.443	0.0113	0.0394	2.7E-29	35369	-0.019	0.0235	0.014	0.1566	2E+06	C1QC	decode	0.0036	126.3
rs17452514	T	C	-0.411	0.0258	0.0305	1.4E-41	35356	0.001	0.0207	0.014	0.9556	2E+06	FCGR2A	decode	0.0051	182.4
rs76034548	C	T	-0.802	0.0325	0.0237	1.0E-200	35378	0.024	0.0304	0.011	0.0299	2E+06	ATRN	decode	0.0313	1143.4
s144655897	T	C	0.304	0.0197	0.0269	1.4E-29	35359	-0.022	0.0126	0.017	0.2095	2E+06	OGN	decode	0.0036	127.6
rs62565677	C	T	0.202	0.0733	0.0150	1.2E-41	35358	0.002	0.0551	0.009	0.8528	2E+06	OGN	decode	0.0051	182.7
rs7022820	C	T	0.268	0.3135	0.0084	1.0E-200	35354	-0.011	0.2597	0.004	0.0077	2E+06	OGN	decode	0.0282	1025.4

rs14467497[illegible]	T	C	0.494	0.0139	0.0367	3.1E-41	35396	-0.005	0.0122	0.019	0.8014	2E+06	GREM1	decode	0.0051	180.9
rs17816447	T	C	0.341	0.0303	0.0249	1.3E-42	35396	-0.006	0.0252	0.012	0.6031	2E+06	GREM1	decode	0.0053	187.2
rs4779584	C	T	-0.599	0.1857	0.0084	1.0E-200	35398	0.007	0.7923	0.005	0.1516	2E+06	GREM1	decode	0.1255	5077.7
rs14518803[illegible]	A	G	1.028	0.0176	0.0311	1.0E-200	35347	-0.015	0.019	0.015	0.3176	2E+06	IGFBP3	decode	0.0300	1093.3
rs14576718[illegible]	G	A	-0.343	0.0116	0.0391	1.4E-18	35376	-0.005	0.0146	0.016	0.7778	2E+06	CD300C	decode	0.0022	77.3
rs72844326	G	T	-0.607	0.0422	0.0201	1.4E-200	35377	-0.001	0.0466	0.009	0.9286	2E+06	CD300C	decode	0.0252	913.0
rs14585186[illegible]	A	G	-0.305	0.0371	0.0218	1.6E-44	35358	0.004	0.0224	0.013	0.7585	2E+06	GUSB	decode	0.0055	195.9
rs14617421[illegible]	C	A	-0.537	0.0311	0.0235	2.7E-115	35357	-0.014	0.0264	0.012	0.2243	2E+06	APCS	decode	0.0145	520.9
rs28383573	T	G	0.232	0.1390	0.0119	4.3E-84	35357	-0.009	0.11	0.006	0.1338	2E+06	APCS	decode	0.0106	377.5
rs14638505[illegible]	A	C	-0.204	0.2026	0.0105	4.3E-84	35368	0.020	0.1823	0.005	0.0001	2E+06	MRC2	decode	0.0106	377.5
rs14663700[illegible]	T	C	0.228	0.0456	0.0183	1.6E-35	35356	-0.004	0.0287	0.012	0.7281	2E+06	FUT10	decode	0.0044	154.7
rs4733407	C	T	0.390	0.2766	0.0061	1.0E-200	35358	-0.007	0.264	0.004	0.1009	2E+06	FUT10	decode	0.1029	4055.8
rs7838624	C	A	0.234	0.4443	0.0076	1.0E-200	35356	-0.004	0.4237	0.004	0.3302	2E+06	FUT10	decode	0.0264	959.5
rs14714569[illegible]	T	C	-0.267	0.0225	0.0282	3.4E-21	35348	0.005	0.0222	0.013	0.7215	2E+06	DSG2	decode	0.0025	89.3
rs9304098	T	G	-0.191	0.4542	0.0082	1.6E-119	35348	0.003	0.4554	0.004	0.4711	2E+06	DSG2	decode	0.0151	540.3
rs14723621[illegible]	G	A	0.208	0.0420	0.0203	1.1E-24	35324	-0.013	0.023	0.014	0.3225	2E+06	SMAP1	decode	0.0030	105.1
rs2691477	A	G	-0.312	0.2473	0.0094	1.0E-200	35324	-0.003	0.2869	0.004	0.4624	2E+06	SMAP1	decode	0.0303	1104.4
rs19281877[illegible]	C	A	-0.389	0.0305	0.0254	3.1E-53	35378	-0.016	0.0162	0.017	0.3393	2E+06	ITPA	decode	0.0066	235.9
rs14805437[illegible]	A	G	-1.261	0.0144	0.0352	1.0E-200	35354	0.020	0.011	0.019	0.2819	2E+06	PPIL1	decode	0.0351	1284.1
rs14821573[illegible]	C	T	0.955	0.0190	0.0159	1.0E-200	35373	-0.046	0.0156	0.015	0.0023	2E+06	NAGLU	decode	0.0920	3582.2
rs58947745	A	G	1.137	0.0101	0.0358	1.0E-200	35383	-0.001	0.0197	0.014	0.9464	2E+06	NAGLU	decode	0.0277	1006.6
rs13047780	C	T	-0.103	0.1622	0.0112	3.7E-20	34648	0.025	0.8004	0.005	0.0000	2E+06	COL6A2	decode	0.0024	84.6
rs14833712[illegible]	G	T	0.357	0.0473	0.0193	1.5E-76	34733	0.001	0.0277	0.012	0.9511	2E+06	COL6A2	decode	0.0098	342.9
rs35548026	A	G	-0.387	0.0933	0.0138	2.4E-172	34733	0.029	0.0859	0.007	0.0000	2E+06	COL6A2	decode	0.0221	783.2
rs14854133[illegible]	G	A	1.024	0.0122	0.0431	1.1E-124	35362	0.030	0.0164	0.017	0.0661	2E+06	CHIT1	decode	0.0157	564.1
rs74736715	C	A	0.562	0.0481	0.0185	1.0E-200	35368	0.017	0.051	0.008	0.0387	2E+06	SWAP70	decode	0.0254	921.4
rs14865983[illegible]	A	G	-0.508	0.0134	0.0375	9.0E-42	35362	0.001	0.0143	0.016	0.9493	2E+06	LGMN	decode	0.0052	183.3
rs14993568[illegible]	A	G	0.835	0.0225	0.0267	1.0E-200	35363	-0.038	0.027	0.012	0.0011	2E+06	CTRB1	decode	0.0269	976.0
rs9928744	T	C	0.176	0.2327	0.0096	1.8E-74	35362	0.017	0.199	0.005	0.0002	2E+06	CTRB1	decode	0.0093	333.3
rs15033142[illegible]	G	A	0.732	0.0322	0.0229	1.0E-200	35363	0.007	0.017	0.015	0.6269	2E+06	SERPINA1[illegible]	decode	0.0282	1025.9
rs15039489[illegible]	T	G	-0.781	0.0126	0.0375	2.8E-96	35361	-0.042	0.0102	0.02	0.0361	2E+06	CAPN2	decode	0.0121	433.5
rs15124191[illegible]	T	C	-0.455	0.0194	0.0306	6.5E-50	35360	-0.008	0.0214	0.013	0.5354	2E+06	DPT	decode	0.0062	220.6

rs15125081[illegible]	T	C	0.365	0.0167	0.0336	1.6E-27	35362	-0.015	0.0193	0.014	0.2719	2E+06	DPT	decode	0.0033	118.2
rs580360	T	C	-0.272	0.4937	0.0083	1.0E-200	35362	0.007	0.435	0.004	0.0630	2E+06	DPT	decode	0.0293	1068.0
rs1538821	G	A	0.095	0.0728	0.0152	4.9E-10	35367	-0.003	0.0704	0.007	0.6991	2E+06	ANXA11	decode	0.0011	38.7
rs2152546	T	G	-0.147	0.4544	0.0079	1.7E-76	35368	-0.002	0.4838	0.004	0.6686	2E+06	ANXA11	decode	0.0096	342.7
rs1539790	A	G	-0.131	0.2342	0.0101	6.9E-39	35329	0.005	0.2418	0.004	0.2505	2E+06	COLEC12	decode	0.0048	170.1
rs1561369	A	G	0.527	0.0860	0.0148	1.0E-200	35359	-0.010	0.1141	0.006	0.0892	2E+06	FRZB	decode	0.0346	1267.3
rs72890325	G	T	-0.515	0.0751	0.0159	1.0E-200	35365	-0.004	0.0864	0.007	0.5923	2E+06	FRZB	decode	0.0288	1050.1
rs1650127	T	C	-0.276	0.3150	0.0088	1.0E-200	35360	0.000	0.3087	0.004	0.9966	2E+06	CHST11	decode	0.0272	988.2
rs856595	C	T	-0.097	0.4673	0.0083	3.8E-32	35355	-0.004	0.5563	0.004	0.2420	2E+06	CHST11	decode	0.0039	139.3
rs1675513	G	A	-0.188	0.1390	0.0117	5.6E-58	35330	-0.007	0.1666	0.005	0.1436	2E+06	CPOX	decode	0.0072	257.6
rs16829593	G	T	0.308	0.2630	0.0090	1.0E-200	35346	-0.010	0.2396	0.004	0.0267	2E+06	TNFAIP6	decode	0.0319	1163.3
rs16851364	A	G	-0.240	0.0771	0.0154	1.3E-54	35368	0.004	0.1177	0.006	0.4584	2E+06	FMOD	decode	0.0068	242.1
rs16854533	A	G	0.285	0.1015	0.0138	2.9E-95	35369	0.020	0.102	0.006	0.0021	2E+06	NFASC	decode	0.0120	428.8
rs6656887	C	T	0.515	0.0893	0.0142	1.0E-200	35373	0.001	0.1035	0.006	0.8368	2E+06	NFASC	decode	0.0357	1310.9
rs34411312	T	C	-0.083	0.4597	0.0083	4.3E-23	35383	-0.008	0.5212	0.004	0.0276	2E+06	LIFR	decode	0.0028	97.9
rs16918163	G	A	0.248	0.0917	0.0144	8.1E-67	35343	-0.008	0.1151	0.006	0.1863	2E+06	COL15A1	decode	0.0084	298.2
rs2600261	T	C	0.324	0.2155	0.0101	1.0E-200	35367	0.008	0.7777	0.005	0.0679	2E+06	TIMP4	decode	0.0285	1036.8
rs17077267	T	C	-0.157	0.1648	0.0111	3.7E-45	35345	0.004	0.1489	0.005	0.4837	2E+06	CDCP1	decode	0.0056	198.8
rs17168679	C	T	0.360	0.0139	0.0347	4.3E-25	35354	0.035	0.0239	0.013	0.0056	2E+06	TAC1	decode	0.0030	107.1
rs3779470	A	G	-0.200	0.1831	0.0105	2.5E-80	35366	-0.005	0.1859	0.005	0.3325	2E+06	TAC1	decode	0.0101	360.2
rs13244925	C	A	-0.102	0.4168	0.0083	1.6E-34	35339	0.007	0.5528	0.004	0.0516	2E+06	EGFR	decode	0.0042	150.2
rs17172451	A	G	0.061	0.2713	0.0093	3.3E-11	35339	-0.006	0.2425	0.004	0.1396	2E+06	EGFR	decode	0.0012	44.0
rs17176065	T	C	0.358	0.0447	0.0189	4.5E-80	35361	-0.017	0.0362	0.01	0.0988	2E+06	CYB5D2	decode	0.0101	359.1
rs17288108	G	A	-0.253	0.1424	0.0118	2.3E-101	35364	-0.009	0.174	0.005	0.0725	2E+06	NTN4	decode	0.0128	456.8
rs17383694	G	A	0.167	0.3976	0.0083	6.0E-90	35338	-0.007	0.3658	0.004	0.0836	2E+06	UNC5C	decode	0.0113	404.4
rs17447113	G	T	0.682	0.0440	0.0203	1.0E-200	35349	-0.007	0.0378	0.01	0.5004	2E+06	NTNG1	decode	0.0308	1123.8
rs17554536	A	G	0.088	0.1784	0.0107	1.7E-16	35359	0.005	0.198	0.005	0.3159	2E+06	NID1	decode	0.0019	67.9
rs17599360	G	A	-0.694	0.0347	0.0216	1.0E-200	35356	0.003	0.0505	0.009	0.7802	2E+06	SPINK6	decode	0.0283	1028.5
rs2161431	T	C	-0.330	0.0251	0.0256	6.9E-38	35358	-0.008	0.0319	0.011	0.4711	2E+06	SPINK6	decode	0.0047	165.6
rs17647647	C	T	-0.202	0.1879	0.0106	2.5E-81	35351	0.001	0.2175	0.005	0.8645	2E+06	CPB2	decode	0.0102	364.8
rs2573284	C	T	-0.130	0.3004	0.0089	6.5E-48	35359	0.004	0.6927	0.004	0.3057	2E+06	CPB2	decode	0.0059	211.5
rs7325308	T	C	0.346	0.3966	0.0066	1.0E-200	35357	0.007	0.3872	0.004	0.0691	2E+06	CPB2	decode	0.0712	2709.4

rs34463787	T	C	-0.550	0.2715	0.0057	1.0E-200	35343	-0.003	0.2436	0.004	0.5664	2E+06	MANEA	decode	0.2088	9326.6
rs9403054	G	T	-0.332	0.3627	0.0054	1.0E-200	35342	0.002	0.3571	0.004	0.6933	2E+06	MANEA	decode	0.0962	3760.0
rs17753556	A	C	-0.322	0.1883	0.0105	1.0E-200	35371	0.001	0.1813	0.005	0.9036	2E+06	SERPINA3	decode	0.0259	941.0
rs17783344	G	T	-0.433	0.0954	0.0136	1.0E-200	35333	0.002	0.1362	0.006	0.6975	2E+06	GCA	decode	0.0279	1015.2
rs17850756	A	G	-0.294	0.2793	0.0092	1.0E-200	35368	0.021	0.3169	0.004	0.0000	2E+06	QPCTL	decode	0.0280	1018.5
rs1799886	C	T	0.204	0.4101	0.0086	1.2E-124	35374	0.007	0.433	0.004	0.0711	2E+06	PRSS2	decode	0.0157	563.9
rs1800493	T	C	-0.813	0.0101	0.0399	1.5E-92	35280	0.036	0.0144	0.016	0.0248	2E+06	LRPAP1	decode	0.0117	416.4
rs1838343	T	C	-0.200	0.4596	0.0083	1.1E-127	35348	-0.012	0.437	0.004	0.0017	2E+06	CNTN1	decode	0.0161	577.7
rs4548696	C	A	-0.268	0.1254	0.0126	2.5E-101	35352	0.003	0.1317	0.006	0.6290	2E+06	CNTN1	decode	0.0128	456.7
rs1846934	T	C	0.332	0.4809	0.0097	1.0E-200	35372	0.003	0.4667	0.004	0.4922	2E+06	CD33	decode	0.0322	1177.5
rs2459147	G	A	-0.391	0.4202	0.0058	1.0E-200	35379	-0.013	0.5452	0.004	0.0003	2E+06	CD33	decode	0.1136	4533.3
s186433582	G	A	0.739	0.0351	0.0227	1.0E-200	35357	-0.004	0.019	0.015	0.8012	2E+06	GSTZ1	decode	0.0290	1055.1
rs755072	G	A	0.909	0.0235	0.0260	1.0E-200	35355	-0.003	0.0238	0.012	0.7895	2E+06	GSTZ1	decode	0.0333	1218.1
rs78371502	T	C	-0.356	0.0894	0.0145	4.5E-133	35354	-0.003	0.0582	0.008	0.6832	2E+06	GSTZ1	decode	0.0168	602.6
rs1869085	C	A	-0.095	0.3255	0.0085	5.4E-29	35376	-0.004	0.3179	0.004	0.2690	2E+06	STIM1	decode	0.0035	124.9
s188745628	G	T	0.180	0.0311	0.0244	1.5E-13	35356	-0.011	0.0381	0.01	0.2542	2E+06	B4GALT7	decode	0.0015	54.6
s188904277	A	G	-1.185	0.0102	0.0386	1.0E-200	35366	-0.010	0.0124	0.019	0.6057	2E+06	TREM2	decode	0.0260	944.6
rs2181205	A	G	-0.838	0.0363	0.0144	1.0E-200	35368	-0.021	0.0315	0.011	0.0568	2E+06	SFTPD	decode	0.0870	3371.0
rs4253536	C	T	-0.445	0.0816	0.0146	1.0E-200	35364	-0.008	0.0751	0.007	0.2622	2E+06	SFTPD	decode	0.0256	930.9
rs34231058	A	G	-0.729	0.0303	0.0228	1.0E-200	35339	0.012	0.0441	0.009	0.1885	2E+06	CFHR1	decode	0.0281	1022.2
rs35449482	C	A	-0.746	0.0321	0.0224	1.0E-200	35338	0.003	0.0197	0.014	0.8261	2E+06	CFHR1	decode	0.0303	1105.7
rs1926318	T	C	0.217	0.2111	0.0099	6.8E-108	35364	-0.013	0.2233	0.004	0.0043	2E+06	DCLK1	decode	0.0136	486.8
s192923146	T	G	-0.409	0.0184	0.0315	1.6E-38	35381	0.014	0.0155	0.015	0.3507	2E+06	GFRA2	decode	0.0047	168.5
rs1944270	A	G	0.096	0.3366	0.0085	2.3E-29	35363	-0.002	0.2977	0.004	0.5518	2E+06	SERPINB8	decode	0.0036	126.6
rs3826616	G	A	-0.182	0.4142	0.0082	2.3E-108	35366	0.005	0.5649	0.004	0.1923	2E+06	SERPINB8	decode	0.0136	488.9
rs56321661	A	C	-0.174	0.2097	0.0099	2.5E-69	35365	0.005	0.202	0.005	0.2615	2E+06	SERPINB8	decode	0.0087	309.7
rs1980606	A	G	-0.281	0.1829	0.0109	5.1E-146	35356	0.002	0.2033	0.005	0.6434	2E+06	CD48	decode	0.0184	662.0
rs6427541	T	C	0.088	0.3384	0.0090	8.2E-23	35352	-0.004	0.2947	0.004	0.3238	2E+06	CD48	decode	0.0027	96.7
rs198379	C	T	0.257	0.3965	0.0083	1.0E-200	35387	-0.009	0.4056	0.004	0.0130	2E+06	NPPB	decode	0.0266	965.9
rs2020854	C	T	-0.313	0.0901	0.0146	3.2E-102	35358	-0.010	0.0707	0.007	0.1630	2E+06	APOF	decode	0.0129	460.8
rs2069398	A	G	0.416	0.0940	0.0141	8.2E-191	35366	-0.001	0.0772	0.007	0.8596	2E+06	PMEL	decode	0.0240	868.1
rs2072563	A	G	-0.191	0.3186	0.0087	7.4E-107	35369	0.002	0.3033	0.004	0.6845	2E+06	PGLYRP1	decode	0.0134	482.1

rs2179795	T	G	-0.139	0.2760	0.0092	1.1E-51	35365	-0.003	0.2888	0.004	0.4931	2E+06	PTPRU	decode	0.0064	228.7
rs2205771	A	G	0.226	0.1746	0.0111	2.7E-91	35377	-0.003	0.1797	0.005	0.5952	2E+06	KREMEN1	decode	0.0115	410.6
rs2229475	T	C	0.514	0.0606	0.0174	2.3E-191	35375	-0.006	0.0576	0.008	0.4338	2E+06	HSPG2	decode	0.0240	870.7
rs45589832	A	G	-0.890	0.0391	0.0170	1.0E-200	35371	-0.001	0.0278	0.012	0.9659	2E+06	LBP	decode	0.0722	2750.7
rs73095812	T	C	-0.871	0.0115	0.0410	3.5E-100	35372	-0.002	0.0205	0.014	0.8912	2E+06	LBP	decode	0.0126	451.4
rs2241044	C	A	-0.446	0.4844	0.0054	1.0E-200	35333	0.001	0.5009	0.004	0.8350	2E+06	IL17RA	decode	0.1610	6779.3
rs7287672	A	G	0.311	0.2741	0.0105	2.7E-191	35338	-0.002	0.2984	0.004	0.5650	2E+06	IL17RA	decode	0.0240	870.3
rs2255703	C	T	-0.213	0.3994	0.0083	1.7E-145	35364	-0.021	0.3788	0.004	0.0000	2E+06	PLXND1	decode	0.0183	659.7
rs28850104	T	C	-0.095	0.3184	0.0088	2.0E-27	35362	-0.007	0.2856	0.004	0.0921	2E+06	PLXND1	decode	0.0033	117.7
rs2273185	A	C	-0.244	0.3681	0.0082	3.3E-192	35361	0.003	0.3304	0.004	0.5063	2E+06	ATXN3	decode	0.0241	874.6
rs2277382	T	C	0.316	0.0903	0.0139	4.6E-115	35375	-0.001	0.0866	0.007	0.8747	2E+06	ACVRL1	decode	0.0145	519.8
rs2284031	C	T	-0.328	0.4055	0.0071	1.0E-200	35356	0.000	0.4696	0.004	0.9218	2E+06	CSF2RB	decode	0.0564	2112.9
rs77818863	G	A	0.220	0.0305	0.0249	7.8E-19	35355	0.012	0.0377	0.01	0.2234	2E+06	CSF2RB	decode	0.0022	78.5
rs2292428	C	T	-0.282	0.3833	0.0085	1.0E-200	35393	0.000	0.3962	0.004	0.9928	2E+06	KLKB1	decode	0.0303	1105.5
rs2298087	C	T	-0.359	0.0896	0.0139	1.0E-147	35360	0.004	0.0905	0.007	0.5297	2E+06	ESD	decode	0.0186	669.9
rs2302524	C	T	-0.244	0.1754	0.0108	3.3E-113	35369	0.005	0.159	0.005	0.3341	2E+06	PLAUR	decode	0.0142	511.3
rs4251824	T	C	-0.289	0.0286	0.0244	1.6E-32	35367	0.022	0.036	0.01	0.0331	2E+06	PLAUR	decode	0.0040	141.0
rs2304850	T	C	-0.334	0.2245	0.0098	1.0E-200	35383	0.004	0.2381	0.004	0.3960	2E+06	GAA	decode	0.0316	1153.2
rs77319086	A	C	-0.155	0.0487	0.0195	2.3E-15	35386	0.003	0.0734	0.007	0.6660	2E+06	GAA	decode	0.0018	62.8
rs2304969	T	G	-0.217	0.1471	0.0118	6.0E-75	35379	-0.002	0.1701	0.005	0.7089	2E+06	GLTPD2	decode	0.0094	335.5
rs2305948	T	C	0.330	0.0709	0.0163	1.8E-91	35357	-0.001	0.0975	0.006	0.8719	2E+06	KDR	decode	0.0115	411.4
rs34231037	G	A	-0.886	0.0229	0.0270	1.0E-200	35357	-0.005	0.0322	0.011	0.6253	2E+06	KDR	decode	0.0296	1076.8
rs2307050	A	G	-0.157	0.0779	0.0148	2.4E-26	35334	0.004	0.1046	0.006	0.5669	2E+06	PDGFRA	decode	0.0032	112.8
rs2311597	G	A	-0.271	0.4237	0.0084	1.0E-200	35358	-0.014	0.4106	0.004	0.0002	2E+06	INHBB	decode	0.0288	1046.9
rs2370794	G	A	0.231	0.3272	0.0088	1.4E-151	35371	0.002	0.3417	0.004	0.6340	2E+06	CRTAM	decode	0.0191	687.6
rs7925381	C	T	0.081	0.4869	0.0083	3.5E-22	35371	0.002	0.4466	0.004	0.6886	2E+06	CRTAM	decode	0.0026	93.8
rs2383984	A	G	-0.307	0.2398	0.0095	1.0E-200	35359	-0.010	0.2617	0.004	0.0164	2E+06	NRP1	decode	0.0285	1035.7
rs2434484	G	T	-0.241	0.3393	0.0086	1.9E-172	35372	-0.008	0.329	0.004	0.0399	2E+06	ALKBH3	decode	0.0217	783.7
rs7833351	A	G	-0.268	0.3118	0.0086	1.0E-200	35351	0.005	0.6726	0.004	0.2444	2E+06	CDH17	decode	0.0266	966.6
rs246392	T	C	-0.527	0.3456	0.0062	1.0E-200	35356	0.000	0.2989	0.004	0.9495	2E+06	PDGFRB	decode	0.1680	7140.0
rs72832182	T	C	-0.883	0.0884	0.0104	1.0E-200	35355	0.015	0.0969	0.006	0.0184	2E+06	PDGFRB	decode	0.1701	7246.3
rs2526256	C	T	0.161	0.3903	0.0082	5.4E-86	35349	0.002	0.3965	0.004	0.5695	2E+06	ISOC1	decode	0.0108	386.3

rs2569441	T	C	-0.129	0.4191	0.0088	3.0E-49	35367	-0.001	0.3824	0.004	0.7579	2E+06	KLK14	decode	0.0061	217.6
rs34093024	C	T	-0.305	0.1173	0.0134	2.0E-115	35373	0.008	0.1084	0.006	0.1661	2E+06	KLK14	decode	0.0145	521.4
rs2589775	G	A	-0.179	0.1893	0.0110	3.1E-60	35364	0.001	0.218	0.005	0.8483	2E+06	EGFLAM	decode	0.0075	268.0
rs1320040	C	T	-0.116	0.2204	0.0103	2.4E-29	35372	0.004	0.7829	0.005	0.4439	2E+06	CD300A	decode	0.0036	126.5
rs2706525	A	G	-0.562	0.1796	0.0079	1.0E-200	35377	-0.006	0.1895	0.005	0.1973	2E+06	CD300A	decode	0.1240	5006.7
rs2711897	T	C	0.201	0.3870	0.0080	5.2E-138	35345	-0.029	0.3896	0.004	0.0000	2E+06	BDH2	decode	0.0174	625.3
rs28444377	C	A	0.086	0.2333	0.0093	3.4E-20	35346	-0.011	0.2252	0.004	0.0110	2E+06	BDH2	decode	0.0024	84.7
rs2712355	C	T	-0.288	0.4992	0.0080	1.0E-200	35365	-0.002	0.4514	0.004	0.6055	2E+06	GRAMD1C	decode	0.0351	1285.1
rs2702884	C	T	-0.137	0.4109	0.0084	9.6E-61	35395	0.004	0.6261	0.004	0.3173	2E+06	DEFB1	decode	0.0076	270.3
rs2741108	C	T	-0.261	0.4593	0.0082	1.0E-200	35394	-0.003	0.5275	0.004	0.4750	2E+06	DEFB1	decode	0.0276	1003.8
rs2741712	C	A	-0.085	0.2889	0.0092	2.1E-20	35393	0.003	0.2637	0.004	0.5160	2E+06	DEFB1	decode	0.0024	85.7
rs2755171	G	T	0.091	0.2530	0.0091	3.4E-23	35368	0.005	0.2423	0.004	0.2794	2E+06	CAT	decode	0.0028	98.4
rs769218	A	G	-0.212	0.2199	0.0096	3.0E-107	35374	0.005	0.2207	0.004	0.3044	2E+06	CAT	decode	0.0135	483.9
rs2763257	A	G	0.257	0.4401	0.0082	1.0E-200	35336	0.005	0.412	0.004	0.1742	2E+06	SMOC2	decode	0.0269	976.4
rs58602032	T	C	0.099	0.1434	0.0118	5.1E-17	35326	-0.010	0.1592	0.006	0.0675	1E+06	SMOC2	decode	0.0020	70.3
rs59291571	A	G	-0.292	0.0773	0.0155	1.0E-78	35327	0.007	0.064	0.008	0.3540	2E+06	SMOC2	decode	0.0099	352.8
rs2769265	A	C	-0.327	0.1683	0.0104	1.0E-200	35360	0.017	0.1802	0.005	0.0004	2E+06	PSMB4	decode	0.0269	978.8
rs2838952	T	C	-0.626	0.0562	0.0186	1.0E-200	35247	-0.002	0.0327	0.011	0.8838	2E+06	COL6A1	decode	0.0313	1137.5
rs34627227	T	C	0.365	0.3636	0.0052	1.0E-200	35198	0.004	0.3794	0.004	0.3043	2E+06	COL6A1	decode	0.1229	4930.2
rs28458356	A	G	-0.419	0.1124	0.0130	1.0E-200	35351	0.018	0.1052	0.006	0.0030	2E+06	ENTPD5	decode	0.0287	1044.5
rs28689705	T	G	0.204	0.2448	0.0097	3.3E-98	35324	0.000	0.1969	0.005	0.9884	2E+06	SHANK3	decode	0.0124	442.3
rs9616896	C	T	0.368	0.2425	0.0080	1.0E-200	35328	-0.002	0.217	0.005	0.7460	2E+06	SHANK3	decode	0.0569	2130.6
rs28721898	T	C	-0.465	0.1489	0.0098	1.0E-200	35362	0.000	0.2049	0.005	0.9623	2E+06	FUT3	decode	0.0595	2237.2
rs300275	A	G	-0.125	0.4965	0.0084	3.0E-50	35361	0.008	0.4833	0.004	0.0371	2E+06	FAM151A	decode	0.0062	222.2
rs7545297	A	G	0.242	0.3261	0.0089	1.0E-161	35363	-0.003	0.3548	0.004	0.4023	2E+06	FAM151A	decode	0.0203	734.4
rs300996	T	G	0.242	0.3565	0.0084	9.9E-184	35329	0.002	0.4106	0.004	0.6422	2E+06	CHMP2B	decode	0.0231	835.6
rs3024798	T	G	-0.317	0.3051	0.0093	1.0E-200	35344	0.003	0.3609	0.004	0.4203	2E+06	GNLY	decode	0.0321	1173.1
rs307803	C	T	0.120	0.3844	0.0083	1.7E-47	35330	0.006	0.3913	0.004	0.1495	2E+06	SCGB3A1	decode	0.0059	209.6
rs320682	C	T	0.191	0.4471	0.0081	2.0E-123	35378	-0.016	0.4297	0.004	0.0000	2E+06	PTN	decode	0.0155	558.2
rs62490463	C	T	-0.214	0.0336	0.0219	1.2E-22	35378	0.020	0.036	0.011	0.0733	2E+06	PTN	decode	0.0027	95.9
rs3213120	T	C	-0.922	0.0250	0.0263	1.0E-200	35366	0.016	0.0308	0.011	0.1400	2E+06	IL12B	decode	0.0336	1229.4
rs3773233	T	C	0.223	0.2128	0.0101	1.4E-107	35345	0.011	0.2074	0.005	0.0163	2E+06	ROBO1	decode	0.0135	485.4

rs34000233	A	G	0.416	0.1873	0.0059	1.0E-200	35342	0.003	0.1956	0.005	0.4685	2E+06	TDGF1	decode	0.1234	4972.7
rs34012279	T	G	-0.115	0.2462	0.0094	1.6E-34	35353	0.003	0.2005	0.005	0.5062	2E+06	B3GNT2	decode	0.0042	150.1
rs4073090	G	A	0.160	0.4475	0.0081	1.8E-86	35352	0.017	0.4254	0.004	0.0000	2E+06	B3GNT2	decode	0.0109	388.4
rs34015250	G	A	-0.246	0.0814	0.0146	2.9E-63	35361	0.008	0.0886	0.007	0.2270	2E+06	AP1G2	decode	0.0079	281.9
rs340829	G	T	0.192	0.4698	0.0088	5.9E-107	35368	0.001	0.426	0.004	0.8816	2E+06	IL5RA	decode	0.0135	482.6
rs7635810	T	C	0.292	0.3775	0.0090	1.0E-200	35366	-0.001	0.3374	0.004	0.8770	2E+06	IL5RA	decode	0.0287	1045.8
rs9831674	A	G	-0.270	0.1994	0.0109	8.8E-135	35369	-0.003	0.2083	0.005	0.5376	2E+06	IL5RA	decode	0.0170	610.4
rs34324219	A	C	-0.489	0.1090	0.0132	1.0E-200	35365	0.004	0.1122	0.006	0.4894	2E+06	TCN1	decode	0.0371	1363.2
rs34393987	T	C	0.807	0.0261	0.0253	1.0E-200	35350	-0.005	0.0194	0.014	0.6976	2E+06	SPINK2	decode	0.0280	1018.6
rs11911765	C	T	0.188	0.4768	0.0084	9.2E-110	35363	0.004	0.5229	0.004	0.2768	2E+06	NCAM2	decode	0.0138	495.4
rs7736104	G	T	-0.413	0.3047	0.0070	1.0E-200	35354	0.001	0.6069	0.004	0.7767	2E+06	CRHBP	decode	0.0886	3435.8
rs34497316	A	G	-0.142	0.1437	0.0117	4.3E-34	35367	-0.007	0.1313	0.006	0.2312	2E+06	C1QL1	decode	0.0042	148.2
rs7225162	T	C	-0.202	0.4808	0.0081	1.6E-136	35368	0.000	0.5186	0.004	0.9261	2E+06	C1QL1	decode	0.0172	618.4
rs34511054	C	A	-0.184	0.0569	0.0177	2.3E-25	35291	-0.017	0.057	0.008	0.0378	2E+06	PDCD6	decode	0.0031	108.3
rs34928277	T	C	0.194	0.3447	0.0089	1.0E-105	35389	-0.008	0.2952	0.004	0.0586	2E+06	TLR3	decode	0.0133	476.9
rs34668207	A	G	0.282	0.1111	0.0132	2.0E-101	35381	0.004	0.0986	0.006	0.5451	2E+06	VWA2	decode	0.0128	457.1
rs80113039	A	G	-0.402	0.0195	0.0304	7.4E-40	35381	0.013	0.0306	0.011	0.2296	2E+06	VWA2	decode	0.0049	174.6
rs34695217	C	T	-0.622	0.0504	0.0184	1.0E-200	35363	-0.029	0.048	0.009	0.0015	2E+06	CHRDL2	decode	0.0314	1145.7
rs6592590	T	C	0.149	0.4440	0.0082	5.2E-74	35370	0.005	0.4079	0.004	0.2285	2E+06	CHRDL2	decode	0.0093	331.2
rs34712979	A	G	-0.196	0.2440	0.0092	2.1E-100	35350	-0.013	0.2522	0.004	0.0034	2E+06	NPNT	decode	0.0126	452.5
rs34933869	C	T	0.382	0.1711	0.0109	1.0E-200	35344	-0.009	0.1438	0.005	0.0837	2E+06	CD8A	decode	0.0338	1235.8
rs3014824	G	A	-0.394	0.2456	0.0074	1.0E-200	35360	-0.002	0.7389	0.004	0.7289	2E+06	S100A7	decode	0.0733	2798.3
rs34961571	G	T	0.522	0.0835	0.0149	1.0E-200	35361	0.007	0.0737	0.007	0.3496	2E+06	S100A7	decode	0.0334	1221.3
rs12052464	G	T	-0.090	0.4765	0.0083	2.1E-27	35360	0.005	0.5249	0.004	0.2209	2E+06	DNER	decode	0.0033	117.7
rs35032874	G	T	-0.136	0.2786	0.0091	3.5E-50	35358	-0.004	0.3093	0.004	0.3546	2E+06	DNER	decode	0.0062	221.9
rs35067598	G	A	-0.124	0.0452	0.0195	2.1E-10	35326	-0.006	0.0561	0.008	0.4802	2E+06	ALCAM	decode	0.0011	40.4
rs9834384	C	T	-0.149	0.0437	0.0201	1.4E-13	35326	-0.015	0.032	0.011	0.1742	2E+06	ALCAM	decode	0.0015	54.7
rs35220837	A	G	0.412	0.0758	0.0152	2.0E-160	35363	0.001	0.051	0.008	0.9193	2E+06	CLSTN2	decode	0.0202	728.4
rs4073898	G	T	0.128	0.2845	0.0091	2.7E-44	35364	-0.006	0.2922	0.004	0.1732	2E+06	CLSTN2	decode	0.0055	194.9
rs35383686	A	G	-0.200	0.2314	0.0098	7.1E-92	35374	-0.009	0.2266	0.005	0.0412	2E+06	CXCL16	decode	0.0115	413.3
rs35388278	G	T	-0.260	0.2897	0.0081	1.0E-200	35338	0.001	0.3354	0.004	0.8551	2E+06	RARRES1	decode	0.0282	1023.8
rs35692207	G	A	0.311	0.4676	0.0093	1.0E-200	35384	0.000	0.4568	0.004	0.9526	2E+06	SIGLEC5	decode	0.0306	1117.0

rs3829649	A	G	-0.282	0.4756	0.0093	1.0E-200	35377	0.002	0.5401	0.004	0.6608	2E+06	SIGLEC5	decode	0.0256	927.8
rs35791045	G	A	0.445	0.1781	0.0089	1.0E-200	35377	-0.001	0.1618	0.005	0.8875	2E+06	PSAPL1	decode	0.0660	2499.3
rs62277606	A	G	-0.107	0.4569	0.0086	1.5E-35	35378	-0.001	0.4374	0.004	0.8833	2E+06	PSAPL1	decode	0.0044	154.9
rs35822882	T	G	-1.126	0.0227	0.0223	1.0E-200	35350	-0.005	0.0258	0.012	0.7158	2E+06	CLIC5	decode	0.0672	2548.3
rs36043533	G	T	0.442	0.0414	0.0206	1.6E-102	35382	-0.002	0.0524	0.008	0.7756	2E+06	RSPO1	decode	0.0129	462.2
rs1667515	A	G	-0.082	0.3277	0.0088	1.3E-20	35349	-0.001	0.642	0.004	0.7743	2E+06	FLRT2	decode	0.0024	86.6
rs2747001	G	T	0.168	0.1584	0.0113	2.4E-50	35346	-0.006	0.8422	0.005	0.2807	2E+06	FLRT2	decode	0.0063	222.6
rs36101989	T	G	-0.273	0.2813	0.0091	1.8E-199	35348	-0.007	0.2753	0.004	0.0904	2E+06	FLRT2	decode	0.0250	908.0
rs36187	G	A	0.262	0.4517	0.0080	1.0E-200	35360	-0.007	0.4606	0.004	0.0679	2E+06	EPHB1	decode	0.0296	1080.3
rs57512309	T	G	0.071	0.2737	0.0090	2.6E-15	35360	-0.001	0.2609	0.004	0.8149	2E+06	EPHB1	decode	0.0018	62.5
rs73229138	G	A	0.213	0.0471	0.0188	1.3E-29	35361	-0.008	0.0608	0.008	0.3233	2E+06	EPHB1	decode	0.0036	127.8
rs79903267	C	T	-1.025	0.0230	0.0206	1.0E-200	35361	0.004	0.0253	0.012	0.7543	2E+06	SEMA4D	decode	0.0654	2473.3
rs3733897	G	A	0.473	0.1257	0.0094	1.0E-200	35362	0.019	0.1432	0.005	0.0004	2E+06	TXNDC15	decode	0.0663	2512.1
rs3739613	A	G	-0.276	0.2920	0.0090	1.0E-200	35356	-0.007	0.2946	0.004	0.0824	2E+06	GLIPR2	decode	0.0256	929.7
rs72729437	G	A	0.216	0.0314	0.0235	4.1E-20	35352	-0.003	0.0344	0.01	0.7754	2E+06	GLIPR2	decode	0.0024	84.4
rs3743268	A	G	-0.330	0.3695	0.0066	1.0E-200	35366	0.003	0.347	0.004	0.4779	2E+06	ANXA2	decode	0.0652	2467.6
rs375396	A	C	0.173	0.1727	0.0110	1.7E-55	35353	0.001	0.1972	0.005	0.7653	2E+06	GM2A	decode	0.0069	246.3
rs72794132	T	C	0.321	0.1639	0.0111	3.5E-184	35353	-0.012	0.1647	0.005	0.0144	2E+06	GM2A	decode	0.0231	837.6
rs62350309	G	A	-0.153	0.0918	0.0148	4.1E-25	35377	0.003	0.0672	0.008	0.7147	2E+06	F11	decode	0.0030	107.1
rs3762423	C	T	-0.549	0.0304	0.0229	1.7E-127	35363	0.023	0.0509	0.009	0.0079	2E+06	B4GALT2	decode	0.0161	576.9
rs3772197	G	A	-0.465	0.0902	0.0144	1.0E-200	35351	-0.006	0.0755	0.007	0.4419	2E+06	LRIG1	decode	0.0287	1044.3
rs77048548	G	A	0.254	0.0358	0.0229	9.8E-29	35351	0.006	0.0329	0.011	0.5847	2E+06	LRIG1	decode	0.0035	123.7
rs3782676	T	C	-0.619	0.0921	0.0102	1.0E-200	35385	0.002	0.0726	0.007	0.8316	2E+06	PZP	decode	0.0946	3696.3
rs3825259	G	A	0.384	0.0955	0.0140	2.7E-166	35376	0.006	0.1168	0.006	0.3396	2E+06	MANSC1	decode	0.0209	755.4
rs12462691	C	T	0.123	0.3870	0.0094	8.7E-39	35385	0.008	0.657	0.004	0.0422	2E+06	SIGLEC14	decode	0.0048	169.7
rs2864138	A	G	-0.191	0.4098	0.0093	5.4E-93	35382	-0.005	0.5518	0.004	0.2311	2E+06	SIGLEC14	decode	0.0117	418.4
rs136148	T	C	0.352	0.2486	0.0070	1.0E-200	35358	-0.003	0.6865	0.004	0.5072	2E+06	APOL1	decode	0.0661	2502.0
rs3886200	T	C	0.088	0.4275	0.0082	8.4E-27	35357	0.003	0.4614	0.004	0.5171	2E+06	APOL1	decode	0.0032	114.9
rs61048056	G	A	0.142	0.4396	0.0083	9.8E-67	35344	-0.005	0.5293	0.004	0.1892	2E+06	RGMB	decode	0.0084	297.8
rs41277305	C	A	0.343	0.0349	0.0225	2.1E-52	35369	0.007	0.0356	0.011	0.5201	2E+06	DDT	decode	0.0065	232.1
rs6986061	C	T	0.258	0.3994	0.0081	1.0E-200	35372	0.021	0.5861	0.004	0.0000	2E+06	FGL1	decode	0.0276	1002.5
rs4296866	T	C	-0.123	0.4876	0.0082	6.4E-51	35346	-0.007	0.4512	0.004	0.0708	2E+06	NT5E	decode	0.0063	225.3

rs632350	T	C	-0.532	0.1180	0.0101	1.0E-200	35343	-0.004	0.1152	0.006	0.5406	2E+06	NT5E	decode	0.0723	2752.5
rs433373	G	A	0.380	0.1377	0.0116	1.0E-200	35360	-0.001	0.1223	0.006	0.8385	2E+06	PLEKHA7	decode	0.0294	1070.9
rs436075	C	T	0.285	0.4955	0.0083	1.0E-200	35318	0.009	0.4359	0.004	0.0258	2E+06	DECR2	decode	0.0325	1187.3
rs4384764	A	G	0.221	0.2873	0.0092	3.2E-127	35362	0.003	0.261	0.004	0.5114	2E+06	QPCT	decode	0.0160	575.7
rs4396423	T	C	0.098	0.3510	0.0088	2.4E-28	35341	0.001	0.38	0.004	0.8899	2E+06	PCDH9	decode	0.0034	121.9
rs4457535	G	A	-0.253	0.3720	0.0084	1.0E-200	35348	0.001	0.3836	0.004	0.7138	2E+06	ANGPTL1	decode	0.0253	915.7
rs4459759	A	G	0.407	0.0466	0.0197	1.2E-94	35353	-0.003	0.0575	0.008	0.6741	2E+06	REG3A	decode	0.0119	426.0
rs4464946	A	G	-0.269	0.2739	0.0089	1.0E-200	35349	-0.001	0.245	0.004	0.7777	2E+06	RMDN1	decode	0.0252	914.4
rs4961042	G	T	-0.096	0.3993	0.0082	1.2E-31	35348	-0.006	0.5319	0.004	0.1371	2E+06	RMDN1	decode	0.0039	137.1
rs4468199	G	A	-0.533	0.0938	0.0083	1.0E-200	35344	-0.018	0.0883	0.007	0.0072	2E+06	LEPR	decode	0.1035	4079.0
rs6678033	A	G	-0.681	0.3789	0.0047	1.0E-200	35347	-0.007	0.3848	0.004	0.0731	2E+06	LEPR	decode	0.3758	21282.3
rs45583840	A	G	-0.579	0.0699	0.0191	1.0E-200	35370	0.005	0.0789	0.007	0.5058	2E+06	TMEM190	decode	0.0254	920.5
rs79012440	A	G	-0.360	0.2988	0.0105	1.0E-200	35371	-0.001	0.3031	0.004	0.7698	2E+06	TMEM190	decode	0.0322	1175.9
rs4575	C	T	0.085	0.3082	0.0086	9.1E-23	35377	0.002	0.2867	0.004	0.6402	2E+06	PSME2	decode	0.0027	96.5
rs4610468	C	T	-0.209	0.2299	0.0097	2.4E-102	35357	0.016	0.2664	0.004	0.0003	2E+06	GHR	decode	0.0129	461.4
rs4619875	T	C	0.222	0.3724	0.0084	8.6E-154	35371	-0.002	0.4116	0.004	0.6719	2E+06	ARFIP1	decode	0.0193	697.8
rs4661012	G	T	-0.191	0.3625	0.0086	1.7E-110	35362	-0.001	0.3628	0.004	0.8440	2E+06	PEAR1	decode	0.0139	498.9
rs4674836	A	G	0.336	0.3456	0.0065	1.0E-200	35366	0.000	0.6087	0.004	0.9459	2E+06	SERPINE2	decode	0.0694	2637.9
rs4682481	T	C	0.268	0.1632	0.0111	3.3E-128	35366	0.023	0.1961	0.005	0.0000	2E+06	BOC	decode	0.0161	580.2
rs10768174	A	G	-0.180	0.4050	0.0084	3.1E-103	35368	-0.002	0.5746	0.004	0.5625	2E+06	FJX1	decode	0.0130	465.5
rs474415	A	G	0.107	0.3782	0.0085	1.9E-36	35371	-0.004	0.3305	0.004	0.2769	2E+06	FJX1	decode	0.0045	159.0
rs476336	T	C	-0.256	0.3982	0.0080	1.0E-200	35352	0.003	0.3757	0.004	0.4373	2E+06	POMGNT2	decode	0.0278	1010.1
rs4794183	A	G	0.391	0.1894	0.0107	1.0E-200	35359	-0.005	0.2234	0.004	0.2215	2E+06	WFIKKN2	decode	0.0367	1347.4
rs62078063	C	T	1.322	0.0106	0.0399	1.0E-200	35366	-0.011	0.0299	0.012	0.3317	2E+06	CCL15	decode	0.0301	1098.3
rs4802890	A	G	0.448	0.0878	0.0138	1.0E-200	35366	0.014	0.1123	0.006	0.0284	2E+06	ECH1	decode	0.0289	1053.9
rs4807574	C	T	0.221	0.3616	0.0089	1.7E-135	35338	0.001	0.3395	0.004	0.7889	2E+06	EBI3	decode	0.0171	613.6
rs4894018	A	G	0.298	0.2283	0.0094	1.0E-200	35361	-0.001	0.1999	0.005	0.7889	2E+06	FKBP7	decode	0.0277	1007.7
rs79146658	C	T	-0.180	0.1154	0.0128	4.3E-45	35357	0.018	0.0876	0.007	0.0076	2E+06	FKBP7	decode	0.0056	198.5
rs6444134	T	C	0.201	0.3557	0.0095	1.1E-99	35366	0.010	0.6954	0.004	0.0113	2E+06	HRG	decode	0.0125	449.1
rs66965282	G	A	1.110	0.1130	0.0090	1.0E-200	35367	0.014	0.1432	0.005	0.0083	2E+06	HRG	decode	0.3013	15253.5
rs55662831	A	G	0.087	0.3692	0.0085	2.9E-24	35379	-0.011	0.3592	0.004	0.0066	2E+06	SHBG	decode	0.0029	103.3
rs4848312	G	A	-0.126	0.3123	0.0087	2.7E-47	35360	-0.008	0.6896	0.004	0.0391	2E+06	IL1RN	decode	0.0059	208.6

rs55709272	C	T	-0.178	0.4887	0.0079	1.5E-111	35356	-0.004	0.4202	0.004	0.2416	2E+06	IL1RN	decode	0.0140	503.7
rs557382	G	T	0.133	0.2958	0.0090	2.5E-49	35346	0.008	0.2928	0.004	0.0466	2E+06	NLGN1	decode	0.0061	217.9
rs55986634	A	G	0.261	0.4174	0.0082	1.0E-200	35363	0.001	0.4127	0.004	0.7939	2E+06	DAPK2	decode	0.0281	1023.0
rs56083715	T	C	0.296	0.2403	0.0097	1.0E-200	35276	0.011	0.2156	0.005	0.0201	2E+06	IDUA	decode	0.0257	929.1
rs73201446	A	G	0.400	0.1007	0.0132	1.0E-200	35376	-0.002	0.1075	0.006	0.7256	2E+06	CTSB	decode	0.0252	914.1
rs56336390	T	C	-0.572	0.0589	0.0188	1.0E-200	35363	-0.013	0.0483	0.009	0.1409	2E+06	NQO2	decode	0.0256	928.3
rs62391549	C	T	-0.670	0.1116	0.0096	1.0E-200	35363	-0.015	0.0864	0.007	0.0290	2E+06	NQO2	decode	0.1217	4897.8
rs57362802	C	T	-1.260	0.0138	0.0343	1.0E-200	35358	-0.008	0.0349	0.01	0.4187	2E+06	ADGRF5	decode	0.0367	1346.5
rs5848	T	C	-0.218	0.2556	0.0094	2.1E-119	35370	-0.010	0.2991	0.004	0.0219	2E+06	GRN	decode	0.0150	539.8
rs58509147	T	C	-0.737	0.0480	0.0158	1.0E-200	35158	-0.001	0.0464	0.009	0.8925	2E+06	A1BG	decode	0.0585	2184.2
rs858257	C	T	-0.359	0.0389	0.0213	9.6E-64	35371	0.009	0.0438	0.01	0.3598	2E+06	GPNMB	decode	0.0080	284.1
rs59251421	T	C	-0.402	0.0588	0.0178	2.6E-113	35368	-0.018	0.0744	0.007	0.0121	2E+06	MSR1	decode	0.0143	511.8
rs59477943	G	A	-0.925	0.0350	0.0175	1.0E-200	35337	0.027	0.0384	0.01	0.0061	2E+06	B4GALT6	decode	0.0731	2788.4
rs78796387	T	G	0.478	0.0335	0.0228	8.8E-98	35339	-0.010	0.0348	0.011	0.3372	2E+06	B4GALT6	decode	0.0123	440.4
rs6070664	T	G	-0.606	0.0468	0.0196	1.0E-200	35381	0.030	0.0351	0.01	0.0033	2E+06	CTSZ	decode	0.0262	951.5
rs6072300	T	C	0.159	0.1967	0.0104	7.5E-53	35372	0.030	0.1813	0.005	0.0000	2E+06	EMILIN3	decode	0.0066	234.1
rs78732698	A	C	-0.978	0.0226	0.0285	1.0E-200	35366	-0.007	0.0147	0.016	0.6696	2E+06	CPNE1	decode	0.0322	1178.1
rs61240730	C	T	-0.253	0.1419	0.0123	3.0E-94	35377	0.013	0.1061	0.006	0.0363	2E+06	CR2	decode	0.0118	424.1
rs61765448	T	C	-0.300	0.1711	0.0108	9.0E-169	35355	-0.006	0.1892	0.005	0.2391	2E+06	ROR1	decode	0.0212	766.8
rs61803119	T	G	0.141	0.1066	0.0135	1.6E-25	35362	0.009	0.1075	0.006	0.1423	2E+06	S100A12	decode	0.0031	109.0
rs61806985	T	C	-0.500	0.0770	0.0147	1.0E-200	35364	0.002	0.0463	0.009	0.8174	2E+06	F5	decode	0.0315	1151.4
rs61825157	T	C	0.356	0.0672	0.0161	2.6E-108	35358	-0.002	0.0485	0.009	0.7885	2E+06	GUK1	decode	0.0136	488.8
rs61885329	A	G	0.364	0.0369	0.0217	8.1E-63	35364	-0.012	0.0504	0.009	0.1498	2E+06	FOLH1	decode	0.0079	279.8
rs7123666	G	A	0.269	0.1158	0.0128	2.7E-98	35376	-0.023	0.8911	0.006	0.0001	2E+06	APOA5	decode	0.0124	442.8
rs619833	A	G	-0.210	0.0424	0.0209	1.2E-23	35372	0.004	0.0359	0.01	0.6699	2E+06	KIRREL2	decode	0.0028	100.4
rs61993080	T	C	-0.174	0.0627	0.0167	2.4E-25	35362	0.003	0.0749	0.007	0.6846	2E+06	MDGA2	decode	0.0031	108.2
rs62037104	A	C	-0.651	0.0262	0.0256	4.0E-142	35373	0.019	0.0196	0.014	0.1636	2E+06	CCL22	decode	0.0179	644.2
rs9921051	A	G	0.266	0.0592	0.0174	9.7E-53	35373	-0.017	0.0703	0.007	0.0220	2E+06	CCL22	decode	0.0066	233.6
rs62087497	A	G	0.174	0.0835	0.0148	1.2E-31	35369	-0.005	0.0749	0.007	0.4568	2E+06	TWSG1	decode	0.0039	137.0
rs62115743	T	C	-1.346	0.0695	0.0121	1.0E-200	35371	0.007	0.0881	0.007	0.3049	2E+06	KLK11	decode	0.2581	12306.1
rs62162757	A	C	0.195	0.3279	0.0087	1.3E-111	35342	-0.001	0.3197	0.004	0.7571	2E+06	CAPG	decode	0.0141	504.0
rs950362	A	G	-1.208	0.0169	0.0252	1.0E-200	35344	0.005	0.0123	0.017	0.7561	2E+06	CAPG	decode	0.0608	2288.8

rs62184386	T	C	0.242	0.4758	0.0079	1.0E-200	35369	-0.016	0.4491	0.004	0.0000	2E+06	HIBCH	decode	0.0259	941.9
rs3804749	T	C	-0.631	0.3447	0.0066	1.0E-200	35366	-0.002	0.5932	0.004	0.6982	2E+06	PDIA5	decode	0.2068	9220.3
rs62263781	G	A	0.321	0.0245	0.0266	1.5E-33	35369	0.001	0.0175	0.015	0.9374	2E+06	PDIA5	decode	0.0041	145.7
rs6232	C	T	-0.624	0.0868	0.0108	1.0E-200	35342	0.018	0.0533	0.008	0.0308	2E+06	PCSK1	decode	0.0864	3343.3
rs76239650	A	C	-0.491	0.1081	0.0097	1.0E-200	35342	-0.008	0.1052	0.006	0.2145	2E+06	PCSK1	decode	0.0674	2554.7
rs62376423	A	G	-0.353	0.0377	0.0194	6.8E-74	35349	-0.008	0.031	0.011	0.4983	2E+06	ERAP2	decode	0.0093	330.7
rs7705528	G	A	-0.213	0.3364	0.0077	1.6E-168	35345	0.010	0.3799	0.004	0.0091	2E+06	ERAP2	decode	0.0212	765.6
rs7709763	C	T	-0.296	0.2278	0.0086	1.0E-200	35348	-0.003	0.2049	0.005	0.5216	2E+06	ERAP2	decode	0.0321	1173.0
rs62642270	G	A	-0.129	0.1617	0.0115	1.5E-29	35356	0.001	0.16	0.005	0.8940	2E+06	SFRP1	decode	0.0036	127.4
rs72643819	T	G	-0.158	0.4181	0.0085	5.9E-77	35354	0.005	0.4002	0.004	0.1629	2E+06	SFRP1	decode	0.0097	344.8
rs62642596	T	C	0.209	0.4859	0.0086	2.3E-130	35385	0.009	0.4776	0.004	0.0152	2E+06	H6PD	decode	0.0164	590.2
rs641320	A	G	-0.551	0.0884	0.0126	1.0E-200	35362	0.009	0.0678	0.008	0.2150	2E+06	FAIM	decode	0.0511	1905.6
rs6492108	C	A	0.051	0.3387	0.0086	3.6E-09	35356	-0.005	0.3668	0.004	0.2418	2E+06	TNFSF13B	decode	0.0010	34.8
rs6510263	G	A	0.308	0.2727	0.0090	1.0E-200	35358	-0.016	0.2707	0.004	0.0002	2E+06	PDCD5	decode	0.0316	1155.2
rs6600145	G	A	-0.127	0.0793	0.0153	7.6E-17	35305	-0.016	0.0841	0.007	0.0159	2E+06	GNPTG	decode	0.0020	69.5
rs6602909	C	T	-0.260	0.3381	0.0087	1.9E-195	34860	0.001	0.3333	0.004	0.7946	2E+06	GAS6	decode	0.0249	889.4
rs6671362	G	A	0.199	0.4572	0.0080	2.6E-136	35356	-0.002	0.4797	0.004	0.6190	2E+06	CREB3L4	decode	0.0172	617.5
rs6686906	A	G	0.098	0.3810	0.0085	7.6E-31	35370	0.001	0.3613	0.004	0.7985	2E+06	EPHB2	decode	0.0038	133.3
rs6699769	G	A	-0.192	0.1848	0.0103	1.3E-77	35355	0.002	0.2025	0.005	0.6903	2E+06	DNAJB4	decode	0.0097	347.7
rs2326055	T	C	0.145	0.2212	0.0100	2.4E-47	35377	0.003	0.795	0.005	0.5257	2E+06	CPXM1	decode	0.0059	208.9
rs67702963	C	T	-0.143	0.2156	0.0102	1.7E-44	35365	0.000	0.2345	0.004	0.9357	2E+06	MMP7	decode	0.0055	195.9
rs6918969	C	T	-0.205	0.2526	0.0099	1.2E-94	35372	0.017	0.2693	0.004	0.0000	2E+06	TREML2	decode	0.0119	426.0
rs991762	T	C	0.334	0.2080	0.0105	1.0E-200	35371	-0.010	0.1965	0.005	0.0247	2E+06	TREML2	decode	0.0276	1004.4
rs7019909	T	C	0.295	0.0923	0.0140	5.4E-99	35340	-0.007	0.1042	0.006	0.2297	2E+06	B4GALT1	decode	0.0125	445.9
rs7116230	A	G	0.271	0.3576	0.0086	1.0E-200	35370	0.000	0.3767	0.004	0.9385	2E+06	SPON1	decode	0.0275	1001.2
rs562672	T	C	0.095	0.3721	0.0085	9.4E-29	35367	-0.001	0.5888	0.004	0.8288	2E+06	CDON	decode	0.0035	123.8
rs657225	A	G	-0.139	0.0880	0.0148	5.7E-21	35367	-0.001	0.9017	0.006	0.8526	2E+06	CDON	decode	0.0025	88.3
rs7120521	T	C	-0.665	0.0758	0.0125	1.0E-200	35368	0.020	0.0908	0.007	0.0022	2E+06	CDON	decode	0.0744	2841.8
rs7122082	A	G	-0.135	0.4578	0.0084	1.3E-57	35375	0.002	0.4203	0.004	0.6379	2E+06	CYB5R2	decode	0.0072	255.9
rs75667685	A	G	0.118	0.0402	0.0210	2.1E-08	35374	0.001	0.0546	0.008	0.8779	2E+06	CYB5R2	decode	0.0009	31.4
rs7131073	G	A	0.073	0.3524	0.0086	2.4E-17	35359	-0.006	0.3428	0.004	0.1149	2E+06	CLMP	decode	0.0020	71.8
rs7946718	G	A	0.211	0.4079	0.0083	3.1E-142	35355	0.004	0.5931	0.004	0.3355	2E+06	CLMP	decode	0.0179	644.6

rs4528296	T	C	-0.426	0.0744	0.0152	7.6E-173	35355	-0.023	0.9226	0.007	0.0009	2E+06	CNTN5	decode	0.0217	785.5
rs7131217	G	A	0.089	0.2085	0.0099	1.4E-19	35353	0.005	0.2186	0.005	0.2703	2E+06	CNTN5	decode	0.0023	82.0
rs7135211	A	G	-0.246	0.3741	0.0083	1.6E-193	35365	-0.007	0.3595	0.004	0.0765	2E+06	MGP	decode	0.0243	880.6
rs7159420	G	T	0.429	0.1198	0.0129	1.0E-200	35362	0.002	0.1231	0.006	0.6943	2E+06	COCH	decode	0.0303	1105.4
rs7204669	A	G	0.239	0.2951	0.0090	7.1E-155	35321	0.009	0.313	0.004	0.0244	2E+06	PRSS22	decode	0.0195	702.9
rs7247115	A	G	-0.153	0.2021	0.0107	8.2E-47	35371	0.013	0.1998	0.005	0.0052	2E+06	OLFM2	decode	0.0058	206.4
rs74178184	A	G	-0.268	0.2214	0.0103	5.7E-148	35367	-0.003	0.2161	0.005	0.5936	2E+06	OLFM2	decode	0.0186	671.0
rs72697218	T	C	-0.225	0.0242	0.0267	4.4E-17	35352	0.038	0.032	0.011	0.0005	2E+06	REG4	decode	0.0020	70.6
rs72704117	T	C	0.289	0.0299	0.0245	4.9E-32	35365	-0.005	0.0206	0.014	0.7041	2E+06	THBS3	decode	0.0039	138.8
rs72709664	A	C	-0.530	0.0564	0.0174	1.0E-200	35348	-0.034	0.0885	0.007	0.0000	2E+06	PRSS3	decode	0.0257	930.6
rs72712829	C	T	0.302	0.0944	0.0142	2.0E-99	35364	0.003	0.0737	0.008	0.7148	2E+06	LY9	decode	0.0125	447.9
rs72715776	T	G	-0.135	0.4410	0.0081	9.1E-63	35355	0.007	0.4225	0.004	0.0842	2E+06	PCDH10	decode	0.0078	279.6
rs72729191	C	T	1.394	0.0875	0.0097	1.0E-200	35371	-0.005	0.0867	0.007	0.4563	2E+06	SEMA5A	decode	0.3700	20769.5
rs2072528	C	T	-0.268	0.3510	0.0089	1.0E-200	35362	-0.001	0.6148	0.004	0.8271	2E+06	VIT	decode	0.0252	915.3
rs72824579	A	G	-0.766	0.0267	0.0253	1.0E-200	35337	-0.008	0.0338	0.011	0.4689	2E+06	MGAT4B	decode	0.0253	918.8
rs72835078	T	G	0.254	0.0817	0.0148	1.9E-65	35379	0.019	0.0828	0.007	0.0053	2E+06	GP1BA	decode	0.0082	291.9
rs72858937	T	C	-0.752	0.0139	0.0361	1.5E-96	35353	-0.003	0.0228	0.013	0.8377	2E+06	AFM	decode	0.0121	434.7
rs73191242	A	G	-0.282	0.2105	0.0102	3.3E-167	35364	0.004	0.2054	0.005	0.4210	2E+06	SELPLG	decode	0.0210	759.6
rs72988065	G	A	0.168	0.1893	0.0102	1.2E-60	35349	0.001	0.1676	0.005	0.7958	2E+06	GFRAL	decode	0.0076	270.0
rs12461895	C	A	0.292	0.4090	0.0085	1.0E-200	35372	-0.002	0.5795	0.004	0.5873	2E+06	B3GNT8	decode	0.0327	1196.8
rs73045078	A	G	-0.459	0.0471	0.0199	5.1E-117	35381	-0.015	0.0312	0.012	0.2066	2E+06	B3GNT8	decode	0.0147	528.8
rs73047241	C	A	-0.500	0.0463	0.0197	3.6E-142	35373	-0.050	0.0253	0.013	0.0002	2E+06	B3GNT8	decode	0.0179	644.4
rs73202262	G	A	0.231	0.0320	0.0231	1.8E-23	35384	0.009	0.0319	0.011	0.4162	2E+06	CBR3	decode	0.0028	99.6
rs73217917	C	T	-0.964	0.0283	0.0205	1.0E-200	35372	0.020	0.0178	0.015	0.1711	2E+06	PEBP1	decode	0.0590	2215.8
rs73287817	G	A	0.298	0.2591	0.0093	1.0E-200	35372	0.017	0.2482	0.004	0.0001	2E+06	CCDC126	decode	0.0280	1017.3
rs73347621	T	C	-0.392	0.1329	0.0121	1.0E-200	35341	0.009	0.1332	0.006	0.1020	2E+06	VWC2	decode	0.0288	1047.8
rs79523632	T	C	-0.397	0.0286	0.0254	3.7E-55	35344	0.038	0.026	0.012	0.0013	2E+06	VWC2	decode	0.0069	244.7
rs73407610	G	A	-0.512	0.0232	0.0270	1.9E-80	35352	-0.004	0.031	0.011	0.6943	2E+06	DEF6	decode	0.0101	360.7
rs73801515	T	C	-0.389	0.0502	0.0186	1.1E-97	35362	-0.008	0.0407	0.01	0.4325	2E+06	BST1	decode	0.0123	440.0
rs7409311	A	G	-0.432	0.1142	0.0124	1.0E-200	35377	0.007	0.0909	0.007	0.2962	2E+06	LHB	decode	0.0332	1214.5
rs74578793	A	G	0.615	0.0539	0.0183	1.0E-200	35274	0.001	0.0493	0.009	0.9112	2E+06	GPC1	decode	0.0310	1126.9
rs7459882	G	A	0.099	0.4966	0.0081	5.4E-34	35342	0.003	0.4876	0.004	0.3968	2E+06	GGH	decode	0.0042	147.8

rs77291996	C	T	0.590	0.0588	0.0170	1.0E-200	35344	-0.002	0.0719	0.007	0.8293	2E+06	GGH	decode	0.0330	1204.4
rs74617719	C	T	-0.441	0.0491	0.0179	8.2E-134	35355	-0.008	0.0462	0.009	0.3526	2E+06	NAAA	decode	0.0169	606.0
rs75003668	G	A	-1.463	0.0137	0.0300	1.0E-200	35370	0.019	0.0326	0.011	0.0816	2E+06	APOH	decode	0.0628	2371.7
rs9912634	T	C	0.330	0.0370	0.0215	3.1E-53	35368	0.008	0.0435	0.009	0.3749	2E+06	APOH	decode	0.0066	235.9
rs175111	T	C	0.150	0.3623	0.0086	2.9E-68	35368	-0.006	0.612	0.004	0.1011	2E+06	'MEM132/	decode	0.0085	304.8
rs75210230	G	T	-0.354	0.0110	0.0401	1.1E-18	35370	-0.018	0.015	0.016	0.2536	2E+06	'MEM132/	decode	0.0022	77.8
rs7525733	G	T	-0.269	0.2913	0.0086	1.0E-200	35366	-0.003	0.2518	0.004	0.4584	2E+06	UROD	decode	0.0269	977.0
rs2795496	A	G	-0.352	0.1777	0.0106	1.0E-200	35331	-0.019	0.8323	0.005	0.0002	2E+06	RET	decode	0.0302	1101.4
rs752978	T	C	0.151	0.4334	0.0083	1.3E-74	35330	-0.007	0.4065	0.004	0.0834	2E+06	RET	decode	0.0094	334.0
rs10918341	G	A	0.246	0.3030	0.0099	6.6E-137	35360	0.001	0.6705	0.004	0.8697	2E+06	FCGR3A	decode	0.0172	620.2
rs4657041	C	T	-0.452	0.4447	0.0050	1.0E-200	35351	-0.001	0.5118	0.004	0.6968	2E+06	FCGR3A	decode	0.1860	8075.9
rs7549876	T	G	-0.196	0.3998	0.0081	2.9E-129	35362	-0.009	0.3664	0.004	0.0198	2E+06	TIE1	decode	0.0163	585.1
rs757537	G	A	0.370	0.1266	0.0122	1.0E-200	35348	-0.026	0.1383	0.005	0.0000	2E+06	LEAP2	decode	0.0254	919.4
rs75776028	G	A	-0.402	0.3042	0.0067	1.0E-200	35362	-0.005	0.2642	0.004	0.2583	2E+06	SELL	decode	0.0918	3575.2
rs75881014	A	G	-0.606	0.0135	0.0343	5.9E-70	35348	-0.044	0.0123	0.017	0.0114	2E+06	CA1	decode	0.0088	312.6
rs75904281	T	C	-0.391	0.0141	0.0346	1.5E-29	35368	0.014	0.0149	0.017	0.3944	2E+06	RSPO4	decode	0.0036	127.5
rs76038906	T	G	0.551	0.0328	0.0236	8.8E-121	35362	0.019	0.0344	0.011	0.0729	2E+06	SVEP1	decode	0.0152	546.1
rs78742138	C	T	0.710	0.0362	0.0223	1.0E-200	35358	0.048	0.0296	0.011	0.0000	2E+06	SVEP1	decode	0.0280	1018.7
rs7607734	G	A	-0.428	0.1455	0.0095	1.0E-200	35363	0.001	0.1141	0.006	0.8374	2E+06	KYNU	decode	0.0543	2030.9
rs76143353	T	C	-0.653	0.0469	0.0187	1.0E-200	35371	0.008	0.0559	0.008	0.3124	2E+06	POFUT1	decode	0.0332	1212.8
rs76204064	T	C	0.469	0.0827	0.0146	1.0E-200	35368	-0.025	0.0658	0.008	0.0007	2E+06	FAM177A1	decode	0.0283	1029.7
rs4756649	C	T	-0.250	0.4058	0.0082	1.0E-200	35347	0.004	0.5874	0.004	0.2956	2E+06	KIAA1549L	decode	0.0255	923.2
rs762044	C	T	-0.127	0.4504	0.0082	3.5E-54	35357	0.004	0.4347	0.004	0.3522	2E+06	KIAA1549L	decode	0.0067	240.2
rs7637064	C	T	-0.106	0.2361	0.0096	1.6E-28	35367	0.002	0.2286	0.004	0.7406	2E+06	RTP4	decode	0.0035	122.8
rs9865045	C	T	-0.265	0.4928	0.0080	1.0E-200	35366	-0.010	0.5026	0.004	0.0104	2E+06	RTP4	decode	0.0301	1096.1
rs7674623	T	C	0.221	0.2132	0.0104	1.3E-100	35355	0.004	0.1941	0.005	0.3829	2E+06	ANTXR2	decode	0.0127	453.4
rs76790102	T	C	-0.550	0.0911	0.0115	1.0E-200	35359	0.004	0.0801	0.007	0.5727	2E+06	LGALS3	decode	0.0607	2286.3
rs76857490	A	G	-0.526	0.0256	0.0261	3.5E-90	35363	0.013	0.0209	0.013	0.3129	2E+06	KLB	decode	0.0113	405.5
rs76985127	G	T	0.201	0.0177	0.0306	4.7E-11	35362	-0.018	0.0197	0.014	0.2083	2E+06	TEK	decode	0.0012	43.3
rs77688991	C	T	0.305	0.0110	0.0404	4.8E-14	35371	0.005	0.0307	0.012	0.6862	2E+06	TEK	decode	0.0016	56.8
rs77157727	A	G	-0.435	0.0430	0.0195	3.6E-110	35368	-0.008	0.032	0.011	0.4793	2E+06	SERPINB13	decode	0.0139	497.3
rs7730934	A	G	0.369	0.1236	0.0126	1.4E-188	35361	-0.002	0.1196	0.006	0.6837	2E+06	IL6ST	decode	0.0237	857.9

rs7739450	A	G	-0.457	0.4652	0.0065	1.0E-200	35363	-0.006	0.4781	0.004	0.1228	2E+06	VEGFA	decode	0.1225	4937.2
rs77444140	A	G	-0.517	0.0238	0.0264	9.1E-86	35357	0.001	0.0175	0.015	0.9527	2E+06	C6	decode	0.0108	385.2
rs77515250	C	A	0.895	0.0139	0.0342	3.9E-151	35360	-0.005	0.0235	0.014	0.7055	2E+06	FUT8	decode	0.0190	685.7
rs4752926	T	C	-0.297	0.3440	0.0086	1.0E-200	35376	-0.008	0.6238	0.004	0.0400	2E+06	LRP4	decode	0.0328	1198.9
rs77682558	A	G	0.279	0.0469	0.0193	3.1E-47	35378	-0.003	0.0309	0.012	0.8229	2E+06	LRP4	decode	0.0059	208.3
rs10183939	A	G	-0.643	0.1865	0.0079	1.0E-200	35333	0.010	0.7771	0.005	0.0380	2E+06	COLEC11	decode	0.1591	6683.8
rs11123637	C	T	-0.201	0.3438	0.0095	2.8E-99	35333	0.011	0.6408	0.004	0.0073	2E+06	COLEC11	decode	0.0125	447.3
rs78111814	C	T	0.326	0.0581	0.0171	7.3E-81	35363	0.003	0.0494	0.009	0.7090	2E+06	DSCAM	decode	0.0102	362.7
rs7811214	A	G	-0.266	0.3455	0.0087	1.0E-200	35358	0.003	0.3395	0.004	0.4553	2E+06	TPST1	decode	0.0258	936.1
rs10905252	A	G	0.108	0.4107	0.0085	4.0E-37	35363	0.003	0.6093	0.004	0.4770	2E+06	ITIH2	decode	0.0046	162.1
rs78189039	A	G	0.476	0.0823	0.0152	1.0E-200	35358	-0.005	0.0855	0.007	0.4515	2E+06	ITIH2	decode	0.0271	984.8
rs10903015	A	C	-0.227	0.4748	0.0087	9.7E-149	35318	-0.009	0.5271	0.004	0.0145	2E+06	HBZ	decode	0.0187	674.6
rs78369087	A	G	-0.301	0.0227	0.0292	6.2E-25	35317	-0.035	0.0425	0.01	0.0003	2E+06	HBZ	decode	0.0030	106.3
rs78423067	A	G	-0.664	0.0346	0.0225	2.3E-192	35368	-0.014	0.0182	0.015	0.3575	2E+06	IL1R2	decode	0.0242	875.3
rs11103373	T	C	-0.283	0.4510	0.0087	1.0E-200	35266	0.003	0.5189	0.004	0.4394	2E+06	QSOX2	decode	0.0291	1055.4
rs7849585	T	G	-0.091	0.3274	0.0093	2.6E-22	35269	0.003	0.3279	0.004	0.4979	2E+06	QSOX2	decode	0.0027	94.4
rs7857240	G	A	0.045	0.4007	0.0082	4.9E-08	35348	-0.018	0.4603	0.004	0.0000	2E+06	FBP1	decode	0.0008	29.8
rs4818855	T	C	-0.128	0.4189	0.0081	6.1E-56	35267	0.013	0.5592	0.004	0.0008	2E+06	PDXK	decode	0.0070	248.2
rs79039769	A	G	-0.300	0.0154	0.0328	6.7E-20	35257	-0.005	0.019	0.015	0.7483	2E+06	PDXK	decode	0.0024	83.4
rs7911226	G	A	-0.236	0.2845	0.0090	1.0E-152	35356	0.004	0.315	0.004	0.3618	2E+06	FAS	decode	0.0192	692.9
rs79609374	G	A	-0.103	0.2848	0.0091	1.6E-29	35365	-0.010	0.3249	0.004	0.0138	2E+06	AMIGO2	decode	0.0036	127.3
rs4609582	G	A	0.305	0.2168	0.0099	1.0E-200	35381	0.000	0.7039	0.004	0.9298	2E+06	SAA1	decode	0.0262	951.0
rs79669707	A	G	0.352	0.1644	0.0109	1.0E-200	35382	-0.003	0.1321	0.006	0.6303	2E+06	SAA1	decode	0.0284	1033.0
rs1683696	T	G	0.105	0.4800	0.0085	5.0E-35	35377	-0.002	0.5021	0.004	0.5564	2E+06	MEM132	decode	0.0043	152.5
rs7973997	T	C	-0.255	0.3279	0.0090	9.4E-176	35381	0.007	0.3566	0.004	0.0818	2E+06	MEM132	decode	0.0221	798.9
rs79744555	C	T	0.494	0.1241	0.0098	1.0E-200	35358	-0.024	0.0842	0.007	0.0004	2E+06	NPTX1	decode	0.0666	2520.7
rs4572299	G	A	-0.335	0.2859	0.0098	1.0E-200	35340	0.006	0.7026	0.004	0.1828	2E+06	RNASE6	decode	0.0321	1173.0
rs80155227	A	G	-0.750	0.0314	0.0239	1.0E-200	35369	-0.015	0.0293	0.012	0.2139	2E+06	NT5C	decode	0.0270	981.2
rs80238657	G	A	-0.862	0.0245	0.0303	6.7E-178	34872	0.003	0.0245	0.012	0.8027	2E+06	F7	decode	0.0227	808.7
rs80241012	A	G	-0.349	0.1438	0.0137	7.8E-143	35356	-0.001	0.1373	0.006	0.8693	2E+06	CHI3L1	decode	0.0180	647.4
rs486890	G	A	0.349	0.4031	0.0048	1.0E-200	35376	-0.001	0.6541	0.004	0.7862	2E+06	CLEC12A	decode	0.1293	5254.4
rs80338457	G	A	-0.595	0.0356	0.0226	2.6E-153	35378	-0.022	0.02	0.014	0.1225	2E+06	CLEC12A	decode	0.0193	695.6

rs8034057	A	G	-0.339	0.0987	0.0139	1.3E-131	35363	0.003	0.0943	0.006	0.6738	2E+06	IGDCC4	decode	0.0166	595.9
rs8177107	G	A	-0.268	0.1752	0.0108	4.1E-135	35374	0.001	0.1778	0.005	0.9106	2E+06	EPHB6	decode	0.0170	612.0
rs8177399	T	C	-0.565	0.0158	0.0315	5.3E-72	35376	-0.011	0.0242	0.012	0.3538	2E+06	TIRAP	decode	0.0090	322.0
rs8178290	A	C	-0.288	0.2625	0.0095	1.0E-200	35369	-0.004	0.2367	0.004	0.3508	2E+06	LPO	decode	0.0253	919.5
rs8178414	T	C	-0.531	0.0174	0.0315	8.0E-64	35369	-0.008	0.0195	0.015	0.5675	2E+06	MPO	decode	0.0080	284.4
rs10836121	T	C	-0.071	0.4832	0.0081	1.6E-18	35357	-0.003	0.5236	0.004	0.3720	2E+06	CD59	decode	0.0022	77.1
rs831630	T	C	-0.183	0.3266	0.0086	5.1E-101	35356	-0.001	0.3206	0.004	0.7961	2E+06	CD59	decode	0.0127	455.3
rs835344	A	G	0.277	0.3868	0.0084	1.0E-200	35368	-0.009	0.4407	0.004	0.0125	2E+06	GPX7	decode	0.0298	1085.1
rs844124	T	C	0.128	0.3198	0.0086	6.1E-50	35367	0.013	0.3088	0.004	0.0015	2E+06	PCBD1	decode	0.0062	220.8
rs883138	C	A	0.249	0.2606	0.0093	1.9E-159	35371	0.002	0.262	0.004	0.6249	2E+06	RARRES2	decode	0.0201	723.9
rs9332575	C	T	-0.386	0.1271	0.0122	1.0E-200	35353	0.002	0.1121	0.006	0.6871	2E+06	SELP	decode	0.0274	996.8
rs9462450	C	T	-0.162	0.0873	0.0145	7.4E-29	35363	-0.015	0.112	0.006	0.0128	2E+06	GLO1	decode	0.0035	124.3
rs9488842	C	A	-0.251	0.2672	0.0088	3.9E-180	35354	0.004	0.2614	0.004	0.3454	2E+06	COL10A1	decode	0.0226	819.1
rs9532075	T	G	0.179	0.3834	0.0084	9.4E-101	35351	-0.005	0.3678	0.004	0.2324	2E+06	POSTN	decode	0.0127	454.0
rs9594222	T	C	0.166	0.1158	0.0130	2.4E-37	35351	0.008	0.1139	0.006	0.2054	2E+06	POSTN	decode	0.0046	163.1
rs9547175	T	C	-0.205	0.2146	0.0097	1.2E-98	35346	0.007	0.2248	0.005	0.1631	2E+06	SLITRK6	decode	0.0124	444.3
rs9811674	A	G	-0.526	0.1137	0.0089	1.0E-200	35375	0.004	0.8946	0.006	0.5699	2E+06	IL1RAP	decode	0.0891	3459.7
rs9860775	T	C	0.399	0.0657	0.0161	1.7E-135	35362	-0.009	0.0656	0.007	0.2214	2E+06	POGLUT1	decode	0.0171	613.8
rs9862503	G	A	0.260	0.3272	0.0084	1.0E-200	35357	-0.013	0.355	0.004	0.0006	2E+06	IL17RD	decode	0.0262	952.5
rs9890200	C	A	-0.320	0.4096	0.0071	1.0E-200	35353	-0.017	0.3616	0.004	0.0000	2E+06	SPATA20	decode	0.0542	2027.2
rs9900613	T	C	-0.271	0.4057	0.0081	1.0E-200	35382	0.002	0.4242	0.004	0.5522	2E+06	MXRA7	decode	0.0309	1127.3
rs995946	T	C	0.166	0.0573	0.0181	5.9E-20	35359	-0.007	0.0465	0.009	0.4111	2E+06	PLA2G7	decode	0.0024	83.6
rs1485803	A	G	-0.090	0.4019	0.0083	2.2E-27	35361	-0.008	0.5566	0.004	0.0248	2E+06	CHST9	decode	0.0033	117.5
rs9961915	T	C	0.260	0.3299	0.0086	1.0E-200	35361	-0.002	0.3141	0.004	0.5788	2E+06	CHST9	decode	0.0253	916.3
rs1023264	T	C	-0.145	0.2583	0.0094	7.2E-54	35323	-0.001	0.6759	0.004	0.8105	2E+06	CXCL12	decode	0.0067	238.8
rs10793518	G	A	0.067	0.2233	0.0099	1.5E-11	35323	0.004	0.816	0.005	0.4126	2E+06	CXCL12	decode	0.0013	45.5
rs1031707	C	T	0.302	0.2777	0.0090	1.0E-200	35354	-0.003	0.7259	0.004	0.5504	2E+06	PPIC	decode	0.0307	1118.9
rs10435378	A	G	0.131	0.4402	0.0084	3.4E-55	35343	-0.001	0.5581	0.004	0.8440	2E+06	MET	decode	0.0069	244.9
rs1060442	G	A	-0.146	0.3534	0.0092	2.6E-56	35319	0.002	0.6146	0.004	0.6131	2E+06	PRTN3	decode	0.0070	250.0
rs10749451	T	C	-0.243	0.4463	0.0080	1.0E-200	35372	-0.015	0.5369	0.004	0.0001	2E+06	PLEKHA1	decode	0.0253	919.0
rs10752113	T	C	0.316	0.2378	0.0103	1.0E-200	35351	-0.007	0.7616	0.004	0.1238	2E+06	ITIH5	decode	0.0261	947.7
rs10766205	A	G	0.249	0.2753	0.0087	6.6E-179	35369	0.004	0.7374	0.004	0.2935	2E+06	CALCB	decode	0.0225	813.3

rs10776914	C	T	-0.298	0.2999	0.0090	1.0E-200	35330	-0.004	0.6793	0.004	0.3092	2E+06	FCN1	decode	0.0300	1091.2
rs10894353	T	C	0.271	0.4665	0.0085	1.0E-200	35369	0.005	0.5528	0.004	0.2207	2E+06	NTM	decode	0.0279	1014.4
rs10901246	T	G	0.354	0.1934	0.0113	1.0E-200	35369	-0.010	0.7725	0.005	0.0299	2E+06	OBP2B	decode	0.0269	977.4
rs11156734	T	C	-0.284	0.2630	0.0093	1.0E-200	35337	0.007	0.7404	0.004	0.1012	2E+06	RNASE3	decode	0.0256	926.9
rs1130866	A	G	-0.395	0.4792	0.0067	1.0E-200	35332	0.000	0.5238	0.004	0.9569	2E+06	SFTPB	decode	0.0901	3498.5
rs11664471	C	A	-0.062	0.3911	0.0085	4.4E-13	35339	0.002	0.5795	0.004	0.6326	2E+06	DSC2	decode	0.0015	52.5
rs1658125	G	A	-0.208	0.1659	0.0110	3.7E-80	35339	-0.002	0.8413	0.005	0.6571	2E+06	DSC2	decode	0.0101	359.4
rs12493131	G	T	0.453	0.0747	0.0151	2.5E-197	35351	-0.002	0.8904	0.006	0.7420	2E+06	FAM3D	decode	0.0248	898.1
rs1250258	T	C	0.159	0.3267	0.0082	5.3E-83	35355	-0.007	0.741	0.004	0.0965	2E+06	FN1	decode	0.0104	372.5
rs12897030	T	C	0.219	0.2965	0.0090	7.2E-131	35332	0.000	0.685	0.004	0.9281	2E+06	RNASE1	decode	0.0165	592.5
rs151064	G	T	0.539	0.1248	0.0103	1.0E-200	35366	-0.006	0.8434	0.005	0.2249	2E+06	ADAMTS5	decode	0.0713	2714.6
rs1884080	G	T	-0.165	0.3070	0.0092	2.5E-72	35372	0.003	0.7112	0.004	0.5310	2E+06	SERPINA4	decode	0.0091	323.4
rs1935451	A	G	-0.296	0.1103	0.0130	2.3E-114	35365	-0.012	0.9098	0.007	0.0613	2E+06	UROS	decode	0.0144	516.5
rs1958078	C	A	0.246	0.1328	0.0120	5.9E-93	35348	0.002	0.8464	0.005	0.6758	2E+06	SMOC1	decode	0.0117	418.2
rs2085765	G	A	-0.151	0.1310	0.0124	7.7E-34	35355	-0.005	0.8812	0.006	0.3456	2E+06	SCARA5	decode	0.0041	147.0
rs2144693	C	T	0.250	0.3686	0.0082	1.0E-200	35365	0.012	0.6272	0.004	0.0014	2E+06	GSTA1	decode	0.0254	923.2
rs2229498	A	G	0.223	0.1651	0.0108	1.7E-94	35362	0.004	0.8423	0.005	0.4936	2E+06	SRGN	decode	0.0119	425.3
rs2465403	A	G	0.150	0.4470	0.0082	2.8E-74	35359	-0.010	0.6072	0.004	0.0092	2E+06	TNFRSF11[illegible]	decode	0.0093	332.4
rs2586528	T	G	-0.283	0.3194	0.0086	1.0E-200	35379	-0.013	0.7072	0.004	0.0014	2E+06	ENO3	decode	0.0299	1089.9
rs2766575	C	T	0.334	0.4102	0.0067	1.0E-200	35354	0.012	0.5898	0.004	0.0021	2E+06	CLPS	decode	0.0662	2508.1
rs2842970	T	C	-0.263	0.4322	0.0081	1.0E-200	35389	-0.002	0.5783	0.004	0.5409	2E+06	ACAT2	decode	0.0288	1048.3
rs2846703	A	G	-0.142	0.2696	0.0097	4.0E-49	35365	0.004	0.7107	0.004	0.3700	2E+06	MMP1	decode	0.0061	217.0
rs36086195	T	C	-0.137	0.4300	0.0083	1.3E-60	35386	0.004	0.5917	0.004	0.2799	2E+06	EPHA2	decode	0.0076	269.7
rs3800403	G	A	0.273	0.1380	0.0116	4.0E-122	35353	-0.003	0.8477	0.005	0.6094	2E+06	SCUBE3	decode	0.0154	552.2
rs4073961	A	G	0.065	0.4923	0.0081	1.1E-15	35013	-0.003	0.5211	0.004	0.4471	2E+06	SECTM1	decode	0.0018	64.3
rs4789763	G	A	0.185	0.4804	0.0081	1.0E-114	35014	0.004	0.4961	0.004	0.2716	2E+06	SECTM1	decode	0.0146	518.2
rs4346716	C	T	-0.360	0.4328	0.0068	1.0E-200	35372	-0.001	0.5313	0.004	0.7304	2E+06	MAN2B2	decode	0.0738	2817.2
rs4394214	T	G	0.209	0.1799	0.0108	4.1E-83	35375	0.007	0.7918	0.005	0.1345	2E+06	TREM1	decode	0.0104	373.1
rs6914090	C	T	0.277	0.4918	0.0082	1.0E-200	35372	0.004	0.4824	0.004	0.2831	2E+06	TREM1	decode	0.0312	1137.7
rs4434842	G	T	0.505	0.0739	0.0163	1.0E-200	35351	0.007	0.9415	0.008	0.4063	2E+06	AMY1A	decode	0.0265	962.8
rs4457570	G	A	0.286	0.4251	0.0083	1.0E-200	35347	-0.006	0.6216	0.004	0.1599	2E+06	CRABP2	decode	0.0323	1179.4
rs4665681	A	G	0.477	0.0187	0.0297	5.6E-58	35369	0.018	0.9719	0.012	0.1328	2E+06	TP53I3	decode	0.0072	257.6

rs4679138	A	G	0.254	0.3037	0.0088	2.9E-182	35369	-0.001	0.6887	0.004	0.8155	2E+06	PLXNA1	decode	0.0229	828.8
rs4704826	A	C	-0.242	0.3579	0.0087	1.0E-168	35351	-0.003	0.6331	0.004	0.4076	2E+06	TIMD4	decode	0.0212	766.4
rs470530	G	A	0.597	0.0989	0.0110	1.0E-200	35364	-0.013	0.8461	0.005	0.0127	2E+06	MMP12	decode	0.0763	2919.7
rs4733505	G	A	0.113	0.3390	0.0086	1.3E-39	35358	0.006	0.6673	0.004	0.1727	2E+06	GSR	decode	0.0049	173.5
rs4767461	T	C	0.289	0.2539	0.0094	1.0E-200	35362	0.001	0.7445	0.004	0.7766	2E+06	TESC	decode	0.0262	950.3
rs4783718	C	T	0.276	0.3905	0.0079	1.0E-200	35375	-0.035	0.5991	0.004	0.0000	2E+06	NQO1	decode	0.0332	1213.0
rs4859610	G	A	-0.273	0.2207	0.0099	2.9E-166	35355	-0.008	0.7687	0.004	0.0612	2E+06	ART3	decode	0.0209	755.2
rs5751764	C	A	-0.254	0.4461	0.0080	1.0E-200	35361	0.002	0.5876	0.004	0.6575	2E+06	GSTT2B	decode	0.0274	997.8
rs5756736	A	G	-0.207	0.4375	0.0080	3.3E-148	35365	-0.006	0.5885	0.004	0.1526	2E+06	LGALS2	decode	0.0187	672.2
rs6655975	G	A	-0.186	0.3969	0.0082	2.4E-113	35357	0.015	0.6513	0.004	0.0001	2E+06	CTSS	decode	0.0143	511.8
rs6663887	G	A	0.146	0.2630	0.0097	9.1E-51	35370	-0.003	0.7448	0.004	0.4918	2E+06	CR1	decode	0.0063	224.5
rs6892697	A	G	-0.262	0.3924	0.0084	1.0E-200	35354	0.010	0.6541	0.004	0.0124	2E+06	TGFBI	decode	0.0268	972.0
rs6906620	C	A	-0.360	0.2802	0.0061	1.0E-200	35357	0.000	0.7551	0.004	0.9563	2E+06	ENPP5	decode	0.0902	3504.8
rs6920109	C	T	-0.141	0.2388	0.0097	7.7E-48	35356	-0.003	0.7509	0.004	0.5242	2E+06	UST	decode	0.0059	211.1
rs7032104	T	C	-0.275	0.4588	0.0082	1.0E-200	35257	0.007	0.5497	0.004	0.0760	2E+06	C8G	decode	0.0308	1122.2
rs7647776	A	C	0.356	0.2937	0.0076	1.0E-200	35367	0.006	0.7191	0.004	0.1651	2E+06	AHSG	decode	0.0588	2209.7
rs7667751	T	C	0.273	0.3714	0.0084	1.0E-200	35364	0.004	0.6864	0.004	0.3854	2E+06	UGDH	decode	0.0288	1048.4
rs7970720	T	G	0.336	0.1753	0.0107	1.0E-200	35390	0.005	0.8174	0.005	0.3402	2E+06	C1R	decode	0.0273	992.1
rs809066	G	A	0.532	0.1079	0.0106	1.0E-200	35347	-0.003	0.9063	0.007	0.6792	2E+06	CROT	decode	0.0660	2498.9
rs840870	T	G	-0.153	0.4317	0.0082	1.9E-78	35361	-0.004	0.5881	0.004	0.3165	2E+06	HS6ST1	decode	0.0098	351.5
rs854784	T	C	0.324	0.4433	0.0060	1.0E-200	35347	-0.014	0.5451	0.004	0.0001	2E+06	SHMT1	decode	0.0756	2890.5
rs858523	T	C	-0.201	0.4304	0.0082	1.6E-133	35370	-0.009	0.5585	0.004	0.0174	2E+06	SAT2	decode	0.0168	604.6
rs9384738	C	T	0.161	0.0820	0.0143	1.1E-29	35372	0.004	0.908	0.007	0.5146	2E+06	METTL24	decode	0.0036	128.0
rs10031141	G	A	0.487	0.4036	0.0159	3.9E-193	7213	0.001	0.3993	0.004	0.7515	2E+06	CXCL6	aric	0.1147	934.4
rs10069277	G	A	-0.225	0.0713	0.0320	2.3E-12	7213	0.000	0.0706	0.007	0.9726	2E+06	THBS4	aric	0.0068	49.4
rs13167730	T	G	0.655	0.0934	0.0277	5.7E-119	7213	-0.003	0.0914	0.006	0.6388	2E+06	THBS4	aric	0.0719	558.2
rs2404710	A	C	0.212	0.4337	0.0166	5.6E-37	7213	0.010	0.4277	0.004	0.0060	2E+06	THBS4	aric	0.0221	163.2
rs10075736	T	C	0.506	0.1560	0.0226	9.0E-108	7213	-0.002	0.1522	0.005	0.7602	2E+06	ERAP1	aric	0.0652	503.0
rs76200405	T	C	0.816	0.0297	0.0476	1.2E-64	7213	0.008	0.0344	0.01	0.4360	2E+06	ERAP1	aric	0.0392	294.0
rs10148939	C	A	-0.394	0.1379	0.0236	1.3E-61	7213	0.018	0.1444	0.005	0.0008	2E+06	NID2	aric	0.0373	279.6
rs754423	T	C	-0.265	0.2340	0.0195	1.6E-41	7213	-0.008	0.234	0.004	0.0570	2E+06	NID2	aric	0.0249	184.5
rs10231308	C	T	0.247	0.2543	0.0189	2.2E-38	7213	0.004	0.2421	0.004	0.3087	2E+06	HSPB1	aric	0.0230	169.8

rs13240755	G	A	-0.475	0.3474	0.0165	6.6E-172	7213	-0.012	0.3396	0.004	0.0021	2E+06	HSPB1	aric	0.1027	824.9
rs1036491	C	A	-0.175	0.2025	0.0208	4.6E-17	7213	0.003	0.2127	0.005	0.5720	2E+06	CBLN4	aric	0.0097	70.8
rs6024417	G	A	-0.199	0.3033	0.0181	3.9E-28	7213	-0.008	0.2997	0.004	0.0587	2E+06	CBLN4	aric	0.0166	122.0
rs7351418	T	C	0.224	0.3366	0.0175	4.4E-37	7213	-0.010	0.3232	0.004	0.0117	2E+06	CBLN4	aric	0.0222	163.7
rs5851	G	A	-0.129	0.4411	0.0168	2.1E-14	7213	-0.008	0.4511	0.004	0.0452	2E+06	ACP5	aric	0.0081	58.6
rs1040402	A	G	-0.343	0.1936	0.0207	2.2E-60	7213	0.002	0.1953	0.005	0.7363	2E+06	FBLN1	aric	0.0366	273.7
rs136743	C	A	0.140	0.4537	0.0166	3.5E-17	7213	-0.006	0.4675	0.004	0.1278	2E+06	FBLN1	aric	0.0098	71.4
rs12981737	T	C	-0.300	0.0279	0.0503	2.5E-09	7213	-0.013	0.0294	0.012	0.2898	2E+06	GP6	aric	0.0049	35.6
rs1043784	C	T	-0.273	0.1097	0.0264	6.2E-25	7213	-0.010	0.1192	0.006	0.0766	2E+06	TXNDC5	aric	0.0146	107.1
rs10467359	G	A	-0.328	0.0491	0.0384	1.5E-17	7213	0.016	0.0546	0.008	0.0457	2E+06	KL	aric	0.0100	73.1
rs10501089	T	C	0.375	0.0392	0.0427	2.0E-18	7213	0.007	0.0543	0.009	0.4203	2E+06	BDNF	aric	0.0106	77.1
rs10506379	A	G	-0.213	0.0557	0.0364	5.6E-09	7213	0.010	0.0578	0.008	0.2141	2E+06	LRIG3	aric	0.0047	34.0
rs6581219	G	A	-0.159	0.4271	0.0170	1.2E-20	7213	0.005	0.4218	0.004	0.1539	2E+06	LRIG3	aric	0.0120	87.2
rs10512462	G	A	-0.256	0.0787	0.0306	7.5E-17	7213	-0.002	0.0751	0.007	0.7839	2E+06	CCL8	aric	0.0096	69.9
rs10853162	A	G	0.326	0.3461	0.0171	6.4E-79	7213	0.005	0.3488	0.004	0.2189	2E+06	CCL8	aric	0.0479	362.5
rs1059367	T	C	-0.172	0.0754	0.0313	4.2E-08	7213	0.023	0.0853	0.007	0.0008	2E+06	HNRNPAB	aric	0.0042	30.1
rs10739734	G	T	-0.171	0.3850	0.0170	1.2E-23	7213	-0.001	0.3757	0.004	0.8284	2E+06	SH3GLB2	aric	0.0138	101.1
rs10814111	A	C	0.529	0.0653	0.0331	1.9E-56	7213	0.011	0.0636	0.008	0.1512	2E+06	NUDT2	aric	0.0341	254.9
rs7025269	C	T	-0.894	0.2295	0.0169	0.0E+00	7213	0.020	0.2221	0.004	0.0000	2E+06	NUDT2	aric	0.2793	2794.2
rs10817700	G	A	-0.299	0.3503	0.0170	1.8E-67	7213	0.006	0.3382	0.004	0.1018	2E+06	TNC	aric	0.0409	307.5
rs10820918	G	A	0.341	0.3271	0.0174	2.6E-83	7213	-0.004	0.3189	0.004	0.3648	2E+06	ROR2	aric	0.0505	383.7
rs10849454	C	T	-0.270	0.2797	0.0182	4.0E-49	7213	0.003	0.2877	0.004	0.4743	2E+06	TAPBPL	aric	0.0296	220.3
s11724448[illegible]	C	T	-0.521	0.0135	0.0719	5.1E-13	7213	-0.026	0.0219	0.014	0.0720	2E+06	TAPBPL	aric	0.0072	52.4
rs56983450	A	G	0.252	0.0340	0.0456	3.4E-08	7213	0.004	0.0391	0.01	0.7217	2E+06	TAPBPL	aric	0.0042	30.5
rs10883974	C	T	-0.477	0.2645	0.0181	5.4E-146	7213	-0.006	0.2585	0.004	0.1552	2E+06	GSTO1	aric	0.0877	693.2
rs805670	T	C	-0.268	0.4130	0.0166	1.2E-57	7213	-0.003	0.417	0.004	0.4451	2E+06	GSTO1	aric	0.0349	260.7
rs10889352	C	T	-0.335	0.3316	0.0171	1.2E-83	7213	-0.004	0.325	0.004	0.3133	2E+06	ANGPTL3	aric	0.0507	385.3
rs10920287	T	G	0.530	0.3108	0.0170	4.6E-201	7213	-0.008	0.3077	0.004	0.0676	2E+06	RNPEP	aric	0.1192	975.8
rs2678208	G	T	-0.412	0.0962	0.0278	4.8E-49	7213	0.018	0.0925	0.007	0.0095	2E+06	RNPEP	aric	0.0296	219.9
rs10941528	T	C	-0.411	0.2403	0.0188	3.5E-102	7213	-0.007	0.2385	0.004	0.1239	2E+06	C7	aric	0.0619	475.5
rs10972159	A	G	-0.996	0.0179	0.0613	2.5E-58	7213	-0.015	0.0286	0.013	0.2359	2E+06	CNTFR	aric	0.0353	263.9
rs2183012	A	G	0.385	0.0193	0.0607	2.4E-10	7213	-0.006	0.019	0.014	0.6598	2E+06	CNTFR	aric	0.0056	40.3

rs72735302	C	T	-0.358	0.0527	0.0368	3.3E-22	7213	-0.009	0.056	0.008	0.2733	2E+06	CNTFR	aric	0.0129	94.5
rs10973326	A	G	0.356	0.3178	0.0173	2.1E-91	7213	0.000	0.3362	0.004	0.9740	2E+06	GRHPR	aric	0.0554	423.0
rs10981890	G	A	-0.185	0.4707	0.0165	1.1E-28	7213	-0.001	0.466	0.004	0.8762	2E+06	CTSV	aric	0.0170	124.4
rs11002760	G	A	-0.302	0.0340	0.0458	4.4E-11	7213	0.013	0.0396	0.01	0.2182	2E+06	MBL2	aric	0.0060	43.6
rs7475006	T	C	0.265	0.4035	0.0167	1.2E-55	7213	0.002	0.4102	0.004	0.6057	2E+06	MBL2	aric	0.0337	251.1
rs10734190	T	C	-0.311	0.1556	0.0225	4.6E-43	7213	-0.003	0.1637	0.005	0.6188	2E+06	DKK3	aric	0.0259	191.7
rs11022114	A	G	0.477	0.3105	0.0170	4.9E-164	7213	0.000	0.3172	0.004	0.9752	2E+06	DKK3	aric	0.0981	784.7
rs11025480	A	G	0.138	0.2590	0.0189	4.0E-13	7213	-0.002	0.2523	0.004	0.6291	2E+06	HTATIP2	aric	0.0073	52.8
rs11055175	G	A	-1.608	0.0338	0.0419	2.0E-293	7213	0.030	0.0331	0.01	0.0045	2E+06	HEBP1	aric	0.1696	1472.6
rs11055473	T	C	-0.242	0.3103	0.0178	1.6E-41	7213	0.003	0.3018	0.004	0.4536	2E+06	CLEC4C	aric	0.0249	184.5
rs8035095	T	C	0.117	0.2441	0.0193	1.6E-09	7213	0.000	0.2346	0.004	0.9805	2E+06	RGMA	aric	0.0050	36.5
rs11078596	T	C	-0.350	0.1832	0.0213	1.3E-59	7213	0.004	0.1862	0.005	0.4581	2E+06	SERPINF2	aric	0.0361	270.1
rs11089474	T	G	0.247	0.2165	0.0201	2.1E-34	7213	-0.004	0.2048	0.005	0.4286	2E+06	TCN2	aric	0.0205	151.1
s112812772	A	G	0.631	0.0308	0.0473	4.1E-40	7213	0.013	0.0291	0.011	0.2315	2E+06	TCN2	aric	0.0241	177.9
rs2413004	G	A	-0.144	0.3606	0.0172	7.9E-17	7213	0.012	0.3531	0.004	0.0036	1E+06	TCN2	aric	0.0096	69.7
rs5997658	T	G	-0.174	0.4859	0.0166	2.0E-25	7213	0.004	0.489	0.004	0.2557	2E+06	TCN2	aric	0.0149	109.3
rs1109980	G	A	0.281	0.0961	0.0282	3.0E-23	7213	0.012	0.0973	0.006	0.0540	2E+06	GLCE	aric	0.0136	99.3
rs12708499	T	C	-0.477	0.2724	0.0179	4.1E-150	7213	0.005	0.286	0.004	0.2551	2E+06	GLCE	aric	0.0901	714.0
rs11103667	T	C	0.137	0.1933	0.0212	1.0E-10	7213	0.019	0.1879	0.005	0.0001	2E+06	OLFM1	aric	0.0058	41.9
rs11107427	A	G	0.546	0.0204	0.0583	9.8E-21	7213	-0.003	0.0235	0.012	0.8251	2E+06	PLXNC1	aric	0.0120	87.7
s115651556	A	G	-1.804	0.0129	0.0707	2.3E-137	7213	-0.006	0.0182	0.015	0.6834	2E+06	PLXNC1	aric	0.0827	649.9
rs11111878	A	C	0.642	0.1078	0.0257	1.8E-132	7213	0.007	0.1203	0.006	0.1976	2E+06	HSP90B1	aric	0.0798	625.3
rs1165593	G	A	0.808	0.1549	0.0212	7.8E-289	7213	0.007	0.1573	0.005	0.1814	2E+06	HSP90B1	aric	0.1671	1447.2
s111257030	T	C	-0.434	0.0950	0.0281	4.8E-53	7213	0.001	0.0964	0.006	0.8208	2E+06	CST4	aric	0.0321	238.8
s111284156	G	T	0.504	0.0427	0.0407	6.1E-35	7213	0.002	0.0438	0.009	0.8009	2E+06	TIMP3	aric	0.0209	153.7
rs3788507	A	G	-0.383	0.0916	0.0286	1.6E-40	7213	-0.003	0.0922	0.006	0.5933	2E+06	TIMP3	aric	0.0243	179.7
rs732446	G	T	0.357	0.2147	0.0199	2.4E-70	7213	-0.002	0.2094	0.005	0.7080	2E+06	TIMP3	aric	0.0427	321.3
rs10013945	A	G	0.543	0.2073	0.0195	5.6E-162	7213	-0.009	0.2202	0.005	0.0460	2E+06	IGFBP7	aric	0.0970	774.2
rs11133472	T	C	0.140	0.3381	0.0175	1.7E-15	7213	-0.005	0.3354	0.004	0.1795	2E+06	IGFBP7	aric	0.0087	63.6
s111486799	A	G	-0.366	0.1980	0.0204	2.0E-70	7213	0.010	0.2033	0.005	0.0363	2E+06	SH3BGRL3	aric	0.0427	321.6
rs6676630	T	C	-1.163	0.0708	0.0294	0.0E+00	7213	0.004	0.0728	0.007	0.5858	2E+06	SH3BGRL3	aric	0.1784	1565.3
rs11158538	T	C	-0.177	0.4450	0.0168	8.0E-26	7213	-0.003	0.4326	0.004	0.3635	2E+06	MTHFD1	aric	0.0152	111.2

s111587591	T	C	0.682	0.0399	0.0423	2.3E-57	7213	0.007	0.044	0.009	0.4387	2E+06	ADAMTS13	aric	0.0347	259.4
rs41302905	T	C	-0.541	0.0225	0.0564	1.1E-21	7213	-0.001	0.0222	0.013	0.9176	2E+06	ADAMTS13	aric	0.0126	92.0
s111613270	T	C	0.933	0.0155	0.0665	4.0E-44	7213	-0.001	0.0144	0.016	0.9308	2E+06	MIA	aric	0.0266	196.7
s184589612	C	T	0.851	0.0166	0.0646	3.7E-39	7213	-0.008	0.025	0.013	0.5565	2E+06	MIA	aric	0.0235	173.4
rs74746550	C	T	0.950	0.0482	0.0374	2.3E-136	7213	0.010	0.0482	0.009	0.2487	2E+06	MIA	aric	0.0821	644.9
s111725844	T	C	0.251	0.0432	0.0410	1.0E-09	7213	0.007	0.0517	0.009	0.4120	2E+06	ACE	aric	0.0052	37.3
s142377191	A	G	0.406	0.0192	0.0608	2.7E-11	7213	0.011	0.0224	0.014	0.4473	2E+06	ACE	aric	0.0061	44.5
s150241958	G	A	0.412	0.0189	0.0610	1.6E-11	7213	0.025	0.0178	0.015	0.0920	2E+06	ACE	aric	0.0063	45.5
rs4968748	A	G	-0.369	0.2167	0.0196	5.5E-77	7213	-0.009	0.2329	0.004	0.0426	2E+06	ACE	aric	0.0467	353.2
rs11186737	T	C	-0.600	0.3096	0.0166	2.3E-264	7213	0.019	0.3165	0.004	0.0000	1E+06	FGFBP3	aric	0.1540	1313.1
rs11197747	C	T	-0.396	0.1596	0.0223	2.7E-69	7213	-0.002	0.1554	0.005	0.7431	2E+06	PNLIPRP2	aric	0.0420	316.2
rs17735613	G	A	0.421	0.1504	0.0229	5.3E-74	7213	0.012	0.1484	0.005	0.0273	2E+06	PNLIPRP2	aric	0.0449	338.8
s112126489	A	G	0.343	0.0194	0.0595	8.8E-09	7213	0.006	0.0217	0.013	0.6494	2E+06	DPEP1	aric	0.0046	33.2
rs12918760	G	T	0.393	0.0182	0.0620	2.4E-10	7213	0.049	0.019	0.015	0.0008	2E+06	DPEP1	aric	0.0055	40.2
rs62067101	T	C	-0.499	0.0229	0.0553	2.4E-19	7213	0.004	0.0274	0.012	0.7256	2E+06	DPEP1	aric	0.0112	81.3
rs11214489	T	C	-0.360	0.1901	0.0208	8.2E-66	7213	0.014	0.205	0.005	0.0032	2E+06	NCAM1	aric	0.0399	299.6
rs2288158	G	T	0.460	0.1341	0.0240	4.0E-80	7213	-0.009	0.1358	0.006	0.0945	2E+06	NCAM1	aric	0.0486	368.3
rs61902388	C	T	-0.222	0.4871	0.0165	7.3E-41	7213	0.005	0.5018	0.004	0.1872	2E+06	NCAM1	aric	0.0245	181.4
rs11219769	T	G	-0.371	0.2656	0.0183	2.7E-89	7213	0.005	0.2562	0.004	0.2520	2E+06	ESAM	aric	0.0541	412.7
s112278379	G	T	-0.502	0.0198	0.0598	5.4E-17	7213	0.001	0.023	0.013	0.9239	2E+06	CD177	aric	0.0097	70.5
rs11229039	A	G	-0.561	0.1277	0.0242	7.9E-115	7213	-0.010	0.1293	0.006	0.0683	2E+06	SERPING1	aric	0.0694	537.8
s117960683	C	T	-0.417	0.0895	0.0288	1.0E-46	7213	-0.004	0.0921	0.007	0.5792	2E+06	SERPING1	aric	0.0282	209.0
s112437560	T	C	0.338	0.0186	0.0609	2.9E-08	7213	0.006	0.0184	0.014	0.6940	2E+06	IL1RL2	aric	0.0043	30.8
s112488423	G	A	0.608	0.0155	0.0675	2.7E-19	7213	-0.010	0.024	0.014	0.4909	2E+06	CFHR4	aric	0.0111	81.1
rs12131802	T	C	-0.235	0.0551	0.0365	1.3E-10	7213	-0.003	0.0543	0.008	0.6837	2E+06	FCRL3	aric	0.0057	41.3
s112735728	C	T	0.677	0.0312	0.0472	4.9E-46	7213	0.014	0.0323	0.011	0.1894	2E+06	SIGLEC12	aric	0.0277	205.7
rs79506121	C	T	0.658	0.0347	0.0450	8.9E-48	7213	-0.006	0.0355	0.01	0.5569	2E+06	SIGLEC12	aric	0.0288	213.9
s112885443	G	T	0.240	0.0596	0.0347	5.3E-12	7213	0.001	0.0567	0.008	0.8675	2E+06	SPINT2	aric	0.0066	47.7
rs6508781	C	A	-0.556	0.1541	0.0219	3.4E-136	7213	-0.003	0.1626	0.005	0.5170	2E+06	SPINT2	aric	0.0820	644.0
s112963922	T	C	-1.165	0.0164	0.0640	2.2E-72	7213	-0.052	0.0199	0.014	0.0003	2E+06	SERPINA1	aric	0.0439	331.0
s148597299	C	T	0.362	0.0196	0.0603	2.0E-09	7213	0.014	0.0244	0.013	0.2674	2E+06	SERPINA1	aric	0.0050	36.0
rs72702354	G	A	-0.799	0.0330	0.0457	5.4E-67	7213	0.007	0.0369	0.011	0.5266	2E+06	SERPINA1	aric	0.0406	305.3

s113098364	C	T	-0.320	0.1133	0.0259	9.5E-35	7213	-0.005	0.1247	0.006	0.4003	2E+06	PLA2R1	aric	0.0207	152.8
rs12471260	G	A	0.222	0.4016	0.0168	3.1E-39	7213	-0.003	0.416	0.004	0.3819	2E+06	PLA2R1	aric	0.0235	173.8
s113286331	T	C	0.558	0.0637	0.0337	1.5E-60	7213	0.009	0.0682	0.008	0.2202	2E+06	ENPP7	aric	0.0367	274.5
s113400434	A	G	0.682	0.0343	0.0452	8.4E-51	7213	0.009	0.0376	0.01	0.3482	2E+06	CTRB2	aric	0.0307	228.2
s113529953	T	C	0.485	0.0315	0.0471	1.3E-24	7213	-0.027	0.0293	0.012	0.0177	2E+06	CEL	aric	0.0144	105.7
rs1135816	G	A	-0.387	0.4127	0.0164	1.4E-118	7213	0.000	0.4223	0.004	0.9069	2E+06	KLRB1	aric	0.0716	556.4
s114648232	A	C	0.645	0.0316	0.0474	1.3E-41	7213	-0.014	0.0411	0.01	0.1547	2E+06	GMPR	aric	0.0250	184.9
s114787025	G	T	-0.421	0.0201	0.0591	1.2E-12	7213	0.000	0.0345	0.012	0.9901	2E+06	GMPR	aric	0.0070	50.7
s138143229	A	G	0.553	0.0112	0.0789	2.6E-12	7213	-0.033	0.0139	0.017	0.0479	2E+06	GMPR	aric	0.0068	49.1
s140659976	A	G	-0.362	0.0211	0.0579	4.2E-10	7213	0.004	0.0231	0.014	0.7986	2E+06	GMPR	aric	0.0054	39.1
s141485758	G	A	0.647	0.0172	0.0642	9.4E-24	7213	-0.002	0.022	0.014	0.8966	2E+06	GMPR	aric	0.0139	101.6
s113856318	C	T	0.863	0.0177	0.0624	6.6E-43	7213	-0.001	0.0185	0.014	0.9472	2E+06	PCOLCE2	aric	0.0258	191.0
rs35351308	A	G	0.364	0.0163	0.0656	2.9E-08	7213	0.004	0.0197	0.014	0.8086	2E+06	PCOLCE2	aric	0.0043	30.8
rs9829564	C	T	-0.474	0.1627	0.0221	1.7E-99	7213	-0.006	0.1719	0.005	0.2484	2E+06	PCOLCE2	aric	0.0603	462.4
rs11103545	G	T	0.312	0.2799	0.0181	6.3E-65	7213	-0.007	0.2825	0.004	0.1291	2E+06	FCN2	aric	0.0393	295.3
s113909851	A	G	0.216	0.0713	0.0320	1.4E-11	7213	-0.002	0.0709	0.007	0.8333	2E+06	FCN2	aric	0.0063	45.8
s113953523	A	G	-0.667	0.0155	0.0668	2.3E-23	7213	-0.015	0.0156	0.016	0.3340	2E+06	INHBC	aric	0.0137	99.8
s113958256	A	G	0.505	0.0701	0.0320	4.4E-55	7213	0.002	0.0699	0.007	0.8197	2E+06	SERPINA12	aric	0.0333	248.5
rs55911632	G	A	-0.132	0.1592	0.0228	7.8E-09	7213	-0.003	0.1645	0.005	0.5399	2E+06	SERPINA12	aric	0.0046	33.4
s113968521	C	T	0.270	0.1314	0.0247	1.3E-27	7213	-0.002	0.133	0.006	0.7555	2E+06	GOLM1	aric	0.0163	119.5
rs7868110	A	G	0.465	0.1110	0.0261	1.5E-69	7213	-0.003	0.1068	0.006	0.6045	2E+06	GOLM1	aric	0.0422	317.4
s113979133	T	C	-1.097	0.0229	0.0545	7.8E-88	7213	-0.019	0.0194	0.014	0.1575	2E+06	CRISP2	aric	0.0533	405.6
s114022440	G	A	0.416	0.0328	0.0463	3.2E-19	7213	0.012	0.0348	0.011	0.2702	2E+06	EPHA4	aric	0.0111	80.7
rs4674589	C	T	0.186	0.4540	0.0165	3.2E-29	7213	-0.001	0.4548	0.004	0.7902	2E+06	EPHA4	aric	0.0173	127.0
s114099765	C	T	0.468	0.0259	0.0520	2.9E-19	7213	-0.001	0.0295	0.011	0.9239	2E+06	MYOC	aric	0.0111	80.9
rs7535044	A	G	0.598	0.0150	0.0684	2.8E-18	7213	0.016	0.0175	0.014	0.2734	2E+06	MYOC	aric	0.0105	76.4
s114116433	T	C	1.284	0.0182	0.0597	2.1E-99	7213	-0.004	0.0201	0.015	0.7912	2E+06	CLSTN1	aric	0.0602	461.9
s114513597	A	G	-0.693	0.0158	0.0665	3.0E-25	7213	0.002	0.0175	0.015	0.9019	2E+06	CLSTN1	aric	0.0148	108.5
s114162507	C	T	-0.438	0.0146	0.0687	1.9E-10	7213	0.004	0.0144	0.016	0.8004	2E+06	AMY2A	aric	0.0056	40.7
s114286445	A	G	-0.955	0.0156	0.0662	1.5E-46	7213	-0.017	0.0158	0.016	0.2679	2E+06	CRELD1	aric	0.0281	208.2
s144363827	A	G	0.594	0.0258	0.0517	3.2E-30	7213	0.011	0.023	0.013	0.4296	2E+06	CRELD1	aric	0.0179	131.7
rs17537486	G	A	0.503	0.0290	0.0492	2.3E-24	7213	0.002	0.0278	0.012	0.8750	2E+06	CRELD1	aric	0.0143	104.5

rs34684179	A	G	-0.752	0.0271	0.0499	1.4E-50	7213	0.012	0.026	0.012	0.3154	2E+06	CRELD1	aric	0.0305	227.2
s114395538	A	G	-0.465	0.0173	0.0636	2.9E-13	7213	-0.002	0.0175	0.015	0.8874	2E+06	TSTD1	aric	0.0074	53.4
rs11580071	A	G	0.896	0.1253	0.0228	4.4E-307	7213	0.006	0.1288	0.006	0.2758	2E+06	TSTD1	aric	0.1768	1548.6
s114425483	T	C	0.465	0.0394	0.0421	4.5E-28	7213	0.007	0.0487	0.009	0.4435	2E+06	CNTNAP2	aric	0.0166	121.7
s114472213	T	C	0.409	0.0381	0.0431	3.0E-21	7213	-0.008	0.0359	0.01	0.4341	2E+06	C9	aric	0.0123	90.1
s115096222	T	C	0.436	0.0119	0.0758	9.2E-09	7213	-0.010	0.0194	0.015	0.5164	2E+06	C9	aric	0.0046	33.1
s114500706	G	A	-0.533	0.0470	0.0391	1.2E-41	7213	-0.006	0.0491	0.009	0.4898	2E+06	AMY2B	aric	0.0250	185.1
rs72861085	G	A	-0.417	0.0166	0.0653	1.9E-10	7213	0.000	0.0245	0.014	0.9896	2E+06	LYVE1	aric	0.0056	40.7
rs76318574	C	T	0.349	0.0304	0.0474	2.0E-13	7213	0.029	0.0277	0.012	0.0120	2E+06	LYVE1	aric	0.0075	54.2
s114928388	A	G	0.335	0.0234	0.0553	1.4E-09	7213	0.007	0.0267	0.012	0.5668	2E+06	CD14	aric	0.0051	36.7
rs5744454	G	T	-0.299	0.2437	0.0188	2.7E-56	7213	-0.012	0.2492	0.004	0.0066	2E+06	CD14	aric	0.0341	254.2
s114965972	A	G	0.423	0.0134	0.0718	3.9E-09	7213	0.000	0.0134	0.017	0.9971	2E+06	ST3GAL6	aric	0.0048	34.7
rs74890720	C	T	0.758	0.0534	0.0359	6.5E-96	7213	-0.002	0.0519	0.009	0.8345	2E+06	ST3GAL6	aric	0.0581	444.9
s114986549	A	G	0.326	0.0516	0.0370	1.5E-18	7213	-0.012	0.0571	0.008	0.1512	2E+06	PLA2G2A	aric	0.0107	77.7
rs2063054	T	G	0.244	0.4407	0.0166	3.6E-48	7213	-0.002	0.4404	0.004	0.5530	2E+06	PLA2G2A	aric	0.0291	215.8
s115000417	T	C	-0.382	0.0225	0.0563	1.3E-11	7213	0.008	0.0208	0.013	0.5513	2E+06	HTN3	aric	0.0063	46.0
rs74320783	A	G	-1.642	0.0123	0.0731	5.8E-108	7213	-0.026	0.0119	0.017	0.1352	2E+06	ITIH3	aric	0.0653	503.9
s115181845	T	C	0.463	0.0722	0.0316	4.3E-48	7213	0.014	0.0682	0.008	0.0838	2E+06	GSTM1	aric	0.0290	215.4
rs2269340	C	T	0.628	0.0753	0.0306	5.4E-91	7213	0.004	0.0798	0.007	0.5572	2E+06	GSTM1	aric	0.0552	421.0
s115214168	T	C	0.720	0.0261	0.0521	7.2E-43	7213	-0.010	0.0265	0.012	0.4228	2E+06	CXCL1	aric	0.0258	190.8
s140569632	A	G	0.373	0.0211	0.0569	5.5E-11	7213	0.002	0.0195	0.014	0.8856	2E+06	CXCL1	aric	0.0059	43.1
s115264949	A	G	0.780	0.0119	0.0759	1.4E-24	7213	-0.021	0.0143	0.017	0.2178	2E+06	FCGR2B	aric	0.0144	105.5
s116769960	T	G	0.576	0.0166	0.0651	1.1E-18	7213	-0.009	0.0213	0.014	0.5031	2E+06	FCGR2B	aric	0.0107	78.2
s115329695	G	A	0.754	0.0272	0.0501	2.2E-50	7213	-0.017	0.0278	0.012	0.1295	2E+06	KNG1	aric	0.0304	226.2
s144274836	T	C	-0.289	0.1114	0.0262	5.1E-28	7213	-0.002	0.1182	0.006	0.7412	2E+06	KNG1	aric	0.0166	121.4
s115370013	T	C	0.206	0.0623	0.0343	1.9E-09	7213	-0.002	0.0603	0.008	0.8235	2E+06	CNTN4	aric	0.0050	36.1
rs13071423	A	C	0.371	0.1812	0.0210	2.0E-68	7213	0.008	0.1918	0.005	0.1177	2E+06	CNTN4	aric	0.0415	312.0
rs11555096	T	C	-1.656	0.0214	0.0542	9.5E-193	7213	0.000	0.0253	0.012	0.9752	2E+06	FAH	aric	0.1145	932.4
s115645824	T	C	0.618	0.0275	0.0496	2.7E-35	7213	0.029	0.0295	0.012	0.0110	2E+06	LECT2	aric	0.0211	155.3
rs801576	T	C	-0.344	0.0934	0.0282	7.5E-34	7213	0.001	0.0993	0.007	0.9160	2E+06	LECT2	aric	0.0202	148.6
rs11591147	T	G	-1.045	0.0145	0.0687	2.4E-51	7213	0.023	0.0209	0.014	0.1059	2E+06	PCSK9	aric	0.0310	230.8
rs472495	G	T	-0.163	0.3569	0.0172	3.4E-21	7213	0.003	0.368	0.004	0.3759	2E+06	PCSK9	aric	0.0123	89.8

s116161634	T	C	-0.633	0.0218	0.0568	1.4E-28	7213	-0.002	0.0217	0.013	0.8940	2E+06	ACP6	aric	0.0169	124.0
s116523799	T	C	-0.582	0.0601	0.0347	3.9E-62	7213	-0.004	0.0605	0.008	0.6790	2E+06	ACP6	aric	0.0376	282.0
s143333817	G	A	-1.009	0.0119	0.0760	9.7E-40	7213	-0.021	0.0136	0.018	0.2392	2E+06	ACP6	aric	0.0238	176.1
s145317206	T	C	-1.138	0.0146	0.0674	7.6E-63	7213	-0.017	0.014	0.017	0.3291	2E+06	ACP6	aric	0.0381	285.4
rs76390540	A	G	-0.571	0.0238	0.0546	1.9E-25	7213	0.029	0.0303	0.011	0.0094	2E+06	ACP6	aric	0.0150	109.4
s116165405	G	T	-0.724	0.0214	0.0562	1.4E-37	7213	0.043	0.0206	0.014	0.0023	2E+06	IDH1	aric	0.0225	166.0
rs73070958	T	C	-0.847	0.0561	0.0349	3.7E-125	7213	-0.019	0.0544	0.009	0.0287	2E+06	IDH1	aric	0.0755	588.9
rs72659937	T	G	-0.193	0.0762	0.0312	6.8E-10	7213	0.007	0.0887	0.007	0.3318	2E+06	ANG	aric	0.0053	38.2
rs11629094	C	T	-0.164	0.2333	0.0195	6.0E-17	7213	0.006	0.2457	0.004	0.1869	2E+06	GALNT16	aric	0.0097	70.3
rs12100668	G	A	-0.314	0.4000	0.0166	2.6E-78	7213	0.016	0.398	0.004	0.0000	2E+06	GALNT16	aric	0.0475	359.5
rs1303451	T	C	-0.369	0.0289	0.0494	9.4E-14	7213	-0.010	0.027	0.013	0.4140	2E+06	GALNT16	aric	0.0077	55.7
rs4392019	T	C	0.635	0.0322	0.0466	9.4E-42	7213	0.003	0.0309	0.011	0.8148	2E+06	STARD5	aric	0.0251	185.6
rs11638020	A	G	0.252	0.3991	0.0166	5.1E-51	7213	-0.003	0.3828	0.004	0.4042	2E+06	CTSH	aric	0.0308	229.3
rs75299846	G	A	-0.314	0.0247	0.0540	6.4E-09	7213	-0.024	0.0583	0.011	0.0212	2E+06	CTSH	aric	0.0047	33.8
rs11667546	T	C	-0.583	0.1852	0.0205	3.1E-169	7213	-0.003	0.1923	0.005	0.5112	2E+06	IL27RA	aric	0.1011	811.2
rs34317732	T	C	-0.865	0.0189	0.0600	2.0E-46	7213	0.010	0.0216	0.014	0.4676	2E+06	IL27RA	aric	0.0280	207.6
rs2098953	C	T	-0.298	0.0548	0.0365	4.2E-16	7213	0.005	0.0533	0.009	0.5450	2E+06	LILRA2	aric	0.0091	66.4
rs11121209	G	A	-0.121	0.3749	0.0171	1.4E-12	7213	-0.009	0.3666	0.004	0.0215	2E+06	CA6	aric	0.0069	50.4
s116775713	A	C	0.430	0.0234	0.0549	5.6E-15	7213	-0.005	0.0267	0.012	0.6511	2E+06	CA6	aric	0.0084	61.3
rs35994272	C	T	0.466	0.0166	0.0653	1.0E-12	7213	0.009	0.0168	0.016	0.5678	2E+06	CA6	aric	0.0070	50.9
rs72641551	G	T	-0.455	0.0947	0.0280	4.0E-58	7213	0.007	0.0932	0.007	0.2575	2E+06	CA6	aric	0.0352	262.9
rs11680831	C	T	-0.514	0.3492	0.0163	4.0E-204	7213	-0.001	0.3479	0.004	0.7784	2E+06	NAGK	aric	0.1209	991.8
s116891509	T	C	0.557	0.0734	0.0311	4.1E-70	7213	-0.008	0.075	0.007	0.2312	2E+06	TXNL4B	aric	0.0425	320.2
s116977380	T	C	0.884	0.0368	0.0432	1.6E-90	7213	0.031	0.0467	0.01	0.0012	2E+06	PGP	aric	0.0549	418.7
rs11698358	G	A	0.596	0.0530	0.0364	5.2E-59	7213	0.014	0.0586	0.008	0.0742	2E+06	FLRT3	aric	0.0357	267.2
s117932517	C	T	-0.254	0.0376	0.0438	6.6E-09	7213	-0.003	0.0367	0.01	0.7925	2E+06	FLRT3	aric	0.0047	33.7
s144808273	G	A	-0.458	0.0269	0.0505	1.6E-19	7213	-0.001	0.0263	0.012	0.9357	2E+06	FLRT3	aric	0.0113	82.2
s145650062	T	G	-0.479	0.0134	0.0725	4.2E-11	7213	0.026	0.0146	0.016	0.1033	2E+06	FLRT3	aric	0.0060	43.6
rs56265150	A	G	0.410	0.1031	0.0268	7.0E-52	7213	0.011	0.0953	0.006	0.0903	2E+06	FLRT3	aric	0.0313	233.3
rs11698530	A	G	-0.639	0.0421	0.0405	3.9E-55	7213	0.000	0.042	0.009	0.9763	2E+06	BPI	aric	0.0333	248.8
rs6069524	C	T	0.315	0.3533	0.0169	6.0E-76	7213	-0.005	0.3556	0.004	0.2052	2E+06	BPI	aric	0.0461	348.2
s117014247	T	C	-0.856	0.0225	0.0555	7.2E-53	7213	-0.015	0.0217	0.014	0.2846	2E+06	ENGASE	aric	0.0320	238.0

s117502073	A	G	-0.996	0.0272	0.0496	2.3E-87	7213	0.028	0.0306	0.011	0.0112	2E+06	ENGASE	aric	0.0530	403.4
s117570724	A	G	-0.516	0.0148	0.0688	7.2E-14	7213	0.017	0.0142	0.017	0.3150	2E+06	ENGASE	aric	0.0077	56.2
rs3803780	G	A	-0.653	0.0409	0.0412	1.1E-55	7213	-0.015	0.037	0.01	0.1304	2E+06	ENGASE	aric	0.0337	251.3
s117029024	A	G	-0.433	0.0164	0.0656	4.2E-11	7213	-0.002	0.0182	0.015	0.9106	2E+06	RETN	aric	0.0060	43.6
rs11703790	C	T	0.482	0.1442	0.0229	1.0E-95	7213	0.000	0.1456	0.005	0.9999	2E+06	PLXNB2	aric	0.0580	444.0
rs73183310	G	A	0.113	0.3397	0.0175	1.1E-10	7213	-0.023	0.3274	0.004	0.0000	2E+06	PLXNB2	aric	0.0057	41.7
rs11704345	T	C	-0.122	0.2341	0.0196	5.0E-10	7213	0.003	0.2279	0.004	0.4736	2E+06	APOL3	aric	0.0053	38.8
rs11704700	T	C	0.120	0.2921	0.0183	6.6E-11	7213	-0.012	0.2851	0.004	0.0038	2E+06	SCUBE1	aric	0.0059	42.7
rs695786	T	C	-0.179	0.4703	0.0163	9.5E-28	7213	0.007	0.4781	0.004	0.0582	2E+06	SCUBE1	aric	0.0164	120.2
s117083993	G	A	0.977	0.0323	0.0453	3.4E-100	7213	-0.005	0.0355	0.011	0.6603	2E+06	CLEC11A	aric	0.0607	465.8
s117147437	A	G	0.657	0.0313	0.0475	6.0E-43	7213	0.026	0.0319	0.011	0.0221	2E+06	SAA4	aric	0.0258	191.2
rs1356979	C	T	-0.384	0.3912	0.0163	9.4E-118	7213	0.007	0.3995	0.004	0.0562	2E+06	SAA4	aric	0.0711	552.2
rs11727676	C	T	0.300	0.0907	0.0288	3.3E-25	7213	0.040	0.0964	0.006	0.0000	2E+06	HHIP	aric	0.0148	108.4
s117284274	T	G	-0.814	0.0363	0.0429	1.5E-78	7213	0.020	0.0346	0.011	0.0664	2E+06	SIRPB1	aric	0.0476	360.8
rs17791824	T	C	0.671	0.0182	0.0623	8.1E-27	7213	-0.020	0.0197	0.014	0.1764	2E+06	SIRPB1	aric	0.0158	115.8
rs62187494	A	G	0.440	0.0463	0.0390	2.5E-29	7213	0.004	0.0476	0.009	0.6319	2E+06	SIRPB1	aric	0.0174	127.5
rs73081790	A	G	-0.319	0.1487	0.0230	2.4E-43	7213	0.011	0.1422	0.005	0.0393	2E+06	SIRPB1	aric	0.0261	193.1
rs11729397	A	G	-0.315	0.0944	0.0283	1.6E-28	7213	-0.026	0.0928	0.007	0.0001	2E+06	HPGDS	aric	0.0169	123.8
rs28649197	G	T	0.512	0.0238	0.0540	3.1E-21	7213	-0.005	0.0228	0.013	0.6992	2E+06	HPGDS	aric	0.0123	90.0
rs74354031	C	T	-1.126	0.0220	0.0547	1.3E-91	7213	0.025	0.0434	0.011	0.0248	2E+06	HPGDS	aric	0.0555	424.0
rs78283599	G	A	-0.644	0.0669	0.0325	6.8E-85	7213	-0.027	0.0652	0.008	0.0004	2E+06	CPA4	aric	0.0515	391.4
rs9641869	G	A	0.254	0.1563	0.0226	3.2E-29	7213	0.004	0.1547	0.005	0.4223	2E+06	CPA4	aric	0.0173	127.0
s117396825	C	T	0.440	0.0121	0.0759	6.7E-09	7213	0.023	0.0128	0.018	0.1873	2E+06	TFF1	aric	0.0046	33.7
rs225358	C	T	-0.288	0.2978	0.0180	1.2E-56	7213	-0.005	0.2947	0.004	0.2494	2E+06	TFF1	aric	0.0343	256.0
s117399000	A	G	-0.735	0.0295	0.0480	5.1E-52	7213	-0.009	0.0419	0.01	0.3870	2E+06	CA10	aric	0.0314	234.0
rs2106331	A	G	-0.098	0.3486	0.0173	1.4E-08	7213	-0.008	0.3367	0.004	0.0510	2E+06	CA10	aric	0.0044	32.2
rs2938140	T	C	-0.167	0.4545	0.0166	1.4E-23	7213	-0.009	0.4556	0.004	0.0124	2E+06	CA10	aric	0.0138	100.9
rs79833227	A	C	-0.431	0.1802	0.0213	1.4E-88	7213	-0.001	0.1747	0.005	0.8440	2E+06	PLTP	aric	0.0537	409.2
s117482439	A	G	-0.742	0.0412	0.0410	9.5E-72	7213	0.014	0.0459	0.009	0.1250	2E+06	PDGFRL	aric	0.0435	328.0
rs79088892	T	G	-0.435	0.0293	0.0490	8.0E-19	7213	-0.002	0.0327	0.011	0.8377	2E+06	PDGFRL	aric	0.0108	78.9
rs11751347	T	C	-0.540	0.1014	0.0268	7.9E-88	7213	0.013	0.1011	0.006	0.0347	2E+06	PLG	aric	0.0533	405.6
s142126734	A	G	0.358	0.0507	0.0375	1.6E-21	7213	-0.021	0.0481	0.009	0.0198	2E+06	PLG	aric	0.0125	91.4

s11755773[illegible]	G	A	0.327	0.0292	0.0492	3.2E-11	7213	0.008	0.0324	0.011	0.4567	2E+06	MMP8	aric	0.0061	44.2
rs11757527	A	G	0.180	0.3050	0.0180	1.6E-23	7213	-0.003	0.2898	0.004	0.5165	2E+06	MDGA1	aric	0.0138	100.6
rs1619327	T	C	0.270	0.1007	0.0275	1.3E-22	7213	0.008	0.102	0.006	0.2194	2E+06	MDGA1	aric	0.0132	96.4
rs1776441	A	G	0.145	0.4971	0.0165	2.0E-18	7213	-0.005	0.4814	0.004	0.1563	2E+06	MDGA1	aric	0.0106	77.1
s11765869[illegible]	C	T	-0.406	0.0271	0.0502	7.2E-16	7213	-0.027	0.0315	0.011	0.0162	2E+06	NMRAL1	aric	0.0090	65.4
s11777390[illegible]	T	C	1.581	0.0193	0.0580	1.2E-155	7213	0.012	0.0279	0.012	0.3250	2E+06	NPTXR	aric	0.0933	742.1
s11778134[illegible]	T	C	-0.646	0.0133	0.0727	8.0E-19	7213	0.012	0.0134	0.016	0.4699	2E+06	CRTAC1	aric	0.0108	78.9
rs684225	A	C	-0.367	0.4148	0.0162	1.9E-110	7213	0.003	0.4265	0.004	0.4637	2E+06	CRTAC1	aric	0.0668	516.2
s11779931[illegible]	T	G	-0.338	0.0184	0.0617	4.4E-08	7213	-0.021	0.0251	0.013	0.0999	2E+06	NME4	aric	0.0041	30.0
rs6600214	T	C	0.174	0.3191	0.0176	9.1E-23	7213	0.011	0.3321	0.004	0.0050	2E+06	NME4	aric	0.0133	97.1
rs75203664	A	C	0.665	0.0218	0.0567	1.5E-31	7213	-0.001	0.0222	0.013	0.9223	2E+06	HP	aric	0.0188	137.8
rs9922883	T	C	0.413	0.0166	0.0652	2.5E-10	7213	-0.001	0.0167	0.015	0.9740	2E+06	HP	aric	0.0055	40.1
s11788624[illegible]	T	C	-0.659	0.0303	0.0486	2.4E-41	7213	0.012	0.0329	0.011	0.2609	2E+06	LILRB1	aric	0.0248	183.6
rs11794565	T	C	0.242	0.4563	0.0164	1.3E-48	7213	-0.013	0.4662	0.004	0.0011	2E+06	ENG	aric	0.0293	217.9
rs72748038	T	C	-0.530	0.0547	0.0361	3.7E-48	7213	0.012	0.0489	0.009	0.2042	2E+06	PTGR1	aric	0.0291	215.7
rs74602773	A	G	0.888	0.0215	0.0569	5.0E-54	7213	-0.011	0.0258	0.012	0.3746	2E+06	PTGR1	aric	0.0327	243.5
s11796646[illegible]	T	G	-0.356	0.0297	0.0492	5.4E-13	7213	-0.006	0.0334	0.011	0.5798	2E+06	SEZ6L	aric	0.0072	52.2
rs6005031	G	A	-0.146	0.4952	0.0165	1.0E-18	7213	0.009	0.4954	0.004	0.0164	2E+06	SEZ6L	aric	0.0108	78.4
s11816172[illegible]	G	A	-0.712	0.0811	0.0292	1.6E-126	7213	0.001	0.0795	0.007	0.8503	2E+06	SEMA3E	aric	0.0763	595.7
rs4577905	A	G	0.241	0.0476	0.0391	6.8E-10	7213	-0.003	0.0443	0.009	0.7619	2E+06	SEMA3E	aric	0.0053	38.2
rs11850199	A	C	-0.191	0.2715	0.0183	3.6E-25	7213	-0.002	0.2587	0.004	0.7073	2E+06	SERPINA9	aric	0.0148	108.2
rs11886092	G	A	0.393	0.3791	0.0163	1.0E-122	7213	0.001	0.3749	0.004	0.8699	2E+06	MATN3	aric	0.0741	576.8
rs3771248	G	A	0.126	0.4594	0.0166	3.7E-14	7213	-0.001	0.4587	0.004	0.7961	2E+06	MATN3	aric	0.0079	57.6
rs9306885	C	T	-0.114	0.2417	0.0193	3.4E-09	7213	0.005	0.2546	0.004	0.2749	2E+06	MATN3	aric	0.0048	35.0
rs11900990	G	T	-0.160	0.1338	0.0244	6.3E-11	7213	0.007	0.1321	0.006	0.2196	2E+06	EDAR	aric	0.0059	42.8
rs77304476	G	A	1.475	0.0134	0.0695	5.0E-97	7213	-0.021	0.0138	0.016	0.1904	2E+06	EDAR	aric	0.0588	450.3
rs11909509	A	G	-0.298	0.2035	0.0204	2.1E-47	7213	0.001	0.2015	0.005	0.7754	2E+06	COL18A1	aric	0.0286	212.2
rs12024571	T	G	-0.721	0.0354	0.0435	1.3E-60	7213	0.008	0.0499	0.009	0.4108	2E+06	PTGFRN	aric	0.0367	274.8
rs12037202	C	T	-0.414	0.1497	0.0229	3.1E-71	7213	0.008	0.1575	0.005	0.1023	2E+06	PTGFRN	aric	0.0432	325.5
rs75567925	A	G	-1.134	0.0146	0.0680	3.4E-61	7213	-0.002	0.0144	0.017	0.9301	2E+06	PTGFRN	aric	0.0371	277.6
rs12067235	T	G	0.330	0.2144	0.0200	5.2E-60	7213	-0.009	0.2118	0.005	0.0488	2E+06	CNTN2	aric	0.0363	271.9
rs2802840	T	C	0.376	0.0801	0.0305	1.6E-34	7213	0.000	0.0927	0.007	0.9822	2E+06	CNTN2	aric	0.0206	151.8

rs3903399	C	T	-0.384	0.2080	0.0200	3.5E-80	7213	0.035	0.2122	0.005	0.0000	2E+06	CNTN2	aric	0.0486	368.6
rs12074147	C	T	-0.589	0.3645	0.0158	3.6E-278	7213	0.001	0.3673	0.004	0.8757	2E+06	PPIE	aric	0.1615	1388.5
rs12117281	C	T	0.196	0.1157	0.0261	7.5E-14	7213	-0.001	0.1163	0.006	0.9152	2E+06	MAN1A2	aric	0.0077	56.1
rs73013841	T	C	-0.565	0.1151	0.0252	1.3E-107	7213	-0.007	0.1173	0.006	0.2500	2E+06	MAN1A2	aric	0.0651	502.3
rs12121180	T	C	0.379	0.1576	0.0224	2.8E-63	7213	-0.015	0.1535	0.005	0.0052	2E+06	IL6R	aric	0.0383	287.5
's14276804Ź	T	C	-0.438	0.0261	0.0519	4.1E-17	7213	-0.003	0.0281	0.012	0.8048	2E+06	IL6R	aric	0.0098	71.0
rs57569414	A	C	0.517	0.1209	0.0245	2.9E-96	7213	0.003	0.1215	0.006	0.6564	2E+06	IL6R	aric	0.0583	446.6
rs12129832	C	T	-0.223	0.0505	0.0378	3.8E-09	7213	0.008	0.0446	0.009	0.4046	2E+06	GLRX2	aric	0.0048	34.8
's14821259(	G	A	1.017	0.0177	0.0622	6.0E-59	7213	-0.004	0.0194	0.014	0.7624	2E+06	GLRX2	aric	0.0357	266.9
rs12146099	A	G	0.288	0.3932	0.0167	1.7E-65	7213	0.019	0.3969	0.004	0.0000	2E+06	LAMC2	aric	0.0397	298.1
rs12192369	G	A	-0.459	0.1440	0.0229	4.8E-87	7213	0.002	0.1456	0.005	0.7264	2E+06	PREP	aric	0.0528	401.8
rs12205095	G	T	-0.609	0.3607	0.0156	2.9E-302	7213	0.004	0.359	0.004	0.3268	2E+06	VNN2	aric	0.1743	1521.7
rs10999762	G	A	0.200	0.2256	0.0198	7.4E-24	7213	-0.006	0.2262	0.004	0.1623	2E+06	UNC5B	aric	0.0140	102.1
rs12318199	A	G	0.449	0.0507	0.0372	3.8E-33	7213	-0.029	0.0484	0.009	0.0009	2E+06	ART4	aric	0.0197	145.3
's13858560(	T	C	0.443	0.0193	0.0597	1.2E-13	7213	0.015	0.0161	0.016	0.3449	2E+06	ART4	aric	0.0076	55.1
rs12326826	C	T	-0.128	0.2779	0.0185	5.3E-12	7213	0.002	0.2896	0.004	0.7021	2E+06	CNDP1	aric	0.0066	47.7
rs17817077	A	G	0.245	0.3957	0.0168	1.5E-47	7213	0.004	0.389	0.004	0.3374	2E+06	CNDP1	aric	0.0287	212.8
rs1238449	T	C	-0.154	0.4973	0.0165	1.0E-20	7213	-0.005	0.5039	0.004	0.1499	2E+06	LIPN	aric	0.0120	87.6
rs2576155	C	T	0.338	0.2891	0.0180	1.1E-76	7213	-0.006	0.277	0.004	0.1351	2E+06	LIPN	aric	0.0465	351.7
rs12423250	T	C	-0.256	0.1798	0.0213	3.4E-33	7213	0.009	0.1787	0.005	0.0708	2E+06	COL2A1	aric	0.0198	145.5
rs12459073	A	G	-0.674	0.1795	0.0205	3.4E-222	7213	0.004	0.1755	0.005	0.4628	2E+06	IGFLR1	aric	0.1310	1086.9
rs12461127	T	C	0.113	0.4737	0.0167	1.5E-11	7213	0.009	0.4696	0.004	0.0186	2E+06	BCAM	aric	0.0063	45.6
rs8105118	C	T	0.344	0.1239	0.0249	7.6E-43	7213	0.002	0.1293	0.006	0.6980	2E+06	GDF15	aric	0.0258	190.7
rs12535512	C	T	0.392	0.4289	0.0161	1.0E-125	7213	-0.006	0.4358	0.004	0.1332	2E+06	ADAM22	aric	0.0758	591.7
rs35844181	A	C	0.236	0.3209	0.0177	2.6E-40	7213	-0.003	0.3354	0.004	0.4770	2E+06	ADAM22	aric	0.0242	178.8
rs35186095	T	C	-0.540	0.1488	0.0225	4.9E-123	7213	-0.007	0.1511	0.006	0.1806	2E+06	ICAM1	aric	0.0743	578.4
rs12635047	A	G	0.265	0.1495	0.0229	8.7E-31	7213	-0.001	0.1502	0.005	0.8780	2E+06	XXYLT1	aric	0.0183	134.3
rs12695049	C	T	-0.291	0.4234	0.0166	2.9E-67	7213	-0.007	0.4234	0.004	0.0548	2E+06	XXYLT1	aric	0.0408	306.5
rs12657079	C	T	0.322	0.1431	0.0235	2.3E-42	7213	-0.020	0.1606	0.005	0.0002	2E+06	PAM	aric	0.0255	188.4
rs12664015	A	G	-0.372	0.1212	0.0248	5.6E-50	7213	0.007	0.1196	0.006	0.2400	2E+06	THBS2	aric	0.0302	224.3
rs73034020	T	C	0.476	0.0896	0.0288	2.1E-60	7213	0.017	0.0939	0.007	0.0106	2E+06	THBS2	aric	0.0366	273.8
rs3136630	T	C	-0.457	0.2961	0.0175	2.3E-144	7213	0.007	0.2923	0.004	0.1036	2E+06	IL15RA	aric	0.0868	685.0

rs74116316	G	A	0.112	0.2307	0.0195	8.4E-09	7213	-0.009	0.2245	0.004	0.0443	2E+06	IL15RA	aric	0.0046	33.2
rs12763713	A	C	-0.355	0.0315	0.0472	6.2E-14	7213	-0.007	0.0306	0.012	0.5292	1E+06	ASAH2	aric	0.0078	56.5
rs14662274[illegible]	A	G	-0.787	0.0294	0.0480	1.9E-59	7213	0.013	0.0297	0.012	0.2702	2E+06	ASAH2	aric	0.0360	269.2
rs12877225	A	G	-0.223	0.2913	0.0181	1.8E-34	7213	0.007	0.2882	0.004	0.0873	2E+06	SLITRK5	aric	0.0206	151.5
rs12941038	T	C	-0.266	0.2366	0.0195	7.1E-42	7213	0.014	0.2335	0.004	0.0014	2E+06	FAM20A	aric	0.0252	186.1
rs929477	A	G	-0.324	0.0914	0.0286	1.4E-29	7213	0.001	0.0968	0.006	0.9046	2E+06	FAM20A	aric	0.0175	128.7
rs12950560	C	T	-0.100	0.4125	0.0169	2.7E-09	7213	0.000	0.42	0.004	0.9822	2E+06	CCL3	aric	0.0049	35.4
rs12980031	G	T	0.228	0.2414	0.0192	3.8E-32	7213	-0.007	0.2469	0.005	0.1099	2E+06	FCER2	aric	0.0191	140.6
rs12974746	G	A	-0.699	0.0256	0.0521	1.6E-40	7213	-0.013	0.0278	0.012	0.2916	2E+06	COMP	aric	0.0243	179.8
rs12980552	G	A	-0.382	0.4957	0.0160	3.7E-121	7213	0.004	0.5002	0.004	0.2575	2E+06	CCL25	aric	0.0731	569.1
rs78039161	C	T	-0.355	0.1088	0.0263	3.6E-41	7213	-0.003	0.1107	0.006	0.6580	2E+06	CCL25	aric	0.0247	182.8
rs12984853	A	G	-0.527	0.3850	0.0160	2.1E-221	7213	-0.003	0.3896	0.004	0.4777	2E+06	SIGLEC9	aric	0.1305	1082.6
rs2072689	T	C	-0.221	0.4741	0.0164	1.1E-40	7213	0.003	0.472	0.004	0.4158	2E+06	SIGLEC9	aric	0.0244	180.6
rs12990312	T	C	-0.321	0.1223	0.0253	1.0E-36	7213	-0.004	0.1262	0.006	0.4313	2E+06	REG1A	aric	0.0220	161.9
rs12999504	G	T	-0.423	0.0366	0.0438	6.4E-22	7213	0.000	0.0403	0.01	0.9917	2E+06	REG1A	aric	0.0128	93.2
rs76841471	G	T	0.510	0.0741	0.0311	2.5E-59	7213	0.001	0.0728	0.007	0.8868	2E+06	REG1A	aric	0.0359	268.6
rs13008230	G	T	-0.937	0.0545	0.0350	3.8E-150	7213	-0.002	0.0537	0.008	0.8394	2E+06	GKN2	aric	0.0901	714.2
rs11129766	G	A	-0.354	0.0174	0.0636	2.7E-08	7213	0.000	0.0222	0.015	0.9837	2E+06	CHL1	aric	0.0043	30.9
rs13077895	G	A	-0.305	0.3562	0.0172	3.2E-69	7213	0.003	0.3547	0.004	0.5269	2E+06	CHL1	aric	0.0420	315.9
rs13091025	C	A	-0.292	0.1278	0.0248	9.9E-32	7213	0.021	0.1358	0.006	0.0001	2E+06	SEMA3G	aric	0.0189	138.7
rs13100619	C	A	-1.232	0.0528	0.0344	6.8E-258	7213	-0.006	0.0556	0.008	0.4509	2E+06	BTD	aric	0.1505	1278.0
rs13115901	A	G	-0.154	0.2064	0.0204	4.5E-14	7213	-0.004	0.194	0.005	0.4044	2E+06	FGF2	aric	0.0079	57.2
rs6833731	A	G	0.379	0.3221	0.0173	2.2E-103	7213	0.002	0.3173	0.004	0.5732	2E+06	ENPEP	aric	0.0626	481.4
rs13125919	T	C	0.387	0.1054	0.0269	4.4E-46	7213	0.014	0.0986	0.006	0.0243	2E+06	ADH5	aric	0.0278	205.9
rs28894371	A	G	-0.976	0.0379	0.0423	1.9E-113	7213	-0.025	0.0379	0.01	0.0092	2E+06	ADH5	aric	0.0686	531.0
rs13143783	T	C	-0.472	0.0900	0.0285	1.3E-60	7213	0.002	0.0851	0.007	0.7259	2E+06	SPOCK3	aric	0.0367	274.8
rs77904897	C	A	-0.411	0.0213	0.0575	1.0E-12	7213	0.004	0.0214	0.014	0.7716	2E+06	SPOCK3	aric	0.0070	51.0
rs13144424	A	G	-0.314	0.2035	0.0203	2.9E-53	7213	-0.001	0.201	0.005	0.7851	2E+06	SPARCL1	aric	0.0322	239.9
rs13158921	T	C	-0.465	0.0166	0.0650	9.3E-13	7213	-0.012	0.0171	0.015	0.4116	2E+06	UBLCP1	aric	0.0070	51.2
rs13164140	A	G	0.463	0.2472	0.0185	7.4E-132	7213	0.004	0.2481	0.004	0.3161	2E+06	HEXB	aric	0.0794	622.3
rs13209147	A	G	0.121	0.2034	0.0206	4.4E-09	7213	0.012	0.2	0.005	0.0129	2E+06	MLN	aric	0.0048	34.5
rs2296329	G	A	-0.334	0.1987	0.0203	6.8E-60	7213	-0.021	0.2075	0.005	0.0000	2E+06	MLN	aric	0.0363	271.4

rs13216122	C	T	-0.730	0.0795	0.0294	6.1E-131	7213	0.000	0.0749	0.007	0.9560	2E+06	RNASET2	aric	0.0789	617.7
rs13263968	C	T	0.383	0.1919	0.0205	2.4E-76	7213	0.003	0.1869	0.005	0.4846	2E+06	PENK	aric	0.0463	350.1
rs2610035	A	G	0.141	0.4432	0.0165	1.6E-17	7213	-0.018	0.4436	0.004	0.0000	2E+06	PENK	aric	0.0100	73.0
rs1330064	A	G	-0.315	0.3458	0.0172	2.4E-73	7213	-0.002	0.3392	0.004	0.5914	2E+06	GPC5	aric	0.0445	335.7
s13928238	A	G	-0.491	0.1527	0.0221	7.5E-106	7213	-0.012	0.1596	0.005	0.0216	2E+06	GPC5	aric	0.0641	493.6
rs13342837	T	C	0.743	0.3081	0.0157	0.0E+00	7213	0.008	0.3025	0.004	0.0722	2E+06	SCARF1	aric	0.2367	2236.7
rs13375787	T	C	0.985	0.0378	0.0421	9.2E-117	7213	0.000	0.067	0.009	0.9651	2E+06	CRYZ	aric	0.0705	547.3
rs277392	G	T	-0.215	0.2810	0.0182	6.8E-32	7213	-0.003	0.2865	0.004	0.5448	2E+06	CRYZ	aric	0.0190	139.4
rs76296629	T	G	0.646	0.0541	0.0361	3.5E-70	7213	0.012	0.0515	0.009	0.1673	2E+06	CRYZ	aric	0.0426	320.5
rs1344544	G	A	0.243	0.3178	0.0176	5.7E-43	7213	0.007	0.3175	0.004	0.0739	2E+06	MMAB	aric	0.0258	191.3
rs61898281	G	A	0.880	0.0517	0.0360	1.2E-126	7213	-0.002	0.0509	0.008	0.8473	2E+06	OAF	aric	0.0764	596.4
rs1363864	C	T	-0.101	0.4999	0.0166	1.3E-09	7213	0.002	0.5058	0.004	0.5554	2E+06	ESM1	aric	0.0051	36.9
rs4242051	T	C	-0.181	0.2489	0.0191	3.8E-21	7213	0.002	0.2528	0.004	0.5759	2E+06	ESM1	aric	0.0123	89.6
rs1380642	T	C	-0.515	0.2032	0.0199	5.9E-142	7213	-0.017	0.197	0.005	0.0002	2E+06	MTHFS	aric	0.0854	672.9
rs72736560	A	C	0.567	0.0276	0.0499	1.3E-29	7213	0.013	0.0259	0.012	0.2657	2E+06	MTHFS	aric	0.0176	128.8
s13807324	A	C	0.372	0.0315	0.0474	5.4E-15	7213	0.002	0.0358	0.01	0.8429	2E+06	CPN2	aric	0.0084	61.4
rs6774800	A	C	-0.468	0.2936	0.0174	1.6E-152	7213	-0.004	0.3041	0.004	0.3904	2E+06	CPN2	aric	0.0915	726.1
s13883488	A	C	0.558	0.0123	0.0754	1.5E-13	7213	-0.020	0.0141	0.017	0.2390	2E+06	UCMA	aric	0.0075	54.8
s13927726	A	G	-0.422	0.0241	0.0545	1.1E-14	7213	0.015	0.0262	0.012	0.2215	2E+06	CD109	aric	0.0082	59.9
rs76081669	T	C	-0.307	0.0302	0.0484	2.3E-10	7213	-0.001	0.0305	0.011	0.9306	2E+06	CD109	aric	0.0056	40.3
rs140174	G	A	-0.213	0.2779	0.0184	1.1E-30	7213	-0.002	0.271	0.004	0.5745	2E+06	IGLL1	aric	0.0182	133.8
s14059004	G	A	-0.746	0.0143	0.0698	1.7E-26	7213	0.012	0.0135	0.016	0.4703	2E+06	CNRIP1	aric	0.0156	114.4
s18483246	T	C	-0.631	0.0293	0.0492	2.9E-37	7213	-0.014	0.0261	0.012	0.2467	2E+06	CNRIP1	aric	0.0223	164.5
rs7592976	G	A	-0.738	0.0764	0.0300	3.4E-128	7213	-0.006	0.0841	0.007	0.3866	2E+06	CNRIP1	aric	0.0773	604.1
s14086496	A	G	-0.954	0.0195	0.0591	1.3E-57	7213	-0.009	0.0229	0.013	0.5182	2E+06	MST1	aric	0.0349	260.5
rs34810691	A	G	0.316	0.0229	0.0558	1.5E-08	7213	0.015	0.0259	0.012	0.2216	2E+06	MST1	aric	0.0044	32.1
s14195054	T	C	-0.830	0.0272	0.0506	1.9E-59	7213	0.004	0.0256	0.012	0.7527	2E+06	ADAM23	aric	0.0360	269.2
rs78652356	G	A	0.565	0.0136	0.0717	3.7E-15	7213	0.007	0.0204	0.014	0.6078	2E+06	ADAM23	aric	0.0085	62.1
rs79387475	A	G	0.327	0.1749	0.0218	4.8E-50	7213	-0.007	0.1693	0.005	0.1790	2E+06	ADAM23	aric	0.0302	224.6
s14208657	T	C	0.451	0.0107	0.0813	3.0E-08	7213	0.017	0.0121	0.018	0.3410	2E+06	LUM	aric	0.0042	30.8
rs1803343	C	T	-0.606	0.0222	0.0558	3.2E-27	7213	0.016	0.0214	0.014	0.2272	2E+06	LUM	aric	0.0161	117.7
rs77751442	T	G	-0.729	0.0189	0.0607	6.7E-33	7213	0.009	0.0203	0.013	0.4975	2E+06	LUM	aric	0.0196	144.1

s142332135	A	G	-0.700	0.0227	0.0545	2.2E-37	7213	-0.010	0.025	0.013	0.4582	2E+06	IL16	aric	0.0224	165.1
rs17875585	T	G	0.471	0.0171	0.0642	2.4E-13	7213	-0.007	0.0189	0.014	0.6295	2E+06	IL16	aric	0.0074	53.8
rs4778640	G	A	-1.299	0.0315	0.0455	8.7E-170	7213	0.021	0.0308	0.011	0.0576	2E+06	IL16	aric	0.1014	814.1
rs72746169	T	C	-1.270	0.0179	0.0613	1.7E-92	7213	-0.017	0.0188	0.015	0.2556	2E+06	IL16	aric	0.0561	428.2
s142439242	G	A	-1.121	0.0166	0.0630	2.8E-69	7213	0.000	0.0152	0.016	0.9934	2E+06	CPQ	aric	0.0420	316.2
rs72680193	A	C	-0.592	0.0362	0.0440	7.2E-41	7213	0.003	0.0364	0.01	0.7659	2E+06	CPQ	aric	0.0245	181.4
rs76898837	T	G	-0.485	0.0177	0.0627	1.2E-14	7213	-0.008	0.0178	0.015	0.6038	2E+06	CPQ	aric	0.0082	59.8
s143104579	A	G	0.423	0.0175	0.0634	2.7E-11	7213	0.015	0.0188	0.014	0.2841	2E+06	BTN3A3	aric	0.0061	44.5
rs72845505	G	A	-0.366	0.0207	0.0581	3.3E-10	7213	-0.022	0.024	0.013	0.0806	2E+06	BTN3A3	aric	0.0055	39.6
s143257534	T	C	-0.887	0.0277	0.0497	9.7E-70	7213	-0.011	0.0267	0.012	0.3583	2E+06	ADIPOQ	aric	0.0423	318.4
s144449914	A	C	0.486	0.0231	0.0546	6.7E-19	7213	-0.019	0.0235	0.014	0.1566	2E+06	C1QC	aric	0.0109	79.3
rs17452514	T	C	-0.372	0.0162	0.0657	1.6E-08	7213	0.001	0.0207	0.014	0.9556	2E+06	FCGR2A	aric	0.0044	32.0
rs76034548	C	T	-0.609	0.0284	0.0493	1.1E-34	7213	0.024	0.0304	0.011	0.0299	2E+06	ATRN	aric	0.0207	152.4
s144655897	T	C	0.480	0.0127	0.0742	1.1E-10	7213	-0.022	0.0126	0.017	0.2095	2E+06	OGN	aric	0.0058	41.8
rs62565677	C	T	0.295	0.0534	0.0370	1.6E-15	7213	0.002	0.0551	0.009	0.8528	2E+06	OGN	aric	0.0088	63.7
rs7022820	C	T	0.405	0.2547	0.0184	1.5E-103	7213	-0.011	0.2597	0.004	0.0077	2E+06	OGN	aric	0.0627	482.2
s144674978	T	C	0.469	0.0116	0.0776	1.6E-09	7213	-0.005	0.0122	0.019	0.8014	2E+06	GREM1	aric	0.0050	36.5
rs17816447	T	C	0.422	0.0232	0.0547	1.4E-14	7213	-0.006	0.0252	0.012	0.6031	2E+06	GREM1	aric	0.0082	59.4
rs4779584	T	C	0.829	0.1849	0.0192	0.0E+00	7213	-0.007	0.2077	0.005	0.1516	2E+06	GREM1	aric	0.2063	1874.1
s145188037	A	G	1.114	0.0161	0.0645	1.6E-65	7213	-0.015	0.019	0.015	0.3176	2E+06	IGFBP3	aric	0.0397	298.1
s145767181	G	A	-0.441	0.0131	0.0731	1.7E-09	7213	-0.005	0.0146	0.016	0.7778	2E+06	CD300C	aric	0.0050	36.4
rs72844326	G	T	-0.761	0.0494	0.0373	8.0E-90	7213	-0.001	0.0466	0.009	0.9286	2E+06	CD300C	aric	0.0545	415.3
s145851867	A	G	-0.374	0.0233	0.0550	1.1E-11	7213	0.004	0.0224	0.013	0.7585	2E+06	GUSB	aric	0.0064	46.2
s146174215	C	A	-0.901	0.0243	0.0529	9.3E-64	7213	-0.014	0.0264	0.012	0.2243	2E+06	APCS	aric	0.0386	289.8
rs28383573	T	G	0.381	0.0988	0.0273	1.3E-43	7213	-0.009	0.11	0.006	0.1338	2E+06	APCS	aric	0.0262	194.4
s146385050	A	C	-0.414	0.1964	0.0205	3.4E-88	7213	0.020	0.1823	0.005	0.0001	2E+06	MRC2	aric	0.0535	407.4
s146637002	T	C	0.362	0.0301	0.0484	7.8E-14	7213	-0.004	0.0287	0.012	0.7281	2E+06	FUT10	aric	0.0077	56.1
rs4733407	C	T	0.339	0.2654	0.0183	1.1E-74	7213	-0.007	0.264	0.004	0.1009	2E+06	FUT10	aric	0.0453	342.2
rs7838624	C	A	0.253	0.4198	0.0165	5.0E-52	7213	-0.004	0.4237	0.004	0.3302	2E+06	FUT10	aric	0.0314	234.0
s147145691	T	C	-0.597	0.0227	0.0557	1.4E-26	7213	0.005	0.0222	0.013	0.7215	2E+06	DSG2	aric	0.0157	114.8
rs9304098	T	G	-0.323	0.4558	0.0162	2.7E-86	7213	0.003	0.4554	0.004	0.4711	2E+06	DSG2	aric	0.0523	398.2
s147236212	G	A	0.345	0.0195	0.0598	8.3E-09	7213	-0.013	0.023	0.014	0.3225	2E+06	SMAP1	aric	0.0046	33.3

rs2691477	A	G	-0.721	0.2812	0.0165	0.0E+00	7213	-0.003	0.2869	0.004	0.4624	2E+06	SMAP1	aric	0.2086	1900.2
s192818776	C	A	-0.676	0.0134	0.0723	1.2E-20	7213	-0.016	0.0162	0.017	0.3393	2E+06	ITPA	aric	0.0120	87.3
s148054374	A	G	-1.050	0.0118	0.0766	2.9E-42	7213	0.020	0.011	0.019	0.2819	2E+06	PPIL1	aric	0.0254	188.0
s148215732	C	T	0.771	0.0164	0.0654	8.2E-32	7213	-0.046	0.0156	0.015	0.0023	2E+06	NAGLU	aric	0.0189	139.1
rs58947745	A	G	0.894	0.0209	0.0577	2.5E-53	7213	-0.001	0.0197	0.014	0.9464	2E+06	NAGLU	aric	0.0322	240.2
rs13047780	T	C	0.124	0.1924	0.0210	3.5E-09	7213	-0.025	0.1996	0.005	0.0000	2E+06	COL6A2	aric	0.0048	34.9
s148337125	G	T	0.512	0.0251	0.0518	6.6E-23	7213	0.001	0.0277	0.012	0.9511	2E+06	COL6A2	aric	0.0134	97.7
rs35548026	A	G	-0.548	0.0870	0.0288	1.0E-78	7213	0.029	0.0859	0.007	0.0000	2E+06	COL6A2	aric	0.0477	361.6
s148541330	G	A	0.916	0.0125	0.0741	8.3E-35	7213	0.030	0.0164	0.017	0.0661	2E+06	CHIT1	aric	0.0208	153.0
rs74736715	C	A	0.479	0.0558	0.0355	6.0E-41	7213	0.017	0.051	0.008	0.0387	2E+06	SWAP70	aric	0.0246	181.8
s148659834	A	G	-0.854	0.0136	0.0702	1.1E-33	7213	0.001	0.0143	0.016	0.9493	2E+06	LGMN	aric	0.0201	147.8
s149935681	A	G	1.344	0.0265	0.0497	9.9E-154	7213	-0.038	0.027	0.012	0.0011	2E+06	CTRB1	aric	0.0922	732.3
rs9928744	T	C	0.158	0.1990	0.0209	5.7E-14	7213	0.017	0.199	0.005	0.0002	2E+06	CTRB1	aric	0.0078	56.7
s150331426	G	A	0.484	0.0141	0.0695	3.7E-12	7213	0.007	0.017	0.015	0.6269	2E+06	ERPINA10	aric	0.0067	48.4
s150394890	T	G	-1.428	0.0110	0.0786	4.2E-72	7213	-0.042	0.0102	0.02	0.0361	2E+06	CAPN2	aric	0.0437	329.7
s151241919	T	C	-0.489	0.0197	0.0596	2.7E-16	7213	-0.008	0.0214	0.013	0.5354	2E+06	DPT	aric	0.0092	67.3
s151250811	T	C	0.571	0.0212	0.0574	3.8E-23	7213	-0.015	0.0193	0.014	0.2719	2E+06	DPT	aric	0.0135	98.8
rs580360	T	C	-0.321	0.4398	0.0162	7.7E-85	7213	0.007	0.435	0.004	0.0630	2E+06	DPT	aric	0.0514	391.1
rs1538821	G	A	0.225	0.0673	0.0329	8.4E-12	7213	-0.003	0.0704	0.007	0.6991	2E+06	ANXA11	aric	0.0065	46.8
rs2152546	T	G	-0.599	0.4846	0.0151	0.0E+00	7213	-0.002	0.4838	0.004	0.6686	2E+06	ANXA11	aric	0.1785	1566.7
rs1539790	A	G	-0.195	0.2344	0.0199	1.6E-22	7213	0.005	0.2418	0.004	0.2505	2E+06	COLEC12	aric	0.0131	96.0
rs1561369	A	G	0.634	0.1178	0.0248	1.6E-137	7213	-0.010	0.1141	0.006	0.0892	2E+06	FRZB	aric	0.0828	650.6
rs72890325	G	T	-0.544	0.0932	0.0280	3.7E-82	7213	-0.004	0.0864	0.007	0.5923	2E+06	FRZB	aric	0.0498	378.2
rs1650127	T	C	-0.544	0.3077	0.0170	1.4E-210	7213	0.000	0.3087	0.004	0.9966	2E+06	CHST11	aric	0.1245	1025.5
rs856595	T	C	0.101	0.4407	0.0166	1.4E-09	7213	0.004	0.4437	0.004	0.2420	2E+06	CHST11	aric	0.0051	36.8
rs1675513	G	A	-0.267	0.1556	0.0227	9.7E-32	7213	-0.007	0.1666	0.005	0.1436	2E+06	CPOX	aric	0.0189	138.7
rs16829593	G	T	0.279	0.2355	0.0192	6.9E-47	7213	-0.010	0.2396	0.004	0.0267	2E+06	TNFAIP6	aric	0.0283	209.7
rs16851364	A	G	-0.446	0.1201	0.0250	5.6E-70	7213	0.004	0.1177	0.006	0.4584	2E+06	FMOD	aric	0.0424	319.5
rs16854533	A	G	0.491	0.0878	0.0290	2.7E-63	7213	0.020	0.102	0.006	0.0021	2E+06	NFASC	aric	0.0383	287.5
rs6656887	C	T	0.821	0.1068	0.0251	9.2E-219	7213	0.001	0.1035	0.006	0.8368	2E+06	NFASC	aric	0.1291	1068.7
rs34411312	C	T	0.130	0.4836	0.0166	6.4E-15	7213	0.008	0.4788	0.004	0.0276	2E+06	LIFR	aric	0.0084	61.0
rs16918163	G	A	0.498	0.1185	0.0250	5.6E-86	7213	-0.008	0.1151	0.006	0.1863	2E+06	COL15A1	aric	0.0521	396.6

rs2600261	C	T	-0.431	0.2196	0.0196	1.5E-103	7213	-0.008	0.2223	0.005	0.0679	2E+06	TIMP4	aric	0.0627	482.2
rs17077267	T	C	-0.304	0.1470	0.0232	1.1E-38	7213	0.004	0.1489	0.005	0.4837	2E+06	CDCP1	aric	0.0232	171.1
rs17168679	C	T	0.408	0.0204	0.0585	3.2E-12	7213	0.035	0.0239	0.013	0.0056	2E+06	TAC1	aric	0.0067	48.7
rs3779470	A	G	-0.266	0.1697	0.0219	1.2E-33	7213	-0.005	0.1859	0.005	0.3325	2E+06	TAC1	aric	0.0201	147.6
rs13244925	A	C	0.170	0.4487	0.0166	1.6E-24	7213	-0.007	0.4472	0.004	0.0516	2E+06	EGFR	aric	0.0144	105.2
rs17172451	A	G	0.127	0.2395	0.0195	6.1E-11	7213	-0.006	0.2425	0.004	0.1396	2E+06	EGFR	aric	0.0059	42.9
rs17176065	T	C	0.540	0.0340	0.0454	2.7E-32	7213	-0.017	0.0362	0.01	0.0988	2E+06	CYB5D2	aric	0.0192	141.3
rs17288108	G	A	-0.373	0.1760	0.0213	6.4E-67	7213	-0.009	0.174	0.005	0.0725	2E+06	NTN4	aric	0.0406	304.9
rs17383694	G	A	0.309	0.3526	0.0170	6.5E-72	7213	-0.007	0.3658	0.004	0.0836	2E+06	UNC5C	aric	0.0436	328.8
rs17447113	G	T	0.404	0.0322	0.0468	7.2E-18	7213	-0.007	0.0378	0.01	0.5004	2E+06	NTNG1	aric	0.0102	74.5
rs17554536	A	G	0.149	0.1692	0.0221	1.5E-11	7213	0.005	0.198	0.005	0.3159	2E+06	NID1	aric	0.0063	45.7
rs17599360	G	A	-0.624	0.0365	0.0435	5.5E-46	7213	0.003	0.0505	0.009	0.7802	2E+06	SPINK6	aric	0.0277	205.5
rs2161431	T	C	-0.293	0.0347	0.0454	1.2E-10	7213	-0.008	0.0319	0.011	0.4711	2E+06	SPINK6	aric	0.0057	41.6
rs17647647	C	T	-0.178	0.2179	0.0200	5.7E-19	7213	0.001	0.2175	0.005	0.8645	2E+06	CPB2	aric	0.0109	79.6
rs2573284	T	C	0.122	0.3002	0.0182	1.8E-11	7213	-0.004	0.3073	0.004	0.3057	2E+06	CPB2	aric	0.0063	45.4
rs7325308	T	C	0.528	0.3840	0.0160	1.3E-222	7213	0.007	0.3872	0.004	0.0691	2E+06	CPB2	aric	0.1312	1089.1
rs34463787	T	C	-0.416	0.2467	0.0188	1.1E-104	7213	-0.003	0.2436	0.004	0.5664	2E+06	MANEA	aric	0.0634	487.7
rs9403054	G	T	-0.294	0.3557	0.0170	1.0E-65	7213	0.002	0.3571	0.004	0.6933	2E+06	MANEA	aric	0.0398	299.2
rs17753556	A	C	-0.247	0.1892	0.0212	3.4E-31	7213	0.001	0.1813	0.005	0.9036	2E+06	SERPINA3	aric	0.0185	136.2
rs17783344	G	T	-0.592	0.1330	0.0236	9.7E-134	7213	0.002	0.1362	0.006	0.6975	2E+06	GCA	aric	0.0805	631.7
rs17850756	A	G	-0.477	0.3221	0.0170	1.1E-164	7213	0.021	0.3169	0.004	0.0000	2E+06	QPCTL	aric	0.0985	788.0
rs1799886	C	T	0.390	0.4303	0.0162	1.7E-123	7213	0.007	0.433	0.004	0.0711	2E+06	PRSS2	aric	0.0745	580.7
rs1800493	T	C	-1.324	0.0143	0.0688	1.5E-80	7213	0.036	0.0144	0.016	0.0248	2E+06	LRPAP1	aric	0.0489	370.4
rs1838343	T	C	-0.262	0.4335	0.0163	6.2E-57	7213	-0.012	0.437	0.004	0.0017	2E+06	CNTN1	aric	0.0344	257.3
rs4548696	C	A	-0.355	0.1210	0.0251	7.3E-45	7213	0.003	0.1317	0.006	0.6290	2E+06	CNTN1	aric	0.0270	200.2
rs1846934	T	C	0.185	0.4619	0.0166	1.2E-28	7213	0.003	0.4667	0.004	0.4922	2E+06	CD33	aric	0.0169	124.3
rs2459147	A	G	0.232	0.4639	0.0166	6.4E-44	7213	0.013	0.4548	0.004	0.0003	2E+06	CD33	aric	0.0264	195.8
rs18643358[illegible]	G	A	0.384	0.0147	0.0692	3.1E-08	7213	-0.004	0.019	0.015	0.8012	2E+06	GSTZ1	aric	0.0042	30.7
rs755072	G	A	0.768	0.0232	0.0543	1.0E-44	7213	-0.003	0.0238	0.012	0.7895	2E+06	GSTZ1	aric	0.0269	199.5
rs78371502	T	C	-0.293	0.0506	0.0375	5.5E-15	7213	-0.003	0.0582	0.008	0.6832	2E+06	GSTZ1	aric	0.0084	61.3
rs1869085	C	A	-0.123	0.3076	0.0179	7.8E-12	7213	-0.004	0.3179	0.004	0.2690	2E+06	STIM1	aric	0.0065	47.0
rs18874562[illegible]	G	T	0.474	0.0414	0.0415	6.0E-30	7213	-0.011	0.0381	0.01	0.2542	2E+06	B4GALT7	aric	0.0178	130.4

s18890427	A	G	-1.491	0.0107	0.0787	4.4E-78	7213	-0.010	0.0124	0.019	0.6057	2E+06	TREM2	aric	0.0474	358.5
rs2181205	A	G	-0.834	0.0310	0.0469	2.7E-69	7213	-0.021	0.0315	0.011	0.0568	2E+06	SFTPD	aric	0.0420	316.2
rs4253536	C	T	-0.387	0.0677	0.0325	1.8E-32	7213	-0.008	0.0751	0.007	0.2622	2E+06	SFTPD	aric	0.0193	142.2
rs34231058	A	G	-0.763	0.0474	0.0378	3.2E-88	7213	0.012	0.0441	0.009	0.1885	2E+06	CFHR1	aric	0.0535	407.5
rs35449482	C	A	-0.809	0.0192	0.0601	6.8E-41	7213	0.003	0.0197	0.014	0.8261	2E+06	CFHR1	aric	0.0246	181.5
rs1926318	T	C	0.310	0.2165	0.0200	4.3E-53	7213	-0.013	0.2233	0.004	0.0043	2E+06	DCLK1	aric	0.0321	239.1
s19292314	T	G	-0.398	0.0168	0.0649	9.6E-10	7213	0.014	0.0155	0.015	0.3507	2E+06	GFRA2	aric	0.0052	37.5
rs1944270	A	G	0.131	0.2891	0.0183	1.0E-12	7213	-0.002	0.2977	0.004	0.5518	2E+06	SERPINB8	aric	0.0070	51.0
rs3826616	A	G	0.428	0.4258	0.0160	4.3E-150	7213	-0.005	0.4351	0.004	0.1923	2E+06	SERPINB8	aric	0.0901	713.9
rs56321661	A	C	-0.230	0.2047	0.0204	3.5E-29	7213	0.005	0.202	0.005	0.2615	2E+06	SERPINB8	aric	0.0173	126.8
rs1980606	A	G	-0.426	0.2024	0.0202	1.1E-95	7213	0.002	0.2033	0.005	0.6434	2E+06	CD48	aric	0.0580	443.9
rs6427541	T	C	0.142	0.2933	0.0181	5.7E-15	7213	-0.004	0.2947	0.004	0.3238	2E+06	CD48	aric	0.0084	61.3
rs198379	C	T	0.205	0.4240	0.0169	1.5E-33	7213	-0.009	0.4056	0.004	0.0130	2E+06	NPPB	aric	0.0200	147.2
rs2020854	C	T	-0.588	0.0704	0.0318	9.1E-75	7213	-0.010	0.0707	0.007	0.1630	2E+06	APOF	aric	0.0453	342.5
rs2069398	A	G	0.449	0.0783	0.0309	3.0E-47	7213	-0.001	0.0772	0.007	0.8596	2E+06	PMEL	aric	0.0285	211.4
rs2072563	A	G	-0.214	0.3162	0.0178	6.7E-33	7213	0.002	0.3033	0.004	0.6845	2E+06	PGLYRP1	aric	0.0196	144.1
rs2179795	T	G	-0.253	0.2824	0.0183	3.4E-43	7213	-0.003	0.2888	0.004	0.4931	2E+06	PTPRU	aric	0.0260	192.3
rs2205771	A	G	0.428	0.1511	0.0229	3.4E-76	7213	-0.003	0.1797	0.005	0.5952	2E+06	KREMEN1	aric	0.0462	349.4
rs2229475	T	C	0.728	0.0590	0.0345	5.0E-96	7213	-0.006	0.0576	0.008	0.4338	2E+06	HSPG2	aric	0.0582	445.5
rs45589832	A	G	-1.050	0.0239	0.0521	8.0E-88	7213	-0.001	0.0278	0.012	0.9659	2E+06	LBP	aric	0.0532	405.6
rs73095812	T	C	-0.945	0.0161	0.0652	5.4E-47	7213	-0.002	0.0205	0.014	0.8912	2E+06	LBP	aric	0.0283	210.3
rs2241044	C	A	-0.280	0.5010	0.0162	1.8E-65	7213	0.001	0.5009	0.004	0.8350	2E+06	IL17RA	aric	0.0397	298.0
rs7287672	A	G	0.192	0.3124	0.0178	7.6E-27	7213	-0.002	0.2984	0.004	0.5650	2E+06	IL17RA	aric	0.0158	116.0
rs2255703	C	T	-0.345	0.3813	0.0167	1.1E-91	7213	-0.021	0.3788	0.004	0.0000	2E+06	PLXND1	aric	0.0556	424.3
rs28850104	T	C	-0.111	0.2773	0.0184	1.8E-09	7213	-0.007	0.2856	0.004	0.0921	2E+06	PLXND1	aric	0.0050	36.3
rs2273185	A	C	-0.594	0.3287	0.0163	3.6E-267	7213	0.003	0.3304	0.004	0.5063	2E+06	ATXN3	aric	0.1556	1328.4
rs2277382	T	C	0.501	0.0862	0.0289	5.5E-66	7213	-0.001	0.0866	0.007	0.8747	2E+06	ACVRL1	aric	0.0400	300.4
rs2284031	C	T	-0.381	0.4529	0.0160	1.5E-120	7213	0.000	0.4696	0.004	0.9218	2E+06	CSF2RB	aric	0.0728	566.1
rs77818863	G	A	0.347	0.0363	0.0442	4.8E-15	7213	0.012	0.0377	0.01	0.2234	2E+06	CSF2RB	aric	0.0085	61.6
rs2292428	C	T	-0.327	0.3970	0.0164	3.4E-86	7213	0.000	0.3962	0.004	0.9928	2E+06	KLKB1	aric	0.0523	397.7
rs2298087	C	T	-0.840	0.1003	0.0261	7.8E-212	7213	0.004	0.0905	0.007	0.5297	2E+06	ESD	aric	0.1252	1032.2
rs2302524	C	T	-0.415	0.1625	0.0217	1.0E-79	7213	0.005	0.159	0.005	0.3341	2E+06	PLAUR	aric	0.0483	366.3

rs4251824	T	C	-0.465	0.0389	0.0428	2.7E-27	7213	0.022	0.036	0.01	0.0331	2E+06	PLAUR	aric	0.0161	118.0
rs2304850	T	C	-0.375	0.2516	0.0186	2.9E-88	7213	0.004	0.2381	0.004	0.3960	2E+06	GAA	aric	0.0535	407.7
rs77319086	A	C	-0.179	0.0710	0.0322	2.8E-08	7213	0.003	0.0734	0.007	0.6660	2E+06	GAA	aric	0.0043	30.9
rs2304969	T	G	-0.264	0.1355	0.0241	1.5E-27	7213	-0.002	0.1701	0.005	0.7089	2E+06	GLTPD2	aric	0.0163	119.3
rs2305948	T	C	0.527	0.0991	0.0273	5.1E-81	7213	-0.001	0.0975	0.006	0.8719	2E+06	KDR	aric	0.0491	372.7
rs34231037	G	A	-1.112	0.0308	0.0466	3.2E-121	7213	-0.005	0.0322	0.011	0.6253	2E+06	KDR	aric	0.0732	569.4
rs2307050	A	G	-0.281	0.1095	0.0264	3.7E-26	7213	0.004	0.1046	0.006	0.5669	2E+06	PDGFRA	aric	0.0154	112.8
rs2311597	G	A	-0.338	0.4185	0.0164	1.7E-91	7213	-0.014	0.4106	0.004	0.0002	2E+06	INHBB	aric	0.0555	423.4
rs2370794	G	A	0.308	0.3271	0.0176	2.7E-67	7213	0.002	0.3417	0.004	0.6340	2E+06	CRTAM	aric	0.0408	306.7
rs7925381	C	T	0.091	0.4410	0.0166	4.9E-08	7213	0.002	0.4466	0.004	0.6886	2E+06	CRTAM	aric	0.0041	29.8
rs2383984	A	G	-0.409	0.2634	0.0184	5.0E-106	7213	-0.010	0.2617	0.004	0.0164	2E+06	NRP1	aric	0.0642	494.4
rs2434484	G	T	-0.201	0.3367	0.0174	1.9E-30	7213	-0.008	0.329	0.004	0.0399	2E+06	ALKBH3	aric	0.0181	132.7
rs7833351	G	A	0.327	0.3254	0.0173	9.7E-78	7213	-0.005	0.3274	0.004	0.2444	2E+06	CDH17	aric	0.0472	356.8
rs246392	T	C	-0.467	0.3119	0.0170	1.5E-157	7213	0.000	0.2989	0.004	0.9495	2E+06	PDGFRB	aric	0.0944	751.7
rs72832182	T	C	-0.745	0.0950	0.0270	5.7E-159	7213	0.015	0.0969	0.006	0.0184	2E+06	PDGFRB	aric	0.0952	758.9
rs2526256	C	T	0.199	0.4098	0.0167	1.3E-32	7213	0.002	0.3965	0.004	0.5695	2E+06	ISOC1	aric	0.0194	142.8
rs2569441	T	C	-0.137	0.3944	0.0170	9.0E-16	7213	-0.001	0.3824	0.004	0.7579	2E+06	KLK14	aric	0.0089	64.9
rs34093024	C	T	-0.318	0.0976	0.0279	7.2E-30	7213	0.008	0.1084	0.006	0.1661	2E+06	KLK14	aric	0.0177	130.0
rs2589775	G	A	-0.198	0.2269	0.0198	1.8E-23	7213	0.001	0.218	0.005	0.8483	2E+06	EGFLAM	aric	0.0137	100.3
rs1320040	T	C	0.126	0.2149	0.0204	6.7E-10	7213	-0.004	0.2171	0.005	0.4439	2E+06	CD300A	aric	0.0053	38.2
rs2706525	A	G	-0.749	0.1695	0.0200	4.1E-280	7213	-0.006	0.1895	0.005	0.1973	2E+06	CD300A	aric	0.1625	1399.2
rs2711897	T	C	0.265	0.3817	0.0168	6.5E-55	7213	-0.029	0.3896	0.004	0.0000	2E+06	BDH2	aric	0.0332	247.7
rs28444377	C	A	0.119	0.2319	0.0195	1.1E-09	7213	-0.011	0.2252	0.004	0.0110	2E+06	BDH2	aric	0.0051	37.3
rs2712355	C	T	-0.249	0.4579	0.0164	3.5E-51	7213	-0.002	0.4514	0.004	0.6055	2E+06	GRAMD1C	aric	0.0309	230.0
rs2702884	T	C	0.131	0.3655	0.0171	2.4E-14	7213	-0.004	0.3739	0.004	0.3173	2E+06	DEFB1	aric	0.0080	58.4
rs2741108	T	C	0.356	0.4822	0.0162	6.2E-104	7213	0.003	0.4725	0.004	0.4750	2E+06	DEFB1	aric	0.0629	484.1
rs2741712	C	A	-0.125	0.2622	0.0189	4.1E-11	7213	0.003	0.2637	0.004	0.5160	2E+06	DEFB1	aric	0.0060	43.7
rs2755171	G	T	0.163	0.2501	0.0189	8.3E-18	7213	0.005	0.2423	0.004	0.2794	2E+06	CAT	aric	0.0102	74.2
rs769218	A	G	-0.347	0.2291	0.0195	8.4E-70	7213	0.005	0.2207	0.004	0.3044	2E+06	CAT	aric	0.0423	318.7
rs2763257	A	G	0.386	0.3936	0.0163	2.1E-119	7213	0.005	0.412	0.004	0.1742	2E+06	SMOC2	aric	0.0721	560.3
rs58602032	T	C	0.164	0.1580	0.0227	5.4E-13	7213	-0.010	0.1592	0.006	0.0675	1E+06	SMOC2	aric	0.0072	52.2
rs59291571	A	G	-0.452	0.0569	0.0356	1.7E-36	7213	0.007	0.064	0.008	0.3540	2E+06	SMOC2	aric	0.0218	160.9

rs2769265	A	C	-0.478	0.1887	0.0204	1.1E-116	7213	0.017	0.1802	0.005	0.0004	2E+06	PSMB4	aric	0.0705	546.9
rs2838952	T	C	-0.302	0.0239	0.0537	2.1E-08	7213	-0.002	0.0327	0.011	0.8838	2E+06	COL6A1	aric	0.0043	31.5
rs34627227	T	C	0.375	0.3751	0.0165	8.3E-111	7213	0.004	0.3794	0.004	0.3043	2E+06	COL6A1	aric	0.0670	517.9
rs28458356	A	G	-0.635	0.1041	0.0263	3.3E-124	7213	0.018	0.1052	0.006	0.0030	2E+06	ENTPD5	aric	0.0749	584.2
rs28689705	T	G	0.149	0.1976	0.0208	9.3E-13	7213	0.000	0.1969	0.005	0.9884	2E+06	SHANK3	aric	0.0070	51.2
rs9616896	C	T	0.425	0.2073	0.0200	1.6E-97	7213	-0.002	0.217	0.005	0.7460	2E+06	SHANK3	aric	0.0591	452.8
rs28721898	T	C	-0.501	0.1982	0.0202	1.5E-130	7213	0.000	0.2049	0.005	0.9623	2E+06	FUT3	aric	0.0787	615.8
rs300275	A	G	-0.111	0.4897	0.0166	2.4E-11	7213	0.008	0.4833	0.004	0.0371	2E+06	FAM151A	aric	0.0062	44.7
rs7545297	A	G	0.294	0.3488	0.0170	4.2E-66	7213	-0.003	0.3548	0.004	0.4023	2E+06	FAM151A	aric	0.0401	301.0
rs300996	T	G	0.555	0.4093	0.0159	2.1E-246	7213	0.002	0.4106	0.004	0.6422	2E+06	CHMP2B	aric	0.1443	1216.0
rs3024798	T	G	-0.350	0.3742	0.0168	3.4E-93	7213	0.003	0.3609	0.004	0.4203	2E+06	GNLY	aric	0.0565	431.7
rs307803	C	T	0.115	0.4039	0.0170	1.8E-11	7213	0.006	0.3913	0.004	0.1495	2E+06	SCGB3A1	aric	0.0062	45.3
rs320682	C	T	0.288	0.4231	0.0166	5.6E-66	7213	-0.016	0.4297	0.004	0.0000	2E+06	PTN	aric	0.0400	300.4
rs62490463	C	T	-0.310	0.0222	0.0561	3.4E-08	7213	0.020	0.036	0.011	0.0733	2E+06	PTN	aric	0.0042	30.5
rs3213120	T	C	-1.160	0.0302	0.0472	4.2E-128	7213	0.016	0.0308	0.011	0.1400	2E+06	IL12B	aric	0.0772	603.6
rs3773233	T	C	0.340	0.1982	0.0206	4.5E-60	7213	0.011	0.2074	0.005	0.0163	2E+06	ROBO1	aric	0.0364	272.2
rs34000233	A	G	0.332	0.1993	0.0207	3.6E-57	7213	0.003	0.1956	0.005	0.4685	2E+06	TDGF1	aric	0.0346	258.4
rs34012279	T	G	-0.174	0.2075	0.0200	5.1E-18	7213	0.003	0.2005	0.005	0.5062	2E+06	B3GNT2	aric	0.0103	75.2
rs4073090	G	A	0.226	0.4199	0.0166	9.6E-42	7213	0.017	0.4254	0.004	0.0000	2E+06	B3GNT2	aric	0.0251	185.5
rs34015250	G	A	-0.444	0.0903	0.0289	3.0E-52	7213	0.008	0.0886	0.007	0.2270	2E+06	AP1G2	aric	0.0316	235.1
rs340829	G	T	0.177	0.4067	0.0168	6.1E-26	7213	0.001	0.426	0.004	0.8816	2E+06	IL5RA	aric	0.0153	111.8
rs7635810	T	C	0.324	0.3499	0.0169	1.2E-79	7213	-0.001	0.3374	0.004	0.8770	2E+06	IL5RA	aric	0.0483	366.1
rs9831674	A	G	-0.234	0.2107	0.0201	5.8E-31	7213	-0.003	0.2083	0.005	0.5376	2E+06	IL5RA	aric	0.0184	135.1
rs34324219	A	C	-0.571	0.1064	0.0262	4.7E-102	7213	0.004	0.1122	0.006	0.4894	2E+06	TCN1	aric	0.0618	474.9
rs34393987	T	C	0.738	0.0206	0.0576	3.6E-37	7213	-0.005	0.0194	0.014	0.6976	2E+06	SPINK2	aric	0.0223	164.1
rs11911765	T	C	-0.246	0.4901	0.0164	4.1E-50	7213	-0.004	0.4771	0.004	0.2768	2E+06	NCAM2	aric	0.0303	225.0
rs7736104	T	G	0.528	0.4006	0.0158	1.8E-226	7213	-0.001	0.3931	0.004	0.7767	2E+06	CRHBP	aric	0.1333	1109.5
rs34497316	A	G	-0.233	0.1292	0.0248	8.2E-21	7213	-0.007	0.1313	0.006	0.2312	2E+06	C1QL1	aric	0.0121	88.1
rs7225162	C	T	0.328	0.4766	0.0160	1.6E-90	7213	0.000	0.4814	0.004	0.9261	2E+06	C1QL1	aric	0.0549	418.6
rs34511054	C	A	-0.209	0.0542	0.0371	1.8E-08	7213	-0.017	0.057	0.008	0.0378	2E+06	PDCD6	aric	0.0044	31.8
rs34928277	T	C	0.112	0.2893	0.0183	8.9E-10	7213	-0.008	0.2952	0.004	0.0586	2E+06	TLR3	aric	0.0052	37.6
rs34668207	A	G	0.412	0.1013	0.0269	7.3E-52	7213	0.004	0.0986	0.006	0.5451	2E+06	VWA2	aric	0.0313	233.2

rs80113039	A	G	-0.399	0.0336	0.0460	4.8E-18	7213	0.013	0.0306	0.011	0.2296	2E+06	VWA2	aric	0.0103	75.3
rs34695217	C	T	-0.721	0.0410	0.0413	6.5E-67	7213	-0.029	0.048	0.009	0.0015	2E+06	CHRDL2	aric	0.0406	304.8
rs6592590	T	C	0.166	0.4057	0.0168	7.7E-23	7213	0.005	0.4079	0.004	0.2285	2E+06	CHRDL2	aric	0.0133	97.4
rs34712979	A	G	-0.387	0.2452	0.0188	5.2E-92	7213	-0.013	0.2522	0.004	0.0034	2E+06	NPNT	aric	0.0558	425.9
rs34933869	C	T	0.472	0.1492	0.0228	1.1E-92	7213	-0.009	0.1438	0.005	0.0837	2E+06	CD8A	aric	0.0562	429.1
rs3014824	A	G	0.309	0.2600	0.0183	2.3E-62	7213	0.002	0.2611	0.004	0.7289	2E+06	S100A7	aric	0.0378	283.2
rs34961571	G	T	0.531	0.0772	0.0306	4.4E-66	7213	0.007	0.0737	0.007	0.3496	2E+06	S100A7	aric	0.0401	300.9
rs12052464	T	G	0.129	0.4514	0.0167	1.2E-14	7213	-0.005	0.4751	0.004	0.2209	2E+06	DNER	aric	0.0082	59.9
rs35032874	G	T	-0.160	0.2965	0.0181	1.5E-18	7213	-0.004	0.3093	0.004	0.3546	2E+06	DNER	aric	0.0106	77.6
rs35067598	G	A	-0.344	0.0544	0.0368	1.2E-20	7213	-0.006	0.0561	0.008	0.4802	2E+06	ALCAM	aric	0.0120	87.3
rs9834384	C	T	-0.371	0.0311	0.0477	8.2E-15	7213	-0.015	0.032	0.011	0.1742	2E+06	ALCAM	aric	0.0083	60.5
rs35220837	A	G	0.637	0.0462	0.0391	1.1E-58	7213	0.001	0.051	0.008	0.9193	2E+06	CLSTN2	aric	0.0355	265.6
rs4073898	G	T	0.134	0.3061	0.0180	8.4E-14	7213	-0.006	0.2922	0.004	0.1732	2E+06	CLSTN2	aric	0.0077	55.9
rs35383686	A	G	-0.209	0.2381	0.0195	1.1E-26	7213	-0.009	0.2266	0.005	0.0412	2E+06	CXCL16	aric	0.0157	115.3
rs35388278	G	T	-0.147	0.3334	0.0175	6.3E-17	7213	0.001	0.3354	0.004	0.8551	2E+06	RARRES1	aric	0.0096	70.2
rs35692207	G	A	0.136	0.4750	0.0164	1.5E-16	7213	0.000	0.4568	0.004	0.9526	2E+06	SIGLEC5	aric	0.0094	68.5
rs3829649	G	A	0.121	0.4626	0.0168	5.2E-13	7213	-0.002	0.4599	0.004	0.6608	2E+06	SIGLEC5	aric	0.0072	52.3
rs35791045	G	A	0.579	0.1709	0.0209	6.7E-160	7213	-0.001	0.1618	0.005	0.8875	2E+06	PSAPL1	aric	0.0958	763.6
rs62277606	A	G	-0.123	0.4330	0.0168	2.5E-13	7213	-0.001	0.4374	0.004	0.8833	2E+06	PSAPL1	aric	0.0074	53.8
rs35822882	T	G	-1.271	0.0234	0.0531	1.0E-121	7213	-0.005	0.0258	0.012	0.7158	2E+06	CLIC5	aric	0.0735	571.9
rs36043533	G	T	0.760	0.0510	0.0367	1.7E-92	7213	-0.002	0.0524	0.008	0.7756	2E+06	RSPO1	aric	0.0561	428.2
rs1667515	G	A	0.121	0.3721	0.0171	1.8E-12	7213	0.001	0.358	0.004	0.7743	2E+06	FLRT2	aric	0.0069	49.8
rs2747001	T	G	-0.306	0.1410	0.0239	2.7E-37	7213	0.006	0.1578	0.005	0.2807	2E+06	FLRT2	aric	0.0223	164.7
rs36101989	T	G	-0.537	0.2860	0.0172	1.1E-199	7213	-0.007	0.2753	0.004	0.0904	2E+06	FLRT2	aric	0.1184	968.6
rs36187	G	A	0.354	0.4661	0.0162	2.2E-102	7213	-0.007	0.4606	0.004	0.0679	2E+06	EPHB1	aric	0.0620	476.5
rs57512309	T	G	0.149	0.2515	0.0190	5.1E-15	7213	-0.001	0.2609	0.004	0.8149	2E+06	EPHB1	aric	0.0085	61.5
rs73229138	G	A	0.258	0.0535	0.0373	5.2E-12	7213	-0.008	0.0608	0.008	0.3233	2E+06	EPHB1	aric	0.0066	47.8
rs79903267	C	T	-1.189	0.0209	0.0567	6.6E-95	7213	0.004	0.0253	0.012	0.7543	2E+06	SEMA4D	aric	0.0575	440.0
rs3733897	G	A	0.472	0.1327	0.0237	4.3E-86	7213	0.019	0.1432	0.005	0.0004	2E+06	TXNDC15	aric	0.0522	397.2
rs3739613	A	G	-0.317	0.3038	0.0177	2.9E-70	7213	-0.007	0.2946	0.004	0.0824	2E+06	GLIPR2	aric	0.0426	320.9
rs72729437	G	A	0.263	0.0371	0.0439	2.4E-09	7213	-0.003	0.0344	0.01	0.7754	2E+06	GLIPR2	aric	0.0049	35.7
rs3743268	A	G	-0.204	0.3341	0.0174	2.0E-31	7213	0.003	0.347	0.004	0.4779	2E+06	ANXA2	aric	0.0187	137.2

rs375396	A	C	0.429	0.1842	0.0207	5.1E-93	7213	0.001	0.1972	0.005	0.7653	2E+06	GM2A	aric	0.0564	430.8
rs72794132	T	C	0.706	0.1658	0.0209	2.1E-233	7213	-0.012	0.1647	0.005	0.0144	2E+06	GM2A	aric	0.1372	1146.4
rs62350309	G	A	-0.235	0.0542	0.0370	2.5E-10	7213	0.003	0.0672	0.008	0.7147	2E+06	F11	aric	0.0055	40.1
rs3762423	C	T	-0.964	0.0511	0.0361	6.9E-150	7213	0.023	0.0509	0.009	0.0079	2E+06	B4GALT2	aric	0.0900	712.9
rs3772197	G	A	-0.535	0.0737	0.0315	1.5E-63	7213	-0.006	0.0755	0.007	0.4419	2E+06	LRIG1	aric	0.0385	288.8
rs77048548	G	A	0.288	0.0313	0.0478	1.8E-09	7213	0.006	0.0329	0.011	0.5847	2E+06	LRIG1	aric	0.0050	36.3
rs3782676	T	C	-0.593	0.0723	0.0314	5.6E-78	7213	0.002	0.0726	0.007	0.8316	2E+06	PZP	aric	0.0473	358.0
rs3825259	G	A	0.631	0.1184	0.0246	6.7E-139	7213	0.006	0.1168	0.006	0.3396	2E+06	MANSC1	aric	0.0836	657.6
rs12462691	T	C	-0.095	0.3493	0.0173	4.0E-08	7213	-0.008	0.343	0.004	0.0422	2E+06	SIGLEC14	aric	0.0042	30.2
rs2864138	G	A	0.098	0.4567	0.0165	3.5E-09	7213	0.005	0.4482	0.004	0.2311	2E+06	SIGLEC14	aric	0.0048	35.0
rs136148	C	T	-0.425	0.3155	0.0174	9.4E-127	7213	0.003	0.3135	0.004	0.5072	2E+06	APOL1	aric	0.0764	596.9
rs3886200	T	C	0.104	0.4568	0.0168	5.1E-10	7213	0.003	0.4614	0.004	0.5171	2E+06	APOL1	aric	0.0053	38.8
rs61048056	A	G	-0.327	0.4511	0.0162	8.9E-88	7213	0.005	0.4707	0.004	0.1892	2E+06	RGMB	aric	0.0532	405.3
rs41277305	C	A	0.371	0.0334	0.0456	4.7E-16	7213	0.007	0.0356	0.011	0.5201	2E+06	DDT	aric	0.0091	66.2
rs6986061	T	C	-0.400	0.4075	0.0164	2.1E-126	7213	-0.021	0.4139	0.004	0.0000	2E+06	FGL1	aric	0.0762	595.1
rs4296866	T	C	-0.210	0.4579	0.0165	1.0E-36	7213	-0.007	0.4512	0.004	0.0708	2E+06	NT5E	aric	0.0220	161.9
rs632350	T	C	-0.602	0.1212	0.0245	2.0E-128	7213	-0.004	0.1152	0.006	0.5406	2E+06	NT5E	aric	0.0774	605.2
rs433373	G	A	0.240	0.1284	0.0247	3.2E-22	7213	-0.001	0.1223	0.006	0.8385	2E+06	PLEKHA7	aric	0.0129	94.5
rs436075	C	T	0.165	0.4108	0.0168	8.1E-23	7213	0.009	0.4359	0.004	0.0258	2E+06	DECR2	aric	0.0133	97.3
rs4384764	A	G	0.160	0.2636	0.0189	2.2E-17	7213	0.003	0.261	0.004	0.5114	2E+06	QPCT	aric	0.0099	72.3
rs4396423	T	C	0.099	0.3777	0.0170	6.6E-09	7213	0.001	0.38	0.004	0.8899	2E+06	PCDH9	aric	0.0047	33.7
rs4457535	G	A	-0.346	0.3870	0.0166	2.0E-94	7213	0.001	0.3836	0.004	0.7138	2E+06	ANGPTL1	aric	0.0572	437.6
rs4459759	A	G	0.460	0.0593	0.0349	3.4E-39	7213	-0.003	0.0575	0.008	0.6741	2E+06	REG3A	aric	0.0235	173.6
rs4464946	A	G	-0.312	0.2393	0.0192	1.1E-58	7213	-0.001	0.245	0.004	0.7777	2E+06	RMDN1	aric	0.0355	265.5
rs4961042	T	G	0.210	0.4540	0.0166	4.6E-36	7213	0.006	0.4681	0.004	0.1371	2E+06	RMDN1	aric	0.0216	158.9
rs4468199	G	A	-0.340	0.0933	0.0284	7.4E-33	7213	-0.018	0.0883	0.007	0.0072	2E+06	LEPR	aric	0.0196	143.9
rs6678033	A	G	-0.633	0.3800	0.0154	0.0E+00	7213	-0.007	0.3848	0.004	0.0731	2E+06	LEPR	aric	0.1903	1694.5
rs45583840	A	G	-0.512	0.0794	0.0300	4.6E-64	7213	0.005	0.0789	0.007	0.5058	2E+06	TMEM190	aric	0.0388	291.2
rs79012440	A	G	-0.322	0.2937	0.0179	2.0E-70	7213	-0.001	0.3031	0.004	0.7698	2E+06	TMEM190	aric	0.0427	321.7
rs4575	C	T	0.295	0.2846	0.0179	6.7E-60	7213	0.002	0.2867	0.004	0.6402	2E+06	PSME2	aric	0.0363	271.4
rs4610468	C	T	-0.345	0.2651	0.0185	4.8E-76	7213	0.016	0.2664	0.004	0.0003	2E+06	GHR	aric	0.0461	348.7
rs4619875	T	C	0.425	0.3930	0.0162	5.6E-145	7213	-0.002	0.4116	0.004	0.6719	2E+06	ARFIP1	aric	0.0871	688.1

rs4661012	G	T	-0.302	0.3545	0.0170	3.9E-69	7213	-0.001	0.3628	0.004	0.8440	2E+06	PEAR1	aric	0.0419	315.5
rs4674836	G	A	-0.371	0.3777	0.0166	3.2E-107	7213	0.000	0.3913	0.004	0.9459	2E+06	SERPINE2	aric	0.0649	500.3
rs4682481	T	C	0.348	0.1668	0.0219	3.9E-56	7213	0.023	0.1961	0.005	0.0000	2E+06	BOC	aric	0.0340	253.5
rs10768174	G	A	0.274	0.4317	0.0164	1.4E-61	7213	0.002	0.4254	0.004	0.5625	2E+06	FJX1	aric	0.0373	279.5
rs474415	A	G	0.124	0.3215	0.0176	1.7E-12	7213	-0.004	0.3305	0.004	0.2769	2E+06	FJX1	aric	0.0069	50.0
rs476336	T	C	-0.312	0.3818	0.0169	6.0E-74	7213	0.003	0.3757	0.004	0.4373	2E+06	POMGNT2	aric	0.0448	338.6
rs4794183	A	G	0.468	0.2247	0.0192	6.9E-127	7213	-0.005	0.2234	0.004	0.2215	2E+06	WFIKKN2	aric	0.0765	597.5
rs62078063	C	T	1.143	0.0245	0.0520	1.1E-103	7213	-0.011	0.0299	0.012	0.3317	2E+06	CCL15	aric	0.0628	483.0
rs4802890	A	G	0.668	0.1132	0.0249	6.3E-151	7213	0.014	0.1123	0.006	0.0284	2E+06	ECH1	aric	0.0906	718.1
rs4807574	C	T	0.204	0.3445	0.0174	2.4E-31	7213	0.001	0.3395	0.004	0.7889	2E+06	EBI3	aric	0.0186	136.9
rs4894018	A	G	0.687	0.2005	0.0192	4.8E-259	7213	-0.001	0.1999	0.005	0.7889	2E+06	FKBP7	aric	0.1512	1284.2
rs79146658	C	T	-0.295	0.0864	0.0292	7.6E-24	7213	0.018	0.0876	0.007	0.0076	2E+06	FKBP7	aric	0.0140	102.1
rs6444134	C	T	-0.123	0.2887	0.0184	2.2E-11	7213	-0.010	0.3046	0.004	0.0113	2E+06	HRG	aric	0.0062	45.0
rs66965282	G	A	0.387	0.1515	0.0227	4.1E-64	7213	0.014	0.1432	0.005	0.0083	2E+06	HRG	aric	0.0389	291.5
rs55662831	A	G	0.105	0.3596	0.0171	8.9E-10	7213	-0.011	0.3592	0.004	0.0066	2E+06	SHBG	aric	0.0052	37.6
rs4848312	A	G	0.276	0.3126	0.0176	1.1E-54	7213	0.008	0.3104	0.004	0.0391	2E+06	IL1RN	aric	0.0331	246.7
rs55709272	C	T	-0.378	0.4404	0.0163	8.2E-115	7213	-0.004	0.4202	0.004	0.2416	2E+06	IL1RN	aric	0.0694	537.7
rs557382	G	T	0.216	0.2965	0.0180	4.4E-33	7213	0.008	0.2928	0.004	0.0466	2E+06	NLGN1	aric	0.0197	145.0
rs55986634	A	G	0.160	0.4278	0.0167	1.3E-21	7213	0.001	0.4127	0.004	0.7939	2E+06	DAPK2	aric	0.0126	91.8
rs56083715	T	C	0.382	0.2240	0.0194	3.0E-84	7213	0.011	0.2156	0.005	0.0201	2E+06	IDUA	aric	0.0511	388.2
rs73201446	A	G	0.469	0.0975	0.0276	1.0E-63	7213	-0.002	0.1075	0.006	0.7256	2E+06	CTSB	aric	0.0386	289.6
rs56336390	T	C	-0.511	0.0467	0.0391	1.7E-38	7213	-0.013	0.0483	0.009	0.1409	2E+06	NQO2	aric	0.0231	170.3
rs62391549	C	T	-0.573	0.0811	0.0296	1.4E-81	7213	-0.015	0.0864	0.007	0.0290	2E+06	NQO2	aric	0.0495	375.3
rs57362802	C	T	-0.957	0.0369	0.0430	2.6E-106	7213	-0.008	0.0349	0.01	0.4187	2E+06	ADGRF5	aric	0.0643	495.8
rs5848	T	C	-0.249	0.2816	0.0182	5.4E-42	7213	-0.010	0.2991	0.004	0.0219	2E+06	GRN	aric	0.0252	186.7
rs58509147	T	C	-1.072	0.0487	0.0367	2.2E-177	7213	-0.001	0.0464	0.009	0.8925	2E+06	A1BG	aric	0.1058	853.0
rs858257	C	T	-0.402	0.0406	0.0418	8.1E-22	7213	0.009	0.0438	0.01	0.3598	2E+06	GPNMB	aric	0.0127	92.7
rs59251421	T	C	-0.513	0.0777	0.0304	1.4E-62	7213	-0.018	0.0744	0.007	0.0121	2E+06	MSR1	aric	0.0379	284.2
rs59477943	G	A	-1.279	0.0383	0.0407	7.2E-203	7213	0.027	0.0384	0.01	0.0061	2E+06	B4GALT6	aric	0.1202	985.2
rs78796387	T	G	0.259	0.0343	0.0460	1.8E-08	7213	-0.010	0.0348	0.011	0.3372	2E+06	B4GALT6	aric	0.0044	31.8
rs6070664	T	G	-0.297	0.0310	0.0477	4.8E-10	7213	0.030	0.0351	0.01	0.0033	2E+06	CTSZ	aric	0.0054	38.8
rs6072300	T	C	0.219	0.1709	0.0220	2.9E-23	7213	0.030	0.1813	0.005	0.0000	2E+06	EMILIN3	aric	0.0136	99.4

rs78732698	A	C	-1.122	0.0133	0.0711	4.1E-55	7213	-0.007	0.0147	0.016	0.6696	2E+06	CPNE1	aric	0.0333	248.6
rs61240730	C	T	-0.237	0.1126	0.0263	2.8E-19	7213	0.013	0.1061	0.006	0.0363	2E+06	CR2	aric	0.0111	81.0
rs61765448	T	C	-0.649	0.1662	0.0210	4.2E-197	7213	-0.006	0.1892	0.005	0.2391	2E+06	ROR1	aric	0.1170	955.1
rs61803119	T	G	0.191	0.1090	0.0265	6.1E-13	7213	0.009	0.1075	0.006	0.1423	2E+06	S100A12	aric	0.0072	52.0
rs61806985	T	C	-0.683	0.0415	0.0409	2.0E-61	7213	0.002	0.0463	0.009	0.8174	2E+06	F5	aric	0.0372	278.6
rs61825157	T	C	1.326	0.0423	0.0381	8.0E-245	7213	-0.002	0.0485	0.009	0.7885	2E+06	GUK1	aric	0.1434	1207.5
rs61885329	A	G	0.320	0.0518	0.0373	1.2E-17	7213	-0.012	0.0504	0.009	0.1498	2E+06	FOLH1	aric	0.0101	73.5
rs7123666	A	G	-0.284	0.0988	0.0274	5.4E-25	7213	0.023	0.1089	0.006	0.0001	2E+06	APOA5	aric	0.0147	107.4
rs619833	A	G	-0.314	0.0352	0.0446	2.0E-12	7213	0.004	0.0359	0.01	0.6699	2E+06	KIRREL2	aric	0.0068	49.6
rs61993080	T	C	-0.185	0.0695	0.0326	1.3E-08	7213	0.003	0.0749	0.007	0.6846	2E+06	MDGA2	aric	0.0045	32.3
rs62037104	A	C	-0.466	0.0202	0.0585	1.9E-15	7213	0.019	0.0196	0.014	0.1636	2E+06	CCL22	aric	0.0087	63.5
rs9921051	A	G	0.346	0.0695	0.0324	1.9E-26	7213	-0.017	0.0703	0.007	0.0220	2E+06	CCL22	aric	0.0156	114.1
rs62087497	A	G	0.420	0.0721	0.0317	1.3E-39	7213	-0.005	0.0749	0.007	0.4568	2E+06	TWSG1	aric	0.0238	175.6
rs62115743	T	C	-1.053	0.0824	0.0279	1.3E-285	7213	0.007	0.0881	0.007	0.3049	2E+06	KLK11	aric	0.1654	1429.5
rs62162757	A	C	0.327	0.3127	0.0177	7.4E-75	7213	-0.001	0.3197	0.004	0.7571	2E+06	CAPG	aric	0.0454	343.0
rs950362	A	G	-1.793	0.0120	0.0731	7.9E-128	7213	0.005	0.0123	0.017	0.7561	2E+06	CAPG	aric	0.0771	602.2
rs62184386	T	C	0.590	0.4420	0.0152	3.0E-299	7213	-0.016	0.4491	0.004	0.0000	2E+06	HIBCH	aric	0.1727	1504.9
rs3804749	C	T	1.002	0.4140	0.0122	0.0E+00	7213	0.002	0.4068	0.004	0.6982	2E+06	PDIA5	aric	0.4823	6718.4
rs62263781	G	A	0.371	0.0177	0.0625	2.9E-09	7213	0.001	0.0175	0.015	0.9374	2E+06	PDIA5	aric	0.0049	35.3
rs6232	C	T	-0.759	0.0525	0.0361	2.4E-95	7213	0.018	0.0533	0.008	0.0308	2E+06	PCSK1	aric	0.0578	442.2
rs76239650	A	C	-0.541	0.0985	0.0271	2.7E-86	7213	-0.008	0.1052	0.006	0.2145	2E+06	PCSK1	aric	0.0523	398.2
rs62376423	A	G	-0.290	0.0295	0.0488	2.9E-09	7213	-0.008	0.031	0.011	0.4983	2E+06	ERAP2	aric	0.0049	35.3
rs7705528	G	A	-0.110	0.3623	0.0174	2.8E-10	7213	0.010	0.3799	0.004	0.0091	2E+06	ERAP2	aric	0.0055	39.9
rs7709763	C	T	-0.127	0.2050	0.0206	7.5E-10	7213	-0.003	0.2049	0.005	0.5216	2E+06	ERAP2	aric	0.0052	38.0
rs62642270	G	A	-0.175	0.1706	0.0221	2.7E-15	7213	0.001	0.16	0.005	0.8940	2E+06	SFRP1	aric	0.0086	62.8
rs72643819	T	G	-0.207	0.3902	0.0168	1.4E-34	7213	0.005	0.4002	0.004	0.1629	2E+06	SFRP1	aric	0.0206	152.0
rs62642596	T	C	0.279	0.4958	0.0163	1.0E-64	7213	0.009	0.4776	0.004	0.0152	2E+06	H6PD	aric	0.0392	294.4
rs641320	A	G	-0.727	0.0696	0.0315	1.1E-113	7213	0.009	0.0678	0.008	0.2150	2E+06	FAIM	aric	0.0687	532.0
rs6492108	C	A	0.095	0.3781	0.0171	2.9E-08	7213	-0.005	0.3668	0.004	0.2418	2E+06	TNFSF13B	aric	0.0043	30.9
rs6510263	G	A	0.677	0.2730	0.0168	0.0E+00	7213	-0.016	0.2707	0.004	0.0002	2E+06	PDCD5	aric	0.1845	1631.4
rs6600145	G	A	-0.232	0.0842	0.0298	6.7E-15	7213	-0.016	0.0841	0.007	0.0159	2E+06	GNPTG	aric	0.0084	60.9
rs6602909	C	T	-0.405	0.3223	0.0172	1.2E-118	7213	0.001	0.3333	0.004	0.7946	2E+06	GAS6	aric	0.0717	556.7

rs6671362	G	A	0.288	0.4746	0.0163	7.8E-68	7213	-0.002	0.4797	0.004	0.6190	2E+06	CREB3L4	aric	0.0411	309.3
rs6686906	A	G	0.157	0.3546	0.0171	7.1E-20	7213	0.001	0.3613	0.004	0.7985	2E+06	EPHB2	aric	0.0115	83.7
rs6699769	G	A	-0.348	0.2052	0.0200	9.6E-67	7213	0.002	0.2025	0.005	0.6903	2E+06	DNAJB4	aric	0.0405	304.0
rs2326055	C	T	-0.151	0.2123	0.0201	6.2E-14	7213	-0.003	0.205	0.005	0.5257	2E+06	CPXM1	aric	0.0078	56.5
rs67702963	C	T	-0.241	0.2356	0.0197	3.0E-34	7213	0.000	0.2345	0.004	0.9357	2E+06	MMP7	aric	0.0204	150.4
rs6918969	C	T	-0.203	0.2734	0.0186	1.7E-27	7213	0.017	0.2693	0.004	0.0000	2E+06	TREML2	aric	0.0162	119.0
rs991762	T	C	0.360	0.1928	0.0206	3.8E-67	7213	-0.010	0.1965	0.005	0.0247	2E+06	TREML2	aric	0.0407	306.0
rs7019909	T	C	0.631	0.1004	0.0264	4.1E-121	7213	-0.007	0.1042	0.006	0.2297	2E+06	B4GALT1	aric	0.0731	568.9
rs7116230	A	G	0.465	0.3845	0.0163	5.2E-169	7213	0.000	0.3767	0.004	0.9385	2E+06	SPON1	aric	0.1010	810.1
rs562672	C	T	-0.155	0.4155	0.0168	2.8E-20	7213	0.001	0.4112	0.004	0.8288	2E+06	CDON	aric	0.0117	85.6
rs657225	G	A	0.154	0.0968	0.0281	4.5E-08	7213	0.001	0.0983	0.006	0.8526	2E+06	CDON	aric	0.0041	30.0
rs7120521	T	C	-1.029	0.0859	0.0270	7.8E-289	7213	0.020	0.0908	0.007	0.0022	2E+06	CDON	aric	0.1671	1447.2
rs7122082	A	G	-0.100	0.4294	0.0169	3.8E-09	7213	0.002	0.4203	0.004	0.6379	2E+06	CYB5R2	aric	0.0048	34.8
rs75667685	A	G	0.301	0.0509	0.0378	1.8E-15	7213	0.001	0.0546	0.008	0.8779	2E+06	CYB5R2	aric	0.0087	63.5
rs7131073	G	A	0.101	0.3555	0.0173	5.8E-09	7213	-0.006	0.3428	0.004	0.1149	2E+06	CLMP	aric	0.0047	34.0
rs7946718	A	G	-0.431	0.4061	0.0160	3.8E-152	7213	-0.004	0.4069	0.004	0.3355	2E+06	CLMP	aric	0.0913	724.3
rs4528296	C	T	0.731	0.0718	0.0309	4.4E-119	7213	0.023	0.0774	0.007	0.0009	2E+06	CNTN5	aric	0.0719	558.8
rs7131217	G	A	0.114	0.2088	0.0204	2.6E-08	7213	0.005	0.2186	0.005	0.2703	2E+06	CNTN5	aric	0.0043	31.0
rs7135211	A	G	-0.427	0.3695	0.0165	1.1E-140	7213	-0.007	0.3595	0.004	0.0765	2E+06	MGP	aric	0.0846	666.6
rs7159420	G	T	0.611	0.1284	0.0235	5.0E-142	7213	0.002	0.1231	0.006	0.6943	2E+06	COCH	aric	0.0854	673.3
rs7204669	A	G	0.303	0.3194	0.0173	4.4E-67	7213	0.009	0.313	0.004	0.0244	2E+06	PRSS22	aric	0.0407	305.7
rs7247115	A	G	-0.281	0.2001	0.0206	1.2E-41	7213	0.013	0.1998	0.005	0.0052	2E+06	OLFM2	aric	0.0250	185.1
rs74178184	A	G	-0.292	0.2105	0.0203	3.6E-46	7213	-0.003	0.2161	0.005	0.5936	2E+06	OLFM2	aric	0.0278	206.4
rs72697218	T	C	-0.278	0.0320	0.0467	2.9E-09	7213	0.038	0.032	0.011	0.0005	2E+06	REG4	aric	0.0049	35.3
rs72704117	T	C	0.411	0.0195	0.0595	5.4E-12	7213	-0.005	0.0206	0.014	0.7041	2E+06	THBS3	aric	0.0066	47.7
rs72709664	A	C	-0.563	0.0882	0.0285	1.4E-84	7213	-0.034	0.0885	0.007	0.0000	2E+06	PRSS3	aric	0.0513	389.9
rs72712829	C	T	0.250	0.0627	0.0341	2.4E-13	7213	0.003	0.0737	0.008	0.7148	2E+06	LY9	aric	0.0074	53.8
rs72715776	T	G	-0.229	0.4306	0.0166	4.2E-43	7213	0.007	0.4225	0.004	0.0842	2E+06	PCDH10	aric	0.0259	191.9
rs72729191	C	T	0.906	0.0906	0.0267	4.3E-234	7213	-0.005	0.0867	0.007	0.4563	2E+06	SEMA5A	aric	0.1375	1150.0
rs2072528	T	C	0.452	0.3771	0.0164	9.7E-160	7213	0.001	0.3852	0.004	0.8271	2E+06	VIT	aric	0.0957	762.8
rs72824579	A	G	-0.797	0.0347	0.0446	7.4E-70	7213	-0.008	0.0338	0.011	0.4689	2E+06	MGAT4B	aric	0.0424	318.9
rs72835078	T	G	0.362	0.0790	0.0305	3.4E-32	7213	0.019	0.0828	0.007	0.0053	2E+06	GP1BA	aric	0.0192	140.8

rs72858937	T	C	-1.128	0.0213	0.0562	3.1E-87	7213	-0.003	0.0228	0.013	0.8377	2E+06	AFM	aric	0.0529	402.7
rs73191242	A	G	-0.355	0.1913	0.0210	3.1E-63	7213	0.004	0.2054	0.005	0.4210	2E+06	SELPLG	aric	0.0383	287.3
rs72988065	G	A	0.227	0.1626	0.0222	1.9E-24	7213	0.001	0.1676	0.005	0.7958	2E+06	GFRAL	aric	0.0143	104.9
rs12461895	A	C	-0.268	0.4031	0.0166	2.3E-57	7213	0.002	0.4205	0.004	0.5873	2E+06	B3GNT8	aric	0.0347	259.3
rs73045078	A	G	-0.384	0.0229	0.0556	5.9E-12	7213	-0.015	0.0312	0.012	0.2066	2E+06	B3GNT8	aric	0.0065	47.5
rs73047241	C	A	-0.575	0.0202	0.0586	1.4E-22	7213	-0.050	0.0253	0.013	0.0002	2E+06	B3GNT8	aric	0.0132	96.2
rs73202262	G	A	0.371	0.0360	0.0442	5.9E-17	7213	0.009	0.0319	0.011	0.4162	2E+06	CBR3	aric	0.0097	70.3
rs73217917	C	T	-0.606	0.0178	0.0628	7.0E-22	7213	0.020	0.0178	0.015	0.1711	2E+06	PEBP1	aric	0.0127	93.0
rs73287817	G	A	0.476	0.2613	0.0181	4.7E-146	7213	0.017	0.2482	0.004	0.0001	2E+06	CCDC126	aric	0.0877	693.5
rs73347621	T	C	-0.635	0.1423	0.0222	4.2E-170	7213	0.009	0.1332	0.006	0.1020	2E+06	VWC2	aric	0.1016	815.7
rs79523632	T	C	-0.449	0.0242	0.0536	6.4E-17	7213	0.038	0.026	0.012	0.0013	2E+06	VWC2	aric	0.0096	70.2
rs73407610	G	A	-0.536	0.0330	0.0462	6.9E-31	7213	-0.004	0.031	0.011	0.6943	2E+06	DEF6	aric	0.0183	134.7
rs73801515	T	C	-0.343	0.0357	0.0450	2.7E-14	7213	-0.008	0.0407	0.01	0.4325	2E+06	BST1	aric	0.0080	58.2
rs7409311	A	G	-0.522	0.0719	0.0314	6.9E-61	7213	0.007	0.0909	0.007	0.2962	2E+06	LHB	aric	0.0369	276.1
rs74578793	A	G	0.687	0.0439	0.0394	9.9E-67	7213	0.001	0.0493	0.009	0.9112	2E+06	GPC1	aric	0.0405	304.0
rs7459882	G	A	0.163	0.4910	0.0166	1.7E-22	7213	0.003	0.4876	0.004	0.3968	2E+06	GGH	aric	0.0131	95.9
rs77291996	C	T	0.776	0.0638	0.0332	1.3E-116	7213	-0.002	0.0719	0.007	0.8293	2E+06	GGH	aric	0.0705	546.6
rs74617719	C	T	-0.402	0.0460	0.0391	1.3E-24	7213	-0.008	0.0462	0.009	0.3526	2E+06	NAAA	aric	0.0144	105.6
rs75003668	G	A	-1.357	0.0333	0.0436	1.6E-199	7213	0.019	0.0326	0.011	0.0816	2E+06	APOH	aric	0.1183	967.7
rs9912634	T	C	0.479	0.0433	0.0406	7.6E-32	7213	0.008	0.0435	0.009	0.3749	2E+06	APOH	aric	0.0189	139.2
rs175111	C	T	-0.181	0.3991	0.0169	1.1E-26	7213	0.006	0.388	0.004	0.1011	2E+06	MEM132	aric	0.0157	115.2
rs75210230	G	T	-0.678	0.0165	0.0648	2.0E-25	7213	-0.018	0.015	0.016	0.2536	2E+06	MEM132	aric	0.0149	109.4
rs7525733	G	T	-0.793	0.2484	0.0167	0.0E+00	7213	-0.003	0.2518	0.004	0.4584	2E+06	UROD	aric	0.2374	2244.6
rs2795496	G	A	0.454	0.1646	0.0219	5.3E-93	7213	0.019	0.1677	0.005	0.0002	2E+06	RET	aric	0.0564	430.8
rs752978	T	C	0.189	0.4244	0.0167	1.6E-29	7213	-0.007	0.4065	0.004	0.0834	2E+06	RET	aric	0.0175	128.4
rs10918341	A	G	-0.120	0.3307	0.0176	1.3E-11	7213	-0.001	0.3295	0.004	0.8697	2E+06	FCGR3A	aric	0.0063	45.9
rs4657041	T	C	0.218	0.4976	0.0165	4.1E-39	7213	0.001	0.4882	0.004	0.6968	2E+06	FCGR3A	aric	0.0235	173.2
rs7549876	T	G	-0.245	0.3734	0.0169	2.3E-47	7213	-0.009	0.3664	0.004	0.0198	2E+06	TIE1	aric	0.0286	212.0
rs757537	G	A	0.401	0.1474	0.0231	4.4E-66	7213	-0.026	0.1383	0.005	0.0000	2E+06	LEAP2	aric	0.0401	300.9
rs75776028	G	A	-0.576	0.2754	0.0173	7.1E-226	7213	-0.005	0.2642	0.004	0.2583	2E+06	SELL	aric	0.1330	1106.3
rs75881014	A	G	-0.828	0.0119	0.0754	7.9E-28	7213	-0.044	0.0123	0.017	0.0114	2E+06	CA1	aric	0.0164	120.5
rs75904281	T	C	-0.686	0.0146	0.0694	6.7E-23	7213	0.014	0.0149	0.017	0.3944	2E+06	RSPO4	aric	0.0134	97.7

rs76038906	T	G	0.591	0.0381	0.0429	1.1E-42	7213	0.019	0.0344	0.011	0.0729	2E+06	SVEP1	aric	0.0257	190.0
rs78742138	C	T	0.976	0.0270	0.0501	1.8E-82	7213	0.048	0.0296	0.011	0.0000	2E+06	SVEP1	aric	0.0500	379.7
rs7607734	G	A	-0.799	0.1199	0.0236	1.2E-232	7213	0.001	0.1141	0.006	0.8374	2E+06	KYNU	aric	0.1367	1142.3
rs76143353	T	C	-1.032	0.0619	0.0322	1.3E-210	7213	0.008	0.0559	0.008	0.3124	2E+06	POFUT1	aric	0.1245	1025.8
rs76204064	T	C	0.668	0.0674	0.0323	1.2E-92	7213	-0.025	0.0658	0.008	0.0007	2E+06	FAM177A1	aric	0.0561	429.0
rs4756649	T	C	0.418	0.4175	0.0162	6.9E-140	7213	-0.004	0.4126	0.004	0.2956	2E+06	KIAA1549L	aric	0.0841	662.5
rs762044	C	T	-0.203	0.4305	0.0167	1.1E-33	7213	0.004	0.4347	0.004	0.3522	2E+06	KIAA1549L	aric	0.0201	147.8
rs7637064	C	T	-0.136	0.2356	0.0197	5.8E-12	7213	0.002	0.2286	0.004	0.7406	2E+06	RTP4	aric	0.0065	47.5
rs9865045	T	C	0.389	0.4948	0.0159	6.7E-127	7213	0.010	0.4974	0.004	0.0104	2E+06	RTP4	aric	0.0765	597.6
rs7674623	T	C	0.407	0.1947	0.0204	1.0E-86	7213	0.004	0.1941	0.005	0.3829	2E+06	ANTXR2	aric	0.0526	400.2
rs76790102	T	C	-0.796	0.0872	0.0278	4.4E-171	7213	0.004	0.0801	0.007	0.5727	2E+06	LGALS3	aric	0.1022	820.7
rs76857490	A	G	-0.540	0.0214	0.0568	2.6E-21	7213	0.013	0.0209	0.013	0.3129	2E+06	KLB	aric	0.0124	90.4
rs76985127	G	T	0.368	0.0212	0.0578	2.1E-10	7213	-0.018	0.0197	0.014	0.2083	2E+06	TEK	aric	0.0056	40.4
rs77688991	C	T	0.652	0.0191	0.0605	7.0E-27	7213	0.005	0.0307	0.012	0.6862	2E+06	TEK	aric	0.0159	116.1
rs77157727	A	G	-0.499	0.0305	0.0481	5.5E-25	7213	-0.008	0.032	0.011	0.4793	2E+06	SERPINB13	aric	0.0147	107.4
rs7730934	A	G	0.650	0.1200	0.0245	6.4E-148	7213	-0.002	0.1196	0.006	0.6837	2E+06	IL6ST	aric	0.0888	702.9
rs7739450	A	G	-0.179	0.4714	0.0167	1.7E-26	7213	-0.006	0.4781	0.004	0.1228	2E+06	VEGFA	aric	0.0156	114.4
rs77444140	A	G	-0.971	0.0184	0.0610	4.6E-56	7213	0.001	0.0175	0.015	0.9527	2E+06	C6	aric	0.0339	253.2
rs77515250	C	A	0.669	0.0165	0.0646	5.4E-25	7213	-0.005	0.0235	0.014	0.7055	2E+06	FUT8	aric	0.0147	107.4
rs4752926	C	T	0.343	0.3718	0.0169	3.1E-89	7213	0.008	0.3762	0.004	0.0400	2E+06	LRP4	aric	0.0541	412.5
rs77682558	A	G	0.313	0.0279	0.0504	5.3E-10	7213	-0.003	0.0309	0.012	0.8229	2E+06	LRP4	aric	0.0053	38.7
rs10183939	G	A	0.534	0.1918	0.0200	5.1E-149	7213	-0.010	0.2229	0.005	0.0380	2E+06	COLEC11	aric	0.0895	708.5
rs11123637	T	C	0.191	0.3632	0.0171	1.1E-28	7213	-0.011	0.3592	0.004	0.0073	2E+06	COLEC11	aric	0.0170	124.6
rs78111814	C	T	0.467	0.0460	0.0389	6.5E-33	7213	0.003	0.0494	0.009	0.7090	2E+06	DSCAM	aric	0.0196	144.2
rs7811214	A	G	-0.354	0.3307	0.0172	1.9E-91	7213	0.003	0.3395	0.004	0.4553	2E+06	TPST1	aric	0.0554	423.2
rs10905252	G	A	-0.115	0.3957	0.0169	1.2E-11	7213	-0.003	0.3907	0.004	0.4770	2E+06	ITIH2	aric	0.0064	46.2
rs78189039	A	G	0.358	0.0880	0.0291	1.8E-34	7213	-0.005	0.0855	0.007	0.4515	2E+06	ITIH2	aric	0.0206	151.5
rs10903015	C	A	0.110	0.4726	0.0166	3.3E-11	7213	0.009	0.4729	0.004	0.0145	2E+06	HBZ	aric	0.0061	44.1
rs78369087	A	G	-0.237	0.0426	0.0410	7.1E-09	7213	-0.035	0.0425	0.01	0.0003	2E+06	HBZ	aric	0.0046	33.6
rs78423067	A	G	-0.812	0.0134	0.0719	2.5E-29	7213	-0.014	0.0182	0.015	0.3575	2E+06	IL1R2	aric	0.0174	127.5
rs11103373	C	T	0.415	0.4762	0.0160	1.1E-141	7213	-0.003	0.4811	0.004	0.4394	2E+06	QSOX2	aric	0.0852	671.5
rs7849585	T	G	-0.139	0.3276	0.0179	8.1E-15	7213	0.003	0.3279	0.004	0.4979	2E+06	QSOX2	aric	0.0083	60.5

rs7857240	G	A	0.097	0.4572	0.0167	6.7E-09	7213	-0.018	0.4603	0.004	0.0000	2E+06	FBP1	aric	0.0046	33.7
rs4818855	C	T	0.113	0.4457	0.0167	1.5E-11	7213	-0.013	0.4408	0.004	0.0008	2E+06	PDXK	aric	0.0063	45.7
rs79039769	A	G	-0.434	0.0211	0.0579	7.7E-14	7213	-0.005	0.019	0.015	0.7483	2E+06	PDXK	aric	0.0077	56.1
rs7911226	G	A	-0.407	0.3042	0.0175	6.5E-116	7213	0.004	0.315	0.004	0.3618	2E+06	FAS	aric	0.0700	543.1
rs79609374	G	A	-0.114	0.3295	0.0177	1.2E-10	7213	-0.010	0.3249	0.004	0.0138	2E+06	AMIGO2	aric	0.0057	41.5
rs4609582	A	G	-0.396	0.3115	0.0174	8.5E-111	7213	0.000	0.2961	0.004	0.9298	2E+06	SAA1	aric	0.0670	517.9
rs79669707	A	G	0.435	0.1178	0.0253	4.1E-65	7213	-0.003	0.1321	0.006	0.6303	2E+06	SAA1	aric	0.0395	296.2
rs1683696	G	T	-0.115	0.4918	0.0167	6.1E-12	7213	0.002	0.4979	0.004	0.5564	2E+06	MEM132(	aric	0.0065	47.5
rs7973997	T	C	-0.318	0.3548	0.0170	1.6E-76	7213	0.007	0.3566	0.004	0.0818	2E+06	MEM132(	aric	0.0464	351.0
rs79744555	C	T	0.652	0.0837	0.0289	9.4E-109	7213	-0.024	0.0842	0.007	0.0004	2E+06	NPTX1	aric	0.0658	507.8
rs4572299	A	G	0.365	0.3084	0.0174	1.3E-94	7213	-0.006	0.2974	0.004	0.1828	2E+06	RNASE6	aric	0.0573	438.5
rs80155227	A	G	-0.681	0.0284	0.0494	8.0E-43	7213	-0.015	0.0293	0.012	0.2139	2E+06	NT5C	aric	0.0258	190.6
rs80238657	G	A	-0.855	0.0258	0.0517	1.9E-60	7213	0.003	0.0245	0.012	0.8027	2E+06	F7	aric	0.0366	274.0
rs80241012	A	G	-0.273	0.1329	0.0242	3.3E-29	7213	-0.001	0.1373	0.006	0.8693	2E+06	CHI3L1	aric	0.0173	127.0
rs486890	A	G	-0.229	0.3381	0.0173	1.3E-39	7213	0.001	0.3459	0.004	0.7862	2E+06	CLEC12A	aric	0.0238	175.6
rs80338457	G	A	-0.502	0.0200	0.0585	1.2E-17	7213	-0.022	0.02	0.014	0.1225	2E+06	CLEC12A	aric	0.0101	73.6
rs8034057	A	G	-0.444	0.0934	0.0280	1.0E-55	7213	0.003	0.0943	0.006	0.6738	2E+06	IGDCC4	aric	0.0337	251.5
rs8177107	G	A	-0.624	0.1818	0.0203	2.3E-194	7213	0.001	0.1778	0.005	0.9106	2E+06	EPHB6	aric	0.1154	940.8
rs8177399	T	C	-1.355	0.0238	0.0519	1.5E-143	7213	-0.011	0.0242	0.012	0.3538	2E+06	TIRAP	aric	0.0863	680.9
rs8178290	A	C	-0.266	0.2150	0.0200	4.7E-40	7213	-0.004	0.2367	0.004	0.3508	2E+06	LPO	aric	0.0240	177.6
rs8178414	T	C	-0.670	0.0117	0.0765	2.4E-18	7213	-0.008	0.0195	0.015	0.5675	2E+06	MPO	aric	0.0105	76.7
rs10836121	C	T	0.114	0.4697	0.0167	1.1E-11	7213	0.003	0.4764	0.004	0.3720	2E+06	CD59	aric	0.0064	46.3
rs831630	T	C	-0.394	0.3090	0.0172	4.2E-112	7213	-0.001	0.3206	0.004	0.7961	2E+06	CD59	aric	0.0678	524.3
rs835344	A	G	0.420	0.4420	0.0161	1.6E-144	7213	-0.009	0.4407	0.004	0.0125	2E+06	GPX7	aric	0.0868	685.8
rs844124	T	C	0.119	0.3148	0.0178	2.3E-11	7213	0.013	0.3088	0.004	0.0015	2E+06	PCBD1	aric	0.0062	44.9
rs883138	C	A	0.493	0.2645	0.0182	1.9E-154	7213	0.002	0.262	0.004	0.6249	2E+06	RARRES2	aric	0.0926	735.9
rs9332575	C	T	-0.624	0.1097	0.0255	3.1E-127	7213	0.002	0.1121	0.006	0.6871	2E+06	SELP	aric	0.0767	599.3
rs9462450	C	T	-0.153	0.1133	0.0262	4.8E-09	7213	-0.015	0.112	0.006	0.0128	2E+06	GLO1	aric	0.0047	34.3
rs9488842	C	A	-0.362	0.2658	0.0184	1.9E-84	7213	0.004	0.2614	0.004	0.3454	2E+06	COL10A1	aric	0.0512	389.2
rs9532075	T	G	0.236	0.3643	0.0171	9.9E-43	7213	-0.005	0.3678	0.004	0.2324	2E+06	POSTN	aric	0.0257	190.2
rs9594222	T	C	0.155	0.1033	0.0274	1.6E-08	7213	0.008	0.1139	0.006	0.2054	2E+06	POSTN	aric	0.0044	31.9
rs9547175	T	C	-0.345	0.2207	0.0194	2.8E-69	7213	0.007	0.2248	0.005	0.1631	2E+06	SLITRK6	aric	0.0420	316.2

rs9811674	G	A	0.375	0.1004	0.0276	1.1E-41	7213	-0.004	0.1054	0.006	0.5699	2E+06	IL1RAP	aric	0.0250	185.2
rs9860775	T	C	1.197	0.0610	0.0321	2.1E-278	7213	-0.009	0.0656	0.007	0.2214	2E+06	POGLUT1	aric	0.1616	1389.8
rs9862503	G	A	0.412	0.3657	0.0165	3.3E-132	7213	-0.013	0.355	0.004	0.0006	2E+06	IL17RD	aric	0.0796	624.1
rs9890200	C	A	-0.674	0.3693	0.0153	0.0E+00	7213	-0.017	0.3616	0.004	0.0000	2E+06	SPATA20	aric	0.2131	1952.7
rs9900613	T	C	-0.450	0.4452	0.0159	7.7E-168	7213	0.002	0.4242	0.004	0.5522	2E+06	MXRA7	aric	0.1003	804.1
rs995946	T	C	0.228	0.0445	0.0404	1.7E-08	7213	-0.007	0.0465	0.009	0.4111	2E+06	PLA2G7	aric	0.0044	31.9
rs1485803	G	A	0.157	0.4298	0.0167	6.0E-21	7213	0.008	0.4434	0.004	0.0248	2E+06	CHST9	aric	0.0121	88.7
rs9961915	T	C	0.301	0.3234	0.0175	6.4E-65	7213	-0.002	0.3141	0.004	0.5788	2E+06	CHST9	aric	0.0393	295.3
rs1023264	C	T	0.176	0.3259	0.0179	8.3E-23	7213	0.001	0.3241	0.004	0.8105	2E+06	CXCL12	aric	0.0133	97.3
rs10793518	A	G	-0.132	0.1808	0.0215	9.9E-10	7213	-0.004	0.184	0.005	0.4126	2E+06	CXCL12	aric	0.0052	37.4
rs1031707	T	C	-0.560	0.2785	0.0173	5.4E-214	7213	0.003	0.2741	0.004	0.5504	2E+06	PPIC	aric	0.1264	1043.5
rs10435378	G	A	-0.192	0.4472	0.0166	1.4E-30	7213	0.001	0.4419	0.004	0.8440	2E+06	MET	aric	0.0182	133.4
rs1060442	A	G	0.140	0.3740	0.0173	6.5E-16	7213	-0.002	0.3854	0.004	0.6131	2E+06	PRTN3	aric	0.0090	65.6
rs10749451	C	T	0.204	0.4689	0.0164	4.0E-35	7213	0.015	0.4631	0.004	0.0001	2E+06	PLEKHA1	aric	0.0210	154.5
rs10752113	C	T	-0.345	0.2355	0.0191	3.7E-71	7213	0.007	0.2384	0.004	0.1238	2E+06	ITIH5	aric	0.0431	325.2
rs10766205	G	A	-0.300	0.2716	0.0185	7.9E-58	7213	-0.004	0.2626	0.004	0.2935	2E+06	CALCB	aric	0.0350	261.5
rs10776914	T	C	0.264	0.2969	0.0180	6.6E-48	7213	0.004	0.3207	0.004	0.3092	2E+06	FCN1	aric	0.0289	214.6
rs10894353	C	T	-0.336	0.4490	0.0164	5.5E-91	7213	-0.005	0.4472	0.004	0.2207	2E+06	NTM	aric	0.0552	420.9
rs10901246	G	T	-0.327	0.2036	0.0203	2.8E-57	7213	0.010	0.2275	0.005	0.0299	2E+06	OBP2B	aric	0.0347	258.9
rs11156734	C	T	0.459	0.2597	0.0182	1.6E-134	7213	-0.007	0.2596	0.004	0.1012	2E+06	RNASE3	aric	0.0810	635.6
rs1130866	G	A	0.517	0.4815	0.0155	7.4E-228	7213	0.000	0.4762	0.004	0.9569	2E+06	SFTPB	aric	0.1341	1116.9
rs11664471	A	C	0.131	0.4287	0.0168	5.2E-15	7213	-0.002	0.4205	0.004	0.6326	2E+06	DSC2	aric	0.0084	61.4
rs1658125	A	G	0.382	0.1656	0.0218	1.3E-67	7213	0.002	0.1587	0.005	0.6571	2E+06	DSC2	aric	0.0410	308.2
rs12493131	T	G	-0.436	0.0991	0.0272	5.5E-57	7213	0.002	0.1096	0.006	0.7420	2E+06	FAM3D	aric	0.0345	257.5
rs1250258	C	T	-0.285	0.2666	0.0186	2.2E-52	7213	0.007	0.259	0.004	0.0965	2E+06	FN1	aric	0.0317	235.8
rs12897030	C	T	-0.390	0.3103	0.0175	4.3E-106	7213	0.000	0.315	0.004	0.9281	2E+06	RNASE1	aric	0.0642	494.8
rs151064	T	G	-0.895	0.1625	0.0199	0.0E+00	7213	0.006	0.1566	0.005	0.2249	2E+06	ADAMTS5	aric	0.2184	2014.9
rs1884080	T	G	0.143	0.2872	0.0183	7.2E-15	7213	-0.003	0.2888	0.004	0.5310	2E+06	SERPINA4	aric	0.0084	60.8
rs1935451	G	A	0.270	0.0853	0.0297	1.3E-19	7213	0.012	0.0902	0.007	0.0613	2E+06	UROS	aric	0.0113	82.5
rs1958078	A	C	-0.516	0.1571	0.0219	6.3E-118	7213	-0.002	0.1536	0.005	0.6758	2E+06	SMOC1	aric	0.0712	553.1
rs2085765	A	G	0.178	0.1162	0.0256	3.4E-12	7213	0.005	0.1188	0.006	0.3456	2E+06	SCARA5	aric	0.0067	48.6
rs2144693	T	C	-0.517	0.3831	0.0159	4.7E-216	7213	-0.012	0.3728	0.004	0.0014	2E+06	GSTA1	aric	0.1276	1054.4

rs2229498	G	A	-0.501	0.1578	0.0221	3.5E-110	7213	-0.004	0.1577	0.005	0.4936	2E+06	SRGN	aric	0.0666	514.8
rs2465403	G	A	-0.137	0.3833	0.0170	9.2E-16	7213	0.010	0.3928	0.004	0.0092	2E+06	TNFRSF11E	aric	0.0089	64.9
rs2586528	G	T	0.298	0.2744	0.0183	1.1E-58	7213	0.013	0.2928	0.004	0.0014	2E+06	ENO3	aric	0.0355	265.7
rs2766575	T	C	-0.379	0.4018	0.0165	1.6E-112	7213	-0.012	0.4102	0.004	0.0021	2E+06	CLPS	aric	0.0680	526.4
rs2842970	C	T	0.334	0.4160	0.0165	4.2E-88	7213	0.002	0.4217	0.004	0.5409	2E+06	ACAT2	aric	0.0534	407.0
rs2846703	G	A	0.149	0.2875	0.0185	1.0E-15	7213	-0.004	0.2893	0.004	0.3700	2E+06	MMP1	aric	0.0089	64.6
rs36086195	C	T	0.371	0.4196	0.0162	1.4E-111	7213	-0.004	0.4083	0.004	0.2799	2E+06	EPHA2	aric	0.0675	521.7
rs3800403	A	G	-0.355	0.1601	0.0225	3.2E-55	7213	0.003	0.1523	0.005	0.6094	2E+06	SCUBE3	aric	0.0334	249.2
rs4073961	G	A	-0.143	0.4856	0.0165	4.3E-18	7213	0.003	0.4789	0.004	0.4471	2E+06	SECTM1	aric	0.0104	75.6
rs4789763	A	G	-0.344	0.4942	0.0162	2.9E-97	7213	-0.004	0.5039	0.004	0.2716	2E+06	SECTM1	aric	0.0589	451.5
rs4346716	T	C	0.450	0.4850	0.0158	1.7E-168	7213	0.001	0.4687	0.004	0.7304	2E+06	MAN2B2	aric	0.1007	807.4
rs4394214	G	T	-0.220	0.2163	0.0199	4.3E-28	7213	-0.007	0.2082	0.005	0.1345	2E+06	TREM1	aric	0.0166	121.7
rs6914090	T	C	-0.455	0.4993	0.0158	4.2E-172	7213	-0.004	0.5176	0.004	0.2831	2E+06	TREM1	aric	0.1028	825.9
rs4434842	T	G	-0.313	0.0473	0.0393	2.0E-15	7213	-0.007	0.0585	0.008	0.4063	2E+06	AMY1A	aric	0.0087	63.4
rs4457570	A	G	-0.518	0.3799	0.0160	3.3E-215	7213	0.006	0.3784	0.004	0.1599	2E+06	CRABP2	aric	0.1271	1049.9
rs4665681	G	A	-0.953	0.0281	0.0492	2.1E-81	7213	-0.018	0.0281	0.012	0.1328	2E+06	TP53I3	aric	0.0494	374.5
rs4679138	G	A	-0.427	0.3147	0.0171	2.9E-132	7213	0.001	0.3113	0.004	0.8155	2E+06	PLXNA1	aric	0.0797	624.3
rs4704826	C	A	0.204	0.3651	0.0171	1.6E-32	7213	0.003	0.3669	0.004	0.4076	2E+06	TIMD4	aric	0.0194	142.4
rs470530	A	G	-0.642	0.1341	0.0233	7.0E-159	7213	0.013	0.1539	0.005	0.0127	2E+06	MMP12	aric	0.0952	758.5
rs4733505	A	G	-0.171	0.3495	0.0171	2.6E-23	7213	-0.006	0.3327	0.004	0.1727	2E+06	GSR	aric	0.0136	99.6
rs4767461	C	T	-0.163	0.2558	0.0191	1.3E-17	7213	-0.001	0.2555	0.004	0.7766	2E+06	TESC	aric	0.0101	73.3
rs4783718	T	C	-0.301	0.4003	0.0167	7.7E-71	7213	0.035	0.4009	0.004	0.0000	2E+06	NQO1	aric	0.0430	323.7
rs4859610	A	G	0.397	0.2259	0.0191	5.8E-93	7213	0.008	0.2313	0.004	0.0612	2E+06	ART3	aric	0.0563	430.6
rs5751764	A	C	0.132	0.4158	0.0169	5.0E-15	7213	-0.002	0.4124	0.004	0.6575	2E+06	GSTT2B	aric	0.0085	61.5
rs5756736	G	A	0.289	0.4143	0.0166	4.8E-67	7213	0.006	0.4115	0.004	0.1526	2E+06	LGALS2	aric	0.0406	305.5
rs6655975	A	G	0.390	0.3475	0.0167	3.8E-116	7213	-0.015	0.3487	0.004	0.0001	2E+06	CTSS	aric	0.0702	544.2
rs6663887	A	G	-0.179	0.2655	0.0186	7.4E-22	7213	0.003	0.2552	0.004	0.4918	2E+06	CR1	aric	0.0127	92.9
rs6892697	G	A	0.387	0.3277	0.0172	1.9E-108	7213	-0.010	0.3459	0.004	0.0124	2E+06	TGFBI	aric	0.0656	506.3
rs6906620	A	C	0.331	0.2518	0.0189	1.4E-67	7213	0.000	0.2449	0.004	0.9563	2E+06	ENPP5	aric	0.0410	308.0
rs6920109	T	C	0.174	0.2346	0.0196	9.8E-19	7213	0.003	0.2491	0.004	0.5242	2E+06	UST	aric	0.0108	78.5
rs7032104	C	T	0.305	0.4443	0.0164	1.6E-75	7213	-0.007	0.4503	0.004	0.0760	2E+06	C8G	aric	0.0458	346.1
rs7647776	C	A	-0.558	0.2633	0.0178	6.8E-203	7213	-0.006	0.2809	0.004	0.1651	2E+06	AHSG	aric	0.1202	985.3

rs7667751	C	T	-0.446	0.3067	0.0174	6.1E-139	7213	-0.004	0.3136	0.004	0.3854	2E+06	UGDH	aric	0.0836	657.8
rs7970720	G	T	-0.439	0.1704	0.0216	5.8E-89	7213	-0.005	0.1826	0.005	0.3402	2E+06	C1R	aric	0.0539	411.1
rs809066	A	G	-0.547	0.0903	0.0281	5.6E-82	7213	0.003	0.0937	0.007	0.6792	2E+06	CROT	aric	0.0497	377.3
rs840870	G	T	0.246	0.4133	0.0165	2.8E-49	7213	0.004	0.4119	0.004	0.3165	2E+06	HS6ST1	aric	0.0297	221.0
rs854784	C	T	-0.313	0.4791	0.0161	1.8E-82	7213	0.014	0.4549	0.004	0.0001	2E+06	SHMT1	aric	0.0500	379.7
rs858523	C	T	0.160	0.4172	0.0168	2.2E-21	7213	0.009	0.4415	0.004	0.0174	2E+06	SAT2	aric	0.0124	90.7
rs9384738	T	C	-0.289	0.0919	0.0285	4.0E-24	7213	-0.004	0.092	0.007	0.5146	2E+06	METTL24	aric	0.0141	103.3

Supplementary Table 2. Primers used for qRT-PCR

Gene		From 5’ to 3’
18s	Forward:	CGCCGCTAGAGGTGCAATTC
	Reverse:	CCAGTCGGCATCGTTTATGG
Pam	Forward:	CTGGGGTCACACCTAAAGAGT
	Reverse:	ATGAGGGCATGTTGCATCCAA

Supplementary Table3. Antibodies used for Western blot

Antibody	Source	Catalog No.
Alpha Tubulin	Proteintech	Cat# 11224-1-AP
Beta Actin	Proteintech	Cat# 66009-1-Ig
Anti-PDX1	Santa	Cat# sc-390808
Anti-GLUT2	Santa	Cat# sc-518022
Anti-Bcl2	ZENBIO	Cat# 383309
Anti-Mouse	ZENBIO	Cat# 511103
Anti-Cyclin D1	HUABIO	Cat# ET1601-31

Supplementary Table 4. MR steiger test between 90 plasma proteins levels and T2D.

id.exposure	exposure	outcome	snp_r2 exposure	snp_r2 outcome	correct causal. directio	steiger pval
10714_7_ACE_ACE_filtered	ACE	outcome	0.04268	4.526E-06	TRUE	0
10832_24_B4GALT6_B4GT6_filtered	B4GALT6	outcome	0.03791	4.661E-06	TRUE	8.6193E-289
10833_64_HHIP_HHIP_filtered	HHIP	outcome	0.00881	2.291E-05	TRUE	3.52013E-62
11102_22_REG4_REG4_filtered	REG4	outcome	0.00200	6.724E-06	TRUE	4.49781E-15
11103_24_HSPB1_HSP_27_filtered	HSPB1	outcome	0.03027	5.807E-06	TRUE	1.6272E-228
11109_56_SVEP1_SVEP1_filtered	SVEP1	outcome	0.04084	1.189E-05	TRUE	4.7788E-308
11117_2_SPATA20_SPT20_filtered	SPATA20	outcome	0.02552	1.113E-05	TRUE	9.6404E-190
11145_72_KIAA1549L_K154L_filtered	KIAA1549L	outcome	0.03229	1.081E-06	TRUE	5.089E-248
11219_95_FGFBP3_FGFP3_filtered	FGFBP3	outcome	0.02551	1.339E-05	TRUE	8.3908E-188
11313_100_PCBD1_PHS_filtered	PCBD1	outcome	0.00622	5.559E-06	TRUE	2.73E-46
11369_23_ADH5_ADHX_filtered	ADH5	outcome	0.01203	6.546E-06	TRUE	3.30175E-89
12334_25_SHMT1_cSHMT_filtered	SHMT1	outcome	0.02552	8.262E-06	TRUE	7.607E-191
12396_19_HIBCH_HIBCH_filtered	HIBCH	outcome	0.02551	9.513E-06	TRUE	2.4363E-190
12436_84_GSTO1_GST_omega_1_filtered	GSTO1	outcome	0.05102	1.437E-06	TRUE	0
12459_13_PLEKHA1_PKHA1_filtered	PLEKHA1	outcome	0.02550	8.782E-06	TRUE	1.257E-190
12517_52_PDCD5_TFAR19_filtered	PDCD5	outcome	0.02551	7.884E-06	TRUE	5.3063E-191
13676_46_INHBB_Inhibin_bB_chain	INHBB	outcome	0.02551	7.616E-06	TRUE	4.0745E-191
13717_15_FCN2_FCN2	FCN2	outcome	0.03211	1.321E-06	TRUE	4.0305E-246
14107_1_MTHFS_MTHFS	MTHFS	outcome	0.02807	8.286E-06	TRUE	1.8047E-210
15363_32_APOA5_Apo_A_V	APOA5	outcome	0.01244	8.360E-06	TRUE	7.9741E-92
15388_24_FCGR3A_FcRIIIa	FCGR3A	outcome	0.04291	9.864E-08	TRUE	0
15521_4_CLSTN1_Alcadein_alpha_1	CLSTN1	outcome	0.03388	4.707E-08	TRUE	5.3131E-263
16616_137_ENO3_ENOB	ENO3	outcome	0.02550	5.631E-06	TRUE	4.8958E-192
16809_1_NME4_NDKM	NME4	outcome	0.01242	5.864E-06	TRUE	2.73643E-92

16914_104_CD14_sCD14	CD14	outcome	0.02463	4.250E-06	TRUE	5.6915E-186
17138_8_GSTA1_GST_A1_1	GSTA1	outcome	0.02551	5.619E-06	TRUE	4.7985E-192
17224_12_OGN_MIME	OGN	outcome	0.03427	4.900E-06	TRUE	3.3195E-260
17329_2_BDH2_BDH2	BDH2	outcome	0.01993	3.564E-05	TRUE	8.6321E-142
17456_53_GOLM1_GOLM1	GOLM1	outcome	0.02462	2.054E-07	TRUE	1.3502E-189
17680_12_EPHB1_EPHB1	EPHB1	outcome	0.03089	2.408E-06	TRUE	1.035E-235
18235_16_PGP_PGP_filtered	PGP	outcome	0.01382	6.950E-06	TRUE	4.963E-102
18340_2_PSMB4_Proteasome_beta_chain	PSMB4	outcome	0.02551	6.851E-06	TRUE	1.8645E-191
18831_6_LRIG1_LRIG1	LRIG1	outcome	0.02901	4.913E-07	TRUE	2.8958E-223
18864_7_PRSS3_TRY3	PRSS3	outcome	0.02552	1.449E-05	TRUE	1.4704E-188
18878_15_GREM1_GREM1	GREM1	outcome	0.03586	1.319E-06	TRUE	4.9069E-276
19130_81_SERPINB8_SPB8	SERPINB8	outcome	0.02603	1.829E-06	TRUE	1.7174E-198
19277_4_TSTD1_KAT	TSTD1	outcome	0.02707	6.665E-07	TRUE	6.9186E-208
19561_216_PLXND1_PLXD1	PLXND1	outcome	0.02181	1.776E-05	TRUE	2.0012E-159
19590_46_SFTPD_SP_D	SFTPD	outcome	0.05102	2.696E-06	TRUE	0
19617_5_PTGR1_LTB4DH	PTGR1	outcome	0.04642	1.469E-06	TRUE	0
2677_1_EGFR_ERBB1	EGFR	outcome	0.00549	3.295E-06	TRUE	2.18107E-41
2843_13_SPINT2_SPINT2	SPINT2	outcome	0.03243	2.471E-07	TRUE	9.7762E-251
2948_58_GHR_Growth_hormone_receptor	GHR	outcome	0.01297	8.208E-06	TRUE	1.79944E-95
3041_55_MRC2_MRC2	MRC2	outcome	0.01062	8.929E-06	TRUE	4.59097E-78
3045_72_PTN_PTN	PTN	outcome	0.01836	1.143E-05	TRUE	1.9696E-135
3054_3_HP_Haptoglobin__Mixed_Type	HP	outcome	0.03259	5.836E-09	TRUE	4.1574E-253
3060_43_C9_C9	C9	outcome	0.00441	5.700E-07	TRUE	1.67932E-34
3181_50_CTSS_Cathepsin_S	CTSS	outcome	0.01437	8.342E-06	TRUE	2.7861E-106
3299_29_CNTN5_Contactin_5	CNTN5	outcome	0.02429	6.767E-06	TRUE	4.332E-182
3310_62_FCGR2B_FCG2B	FCGR2B	outcome	0.05103	1.086E-06	TRUE	0
3320_49_IGFBP7_IGFBP_7	IGFBP7	outcome	0.03002	3.192E-06	TRUE	2.5769E-228
3376_49_IL17RD_IL_17_RD	IL17RD	outcome	0.02551	6.451E-06	TRUE	1.2154E-191
3805_16_ESM1_Endocan	ESM1	outcome	0.00973	3.646E-07	TRUE	6.51733E-75
4328_2_BOC_BOC	BOC	outcome	0.01628	1.356E-05	TRUE	4.1373E-119
4479_14_SERPING1_C1_Esterase_Inhibitor	SERPING1	outcome	0.03716	2.004E-06	TRUE	3.0843E-285
4498_62_NCAM1_NCAM_120	NCAM1	outcome	0.04436	7.290E-06	TRUE	0
4541_49_CDON_CDON	CDON	outcome	0.03149	5.206E-06	TRUE	1.5369E-238
4908_6_ENG_Endoglin	ENG	outcome	0.01335	5.924E-06	TRUE	2.38403E-99
4968_50_CAPG_CAPG	CAPG	outcome	0.03968	1.060E-07	TRUE	0
5231_79_PCSK9_PCSK9	PCSK9	outcome	0.02415	1.876E-06	TRUE	6.0732E-184
5581_28_FGL1_FGL1	FGL1	outcome	0.02550	1.669E-05	TRUE	5.8388E-188
5593_11_PDIA5_PDIA5	PDIA5	outcome	0.02962	8.638E-08	TRUE	3.4916E-229
5620_13_PAM_AMD	PAM	outcome	0.02552	7.869E-06	TRUE	5.1921E-191
5628_21_SEMA3G_SEM3G	SEMA3G	outcome	0.01329	8.301E-06	TRUE	3.85821E-98
5631_83_MLN_MOTI	MLN	outcome	0.04752	1.468E-05	TRUE	0
5703_26_OLFM1_NOE1	OLFM1	outcome	0.00354	8.436E-06	TRUE	5.06567E-26

5708_1_LEAP2_LEAP2	LEAP2	outcome	0.02552	1.268E-05	TRUE	3.2886E-189
5749_53_CLPS_COL	CLPS	outcome	0.02552	5.223E-06	TRUE	2.9863E-192
6086_15_CHRDL2_CRDL2	CHRDL2	outcome	0.03483	6.359E-06	TRUE	7.492E-264
6342_10_NPNT_Nephronectin	NPNT	outcome	0.01272	5.636E-06	TRUE	2.21178E-94
6366_38_TXNDC15_TXD15	TXNDC15	outcome	0.02551	6.816E-06	TRUE	1.7993E-191
6388_21_CCDC126_CC126	CCDC126	outcome	0.02550	8.848E-06	TRUE	1.3357E-190
6393_63_HSP90B1_Endoplasmin	HSP90B1	outcome	0.05058	1.902E-06	TRUE	0
6415_90_CPN2_CPN2	CPN2	outcome	0.02888	4.288E-07	TRUE	5.0572E-222
6947_4_ST3GAL6_SIA10	ST3GAL6	outcome	0.02658	2.799E-08	TRUE	3.3495E-205
7156_2_FUT10_FUT10	FUT10	outcome	0.05540	2.088E-06	TRUE	0
7173_141_TMEM132C_T132C	TMEM132C	outcome	0.02663	1.862E-06	TRUE	3.2246E-203
7206_20_FBP1_F16P1	FBP1	outcome	0.00084	1.294E-05	TRUE	2.20368E-06
7933_75_ADAM22_ADA22	ADAM22	outcome	0.04156	1.523E-06	TRUE	0
8039_41_FAM177A1_F177A	FAM177A1	outcome	0.02551	6.282E-06	TRUE	1.0162E-191
8773_172_EMILIN3_EMIL3	EMILIN3	outcome	0.00660	2.181E-05	TRUE	2.43324E-46
8866_53_QPCTL_QPCTL	QPCTL	outcome	0.02551	1.702E-05	TRUE	3.419E-187
8894_80_HNRNPAB_hnRNP_A_B	HNRNPAB	outcome	0.00200	6.225E-06	TRUE	3.8475E-15
9256_78_NPTX1_NPTX1	NPTX1	outcome	0.02551	6.915E-06	TRUE	1.9932E-191
9267_2_CPA4_CBPA4	CPA4	outcome	0.05103	7.362E-06	TRUE	0
9385_4_GAA_GAA	GAA	outcome	0.02727	5.004E-07	TRUE	7.3352E-210
9484_75_DSG2_Desmoglein_2	DSG2	outcome	0.01769	3.568E-07	TRUE	8.0571E-136
9580_5_LAMC2_Laminin_gamma_2	LAMC2	outcome	0.02551	1.446E-05	TRUE	1.2377E-188
9754_33_NQO2_Quinone_reductase_2	NQO2	outcome	0.05102	3.827E-06	TRUE	0
9837_60_NQO1_NAD_P_H_dehydrogenase	NQO1	outcome	0.02550	4.851E-05	TRUE	3.341E-181
SeqId_10714_7	ACE	outcome	0.06427	4.526E-06	TRUE	3.3949E-105
SeqId_10832_24	B4GALT6	outcome	0.12462	4.661E-06	TRUE	4.9026E-212
SeqId_10833_64	HHIP	outcome	0.01481	2.291E-05	TRUE	2.30398E-23
SeqId_11102_22	REG4	outcome	0.00488	6.724E-06	TRUE	1.146E-08
SeqId_11103_24	HSPB1	outcome	0.12569	5.807E-06	TRUE	9.9012E-214
SeqId_11109_56	SVEP1	outcome	0.07571	1.189E-05	TRUE	1.4329E-123
SeqId_11117_2	SPATA20	outcome	0.00000	1.113E-05	FALSE	0.777355759
SeqId_11145_72	KIAA1549L	outcome	0.10426	1.081E-06	TRUE	4.5356E-176
SeqId_11219_95	FGFBP3	outcome	0.15408	1.339E-05	TRUE	1.34E-265
SeqId_11313_100	PCBD1	outcome	0.00618	5.559E-06	TRUE	9.2763E-11
SeqId_11369_23	ADH5	outcome	0.09638	6.546E-06	TRUE	1.9975E-160
SeqId_12334_25	SHMT1	outcome	0.05003	8.262E-06	TRUE	8.42597E-81
SeqId_12396_19	HIBCH	outcome	0.17270	9.513E-06	TRUE	2.8819E-303
SeqId_12436_84	GSTO1	outcome	0.12263	1.437E-06	TRUE	1.9096E-209
SeqId_12459_13	PLEKHA1	outcome	0.02098	8.782E-06	TRUE	9.1866E-34
SeqId_12517_52	PDCD5	outcome	0.18454	7.884E-06	TRUE	0

SeqId_13676_46	INHBB	outcome	0.05548	7.616E-06	TRUE	6.21155E-90
SeqId_13717_15	FCN2	outcome	0.04566	1.321E-06	TRUE	9.19447E-75
SeqId_14107_1	MTHFS	outcome	0.10293	8.286E-06	TRUE	8.9507E-172
SeqId_15363_32	APOA5	outcome	0.01468	8.360E-06	TRUE	7.34882E-24
SeqId_15388_24	FCGR3A	outcome	0.02979	9.864E-08	TRUE	3.17606E-49
SeqId_15521_4	CLSTN1	outcome	0.07505	4.707E-08	TRUE	2.9211E-125
SeqId_16616_137	ENO3	outcome	0.03554	5.631E-06	TRUE	2.10551E-57
SeqId_16809_1	NME4	outcome	0.01743	5.864E-06	TRUE	2.19905E-28
SeqId_16914_104	CD14	outcome	0.03913	4.250E-06	TRUE	1.94904E-63
SeqId_17138_8	GSTA1	outcome	0.12760	5.619E-06	TRUE	2.5123E-217
SeqId_17224_12	OGN	outcome	0.07723	4.900E-06	TRUE	2.9347E-127
SeqId_17329_2	BDH2	outcome	0.03836	3.564E-05	TRUE	8.45159E-60
SeqId_17456_53	GOLM1	outcome	0.05848	2.054E-07	TRUE	1.04974E-96
SeqId_17680_12	EPHB1	outcome	0.07704	2.408E-06	TRUE	1.5587E-127
SeqId_18235_16	PGP	outcome	0.05489	6.950E-06	TRUE	5.63659E-89
SeqId_18340_2	PSMB4	outcome	0.07051	6.851E-06	TRUE	2.1109E-115
SeqId_18831_6	LRIG1	outcome	0.04352	4.913E-07	TRUE	1.59053E-71
SeqId_18864_7	PRSS3	outcome	0.05131	1.449E-05	TRUE	3.01813E-82
SeqId_18878_15	GREM1	outcome	0.01322	1.319E-06	TRUE	3.37643E-22
SeqId_19130_81	SERPINB8	outcome	0.11442	1.829E-06	TRUE	3.5599E-194
SeqId_19277_4	TSTD1	outcome	0.18419	6.665E-07	TRUE	0
SeqId_19561_216	PLXND1	outcome	0.06059	1.776E-05	TRUE	2.40187E-97
SeqId_19590_46	SFTPD	outcome	0.06136	2.696E-06	TRUE	1.16785E-100
SeqId_19617_5	PTGR1	outcome	0.06173	1.469E-06	TRUE	1.3501E-101
SeqId_2677_1	EGFR	outcome	0.02030	3.295E-06	TRUE	3.43883E-33
SeqId_2843_13	SPINT2	outcome	0.08858	2.471E-07	TRUE	1.1733E-148
SeqId_2948_58	GHR	outcome	0.04614	8.208E-06	TRUE	2.41313E-74
SeqId_3041_55	MRC2	outcome	0.05349	8.929E-06	TRUE	1.88103E-86
SeqId_3045_72	PTN	outcome	0.04422	1.143E-05	TRUE	6.71751E-71
SeqId_3054_3	HP	outcome	0.02429	5.836E-09	TRUE	2.02571E-40
SeqId_3060_43	C9	outcome	0.01691	5.700E-07	TRUE	3.13267E-28
SeqId_3181_50	CTSS	outcome	0.07020	8.342E-06	TRUE	1.2477E-114
SeqId_3299_29	CNTN5	outcome	0.07622	6.767E-06	TRUE	3.4711E-125
SeqId_3310_62	FCGR2B	outcome	0.02516	1.086E-06	TRUE	2.41815E-41
SeqId_3320_49	IGFBP7	outcome	0.10572	3.192E-06	TRUE	6.3194E-178
SeqId_3376_49	IL17RD	outcome	0.07967	6.451E-06	TRUE	3.4279E-131
SeqId_3805_16	ESM1	outcome	0.01737	3.646E-07	TRUE	4.97279E-29
SeqId_4328_2	BOC	outcome	0.03397	1.356E-05	TRUE	4.23785E-54
SeqId_4479_14	SERPING1	outcome	0.09759	2.004E-06	TRUE	1.0062E-163
SeqId_4498_62	NCAM1	outcome	0.11305	7.290E-06	TRUE	3.2428E-190
SeqId_4541_49	CDON	outcome	0.18306	5.206E-06	TRUE	0
SeqId_4908_6	ENG	outcome	0.02934	5.924E-06	TRUE	2.32888E-47
SeqId_4968_50	CAPG	outcome	0.12251	1.060E-07	TRUE	3.1893E-210
SeqId_5231_79	PCSK9	outcome	0.04333	1.876E-06	TRUE	9.03741E-71
SeqId_5581_28	FGL1	outcome	0.07626	1.669E-05	TRUE	5.9835E-124
SeqId_5593_11	PDIA5	outcome	0.00488	8.638E-08	TRUE	3.54468E-09

SeqId_5620_13	PAM	outcome	0.02547	7.869E-06	TRUE	5.72991E-41
SeqId_5628_21	SEMA3G	outcome	0.01887	8.301E-06	TRUE	1.81366E-30
SeqId_5631_83	MLN	outcome	0.04104	1.468E-05	TRUE	1.97951E-65
SeqId_5703_26	OLFM1	outcome	0.00578	8.436E-06	TRUE	5.37378E-10
SeqId_5708_1	LEAP2	outcome	0.04006	1.268E-05	TRUE	5.14892E-64
SeqId_5749_53	CLPS	outcome	0.06805	5.223E-06	TRUE	1.7367E-111
SeqId_6086_15	CHRDL2	outcome	0.05391	6.359E-06	TRUE	1.74091E-87
SeqId_6342_10	NPNT	outcome	0.05579	5.636E-06	TRUE	1.15276E-90
SeqId_6366_38	TXNDC15	outcome	0.05222	6.816E-06	TRUE	1.29932E-84
SeqId_6388_21	CCDC126	outcome	0.08776	8.848E-06	TRUE	7.3463E-145
SeqId_6393_63	HSP90B1	outcome	0.24701	1.902E-06	TRUE	0
SeqId_6415_90	CPN2	outcome	0.09995	4.288E-07	TRUE	1.025E-168
SeqId_6947_4	ST3GAL6	outcome	0.06293	2.799E-08	TRUE	1.8742E-104
SeqId_7156_2	FUT10	outcome	0.08448	2.088E-06	TRUE	1.6056E-140
SeqId_7173_141	TMEM132C	outcome	0.05297	1.862E-06	TRUE	9.09286E-87
SeqId_7206_20	FBP1	outcome	0.00465	1.294E-05	TRUE	4.18597E-08
SeqId_7933_75	ADAM22	outcome	0.10006	1.523E-06	TRUE	2.5122E-168
SeqId_8039_41	FAM177A1	outcome	0.05616	6.282E-06	TRUE	2.90346E-91
SeqId_8773_172	EMILIN3	outcome	0.01360	2.181E-05	TRUE	1.55101E-21
SeqId_8866_53	QPCTL	outcome	0.09854	1.702E-05	TRUE	1.434E-162
SeqId_8894_80	HNRNPAB	outcome	0.00416	6.225E-06	TRUE	1.43633E-07
SeqId_9256_78	NPTX1	outcome	0.06581	6.915E-06	TRUE	2.1684E-107
SeqId_9267_2	CPA4	outcome	0.06881	7.362E-06	TRUE	2.0382E-112
SeqId_9385_4	GAA	outcome	0.05780	5.004E-07	TRUE	2.30036E-95
SeqId_9484_75	DSG2	outcome	0.06802	3.568E-07	TRUE	7.9891E-113
SeqId_9580_5	LAMC2	outcome	0.03971	1.446E-05	TRUE	2.7367E-63
SeqId_9754_33	NQO2	outcome	0.07257	3.827E-06	TRUE	1.8015E-119
SeqId_9837_60	NQO1	outcome	0.04297	4.851E-05	TRUE	1.47734E-66
